# Supplementary material for: A conserved NR5A1-responsive enhancer regulates SRY in testis-determination
Source: Nat Commun. 2024 Mar 30;15:2796. doi: 10.1038/s41467-024-47162-2 (PMC10981742; doi:10.1038/s41467-024-47162-2)
Supplement: Supplementary file 1 — Supplementary_Information.pdf [file 41467_2024_47162_MOESM1_ESM.pdf]

Supplementary Figure 01

Phylogenetic tree describing the evolutionary relationships between the 83 mammalian species shown in Supplementary_Data_03.clc and Supplementary_Data_04.clc.

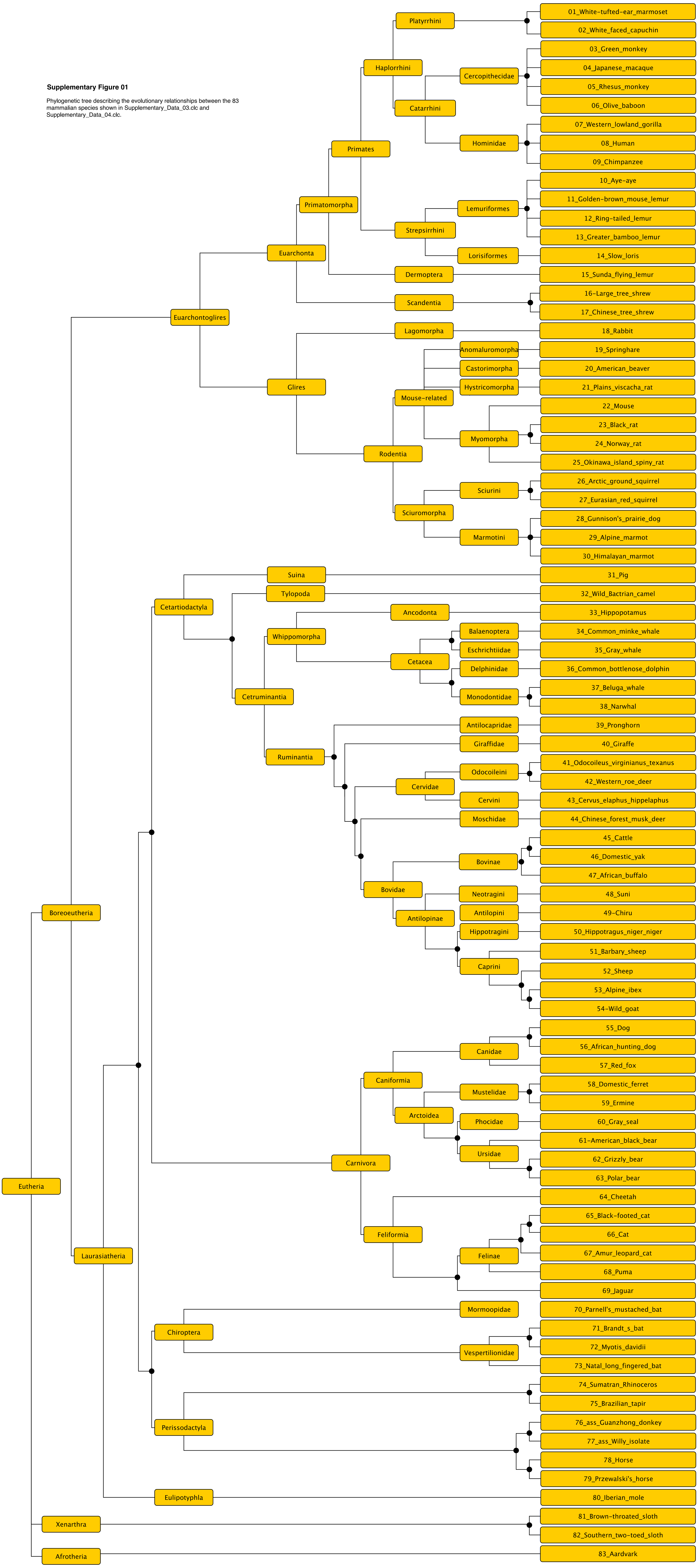

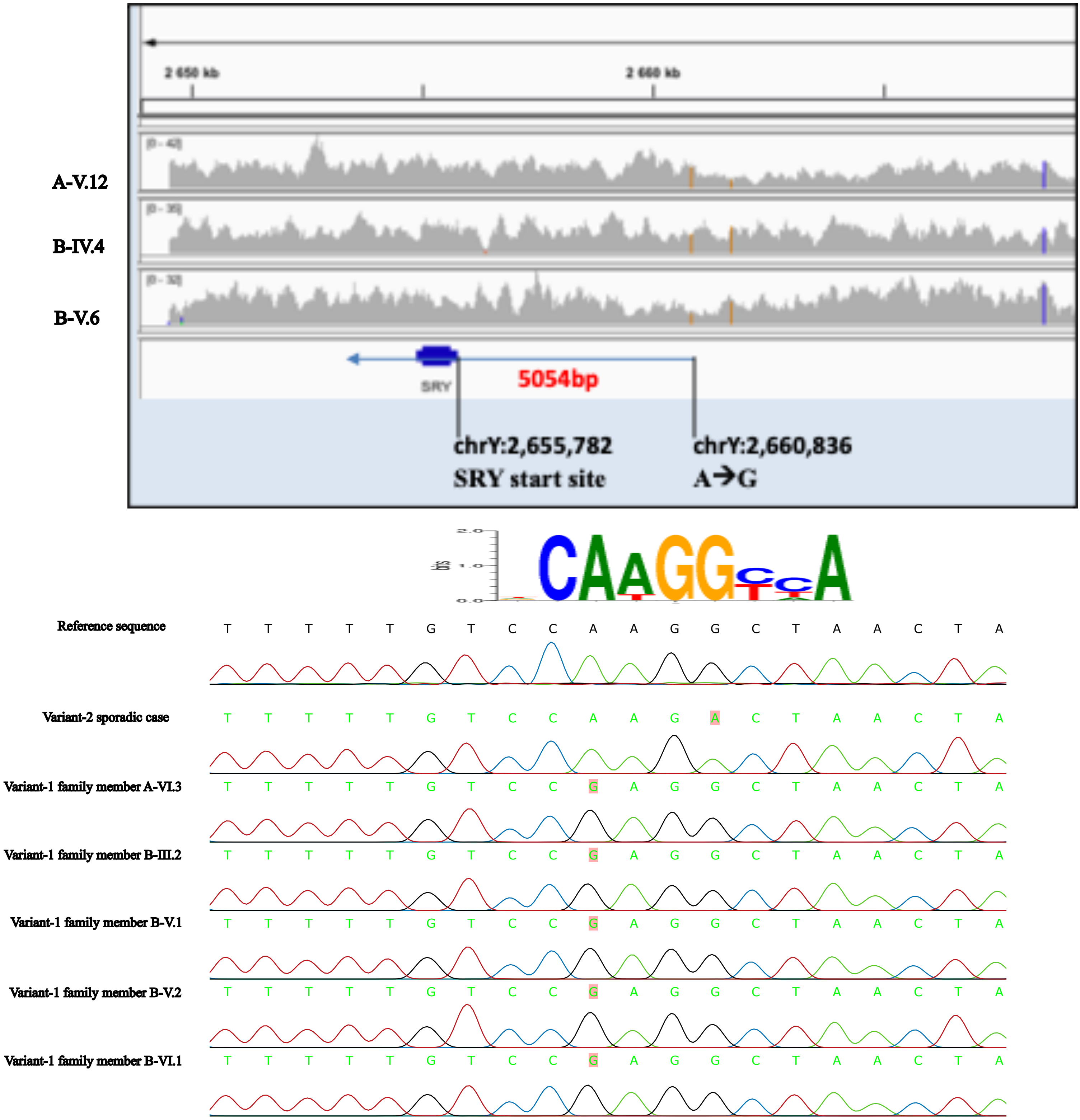

Supplementary Figure 02

Window showing

- the SRY locus from whole genome sequencing (the reference genome is GRC37),

- the Sanger sequencing of 5 members of the Variant-1 family, and

- the Sanger sequencing of the Variant-2 woman with Complete Gonadal Dysgenesis centered on the NR5A1 binding site.

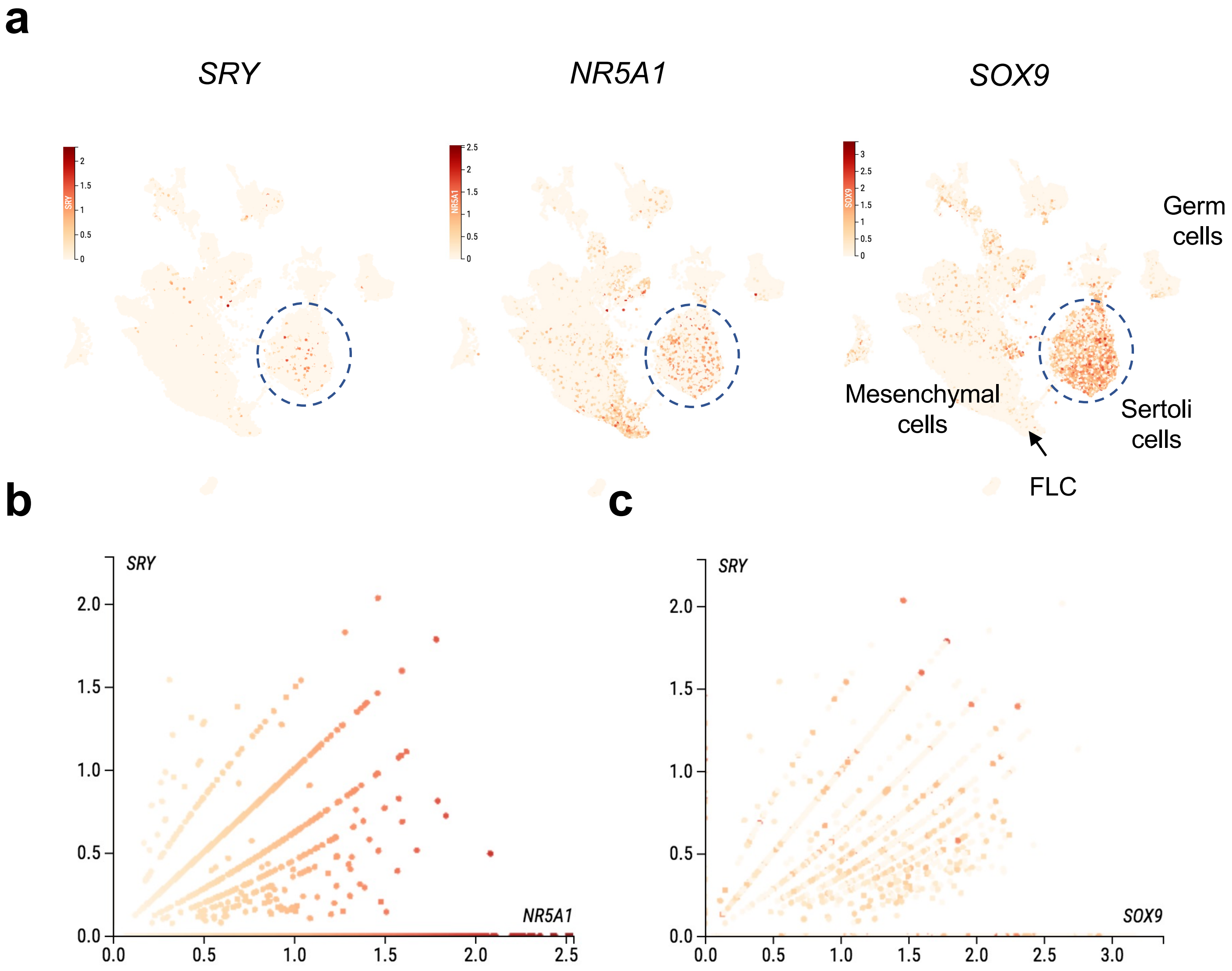

Supplementary Figure 03
Expression of *SRY* and *NR5A1* in human fetal testis using single-cell RNA-sequencing.

a Feature plots of *SRY*, *NR5A1* and *SOX9* showing predominant expression of these genes in the developing Sertoli cell cluster.

b Scatterplot showing the correlation of *SRY* and *NR5A1* expression in single cells.

c Scatterplot showing the correlation of *SRY* and *SOX9* expression in single cells. Analysis represents n=133,181 integrated cells between 7 and 19 weeks post conception.

These data were generated by the Vento-Tormo group at the Wellcome Sanger Institute, Hinxton, UK and can be accessed using CZ CELLxGENE from https://www.reproductivecellatlas.org/gonads/human-main-male/(Garcia-Alonso, L., Lorenzi, V., Mazzeo, C.I., et al. Single-cell roadmap of human gonadal development. Nature 607, 540–547, 2022; https://doi.org/10.1038/s41586-022-04918-4). This work is licensed under a Creative Commons Attribution-BY 4.0 International License (https://creativecommons.org/licenses/by/4.0/). Major cell clusters are shown and detailed annotation is provided in the reference above. FLC, fetal Leydig cells.

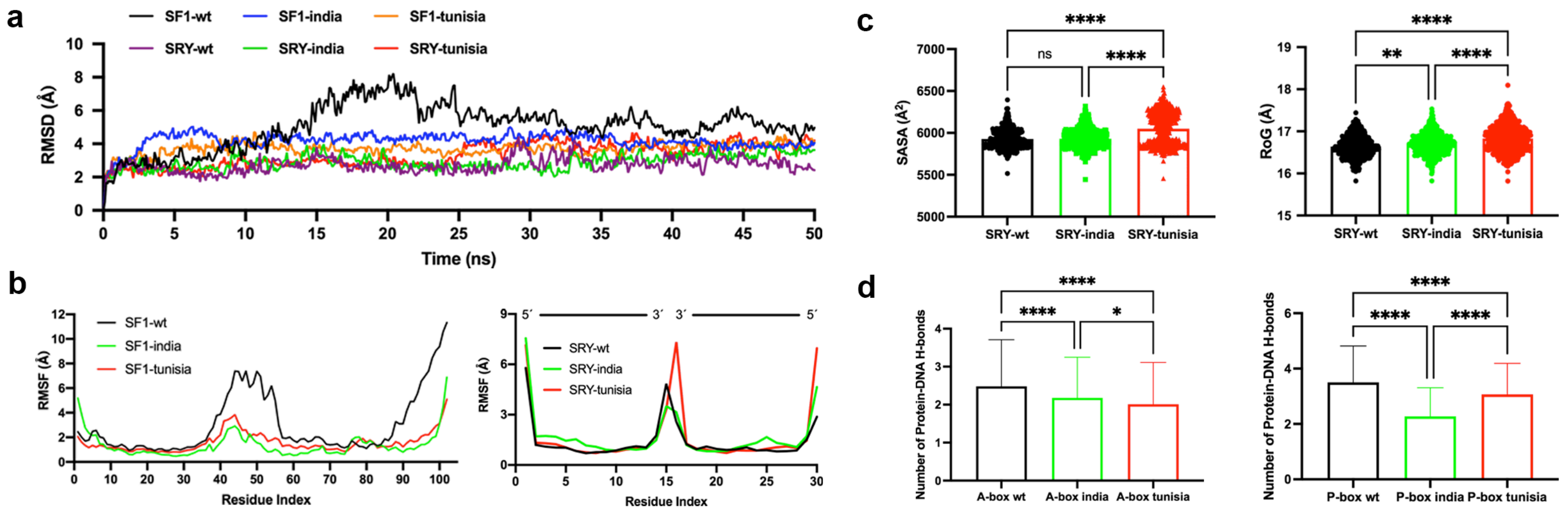

Supplementary Figure 04

Molecular dynamics of NR5A1 and response element interactions.

a. Root-mean square deviation (RMSD) representing the global movement for NR5A1 and target DNA. Reduced movement of NR5A1 with the variant response elements was seen compared to the wild-type response element.

b. Root-mean square fluctuation (RMSF) representing local movements for individual residues on protein and nucleic acid during simulations suggested reduced flexibility with the variant response elements. RMSD and RMSF were defined for Cα and C5`for protein and nucleic acid residues respectively.

c. Solvent accessible surface-area (SASA) and radius of gyration (RoG) to assess the compactness for the wild-type NR5A1 response element and variants during simulation.

d. Number of H-bond interactions between the NR5A1 A-box and P-box with the NR5A1 response elements during simulation showed reduced interaction for the variants.

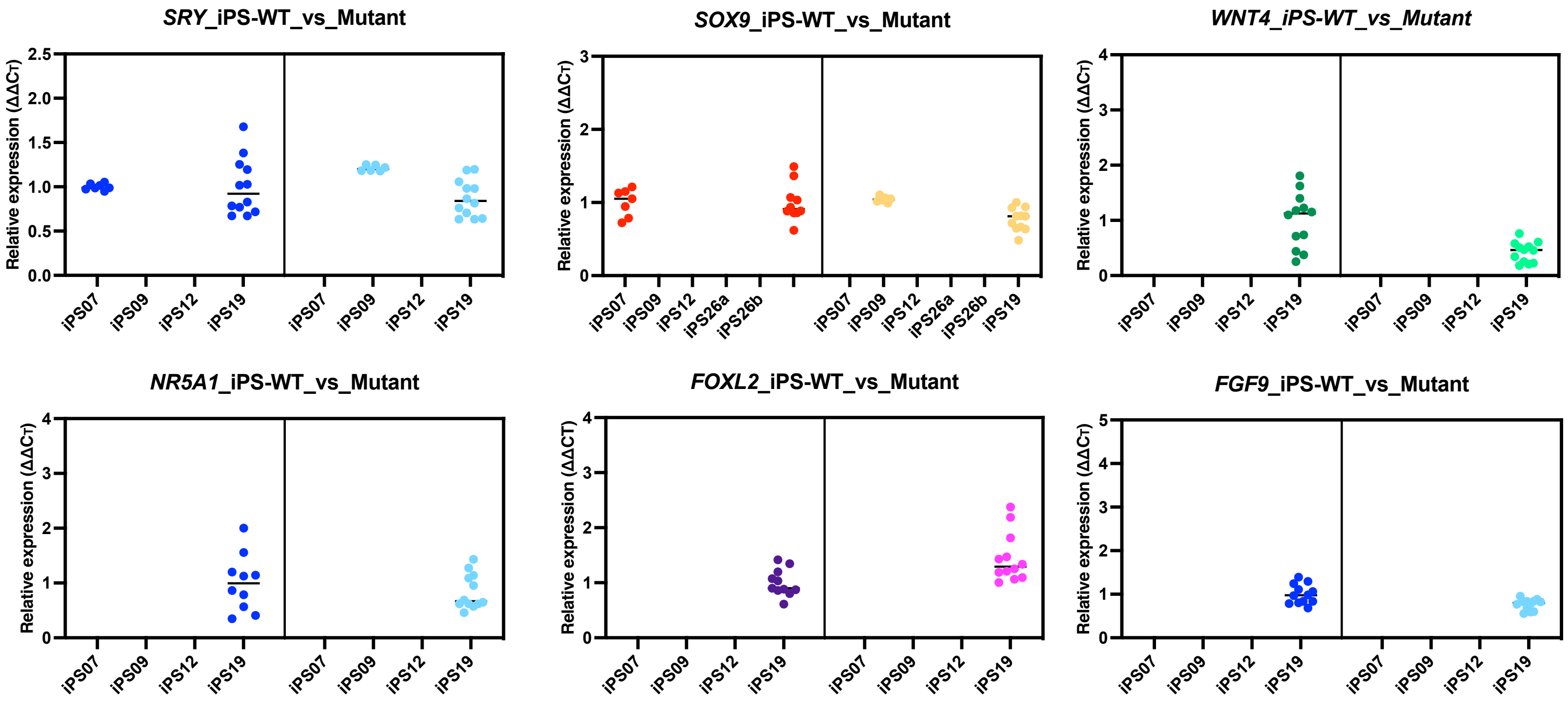

Supplementary_Figure_05

Supplementary to Figure 05

This PDF file is an export of data from Supplementary_Data_29.

rt-qPCR experiments showing the expression kinetics for the *SRY*, *SOX9*, *NR5A1*, *WNT4*, *FOXL2* and *FGF9* genes comparing wild type and mutant samples, with detailed analysis provided for each experiment in undifferentiated hiPSC.

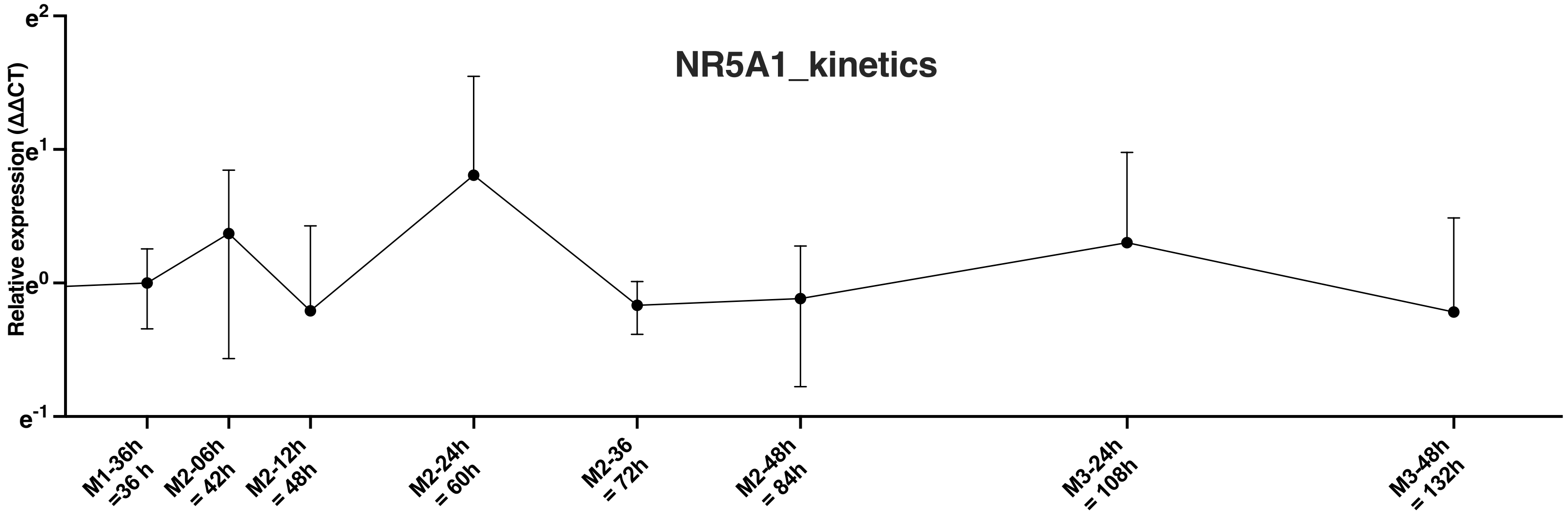

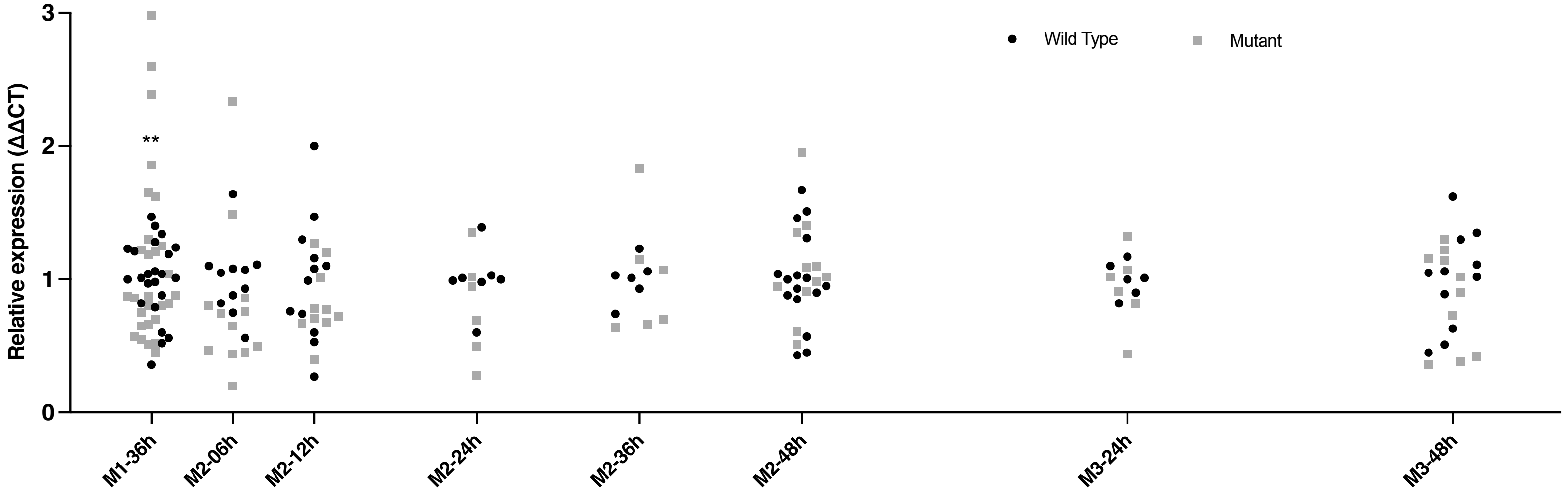

Supplementary_Figure_06

Supplementary to Figure 05

This PDF file is an export of data from Supplementary_Data_29.

Comparison between Wild Type and Mutant hiPSC lines. The expression levels of *NR5A1* and *FGF9* are detailed for each experiment and timepoint.

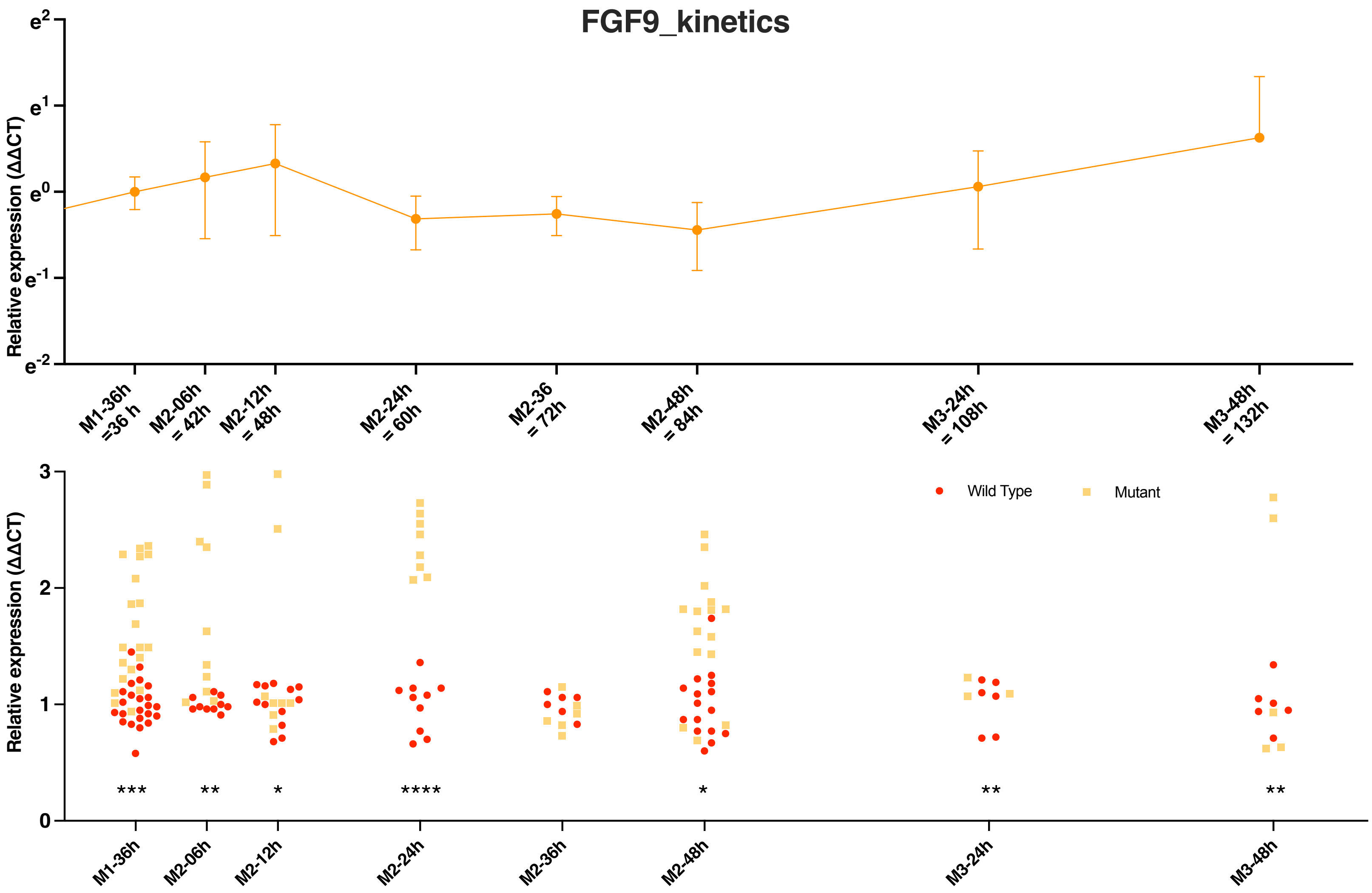

Supplementary_Figure_07

Supplementary to Figure 05

This PDF file is an export of data from Supplementary_Data_29.

Expression kinetics and comparison between Wild Type and Mutant for the *FOXL2* gene in rt-qPCR experiments.

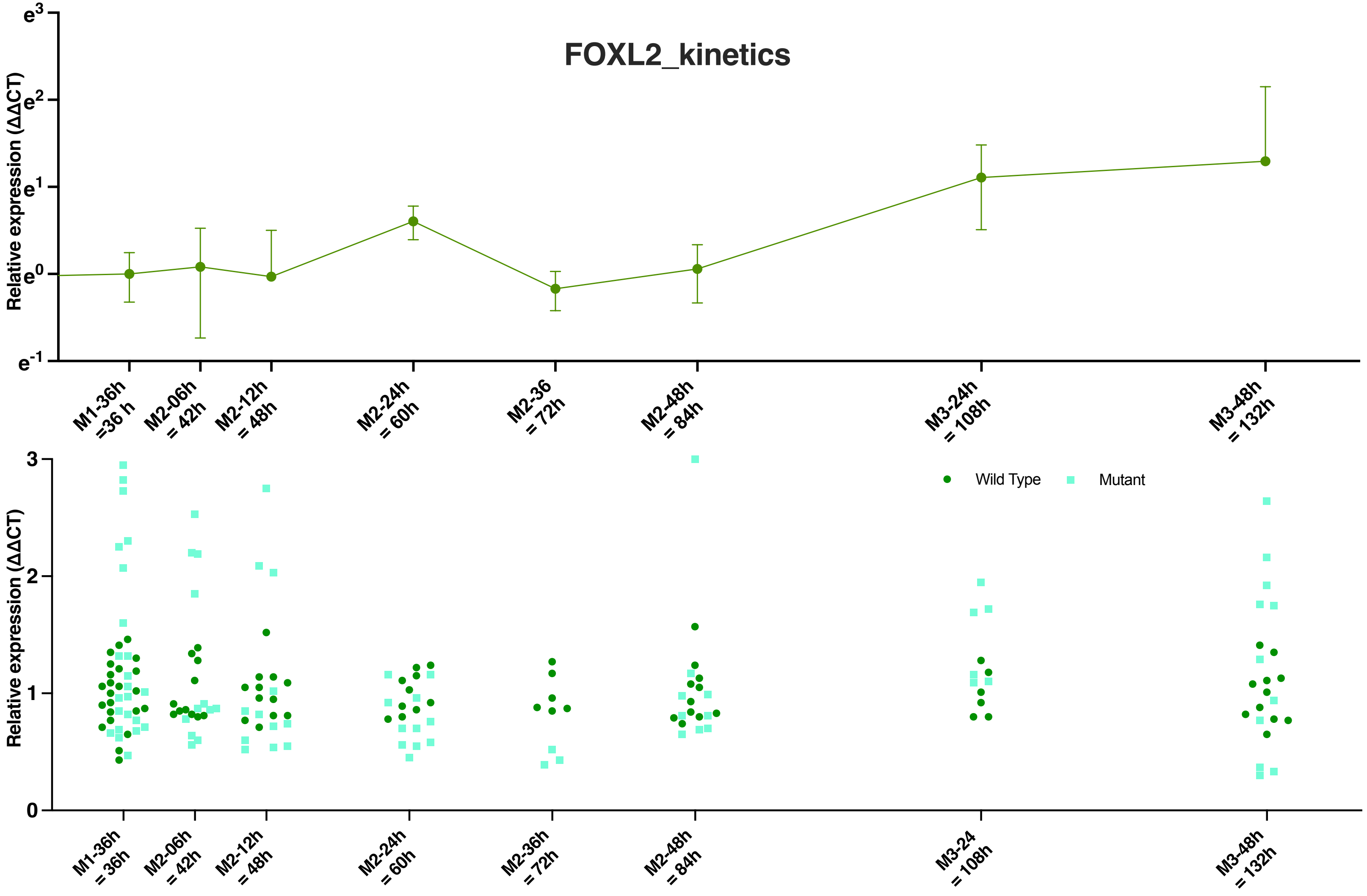

Supplementary_Figure_08

Supplementary to Figure 05

This PDF file is an export of data from Supplementary_Data_29.

Expression kinetics and comparison between Wild Type and Mutant for the *NR5A1* gene in rt-qPCR experiments.

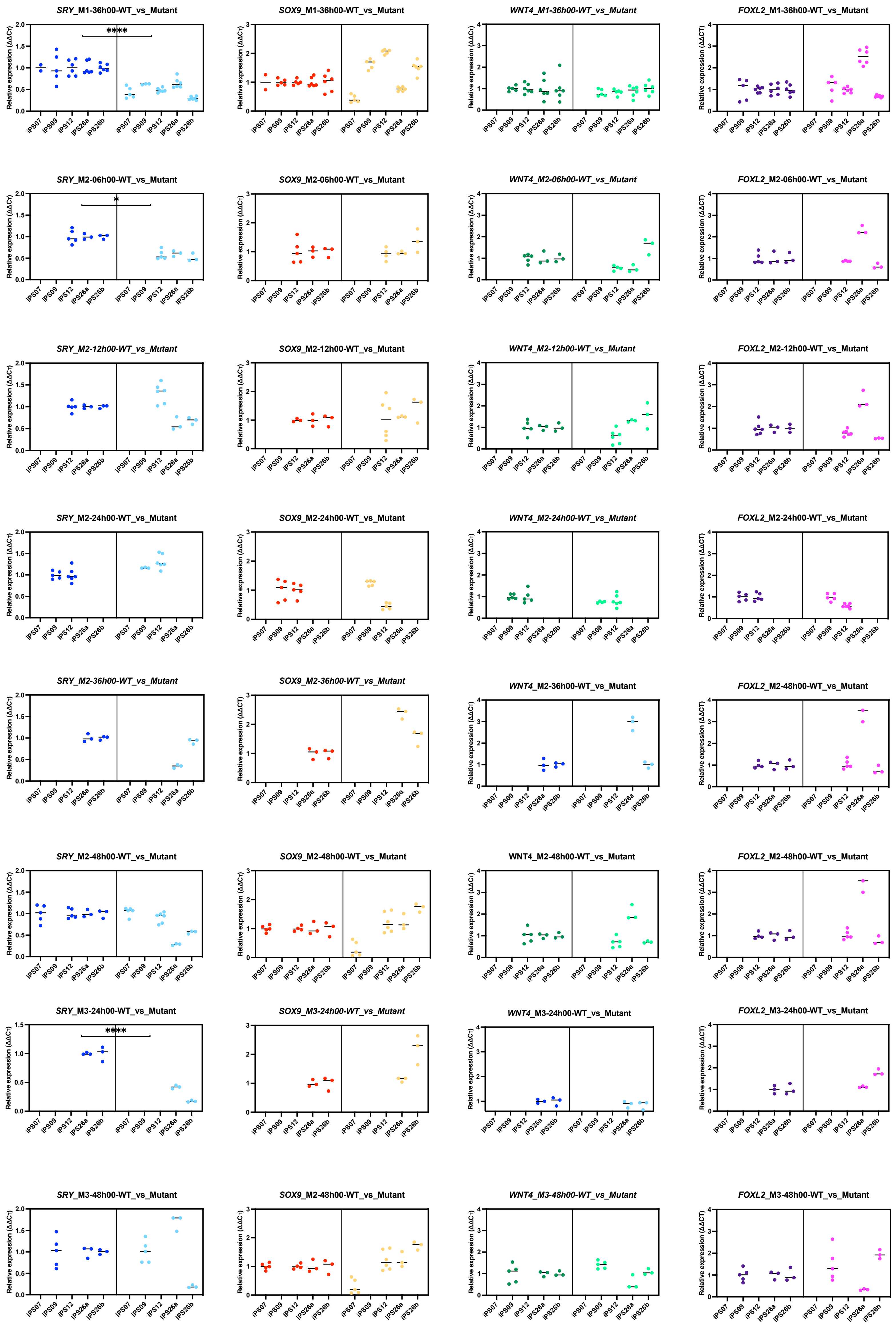

Supplementary_Figure_09

Supplementary to Figure 05

This PDF file is an export of data from Supplementary_Data_29.

A comprehensive file detailing the results of real-time quantitative PCR (rt-qPCR) experiments, comparing wild type and mutant samples, with detailed analysis provided for each experiment, gene (*SRY*, *SOX9*, *FGF9*, and *WNT4*), and timepoint.

Supplementary_Figure_10

Supplementary to Figure 05

Comparison between Wild Type and Mutant hiPSC lines. The expression levels of *NR5A1* and *FGF9* are detailed for each experiment and timepoint.

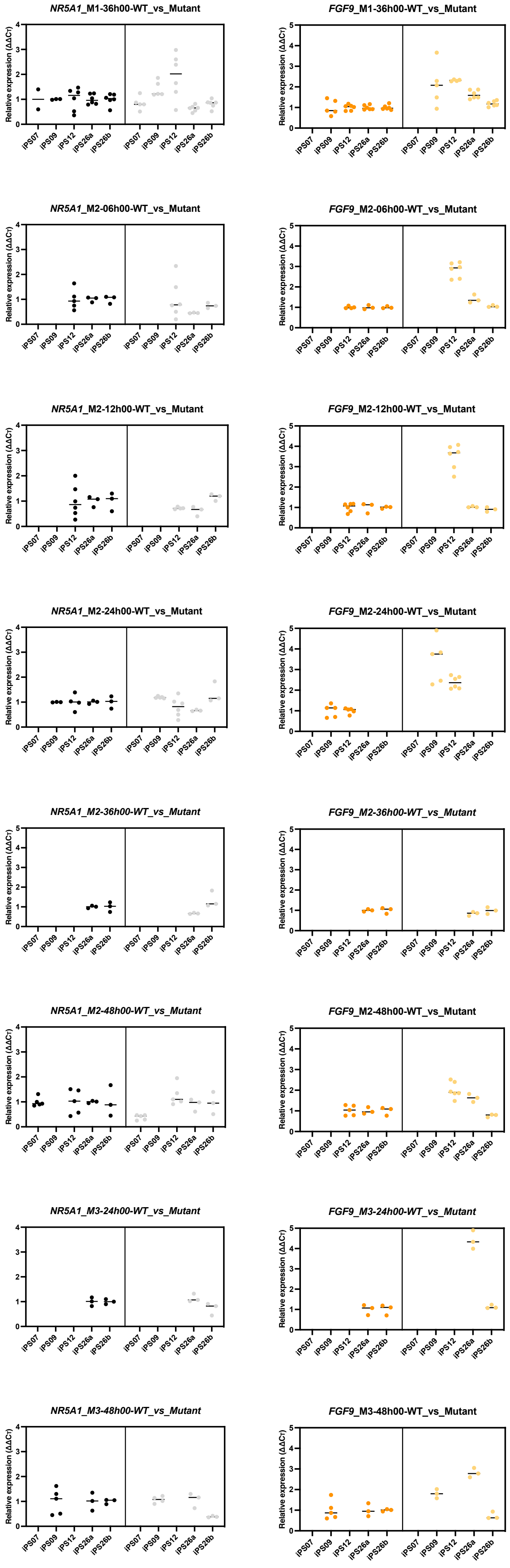

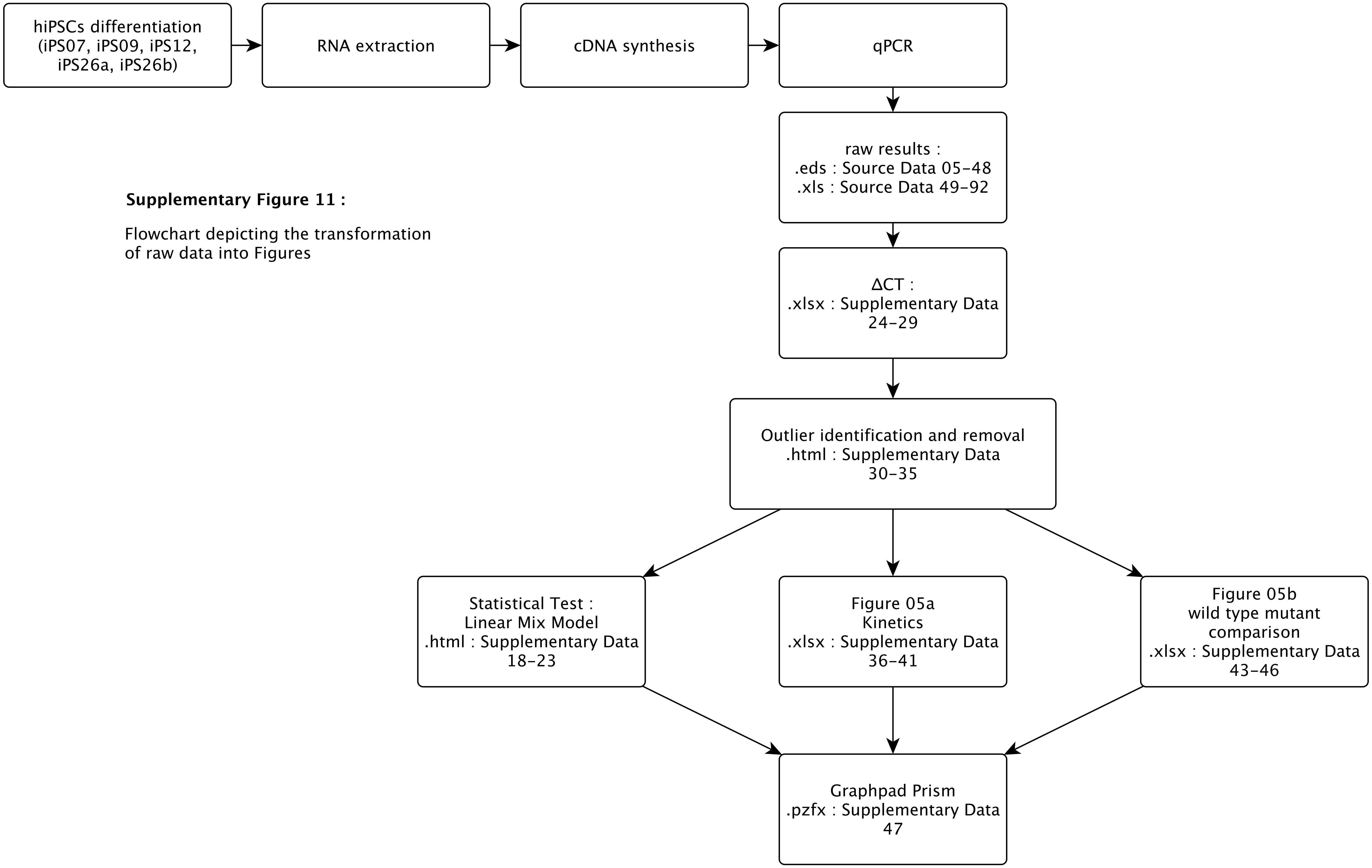

Supplementary Figure 11 :

Flowchart depicting the transformation of raw data into Figures

Case	Sex of rearing	Karyotype	Phenotype, External genitalia	Internal genitalia	Gonad histology	Diagnosis
**Pedigree A**						
V.12 (index)	M	46,XY	Gynaecomastia, micropenis, bilateral cryptorchidism	Rudimentary uterus	R, ovarian-like stromal tissue with hyalinization and calcifications; L, seminoma/dysgerminoma	46,XY gonadal dysgenesis
V.16	M	46,XY	Ambiguous	Uterus, bilateral fallopian tubes	R, testis, hyalinated tubules, semnoma ; L ovarian-like stroma	46,XY gonadal dysgenesis
VI.3	M	46,XY	Ambiguous, empty labio-scrotal folds	Absent uterus, fallopian tube	R, testis with ovarian-like stroma; L, streak gonad	46,XY gonadal dysgenesis
VI.9	M	46,XY	Ambiguous, unilateral cryptorchidism, perineal hypospadias, separate vaginal orifice	Absent uterus, left fallopian tube	R, testis; L, ovarian-like stroma	46,XY gonadal dysgenesis
V.6	F	46,XY	Female, absence of menarche	-	-	Suspected 46,XY gonadal dysgenesis
V.9	F	46,XY	Female, absence of menarche	-	-	Suspected 46,XY gonadal dysgenesis
V.11	F	46,XY	Female, absence of menarche	-	-	Suspected 46,XY gonadal dysgenesis
**Pedigree B**						
V.1	M	46,XY	Ambiguous	-	R, testis with ovarian-like stroma; L, streak gonad	46,XY gonadal dysgenesis
IV.1	F	46,XY	Female absence of menarche	-	-	Suspected 46,XY gonadal dysgenesis
IV.2	F	46,XY	Female absence of menarche	-	-	Suspected 46,XY gonadal dysgenesis
VI.1	F	46,XY	Female	-	Bilateral streak gonads with gonadoblastoma	46,XY gonadal dysgenesis

Supplementary_Table_01

Detailed clinical information for family members where available. The age at clinical presentation ranged from 6 to 23 years of age

Supplementary Notes

I. FILE FORMAT **3**

1. .CLC FILES 3
2. .CSV AND .XML FILES 3
3. .HTML FILES 3
4. .XLS AND .XLSX FILES 3
5. .PDF FILES 3
6. .EDS FILES 3
7. .PZFX FILES 4

II. SUPPLEMENTARY DATA **4**

1. FIGURE 01 4

Supplementary_Data_01.clc 4

Supplementary_Data_02.clc 4

2. FIGURE 02 4

Supplementary_Data_03.clc 4

Supplementary_Data_04.clc 5

Supplementary_Data_05.clc 5

Supplementary_Data_06.xlsx 5

Supplementary_Data_07.xlsx 5

3. FIGURE 03 5

Supplementary_Data_08.xlsx 5

4. FIGURE 04 5

Supplementary_Data_09.clc 5

Supplementary_Data_10.clc 6

Supplementary_Data_11.clc 6

Supplementary_Data_12.clc 6

Supplementary_Data_13.clc 6

Supplementary_Data_14.clc 6

Supplementary_Data_15.html and Supplementary_Data_16.html 6

5. FIGURE 05 6

Supplementary_Data_17.html 7

Supplementary_Data_18.html to Supplementary_Data_23.html 7

Supplementary_Data_24.xlsx to Supplementary_Data_29.xlsx 8

Supplementary_Data_30.html to Supplementary_Data_35.html 8

Supplementary_Data_36.xlsx to Supplementary_Data_41.xlsx 8

Supplementary_Data_42.xlsx to Supplementary_Data_46.xlsx 8

Supplementary_Data_47.pzfx 9

III. SOURCE DATA 10

1. FIGURE 04.. 10

Source_Data_01.csv and Source_Data_02.csv 10

Source_Data_03.xml and Source_Data_04.xml... 10

2. FIGURE 05.. 10

Source_Data_05.eds to Source_Data_91.xls 10

I. File format

1. .clc files

The sequences are provided as .clc files since they were annotated using the CLC Main Workbench software (CLC Main Workbench software).

The sequences and their annotations can be easily visualized using the demo version of CLC main workbench

This software does not require any specific bioinformatic skills and enables clear and precise annotation of the sequences.

2. .csv and .xml files

The Luciferase assay was performed using the Promega Glomax apparatus, and the raw results were obtained in the form of .csv and .xml files.

3. .html files

Statistical analysis was conducted in RStudio, and the scripts were rendered as HTML documents using R Markdown.

4. .xls and .xlsx files

.xls and .xlsx files are Microsoft Excel spreadsheets. These file formats are supported by most spreadsheet applications, including Excel, Apple Numbers, OpenOffice, LibreOffice Calc, and more.

5. .pdf files

The PDF format is now an open standard, maintained by the International Organization for Standardization (ISO). PDF files are widely used to create and share documents, as the PDF will keep formatting intact regardless of the viewer's operating system and software.

6. .eds files

The Quantitative PCR was performed using the a StepOne Plus Real-Time PCR apparatus, and the raw results were obtained in the form of .eds and .xls files.

7. .pzfx files

.pzfx is the default format of GraphPad Prism.

The files can be easily visualized using Prism's viewer mode.

II. Supplementary Data

1. Figure 01

Supplementary_Data_01.clc

The DNA genomic sequences were downloaded from NCBI.

The complete human genomic region and annotations were obtained from the

NC_000024 human contig.

Human annotated sequences from NC_000024 used to build Fig 1A. The figure depicts an 80,413 bp sequence fragment (GRC38h38.p13-chrY: 2786855-2867268) spanning from the *SRY* gene to the RPSY4 gene.

The repeated sequences in the Y chromosome locus between *SRY* and RPS4Y1 were identified with Repeat masker.

Supplementary_Data_02.clc

A 7386bp sequence fragment (GRC38h38.p13-chrY: 2786855-2794240) from *SRY* to the beginning of the repetitive elements.

The predicted binding sites for GATA4, NR5A1, and WT1 were identified with the MatInspector module from the Genomatix suite Precigen Bioinformatics Germany GmbH (product discontinued).

2. Figure 02

Supplementary_Data_03.clc

Annotated sequences for the *SRY* gene and its upstream sequences from 83 eutherian species were downloaded from NCBI. Their accession number, common name and latin name are given in Supplementary_Data_06.xlsx.

The sequences used to build Fig 2B were selected on two criteria: they were in close vicinity to *SRY* and they returned a hit in a blastn search against the human *SRY* with

default settings (Number of threads:8, mask low complexity regions, Expect: 10, Word size: 11, Match 2, Mismatch -3, gap costs: existence 5, extension 2). This association was considered to be a strong indicator of homology.

The annotated sequences are given as a CLC Main Workbench gene list.

Supplementary_Data_04.clc

DNA fragments homologous to *SRY* and its upstream sequence have been identified by BLAST in the genome of the 83 mammalian species. These homologous DNA fragments were aligned on the 7046bp human sequence shown in Fig 1B and 2A using the ClustalW algorithm from CLC main workbench and subsequently hand edited.

Supplementary_Data_05.clc

The 17 most relevant species were selected from Supplementary_Data_04, realigned using the ClustalW algorithm from CLC main workbench and subsequently hand edited.

Supplementary_Data_06.xlsx

This file contains a comprehensive list of the accession numbers, common names, and Latin names of the sequences corresponding to the *SRY* gene and its upstream sequences from 83 eutherian species, downloaded from NCBI.

Supplementary_Data_07.xlsx

A list of the transcription factor binding sites predicted by MatInspector (https://genomatix.de) within the E250 enhancer

3. Figure 03

Supplementary_Data_08.xlsx

Polymorphisms in the genomic sequence of the Y chromosome that allowed the identification of the Variant-1 haplogroup.

4. Figure 04

Supplementary_Data_09.clc

The pcDNA3 vector was employed for expressing human NR5A1 in human cells. The protein is N-terminally tagged with two MYC epitopes.

Supplementary_Data_10.clc

The pCMV-Renilla (#AF025843) vector was employed to express the Renilla luciferase gene. This gene served as a reference gene for normalizing gene expression in transiently transfected HEK293T and HeLa cells.

Supplementary_Data_11.clc

Luciferase expression was achieved using the pGL4.26 vector, with a minimal promoter controlling its transcription. To assess its enhancer activity, the reference variant of E250 was cloned upstream of the minimal promoter.

Supplementary_Data_12.clc

Luciferase expression was achieved using the pGL4.26 vector, with a minimal promoter controlling its transcription. To assess its enhancer activity, the Variant-1 of E250 was cloned upstream of the minimal promoter.

Supplementary_Data_13.clc

Luciferase expression was achieved using the pGL4.26 vector, with a minimal promoter controlling its transcription. To assess its enhancer activity, the Variant-2 of E250 was cloned upstream of the minimal promoter.

Supplementary_Data_14.clc

Luciferase expression was achieved using the pGL4.26 vector, with a minimal promoter controlling its transcription. To assess its enhancer activity, a modified version of E250 was generated by introducing substitutions in the NR5A1-binding site, rendering it unrecognizable. This modified E250 variant was then cloned upstream of the minimal promoter.

Supplementary_Data_15.html and Supplementary_Data_16.html

Statistical analysis for the Luciferase assay performed on HEK293T (Supplementary_Data_15) and HeLa (Supplementary_Data_16) cells.

5. Figure 05

Gene expression was determined using real time quantitative Polymerase Chain Reaction (qPCR).

The ΔCT analysis of qPCRs were performed as described in this video.

The results regarding the expression of several differentiation associated markers *SRY*, *SOX9*, *WNT4*, *NR5A1*, *FOXL2*, *FGF9*, *AMH* during the differentiation of hiPSCs into gonadal-like cells are as follows:

• Five differentiation experiments were conducted, namely iPS07, iPS09, iPS12, iPS26a, and iPS26b.

• Undifferentiated hiPSCs were obtained from two experiments: iPS07, and iPS19.

• Comparisons were performed between wild-type and mutant cell lines. RPL19 was utilized as the normalizer.

• The outliers were identified using the InterQuartile Range (IQR) method on the ΔCT values (dCT column). The outlier values were subsequently replaced with "NA" in the dCT_NA column. For more detailed information on the outlier detection process, refer to Supplementary_Data_17.html to Supplementary_Data_22.html.

• A linear mixed model (LMM) including the genotype, the time and their interaction as fixed effects and the experiment as random effect was fitted to investigate significant differences in the ΔCT values between wild type and mutant cells, allowing for a comprehensive examination of gene expression dynamics over time (Supplementary_Data_23.html to Supplementary_Data_28.html).

• Due to the intricate nature of the system, a substantial experimental effect was evidenced by the statistical test employed. Consequently, the results deemed statistically significant exhibit a high level of robustness.

Supplementary_Data_17.html
An Excel spreadsheet has been created to provide a detailed computation of ΔΔCT for comparing the expression levels of AMH between Wild Type and Mutant cell lines.

Supplementary_Data_18.html to Supplementary_Data_23.html
The Rmarkdown script describing the analysis of the real time qPCR utilizing a linear mixed model.

Supplementary_Data_24 (*SRY*), Supplementary_Data_25 (*SOX9*), Supplementary_Data_26 (*WNT4*), Supplementary_Data_27 (*NR5A1*), Supplementary_Data_28 (*FOXL2*), Supplementary_Data_29 (*FGF9*)

Supplementary_Data_24.xlsx to Supplementary_Data_29.xlsx

Excel spreadsheets have been created to provide a detailed computation of ΔCT used in Supplementary_Data_18.html to Supplementary_Data_23.html.

Supplementary_Data_42 (*SRY*), Supplementary_Data_43 (*SOX9*), Supplementary_Data_44 (*WNT4*), Supplementary_Data_45 (*NR5A1*), Supplementary_Data_46 (*FOXL2*), Supplementary_Data_47 (*FGF9*)

Supplementary_Data_30.html to Supplementary_Data_35.html

The outliers for the *SRY*, *SOX9*, *WNT4*, *NR5A1*, *FOXL2*, *FGF9*, *AMH* qPCRs were identified using the InterQuartile Range (IQR) method and subsequently excluded (R version 4.3.0 and RStudio version 2023.06.0+421).

Supplementary_Data_30 (*SRY*), Supplementary_Data_31 (*SOX9*), Supplementary_Data_32 (*WNT4*), Supplementary_Data_33 (*NR5A1*), Supplementary_Data_34 (*FOXL2*), Supplementary_Data_35 (*FGF9*)

Supplementary_Data_36.xlsx to Supplementary_Data_41.xlsx

Excel spreadsheets have been created to provide a detailed computation of ΔΔCT for comparing the expression levels of the following genes between Wild Type and Mutant cell lines : *SRY*, *SOX9*, *WNT4*, *NR5A1*, *FOXL2*, *FGF9*

The Wild Type condition was used as the calibrator.

Supplementary_Data_36 (*SRY*), Supplementary_Data_37 (*SOX9*), Supplementary_Data_38 (*WNT4*), Supplementary_Data_39 (*NR5A1*), Supplementary_Data_40 (*FOXL2*), Supplementary_Data_41 (*FGF9*), Supplementary_Data_42 (*AMH*)

Supplementary_Data_42.xlsx to Supplementary_Data_46.xlsx

Excel spreadsheets have been created to provide a detailed computation of ΔΔCT, establishing the expression kinetics of the following genes in Wild Type cell lines : *SRY*, *SOX9*, *WNT4*, *NR5A1*, *FOXL2*, *FGF9*, and *AMH*.

The procedure used was the same as described in Supplementary_Data_36.xlsx to Supplementary_Data_41.xlsx with the exception that M1-36h00 was used as the calibrator.

Supplementary_Data_42 (iPS07), Supplementary_Data_43 (iPS09), Supplementary_Data_44 (iPS12), Supplementary_Data_45 (iPS26a), Supplementary_Data_46 (iPS26b)

Supplementary_Data_47.pzfx

Figure 05 was created using GraphPad Prism version 10.1.1 (270). This file compiles data from Supplementary_Data_30.xlsx to Supplementary_Data_36.xlsx.

III. Source Data

1. Figure 04

Source_Data_01.csv and Source_Data_02.csv

Raw results of the Luciferase assay performed on HEK293T (Source_Data_01.csv) and HeLa cells (Source_Data_02.csv).

Source_Data_03.xml and Source_Data_04.xml

Raw results of the Luciferase assay performed on HEK293T (Source_Data_01.xml) and HeLa cells (Source_Data_02.xml).

2. Figure 05

Source_Data_05.eds to Source_Data_91.xls

Raw results of RT-qPCRs performed on RNA extracted from differentiated hiPSC (human induced Pluripotent Stem Cells). Both the .eds (Source_Data_05.eds to Source_Data_48.eds) and .xls (Source_Data_49.xls to Source_Data_91.xls) files are provided.

Figure	File format	Type	Name	Description
Fig04	csv	Source Data	Source_Data_01.csv	Luciferase experiment - Glomax raw data - HEK2937 cells
Fig04	csv	Source Data	Source_Data_02.csv	Luciferase experiment - Glomax raw data - HeLa cells
Fig04	xml	Source Data	Source_Data_03.xml	Luciferase experiment - Glomax raw data - HEK2937 cells
Fig04	xml	Source Data	Source_Data_04.xml	Luciferase experiment - Glomax raw data - HeLa cells
Fig05	eds	Source Data	Source_Data_05.eds	qPCR experiment - StepOne Plus Real-Time PCR raw data - qPCR35
Fig05	eds	Source Data	Source_Data_06.eds	qPCR experiment - StepOne Plus Real-Time PCR raw data - qPCR36
Fig05	eds	Source Data	Source_Data_07.eds	qPCR experiment - StepOne Plus Real-Time PCR raw data - qPCR38
Fig05	eds	Source Data	Source_Data_08.eds	qPCR experiment - StepOne Plus Real-Time PCR raw data - qPCR39
Fig05	eds	Source Data	Source_Data_09.eds	qPCR experiment - StepOne Plus Real-Time PCR raw data - qPCR40
Fig05	eds	Source Data	Source_Data_10.eds	qPCR experiment - StepOne Plus Real-Time PCR raw data - qPCR65
Fig05	eds	Source Data	Source_Data_11.eds	qPCR experiment - StepOne Plus Real-Time PCR raw data - qPCR66
Fig05	eds	Source Data	Source_Data_12.eds	qPCR experiment - StepOne Plus Real-Time PCR raw data - qPCR67
Fig05	eds	Source Data	Source_Data_13.eds	qPCR experiment - StepOne Plus Real-Time PCR raw data - qPCR68
Fig05	eds	Source Data	Source_Data_14.eds	qPCR experiment - StepOne Plus Real-Time PCR raw data - qPCR69
Fig05	eds	Source Data	Source_Data_15.eds	qPCR experiment - StepOne Plus Real-Time PCR raw data - qPCR70
Fig05	eds	Source Data	Source_Data_16.eds	qPCR experiment - StepOne Plus Real-Time PCR raw data - qPCR71
Fig05	eds	Source Data	Source_Data_17.eds	qPCR experiment - StepOne Plus Real-Time PCR raw data - qPCR72
Fig05	eds	Source Data	Source_Data_18.eds	qPCR experiment - StepOne Plus Real-Time PCR raw data - qPCR73
Fig05	eds	Source Data	Source_Data_19.eds	qPCR experiment - StepOne Plus Real-Time PCR raw data - qPCR74
Fig05	eds	Source Data	Source_Data_20.eds	qPCR experiment - StepOne Plus Real-Time PCR raw data - qPCR79
Fig05	eds	Source Data	Source_Data_21.eds	qPCR experiment - StepOne Plus Real-Time PCR raw data - qPCR80
Fig05	eds	Source Data	Source_Data_22.eds	qPCR experiment - StepOne Plus Real-Time PCR raw data - qPCR81
Fig05	eds	Source Data	Source_Data_23.eds	qPCR experiment - StepOne Plus Real-Time PCR raw data - qPCR85
Fig05	eds	Source Data	Source_Data_24.eds	qPCR experiment - StepOne Plus Real-Time PCR raw data - qPCR86
Fig05	eds	Source Data	Source_Data_25.eds	qPCR experiment - StepOne Plus Real-Time PCR raw data - qPCR106
Fig05	eds	Source Data	Source_Data_26.eds	qPCR experiment - StepOne Plus Real-Time PCR raw data - qPCR107
Fig05	eds	Source Data	Source_Data_27.eds	qPCR experiment - StepOne Plus Real-Time PCR raw data - qPCR115
Fig05	eds	Source Data	Source_Data_28.eds	qPCR experiment - StepOne Plus Real-Time PCR raw data - qPCR116
Fig05	eds	Source Data	Source_Data_29.eds	qPCR experiment - StepOne Plus Real-Time PCR raw data - qPCR132
Fig05	eds	Source Data	Source_Data_30.eds	qPCR experiment - StepOne Plus Real-Time PCR raw data - qPCR133
Fig05	eds	Source Data	Source_Data_31.eds	qPCR experiment - StepOne Plus Real-Time PCR raw data - qPCR134
Fig05	eds	Source Data	Source_Data_32.eds	qPCR experiment - StepOne Plus Real-Time PCR raw data - qPCR135
Fig05	eds	Source Data	Source_Data_33.eds	qPCR experiment - StepOne Plus Real-Time PCR raw data - qPCR136
Fig05	eds	Source Data	Source_Data_34.eds	qPCR experiment - StepOne Plus Real-Time PCR raw data - qPCR137
Fig05	eds	Source Data	Source_Data_35.eds	qPCR experiment - StepOne Plus Real-Time PCR raw data - qPCR138
Fig05	eds	Source Data	Source_Data_36.eds	qPCR experiment - StepOne Plus Real-Time PCR raw data - qPCR139

Fig05	eds	Source Data	Source_Data_37.eds	qPCR experiment - StepOne Plus Real-Time PCR raw data - qPCR142
Fig05	eds	Source Data	Source_Data_38.eds	qPCR experiment - StepOne Plus Real-Time PCR raw data - qPCR140
Fig05	eds	Source Data	Source_Data_39.eds	qPCR experiment - StepOne Plus Real-Time PCR raw data - qPCR141
Fig05	eds	Source Data	Source_Data_40.eds	qPCR experiment - StepOne Plus Real-Time PCR raw data - qPCR143
Fig05	eds	Source Data	Source_Data_41.eds	qPCR experiment - StepOne Plus Real-Time PCR raw data - qPCR144
Fig05	eds	Source Data	Source_Data_42.eds	qPCR experiment - StepOne Plus Real-Time PCR raw data - qPCR151
Fig05	eds	Source Data	Source_Data_43.eds	qPCR experiment - StepOne Plus Real-Time PCR raw data - qPCR149
Fig05	eds	Source Data	Source_Data_44.eds	qPCR experiment - StepOne Plus Real-Time PCR raw data - qPCR145
Fig05	eds	Source Data	Source_Data_45.eds	qPCR experiment - StepOne Plus Real-Time PCR raw data - qPCR146
Fig05	eds	Source Data	Source_Data_46.eds	qPCR experiment - StepOne Plus Real-Time PCR raw data - qPCR147
Fig05	eds	Source Data	Source_Data_47.eds	qPCR experiment - StepOne Plus Real-Time PCR raw data - qPCR148
Fig05	eds	Source Data	Source_Data_48.eds	qPCR experiment - StepOne Plus Real-Time PCR raw data - qPCR150
Fig05	xls	Source Data	Source_Data_49.xls	qPCR experiment - StepOne Plus Real-Time PCR raw data - qPCR35
Fig05	xls	Source Data	Source_Data_50.xls	qPCR experiment - StepOne Plus Real-Time PCR raw data - qPCR36
Fig05	xls	Source Data	Source_Data_51.xls	qPCR experiment - StepOne Plus Real-Time PCR raw data - qPCR38
Fig05	xls	Source Data	Source_Data_52.xls	qPCR experiment - StepOne Plus Real-Time PCR raw data - qPCR39
Fig05	xls	Source Data	Source_Data_53.xls	qPCR experiment - StepOne Plus Real-Time PCR raw data - qPCR40
Fig05	xls	Source Data	Source_Data_54.xls	qPCR experiment - StepOne Plus Real-Time PCR raw data - qPCR65
Fig05	xls	Source Data	Source_Data_55.xls	qPCR experiment - StepOne Plus Real-Time PCR raw data - qPCR66
Fig05	xls	Source Data	Source_Data_56.xls	qPCR experiment - StepOne Plus Real-Time PCR raw data - qPCR67
Fig05	xls	Source Data	Source_Data_57.xls	qPCR experiment - StepOne Plus Real-Time PCR raw data - qPCR68
Fig05	xls	Source Data	Source_Data_58.xls	qPCR experiment - StepOne Plus Real-Time PCR raw data - qPCR69
Fig05	xls	Source Data	Source_Data_59.xls	qPCR experiment - StepOne Plus Real-Time PCR raw data - qPCR70
Fig05	xls	Source Data	Source_Data_60.xls	qPCR experiment - StepOne Plus Real-Time PCR raw data - qPCR71
Fig05	xls	Source Data	Source_Data_61.xls	qPCR experiment - StepOne Plus Real-Time PCR raw data - qPCR72
Fig05	xls	Source Data	Source_Data_62.xls	qPCR experiment - StepOne Plus Real-Time PCR raw data - qPCR73
Fig05	xls	Source Data	Source_Data_63.xls	qPCR experiment - StepOne Plus Real-Time PCR raw data - qPCR74
Fig05	xls	Source Data	Source_Data_64.xls	qPCR experiment - StepOne Plus Real-Time PCR raw data - qPCR79
Fig05	xls	Source Data	Source_Data_65.xls	qPCR experiment - StepOne Plus Real-Time PCR raw data - qPCR80
Fig05	xls	Source Data	Source_Data_66.xls	qPCR experiment - StepOne Plus Real-Time PCR raw data - qPCR81
Fig05	xls	Source Data	Source_Data_67.xls	qPCR experiment - StepOne Plus Real-Time PCR raw data - qPCR85
Fig05	xls	Source Data	Source_Data_68.xls	qPCR experiment - StepOne Plus Real-Time PCR raw data - qPCR86
Fig05	xls	Source Data	Source_Data_69.xls	qPCR experiment - StepOne Plus Real-Time PCR raw data - qPCR106
Fig05	xls	Source Data	Source_Data_70.xls	qPCR experiment - StepOne Plus Real-Time PCR raw data - qPCR107
Fig05	xls	Source Data	Source_Data_71.xls	qPCR experiment - StepOne Plus Real-Time PCR raw data - qPCR115
Fig05	xls	Source Data	Source_Data_72.xls	qPCR experiment - StepOne Plus Real-Time PCR raw data - qPCR116
Fig05	xls	Source Data	Source_Data_73.xls	qPCR experiment - StepOne Plus Real-Time PCR raw data - qPCR132
Fig05	xls	Source Data	Source_Data_74.xls	qPCR experiment - StepOne Plus Real-Time PCR raw data - qPCR133

Fig05	xls	Source Data	Source_Data_75.xls	qPCR experiment - StepOne Plus Real-Time PCR raw data - qPCR134
Fig05	xls	Source Data	Source_Data_76.xls	qPCR experiment - StepOne Plus Real-Time PCR raw data - qPCR135
Fig05	xls	Source Data	Source_Data_77.xls	qPCR experiment - StepOne Plus Real-Time PCR raw data - qPCR136
Fig05	xls	Source Data	Source_Data_78.xls	qPCR experiment - StepOne Plus Real-Time PCR raw data - qPCR137
Fig05	xls	Source Data	Source_Data_79.xls	qPCR experiment - StepOne Plus Real-Time PCR raw data - qPCR138
Fig05	xls	Source Data	Source_Data_80.xls	qPCR experiment - StepOne Plus Real-Time PCR raw data - qPCR139
Fig05	xls	Source Data	Source_Data_81.xls	qPCR experiment - StepOne Plus Real-Time PCR raw data - qPCR142
Fig05	xls	Source Data	Source_Data_82.xls	qPCR experiment - StepOne Plus Real-Time PCR raw data - qPCR140
Fig05	xls	Source Data	Source_Data_83.xls	qPCR experiment - StepOne Plus Real-Time PCR raw data - qPCR141
Fig05	xls	Source Data	Source_Data_84.xls	qPCR experiment - StepOne Plus Real-Time PCR raw data - qPCR143
Fig05	xls	Source Data	Source_Data_85.xls	qPCR experiment - StepOne Plus Real-Time PCR raw data - qPCR144
Fig05	xls	Source Data	Source_Data_86.xls	qPCR experiment - StepOne Plus Real-Time PCR raw data - qPCR151
Fig05	xls	Source Data	Source_Data_87.xls	qPCR experiment - StepOne Plus Real-Time PCR raw data - qPCR149
Fig05	xls	Source Data	Source_Data_88.xls	qPCR experiment - StepOne Plus Real-Time PCR raw data - qPCR145
Fig05	xls	Source Data	Source_Data_89.xls	qPCR experiment - StepOne Plus Real-Time PCR raw data - qPCR146
Fig05	xls	Source Data	Source_Data_90.xls	qPCR experiment - StepOne Plus Real-Time PCR raw data - qPCR147
Fig05	xls	Source Data	Source_Data_91.xls	qPCR experiment - StepOne Plus Real-Time PCR raw data - qPCR148
Fig05	xls	Source Data	Source_Data_92.xls	qPCR experiment - StepOne Plus Real-Time PCR raw data - qPCR150
Fig01A	clc	Supplementary Data	Supplementary_Data_01.clc	annotated SRY locus - CLC main workbench file - *SRY* - Complete locus
Fig01B	clc	Supplementary Data	Supplementary_Data_02.clc	annotated SRY locus - CLC main workbench file - *SRY* - 7386bp sequence fragment
Fig02B	clc	Supplementary Data	Supplementary_Data_03.clc	annotated SRY locus - CLC main workbench file - *SRY* - 83 species sequence list
Fig02B	clc	Supplementary Data	Supplementary_Data_04.clc	annotated SRY locus - CLC main workbench file - SRY - 83 species sequence alignment
Fig02B	clc	Supplementary Data	Supplementary_Data_05.clc	annotated SRY locus - CLC main workbench file - SRY - 17 species sequence alignment
Fig02B	xlsx	Supplementary Data	Supplementary_Data_06.xlsx	List of the mammalian species used in this study
Fig02B	xlsx	Supplementary Data	Supplementary_Data_07.xlsx	Genomatix predicted transcription binding sites in the E250 enhancer
Fig03	xlsx	Supplementary Data	Supplementary_Data_08.xlsx	H1a1 haplogroup
Fig04E	clc	Supplementary Data	Supplementary_Data_09.clc	Luciferase: annotated Plasmid-pcDNA3-Myc2X-NR5A1 - CLC main workbench file
Fig04E	clc	Supplementary Data	Supplementary_Data_10.clc	Luciferase: annotated Plasmid-pCMV-Renilla-AF025843 - CLC main workbench file
Fig04E	clc	Supplementary Data	Supplementary_Data_11.clc	Luciferase: annotated Plasmid-pGL4.26_link5_E250-Reference - CLC main workbench file
Fig04E	clc	Supplementary Data	Supplementary_Data_12.clc	Luciferase: annotated Plasmid-pGL4.26_link5_pGL4.26_link5_E250-Variant-1 CLC main workbench file
Fig04E	clc	Supplementary Data	Supplementary_Data_13.clc	Luciferase: annotated Plasmid-pGL4.26_link5_pGL4.26_link5_E250-Variant-2 CLC main workbench file
Fig04E	clc	Supplementary Data	Supplementary_Data_14.clc	Luciferase: annotated Plasmid-pGL4.26_link5_E250-ΔNR5A1 - CLC main workbench file
Fig04	html	Supplementary Data	Supplementary_Data_15.html	Luciferase on HEK293T cells - statistical analysis in R
Fig04	html	Supplementary Data	Supplementary_Data_16.html	Luciferase on HeLa cells - statistical analysis in R
Fig05B	xlsx	Supplementary Data	Supplementary_Data_17.xlsx	RT-qPCR analysis - AMH - Wild type versus mutant comparison
Fig05B	html	Supplementary Data	Supplementary_Data_18.html	RT-qPCR analysis - SRY - Linear Mixed Model in R

Fig05B	html	Supplementary Data	Supplementary_Data_19.html	RT-qPCR analysis - SOX9 - Linear Mixed Model in R
Fig05B	html	Supplementary Data	Supplementary_Data_20.html	RT-qPCR analysis - WNT4 - Linear Mixed Model in R
Fig05B	html	Supplementary Data	Supplementary_Data_21.html	RT-qPCR analysis - NR5A1 - Linear Mixed Model in R
Fig05B	html	Supplementary Data	Supplementary_Data_22.html	RT-qPCR analysis - FOXL2 - Linear Mixed Model in R
Fig05B	html	Supplementary Data	Supplementary_Data_23.html	RT-qPCR analysis - FGF9 - Linear Mixed Model in R
Fig05B	xlsx	Supplementary Data	Supplementary_Data_24.xlsx	RT-qPCR analysis - SRY - data preparation for Linear Mixed Model in R
Fig05B	xlsx	Supplementary Data	Supplementary_Data_25.xlsx	RT-qPCR analysis - SOX9 - data preparation for Linear Mixed Model in R
Fig05B	xlsx	Supplementary Data	Supplementary_Data_26.xlsx	RT-qPCR analysis - WNT4 - data preparation for Linear Mixed Model in R
Fig05B	xlsx	Supplementary Data	Supplementary_Data_27.xlsx	RT-qPCR analysis - NR5A1 - data preparation for Linear Mixed Model in R
Fig05B	xlsx	Supplementary Data	Supplementary_Data_28.xlsx	RT-qPCR analysis - FOXL2 - data preparation for Linear Mixed Model in R
Fig05B	xlsx	Supplementary Data	Supplementary_Data_29.xlsx	RT-qPCR analysis - FGF9 - data preparation for Linear Mixed Model in R
Fig05	html	Supplementary Data	Supplementary_Data_30.html	RT-qPCR analysis - SRY - outlier identification by the IQR method in R
Fig05	html	Supplementary Data	Supplementary_Data_31.html	RT-qPCR analysis - SOX9 - outlier identification by the IQR method in R
Fig05	html	Supplementary Data	Supplementary_Data_32.html	RT-qPCR analysis - WNT4 - outlier identification by the IQR method in R
Fig05	html	Supplementary Data	Supplementary_Data_33.html	RT-qPCR analysis - NR5A1 - outlier identification by the IQR method in R
Fig05	html	Supplementary Data	Supplementary_Data_34.html	RT-qPCR analysis - FOXL2 - outlier identification by the IQR method in R
Fig05	html	Supplementary Data	Supplementary_Data_35.html	RT-qPCR analysis - FGF9 - outlier identification by the IQR method in R
Fig05B	xlsx	Supplementary Data	Supplementary_Data_36.xlsx	RT-qPCR analysis - SRY - Wild type versus mutant comparison
Fig05B	xlsx	Supplementary Data	Supplementary_Data_37.xlsx	RT-qPCR analysis - SOX9 - Wild type versus mutant comparison
Fig05B	xlsx	Supplementary Data	Supplementary_Data_38.xlsx	RT-qPCR analysis - WNT4 - Wild type versus mutant comparison
Fig05B	xlsx	Supplementary Data	Supplementary_Data_39.xlsx	RT-qPCR analysis - NR5A1 - Wild type versus mutant comparison
Fig05B	xlsx	Supplementary Data	Supplementary_Data_40.xlsx	RT-qPCR analysis - FOXL2 - Wild type versus mutant comparison
Fig05B	xlsx	Supplementary Data	Supplementary_Data_41.xlsx	RT-qPCR analysis - FGF9 - Wild type versus mutant comparison
Fig05A	xlsx	Supplementary Data	Supplementary_Data_42.xlsx	RT-qPCR analysis - iPS07-Wild type expression kinetics
Fig05A	xlsx	Supplementary Data	Supplementary_Data_43.xlsx	RT-qPCR analysis - iPS09-Wild type expression kinetics
Fig05A	xlsx	Supplementary Data	Supplementary_Data_44.xlsx	RT-qPCR analysis - iPS12-Wild type expression kinetics
Fig05A	xlsx	Supplementary Data	Supplementary_Data_45.xlsx	RT-qPCR analysis - iPS26a-Wild type expression kinetics
Fig05A	xlsx	Supplementary Data	Supplementary_Data_46.xlsx	RT-qPCR analysis - iPS26b-Wild type expression kinetics
Fig05	pzfx	Supplementary Data	Supplementary_Data_47.pzfx	RT-qPCR analysis: GraphPad Prism file summarizing all RT-qPCR experiment results
Fig02	pdf	Supplementary Figure	Supplementary_Figure_01.pdf	Phylogenetic tree of the mammalian species used to build figure 2
Fig03	pdf	Supplementary Figure	Supplementary_Figure_02.pdf	SRY mutant alleles Sanger sequencing
Fig03	pdf	Supplementary Figure	Supplementary_Figure_03.pdf	Coexpression of SRY, NR5A1 and SOX9 in the human developing gonad
Fig04	pdf	Supplementary Figure	Supplementary_Figure_04.pdf	SRY DNA NR5A1 protein interaction
Fig05	pdf	Supplementary Figure	Supplementary_Figure_05.pdf	rt-qPCR experiments for *SRY*, *SOX9*, *WNT4*, *NR5A1, FOXL2 and* FGF9 comparing wild type and mutant samples, with detailed analysis provided for each experiment in undifferentiated hiPSC.
Fig05	pdf	Supplementary Figure	Supplementary_Figure_06.pdf	*NR5A1* expression throughout the differentiation process of wild type hiPSCs.
Fig05	pdf	Supplementary Figure	Supplementary_Figure_07.pdf	*FGF9* expression throughout the differentiation process of wild type hiPSCs.

Fig05	pdf	Supplementary Figure	Supplementary_Figure_08.pdf	*FOXL2* expression throughout the differentiation process of wild type hiPSCs.
Fig05	pdf	Supplementary Figure	Supplementary_Figure_09.pdf	rt-qPCR experiments for *SRY*, *SOX9*, *WNT4*, *FOXL2* comparing wild type and mutant samples, with detailed analysis provided for each experiment and timepoint.
Fig05	pdf	Supplementary Figure	Supplementary_Figure_10.pdf	*NR5A1* and *FGF9* comparing wild type and mutant samples, with detailed analysis provided for each experiment and timepoint.
Fig05	pdf	Supplementary Figure	Supplementary_Figure_11.pdf	Comparison of *SRY*, *SOX9*, *NR5A1, WNT4, FOXL2, and FGF9* expression throughout the differentiation process of wild type hiPSCs.
Fig05	pdf	Supplementary Figure	Supplementary_Figure_12.pdf	Flowchart depicting the transformation of raw data into Figures
Fig03	pdf	Supplementary Table	Supplementary_Table_01.pdf	Table case report
