# Supplementary material for: A conserved NR5A1-responsive enhancer regulates SRY in testis-determination
Source: Nat Commun. 2024 Mar 30;15:2796. doi: 10.1038/s41467-024-47162-2 (PMC10981742; doi:10.1038/s41467-024-47162-2)
Supplement: Supplementary file 3 — Description of Additional Supplementary Files [file 41467_2024_47162_MOESM3_ESM.pdf]

Description of Additional Supplementary Files:

Supplementary Data 1: annotated SRY locus - CLC main workbench file - SRY - Complete locus

Supplementary Data 2: annotated SRY locus - CLC main workbench file - SRY - 7386bp sequence fragment

Supplementary Data 3: annotated SRY locus - CLC main workbench file - SRY - 83 species sequence list

Supplementary Data 4: annotated SRY locus - CLC main workbench file - SRY - 83 species sequence alignment

Supplementary Data 5: annotated SRY locus - CLC main workbench file - SRY - 17 species sequence alignment

Supplementary Data 6: List of the mammalian species used in this study

Supplementary Data 7: Genomatix predicted transcription binding sites in the E250 enhancer

Supplementary Data 8: H1a1 haplogroup

Supplementary Data 9: Luciferase: annotated Plasmid-pcDNA3-Myc2X-NR5A1 - CLC main workbench file

Supplementary Data 10: Luciferase: annotated Plasmid-pCMV-Renilla-AF025843 - CLC main workbench file

Supplementary Data 11: Luciferase: annotated Plasmid-pGL4.26_link5_E250-Reference - CLC main workbench file

Supplementary Data 12: Luciferase: annotated Plasmid-pGL4.26_link5_pGL4.26_link5_E250-Variant-1

Supplementary Data 13: Luciferase: annotated Plasmid-pGL4.26_link5_pGL4.26_link5_E250-Variant-2

Supplementary Data 14: Luciferase: annotated Plasmid-pGL4.26_link5_E250-ΔNR5A1 - CLC main workbench file

Supplementary Data 15: Luciferase on HEK293T cells - statistical analysis in R

Supplementary Data 16: Luciferase on HeLa cells - statistical analysis in R

Supplementary Data 17: RT-qPCR analysis - AMH - Wild type versus mutant comparison

Supplementary Data 18: RT-qPCR analysis - SRY - Linear Mixed Model in R

Supplementary Data 19: RT-qPCR analysis - SOX9 - Linear Mixed Model in R

Supplementary Data 20: RT-qPCR analysis - WNT4 - Linear Mixed Model in R

Supplementary Data 21: RT-qPCR analysis - NR5A1 - Linear Mixed Model in R

Supplementary Data 22: RT-qPCR analysis - FOXL2 - Linear Mixed Model in R

Supplementary Data 23: RT-qPCR analysis - FGF9 - Linear Mixed Model in R

Supplementary Data 24: RT-qPCR analysis - SRY - data preparation for Linear Mixed Model in R

Supplementary Data 25: RT-qPCR analysis - SOX9 - data preparation for Linear Mixed Model in R

Supplementary Data 26: RT-qPCR analysis - WNT4 - data preparation for Linear Mixed Model in R

Supplementary Data 27: RT-qPCR analysis - NR5A1 - data preparation for Linear Mixed Model in R

Supplementary Data 28: RT-qPCR analysis - FOXL2 - data preparation for Linear Mixed Model in R

Supplementary Data 29: RT-qPCR analysis - FGF9 - data preparation for Linear Mixed Model in R

Supplementary Data 30: RT-qPCR analysis - SRY - outlier identification by the IQR method in R

Supplementary Data 31: RT-qPCR analysis - SOX9 - outlier identification by the IQR method in R

Supplementary Data 32: RT-qPCR analysis - WNT4 - outlier identification by the IQR method in R

Supplementary Data 33: RT-qPCR analysis - NR5A1 - outlier identification by the IQR method in R

Supplementary Data 34: RT-qPCR analysis - FOXL2 - outlier identification by the IQR method in R

Supplementary Data 35: RT-qPCR analysis - FGF9 - outlier identification by the IQR method in R

Supplementary Data 36: RT-qPCR analysis - SRY - Wild type versus mutant comparison

Supplementary Data 37: RT-qPCR analysis - SOX9 - Wild type versus mutant comparison

Supplementary Data 38: RT-qPCR analysis - WNT4 - Wild type versus mutant comparison

Supplementary Data 39: RT-qPCR analysis - NR5A1 - Wild type versus mutant comparison

Supplementary Data 40: RT-qPCR analysis - FOXL2 - Wild type versus mutant comparison

Supplementary Data 41: RT-qPCR analysis - FGF9 - Wild type versus mutant comparison

Supplementary Data 42: RT-qPCR analysis - iPS07-Wild type expression kinetics

Supplementary Data 43: RT-qPCR analysis - iPS09-Wild type expression kinetics

Supplementary Data 44: RT-qPCR analysis - iPS12-Wild type expression kinetics

Supplementary Data 45: RT-qPCR analysis - iPS26a-Wild type expression kinetics

Supplementary Data 46: RT-qPCR analysis - iPS26b-Wild type expression kinetics

Supplementary Data 47: RT-qPCR analysis: GraphPad Prism file summarizing all RT-qPCR experiment results

Supplementary Data 48: Statistical tests - Figure 4 and Figure 5
