# Supplementary material for: A conserved NR5A1-responsive enhancer regulates SRY in testis-determination
Source: Nat Commun. 2024 Mar 30;15:2796. doi: 10.1038/s41467-024-47162-2 (PMC10981742; doi:10.1038/s41467-024-47162-2)
Supplement: Supplementary file 18 — Supplementary Dataset 15 [file 41467_2024_47162_MOESM18_ESM.html]

Supplementary\_Data\_15


# Supplementary\_Data\_15

#### Denis Houzelstein

#### 2024-02-21

Luciferase on HEK293T cells - statistical analysis in R

```
library(tidyverse)
```

```
## ── Attaching core tidyverse packages ──────────────────────── tidyverse 2.0.0 ──
## ✔ dplyr     1.1.4     ✔ readr     2.1.5
## ✔ forcats   1.0.0     ✔ stringr   1.5.1
## ✔ ggplot2   3.4.4     ✔ tibble    3.2.1
## ✔ lubridate 1.9.3     ✔ tidyr     1.3.0
## ✔ purrr     1.0.2     
## ── Conflicts ────────────────────────────────────────── tidyverse_conflicts() ──
## ✖ dplyr::filter() masks stats::filter()
## ✖ dplyr::lag()    masks stats::lag()
## ℹ Use the conflicted package (<http://conflicted.r-lib.org/>) to force all conflicts to become errors
```

```
library(scales) # scientific format
```

```
## 
## Attaching package: 'scales'
## 
## The following object is masked from 'package:purrr':
## 
##     discard
## 
## The following object is masked from 'package:readr':
## 
##     col_factor
```

```
library(ggpubr)
library(broom)
library(car)
```

```
## Loading required package: carData
## 
## Attaching package: 'car'
## 
## The following object is masked from 'package:dplyr':
## 
##     recode
## 
## The following object is masked from 'package:purrr':
## 
##     some
```

```
library(gtsummary)  
library(gt)
```

# 1 Data

data from Luciferase32a  
200307-32a\_Dual-Glo\_3-7-2020\_11-45-17 AM.xml  
200307-32a\_Dual-Glo\_3-7-2020\_11-45-17 AM.csv

## 1.1 Luciferase = Assayed Luciferase Levels

```
Input = (
  "
932165  280694  291766  7579    326610  294131  213808  287660  274511  285368  295928  284701
248281  343599  342646  308856  313576  301555  339357  286528  298245  296319  308823  298451
279594  286938  313191  270324  299091  294240  290934  262438  281495  278678  262717  275721
217401  237318  287149  273201  35584.4 266744  252756  266391  260908  265575  264828  258921
1973580 914549  1056410 1019270 992366  972704  952885  946102  974248  1038220 973668  4800
540331  527855  541003  512053  34843   485106  501617  496061  515398  483273  514919  536478
395287  394912  425305  408337  427163  435909  412991  406536  412621  399348  395261  445417
461254  448295  517611  505456  497184  477564  483230  496222  501602  490351  461691  502010
"
)
luciferase = read.delim(textConnection(Input), header = FALSE)

# Stack
luciferase <-
  luciferase |>
  stack() |>
  pull(values)
```

## 1.2 Renilla = Assayed Renilla Levels

```
Input = (
  "
2109200 3280310 3035520 3152070 3485520 3257440 1280750 3172730 3042680 3127310 3817540 3271880
2255220 3213440 3273870 2915530 2771810 2660370 2951870 2622030 2822620 2794690 2891570 2877910
2585680 2896890 3005140 2696110 3080790 2648400 2777170 2600100 2891280 2886650 2577850 2774690
2610100 2913220 3095140 2828140 627127  2820150 2482040 2807710 2674760 2666920 2753210 2618330
2086170 2117440 2206910 2129310 2146500 2172140 2102720 1995050 2079730 2082990 2107500 2122020
1823620 1745900 1897230 1692220 142261  1735220 1734090 1791410 1821090 1671730 1722000 1827930
1612790 1737550 1814510 1658700 1903090 1851850 1831140 1741370 1625390 1768190 1760830 1900310
1873670 1795110 2021290 2060980 2087650 1850110 1858120 2057470 2077500 2018600 1999450 2129300
"
)
renilla = read.delim(textConnection(Input), header = FALSE)

# Stack
renilla <-
  renilla |>
  stack() |>
  pull(values)
```

## 1.3 Names = Experimental Conditions

```
Input = (
  "
Reference   Reference   Reference   Reference   Reference   Reference   Reference   Reference   Reference   Reference   Reference   Reference
Variant_1   Variant_1   Variant_1   Variant_1   Variant_1   Variant_1   Variant_1   Variant_1   Variant_1   Variant_1   Variant_1   Variant_1
Variant_2   Variant_2   Variant_2   Variant_2   Variant_2   Variant_2   Variant_2   Variant_2   Variant_2   Variant_2   Variant_2   Variant_2
delNR5A1    delNR5A1    delNR5A1    delNR5A1    delNR5A1    delNR5A1    delNR5A1    delNR5A1    delNR5A1    delNR5A1    delNR5A1    delNR5A1
Reference+NR5A1 Reference+NR5A1 Reference+NR5A1 Reference+NR5A1 Reference+NR5A1 Reference+NR5A1 Reference+NR5A1 Reference+NR5A1 Reference+NR5A1 Reference+NR5A1 Reference+NR5A1 Reference+NR5A1
Variant_1+NR5A1 Variant_1+NR5A1 Variant_1+NR5A1 Variant_1+NR5A1 Variant_1+NR5A1 Variant_1+NR5A1 Variant_1+NR5A1 Variant_1+NR5A1 Variant_1+NR5A1 Variant_1+NR5A1 Variant_1+NR5A1 Variant_1+NR5A1
Variant_2+NR5A1 Variant_2+NR5A1 Variant_2+NR5A1 Variant_2+NR5A1 Variant_2+NR5A1 Variant_2+NR5A1 Variant_2+NR5A1 Variant_2+NR5A1 Variant_2+NR5A1 Variant_2+NR5A1 Variant_2+NR5A1 Variant_2+NR5A1
delNR5A1+NR5A1  delNR5A1+NR5A1  delNR5A1+NR5A1  delNR5A1+NR5A1  delNR5A1+NR5A1  delNR5A1+NR5A1  delNR5A1+NR5A1  delNR5A1+NR5A1  delNR5A1+NR5A1  delNR5A1+NR5A1  delNR5A1+NR5A1  delNR5A1+NR5A1"
)
names = read.delim(textConnection(Input), header = FALSE)

# Stack
names <-
  names |>
  stack() |>
  pull(values)
```

## 1.4 Reporter = Luciferase expressing vector

```
Input = (
  "
Reference   Reference   Reference   Reference   Reference   Reference   Reference   Reference   Reference   Reference   Reference   Reference
Variant_1   Variant_1   Variant_1   Variant_1   Variant_1   Variant_1   Variant_1   Variant_1   Variant_1   Variant_1   Variant_1   Variant_1
Variant_2   Variant_2   Variant_2   Variant_2   Variant_2   Variant_2   Variant_2   Variant_2   Variant_2   Variant_2   Variant_2   Variant_2
delNR5A1    delNR5A1    delNR5A1    delNR5A1    delNR5A1    delNR5A1    delNR5A1    delNR5A1    delNR5A1    delNR5A1    delNR5A1    delNR5A1
Reference   Reference   Reference   Reference   Reference   Reference   Reference   Reference   Reference   Reference   Reference   Reference
Variant_1   Variant_1   Variant_1   Variant_1   Variant_1   Variant_1   Variant_1   Variant_1   Variant_1   Variant_1   Variant_1   Variant_1
Variant_2   Variant_2   Variant_2   Variant_2   Variant_2   Variant_2   Variant_2   Variant_2   Variant_2   Variant_2   Variant_2   Variant_2
delNR5A1    delNR5A1    delNR5A1    delNR5A1    delNR5A1    delNR5A1    delNR5A1    delNR5A1    delNR5A1    delNR5A1    delNR5A1    delNR5A1"
)
reporter = read.delim(textConnection(Input), header = FALSE)
# Stack
reporter <-
  reporter |>
  stack() |>
  pull(values)
```

## 1.5 Factor1 = transcription factor (NR5A1)

```
Input = (
  "
-   -   -   -   -   -   -   -   -   -   -   -
-   -   -   -   -   -   -   -   -   -   -   -
-   -   -   -   -   -   -   -   -   -   -   -
-   -   -   -   -   -   -   -   -   -   -   -
NR5A1   NR5A1   NR5A1   NR5A1   NR5A1   NR5A1   NR5A1   NR5A1   NR5A1   NR5A1   NR5A1   NR5A1
NR5A1   NR5A1   NR5A1   NR5A1   NR5A1   NR5A1   NR5A1   NR5A1   NR5A1   NR5A1   NR5A1   NR5A1
NR5A1   NR5A1   NR5A1   NR5A1   NR5A1   NR5A1   NR5A1   NR5A1   NR5A1   NR5A1   NR5A1   NR5A1
NR5A1   NR5A1   NR5A1   NR5A1   NR5A1   NR5A1   NR5A1   NR5A1   NR5A1   NR5A1   NR5A1   NR5A1
"
)
factor1 = read.delim(textConnection(Input), header = FALSE)

# Stack
factor1 <-
  factor1 |>
  stack() |>
  pull(values)
```

# 2 Data Organization

## 2.1 Generate the dataframe

```
level_order<-c("Reference",
      "Variant_1",
      "Variant_2",
      "delNR5A1",
      "Reference+NR5A1",
      "Variant_1+NR5A1",
      "Variant_2+NR5A1",
      "delNR5A1+NR5A1")

dataframe0 <-
  tibble(names,
         reporter,
         factor1,
         renilla,
         luciferase) |>
  mutate_if(is.character, as.factor) |>
  arrange(factor(names, levels = level_order))
```

## 2.2 Exclude Renilla outliers by the Inter Quartile Range (IQR) method

```
boxplot(renilla ~ names, dataframe0)
```

```
dataframe1 <- dataframe0 |>
  group_by(names) |>
  mutate(
    IQR = IQR(renilla, na.rm = TRUE),
    Outlier_upper = quantile(renilla, probs = c(.75), na.rm = TRUE) + 1.5 * IQR,
    Outlier_lower = quantile(renilla, probs = c(.25), na.rm = TRUE) - 1.5 * IQR,
    renilla_wo_extremes = if_else(renilla <= Outlier_lower | renilla >= Outlier_upper, NA, renilla))

boxplot(renilla_wo_extremes ~ names, dataframe1)
```

## 2.3 New column containing the Luc/Ren Ratio Computation from Luc and Ren Columns

```
dataframe2 <-
  dataframe1 |> mutate(Luc_Ren = luciferase / renilla_wo_extremes)
```

## 2.4 Initial Data Exploration Through Scatterplots

### 2.4.1 Renilla

```
ggplot(dataframe2,
       aes(
         x = factor(names, level = level_order),
         y = renilla_wo_extremes,
         colour = reporter,
         group = names,
       )) +
  geom_boxplot() +
  geom_jitter(width = 0.2) +
  theme(axis.text.x = element_text(
    vjust = 1,
    hjust = 1,
    size = 10,
    angle = 30,
    family = "helvetica"
  )) + theme(
    panel.grid.major = element_line(colour = "gray85"),
    panel.grid.minor = element_line(colour = "gray90"),
    panel.background = element_rect(fill = NA)
  ) +
  scale_y_continuous(labels = scientific)
```

```
## Warning: Removed 10 rows containing non-finite values (`stat_boxplot()`).
```

```
## Warning: Removed 10 rows containing missing values (`geom_point()`).
```

### 2.4.2 Luciferase

```
ggplot(dataframe2,
       aes(
         x = factor(names, level = level_order),
         y = luciferase,
         colour = reporter,
         group = names
       )) +
  geom_boxplot() +
  geom_jitter(width = 0.2) +
  theme(axis.text.x = element_text(
    vjust = 1,
    hjust = 1,
    size = 10,
    angle = 30,
    family = "helvetica"
  )) + theme(
    panel.grid.major = element_line(colour = "gray85"),
    panel.grid.minor = element_line(colour = "gray90"),
    panel.background = element_rect(fill = NA)
  ) +
  scale_y_continuous(labels = scientific)
```

## 2.5 luciferase/renilla

```
ggplot(dataframe2,
       aes(
         x = factor(names, level = level_order),
         y = Luc_Ren,
         colour = reporter,
         group = names
       )) +
  geom_boxplot() +
  geom_jitter(width = 0.2) +
  theme(axis.text.x = element_text(
    vjust = 1,
    hjust = 1,
    size = 10,
    angle = 30,
    family = "helvetica"
  )) + theme(
    panel.grid.major = element_line(colour = "gray85"),
    panel.grid.minor = element_line(colour = "gray90"),
    panel.background = element_rect(fill = NA)
  ) +
  scale_y_continuous(labels = scientific)
```

```
## Warning: Removed 10 rows containing non-finite values (`stat_boxplot()`).
```

```
## Warning: Removed 10 rows containing missing values (`geom_point()`).
```

## 2.6 Remove the outliers from Luciferase/Renilla by the IQR method

```
boxplot(Luc_Ren ~ names, dataframe2)
```

```
dataframe3 <- dataframe2 |>
  group_by(names) |>
  mutate(
    IQR = IQR(Luc_Ren, na.rm = TRUE),
    Outlier_upper = quantile(Luc_Ren, probs = c(.75), na.rm = TRUE) + 1.5 * IQR,
    Outlier_lower = quantile(Luc_Ren, probs = c(.25), na.rm = TRUE) - 1.5 * IQR,
    Luc_Ren_wo_outliers = if_else(Luc_Ren <= Outlier_lower | Luc_Ren >= Outlier_upper, NA, Luc_Ren),
        )

boxplot(Luc_Ren_wo_outliers ~ names, dataframe3)
```

## 2.7 Calculate the relative ratio response (RRR)

see:  
RRR = (well\_value - mean\_neg))/(mean\_pos-mean\_neg)

Relative Response ratio (Promega - Dual-Glo® Luciferase Assay System,
Instructions for use of Products E2920, E2940 and E2980)

https://www.promega.com/-/media/files/resources/protocols/technical-manuals/0/dual-glo-luciferase-assay-system-protocol.pdf

```
dataframe4 <- dataframe3 |>
  group_by(names) |>
  mutate(mean = mean(Luc_Ren_wo_outliers, na.rm = TRUE)) |>
  ungroup() |>
  mutate(
    mean_neg = min(mean),
    mean_pos = max(mean),
    RRR = (Luc_Ren_wo_outliers - mean_neg) / (mean_pos - mean_neg),
    RRRp = RRR * 100,
    block = "a") |>
  mutate_if(is.character, as.factor)
```

mean\_pos = mean of the positive reference

mean\_neg = mean of the negative reference

RRR = Relative Response Ratio

RRRp = Relative Response Ratio in percent

block = necessary for the Approximative Two-Sample Fisher-Pitman
Permutation Test, it allows the stratification (it has to be a
factor)

```
ggplot(dataframe4,
       aes(
         x = factor(names, level = level_order),
         y = RRRp,
         colour = reporter,
         group = names
       )) +
  geom_boxplot() +
  geom_jitter(width = 0.2) +
  theme(axis.text.x = element_text(
    vjust = 1,
    hjust = 1,
    size = 10,
    angle = 30,
    family = "helvetica"
  )) + theme(
    panel.grid.major = element_line(colour = "gray85"),
    panel.grid.minor = element_line(colour = "gray90"),
    panel.background = element_rect(fill = NA)
  )  +
  ylim(-10,110)
```

```
## Warning: Removed 15 rows containing non-finite values (`stat_boxplot()`).
```

```
## Warning: Removed 15 rows containing missing values (`geom_point()`).
```

# 3 Statistical tests

## 3.1 Normality test

```
shapiro_test_result <- dataframe4 |> 
  group_by(names) |> 
  do(tidy(shapiro.test(.$RRRp)))

print(shapiro_test_result)
```

```
## # A tibble: 8 × 4
## # Groups:   names [8]
##   names           statistic p.value method                     
##   <fct>               <dbl>   <dbl> <chr>                      
## 1 delNR5A1            0.986  0.989  Shapiro-Wilk normality test
## 2 delNR5A1+NR5A1      0.959  0.773  Shapiro-Wilk normality test
## 3 Reference           0.959  0.802  Shapiro-Wilk normality test
## 4 Reference+NR5A1     0.983  0.976  Shapiro-Wilk normality test
## 5 Variant_1           0.884  0.172  Shapiro-Wilk normality test
## 6 Variant_1+NR5A1     0.950  0.638  Shapiro-Wilk normality test
## 7 Variant_2           0.919  0.280  Shapiro-Wilk normality test
## 8 Variant_2+NR5A1     0.877  0.0810 Shapiro-Wilk normality test
```

```
p_value <- min(shapiro_test_result$p.value)
p_value
```

```
## [1] 0.08097895
```

```
if (p_value < 0.05) {
  cat("The overall p-value obtained from the Bartlett test is less than 0.05, suggesting evidence supporting the rejection of the null hypothesis of homogeneity of variances.\n")
} else {
  cat("The overall p-value obtained from the Bartlett test is greater than or equal to 0.05, indicating insufficient evidence to reject the null hypothesis of homogeneity of variances.\n")
}
```

```
## The overall p-value obtained from the Bartlett test is greater than or equal to 0.05, indicating insufficient evidence to reject the null hypothesis of homogeneity of variances.
```

## 3.2 Homogeneity of variances

The conditions with or without the addition of the transcription
factor NR5A1 exhibited clearly distinct variances. As a result, a direct
comparison between these conditions is not informative.  
To assess the comparability of only conditions with NR5A1 added, both
Bartlett and Levene tests were conducted.

```
dataframe4b <- dataframe4 |>
  filter(factor1 == "NR5A1")

bartlett_test_result <- bartlett.test(RRRp ~ names, data = dataframe4b)
print(bartlett_test_result)
```

```
## 
##  Bartlett test of homogeneity of variances
## 
## data:  RRRp by names
## Bartlett's K-squared = 8.8015, df = 3, p-value = 0.03205
```

```
p_value <- bartlett_test_result$p.value
if (p_value < 0.05) {
  cat("The overall p-value obtained from the Bartlett test is less than 0.05, suggesting evidence supporting the rejection of the null hypothesis of homogeneity of variances.\n")
} else {
  cat("The overall p-value obtained from the Bartlett test is greater than or equal to 0.05, indicating insufficient evidence to reject the null hypothesis of homogeneity of variances.\n")
}
```

```
## The overall p-value obtained from the Bartlett test is less than 0.05, suggesting evidence supporting the rejection of the null hypothesis of homogeneity of variances.
```

```
levene_test_result <- leveneTest(RRRp ~ names, data = dataframe4b)
print(levene_test_result)
```

```
## Levene's Test for Homogeneity of Variance (center = median)
##       Df F value Pr(>F)
## group  3   1.847 0.1546
##       39
```

```
p_value <- levene_test_result$`Pr(>F)`[1]
if (p_value < 0.05) {
  cat("The overall p-value obtained from the Levene test is less than 0.05, suggesting evidence supporting the rejection of the null hypothesis of homogeneity of variances.\n")
} else {
  cat("The overall p-value obtained from the Levene test is greater than or equal to 0.05, indicating insufficient evidence to reject the null hypothesis of homogeneity of variances.\n")
}
```

```
## The overall p-value obtained from the Levene test is greater than or equal to 0.05, indicating insufficient evidence to reject the null hypothesis of homogeneity of variances.
```

```
dataframe5 <- dataframe4[dataframe4$factor1 == "NR5A1", ]
dataframe5$Other <- unname(vapply(as.character(dataframe5$names), FUN = function(x)  if(x == "Reference+NR5A1") x else "Other", FUN.VALUE = ""))
ggplot(dataframe5,
       aes(
         x = factor(names, level = level_order),
         y = RRRp,
         colour = reporter,
         group = names
       )) +
  geom_boxplot() +
  geom_jitter(width = 0.2) +
  theme(axis.text.x = element_text(
    vjust = 1,
    hjust = 1,
    size = 10,
    angle = 30
  )) + theme(
    panel.grid.major = element_line(colour = "gray85"),
    panel.grid.minor = element_line(colour = "gray90"),
    panel.background = element_rect(fill = NA)
  ) +
  scale_y_continuous(labels = scientific) +
  xlab(NULL)
```

```
## Warning: Removed 5 rows containing non-finite values (`stat_boxplot()`).
```

```
## Warning: Removed 5 rows containing missing values (`geom_point()`).
```

## 3.3 Wilcoxon rank sum exact test

```
wilcox.test(RRRp~Other, data = dataframe5)
```

```
## 
##  Wilcoxon rank sum exact test
## 
## data:  RRRp by Other
## W = 0, p-value = 1.379e-08
## alternative hypothesis: true location shift is not equal to 0
```

## 3.4 Kruskal-Wallis rank sum test

```
library(dunn.test)
bb <- dunn.test(dataframe5$RRRp, g = dataframe5$names, kw = T, list = T, method = "bh")
```

```
##   Kruskal-Wallis rank sum test
## 
## data: x and group
## Kruskal-Wallis chi-squared = 35.8827, df = 3, p-value = 0
## 
## 
##                            Comparison of x by group                            
##                              (Benjamini-Hochberg)                              
## Col Mean-|
## Row Mean |   delNR5A1   Referenc   Variant_
## ---------+---------------------------------
## Referenc |  -4.056706
##          |    0.0001*
##          |
## Variant_ |  -2.623347   1.628239
##          |    0.0065*     0.0621
##          |
## Variant_ |   1.463076   5.365322   4.054264
##          |     0.0717    0.0000*    0.0001*
## 
## 
## List of pairwise comparisons: Z statistic (adjusted p-value)
## -------------------------------------------------------
## delNR5A1+NR5A1 - Reference+NR5A1  : -4.056706 (0.0001)*
## delNR5A1+NR5A1 - Variant_1+NR5A1  : -2.623347 (0.0065)*
## Reference+NR5A1 - Variant_1+NR5A1 :  1.628239 (0.0621)
## delNR5A1+NR5A1 - Variant_2+NR5A1  :  1.463076 (0.0717)
## Reference+NR5A1 - Variant_2+NR5A1 :  5.365322 (0.0000)*
## Variant_1+NR5A1 - Variant_2+NR5A1 :  4.054264 (0.0001)*
## 
## alpha = 0.05
## Reject Ho if p <= alpha/2
```

# 4 End results

## 4.1 Plot

```
ggplot(dataframe4,
       aes(
         x = factor(names, level = level_order),
         y = RRRp,
         colour = reporter,
         group = names
       )) +
  geom_boxplot() +
  geom_jitter(width = 0.2) +
  theme(axis.text.x = element_text(
    vjust = 1,
    hjust = 1,
    size = 10,
    angle = 30
  )) + theme(
    panel.grid.major = element_line(colour = "gray85"),
    panel.grid.minor = element_line(colour = "gray90"),
    panel.background = element_rect(fill = NA)
  ) +
  scale_y_continuous(labels = scientific) +
  xlab(NULL)
```

```
## Warning: Removed 15 rows containing non-finite values (`stat_boxplot()`).
```

```
## Warning: Removed 15 rows containing missing values (`geom_point()`).
```

```
gt(dataframe5)
```

| names | reporter | factor1 | renilla | luciferase | IQR | Outlier\_upper | Outlier\_lower | renilla\_wo\_extremes | Luc\_Ren | Luc\_Ren\_wo\_outliers | mean | mean\_neg | mean\_pos | RRR | RRRp | block | Other |
| --- | --- | --- | --- | --- | --- | --- | --- | --- | --- | --- | --- | --- | --- | --- | --- | --- | --- |
| Reference+NR5A1 | Reference | NR5A1 | 2086170 | 1973580 | 0.02697775 | 0.5165931 | 0.4086821 | 2086170 | 0.946030285 | NA | 0.4628465 | 0.09060474 | 0.4628465 | NA | NA | a | Reference+NR5A1 |
| Reference+NR5A1 | Reference | NR5A1 | 2117440 | 914549 | 0.02697775 | 0.5165931 | 0.4086821 | 2117440 | 0.431912593 | 0.4319126 | 0.4628465 | 0.09060474 | 0.4628465 | 0.9168984 | 91.68984 | a | Reference+NR5A1 |
| Reference+NR5A1 | Reference | NR5A1 | 2206910 | 1056410 | 0.02697775 | 0.5165931 | 0.4086821 | NA | NA | NA | 0.4628465 | 0.09060474 | 0.4628465 | NA | NA | a | Reference+NR5A1 |
| Reference+NR5A1 | Reference | NR5A1 | 2129310 | 1019270 | 0.02697775 | 0.5165931 | 0.4086821 | 2129310 | 0.478685584 | 0.4786856 | 0.4628465 | 0.09060474 | 0.4628465 | 1.0425506 | 104.25506 | a | Reference+NR5A1 |
| Reference+NR5A1 | Reference | NR5A1 | 2146500 | 992366 | 0.02697775 | 0.5165931 | 0.4086821 | 2146500 | 0.462318192 | 0.4623182 | 0.4628465 | 0.09060474 | 0.4628465 | 0.9985808 | 99.85808 | a | Reference+NR5A1 |
| Reference+NR5A1 | Reference | NR5A1 | 2172140 | 972704 | 0.02697775 | 0.5165931 | 0.4086821 | 2172140 | 0.447809073 | 0.4478091 | 0.4628465 | 0.09060474 | 0.4628465 | 0.9596032 | 95.96032 | a | Reference+NR5A1 |
| Reference+NR5A1 | Reference | NR5A1 | 2102720 | 952885 | 0.02697775 | 0.5165931 | 0.4086821 | 2102720 | 0.453167802 | 0.4531678 | 0.4628465 | 0.09060474 | 0.4628465 | 0.9739990 | 97.39990 | a | Reference+NR5A1 |
| Reference+NR5A1 | Reference | NR5A1 | 1995050 | 946102 | 0.02697775 | 0.5165931 | 0.4086821 | NA | NA | NA | 0.4628465 | 0.09060474 | 0.4628465 | NA | NA | a | Reference+NR5A1 |
| Reference+NR5A1 | Reference | NR5A1 | 2079730 | 974248 | 0.02697775 | 0.5165931 | 0.4086821 | 2079730 | 0.468449270 | 0.4684493 | 0.4628465 | 0.09060474 | 0.4628465 | 1.0150515 | 101.50515 | a | Reference+NR5A1 |
| Reference+NR5A1 | Reference | NR5A1 | 2082990 | 1038220 | 0.02697775 | 0.5165931 | 0.4086821 | 2082990 | 0.498427741 | 0.4984277 | 0.4628465 | 0.09060474 | 0.4628465 | 1.0955865 | 109.55865 | a | Reference+NR5A1 |
| Reference+NR5A1 | Reference | NR5A1 | 2107500 | 973668 | 0.02697775 | 0.5165931 | 0.4086821 | 2107500 | 0.462001423 | 0.4620014 | 0.4628465 | 0.09060474 | 0.4628465 | 0.9977299 | 99.77299 | a | Reference+NR5A1 |
| Reference+NR5A1 | Reference | NR5A1 | 2122020 | 4800 | 0.02697775 | 0.5165931 | 0.4086821 | 2122020 | 0.002261996 | NA | 0.4628465 | 0.09060474 | 0.4628465 | NA | NA | a | Reference+NR5A1 |
| Variant\_1+NR5A1 | Variant\_1 | NR5A1 | 1823620 | 540331 | 0.01357464 | 0.3180218 | 0.2637232 | 1823620 | 0.296295829 | 0.2962958 | 0.2906128 | 0.09060474 | 0.4628465 | 0.5525740 | 55.25740 | a | Other |
| Variant\_1+NR5A1 | Variant\_1 | NR5A1 | 1745900 | 527855 | 0.01357464 | 0.3180218 | 0.2637232 | 1745900 | 0.302339767 | 0.3023398 | 0.2906128 | 0.09060474 | 0.4628465 | 0.5688106 | 56.88106 | a | Other |
| Variant\_1+NR5A1 | Variant\_1 | NR5A1 | 1897230 | 541003 | 0.01357464 | 0.3180218 | 0.2637232 | 1897230 | 0.285154146 | 0.2851541 | 0.2906128 | 0.09060474 | 0.4628465 | 0.5226427 | 52.26427 | a | Other |
| Variant\_1+NR5A1 | Variant\_1 | NR5A1 | 1692220 | 512053 | 0.01357464 | 0.3180218 | 0.2637232 | 1692220 | 0.302592453 | 0.3025925 | 0.2906128 | 0.09060474 | 0.4628465 | 0.5694894 | 56.94894 | a | Other |
| Variant\_1+NR5A1 | Variant\_1 | NR5A1 | 142261 | 34843 | 0.01357464 | 0.3180218 | 0.2637232 | NA | NA | NA | 0.2906128 | 0.09060474 | 0.4628465 | NA | NA | a | Other |
| Variant\_1+NR5A1 | Variant\_1 | NR5A1 | 1735220 | 485106 | 0.01357464 | 0.3180218 | 0.2637232 | 1735220 | 0.279564551 | 0.2795646 | 0.2906128 | 0.09060474 | 0.4628465 | 0.5076266 | 50.76266 | a | Other |
| Variant\_1+NR5A1 | Variant\_1 | NR5A1 | 1734090 | 501617 | 0.01357464 | 0.3180218 | 0.2637232 | 1734090 | 0.289268146 | 0.2892681 | 0.2906128 | 0.09060474 | 0.4628465 | 0.5336946 | 53.36946 | a | Other |
| Variant\_1+NR5A1 | Variant\_1 | NR5A1 | 1791410 | 496061 | 0.01357464 | 0.3180218 | 0.2637232 | 1791410 | 0.276910925 | 0.2769109 | 0.2906128 | 0.09060474 | 0.4628465 | 0.5004979 | 50.04979 | a | Other |
| Variant\_1+NR5A1 | Variant\_1 | NR5A1 | 1821090 | 515398 | 0.01357464 | 0.3180218 | 0.2637232 | 1821090 | 0.283016216 | 0.2830162 | 0.2906128 | 0.09060474 | 0.4628465 | 0.5168993 | 51.68993 | a | Other |
| Variant\_1+NR5A1 | Variant\_1 | NR5A1 | 1671730 | 483273 | 0.01357464 | 0.3180218 | 0.2637232 | 1671730 | 0.289085558 | 0.2890856 | 0.2906128 | 0.09060474 | 0.4628465 | 0.5332041 | 53.32041 | a | Other |
| Variant\_1+NR5A1 | Variant\_1 | NR5A1 | 1722000 | 514919 | 0.01357464 | 0.3180218 | 0.2637232 | 1722000 | 0.299023810 | 0.2990238 | 0.2906128 | 0.09060474 | 0.4628465 | 0.5599025 | 55.99025 | a | Other |
| Variant\_1+NR5A1 | Variant\_1 | NR5A1 | 1827930 | 536478 | 0.01357464 | 0.3180218 | 0.2637232 | 1827930 | 0.293489357 | 0.2934894 | 0.2906128 | 0.09060474 | 0.4628465 | 0.5450346 | 54.50346 | a | Other |
| Variant\_2+NR5A1 | Variant\_2 | NR5A1 | 1612790 | 395287 | 0.01204423 | 0.2558834 | 0.2077065 | 1612790 | 0.245095146 | 0.2450951 | 0.2341973 | 0.09060474 | 0.4628465 | 0.4150271 | 41.50271 | a | Other |
| Variant\_2+NR5A1 | Variant\_2 | NR5A1 | 1737550 | 394912 | 0.01204423 | 0.2558834 | 0.2077065 | 1737550 | 0.227280942 | 0.2272809 | 0.2341973 | 0.09060474 | 0.4628465 | 0.3671706 | 36.71706 | a | Other |
| Variant\_2+NR5A1 | Variant\_2 | NR5A1 | 1814510 | 425305 | 0.01204423 | 0.2558834 | 0.2077065 | 1814510 | 0.234391103 | 0.2343911 | 0.2341973 | 0.09060474 | 0.4628465 | 0.3862715 | 38.62715 | a | Other |
| Variant\_2+NR5A1 | Variant\_2 | NR5A1 | 1658700 | 408337 | 0.01204423 | 0.2558834 | 0.2077065 | 1658700 | 0.246178935 | 0.2461789 | 0.2341973 | 0.09060474 | 0.4628465 | 0.4179386 | 41.79386 | a | Other |
| Variant\_2+NR5A1 | Variant\_2 | NR5A1 | 1903090 | 427163 | 0.01204423 | 0.2558834 | 0.2077065 | 1903090 | 0.224457593 | 0.2244576 | 0.2341973 | 0.09060474 | 0.4628465 | 0.3595858 | 35.95858 | a | Other |
| Variant\_2+NR5A1 | Variant\_2 | NR5A1 | 1851850 | 435909 | 0.01204423 | 0.2558834 | 0.2077065 | 1851850 | 0.235391095 | 0.2353911 | 0.2341973 | 0.09060474 | 0.4628465 | 0.3889579 | 38.89579 | a | Other |
| Variant\_2+NR5A1 | Variant\_2 | NR5A1 | 1831140 | 412991 | 0.01204423 | 0.2558834 | 0.2077065 | 1831140 | 0.225537643 | 0.2255376 | 0.2341973 | 0.09060474 | 0.4628465 | 0.3624873 | 36.24873 | a | Other |
| Variant\_2+NR5A1 | Variant\_2 | NR5A1 | 1741370 | 406536 | 0.01204423 | 0.2558834 | 0.2077065 | 1741370 | 0.233457565 | 0.2334576 | 0.2341973 | 0.09060474 | 0.4628465 | 0.3837636 | 38.37636 | a | Other |
| Variant\_2+NR5A1 | Variant\_2 | NR5A1 | 1625390 | 412621 | 0.01204423 | 0.2558834 | 0.2077065 | 1625390 | 0.253859689 | 0.2538597 | 0.2341973 | 0.09060474 | 0.4628465 | 0.4385724 | 43.85724 | a | Other |
| Variant\_2+NR5A1 | Variant\_2 | NR5A1 | 1768190 | 399348 | 0.01204423 | 0.2558834 | 0.2077065 | 1768190 | 0.225851294 | 0.2258513 | 0.2341973 | 0.09060474 | 0.4628465 | 0.3633299 | 36.33299 | a | Other |
| Variant\_2+NR5A1 | Variant\_2 | NR5A1 | 1760830 | 395261 | 0.01204423 | 0.2558834 | 0.2077065 | 1760830 | 0.224474254 | 0.2244743 | 0.2341973 | 0.09060474 | 0.4628465 | 0.3596306 | 35.96306 | a | Other |
| Variant\_2+NR5A1 | Variant\_2 | NR5A1 | 1900310 | 445417 | 0.01204423 | 0.2558834 | 0.2077065 | 1900310 | 0.234391757 | 0.2343918 | 0.2341973 | 0.09060474 | 0.4628465 | 0.3862732 | 38.62732 | a | Other |
| delNR5A1+NR5A1 | delNR5A1 | NR5A1 | 1873670 | 461254 | 0.01089407 | 0.2676594 | 0.2240831 | 1873670 | 0.246176755 | 0.2461768 | 0.2454832 | 0.09060474 | 0.4628465 | 0.4179328 | 41.79328 | a | Other |
| delNR5A1+NR5A1 | delNR5A1 | NR5A1 | 1795110 | 448295 | 0.01089407 | 0.2676594 | 0.2240831 | 1795110 | 0.249731214 | 0.2497312 | 0.2454832 | 0.09060474 | 0.4628465 | 0.4274816 | 42.74816 | a | Other |
| delNR5A1+NR5A1 | delNR5A1 | NR5A1 | 2021290 | 517611 | 0.01089407 | 0.2676594 | 0.2240831 | 2021290 | 0.256079533 | 0.2560795 | 0.2454832 | 0.09060474 | 0.4628465 | 0.4445359 | 44.45359 | a | Other |
| delNR5A1+NR5A1 | delNR5A1 | NR5A1 | 2060980 | 505456 | 0.01089407 | 0.2676594 | 0.2240831 | 2060980 | 0.245250318 | 0.2452503 | 0.2454832 | 0.09060474 | 0.4628465 | 0.4154440 | 41.54440 | a | Other |
| delNR5A1+NR5A1 | delNR5A1 | NR5A1 | 2087650 | 497184 | 0.01089407 | 0.2676594 | 0.2240831 | 2087650 | 0.238154863 | 0.2381549 | 0.2454832 | 0.09060474 | 0.4628465 | 0.3963825 | 39.63825 | a | Other |
| delNR5A1+NR5A1 | delNR5A1 | NR5A1 | 1850110 | 477564 | 0.01089407 | 0.2676594 | 0.2240831 | 1850110 | 0.258127355 | 0.2581274 | 0.2454832 | 0.09060474 | 0.4628465 | 0.4500372 | 45.00372 | a | Other |
| delNR5A1+NR5A1 | delNR5A1 | NR5A1 | 1858120 | 483230 | 0.01089407 | 0.2676594 | 0.2240831 | 1858120 | 0.260063936 | 0.2600639 | 0.2454832 | 0.09060474 | 0.4628465 | 0.4552397 | 45.52397 | a | Other |
| delNR5A1+NR5A1 | delNR5A1 | NR5A1 | 2057470 | 496222 | 0.01089407 | 0.2676594 | 0.2240831 | 2057470 | 0.241180673 | 0.2411807 | 0.2454832 | 0.09060474 | 0.4628465 | 0.4045112 | 40.45112 | a | Other |
| delNR5A1+NR5A1 | delNR5A1 | NR5A1 | 2077500 | 501602 | 0.01089407 | 0.2676594 | 0.2240831 | 2077500 | 0.241445006 | 0.2414450 | 0.2454832 | 0.09060474 | 0.4628465 | 0.4052213 | 40.52213 | a | Other |
| delNR5A1+NR5A1 | delNR5A1 | NR5A1 | 2018600 | 490351 | 0.01089407 | 0.2676594 | 0.2240831 | 2018600 | 0.242916378 | 0.2429164 | 0.2454832 | 0.09060474 | 0.4628465 | 0.4091740 | 40.91740 | a | Other |
| delNR5A1+NR5A1 | delNR5A1 | NR5A1 | 1999450 | 461691 | 0.01089407 | 0.2676594 | 0.2240831 | 1999450 | 0.230909000 | 0.2309090 | 0.2454832 | 0.09060474 | 0.4628465 | 0.3769171 | 37.69171 | a | Other |
| delNR5A1+NR5A1 | delNR5A1 | NR5A1 | 2129300 | 502010 | 0.01089407 | 0.2676594 | 0.2240831 | 2129300 | 0.235762927 | 0.2357629 | 0.2454832 | 0.09060474 | 0.4628465 | 0.3899568 | 38.99568 | a | Other |

```
library(gtsummary)
dataframe4 |> tbl_summary(
  type = all_continuous() ~ "continuous2",
  include = c(names, RRRp),
  by = names,
  statistic = all_continuous() ~ c("{median}","{mean}", "{sd}", "{min}", "{max}"),
  digits = list(
              all_categorical() ~c(0,1),
              all_continuous() ~ c(1,1)),
  missing_text = "NA"
  )|>
  bold_labels() |>
  italicize_levels()
```

| **Characteristic** | **delNR5A1**, N = 12 | **delNR5A1+NR5A1**, N = 12 | **Reference**, N = 12 | **Reference+NR5A1**, N = 12 | **Variant\_1**, N = 12 | **Variant\_1+NR5A1**, N = 12 | **Variant\_2**, N = 12 | **Variant\_2+NR5A1**, N = 12 |
| --- | --- | --- | --- | --- | --- | --- | --- | --- |
| RRRp |  |  |  |  |  |  |  |  |
| Median | 1.6 | 41.2 | 0.0 | 99.8 | 4.4 | 53.4 | 2.7 | 38.5 |
| Mean | 1.7 | 41.6 | 0.0 | 100.0 | 4.9 | 53.7 | 3.0 | 38.6 |
| SD | 0.8 | 2.4 | 0.9 | 5.4 | 1.1 | 2.4 | 1.2 | 2.6 |
| Minimum | 0.6 | 37.7 | -1.4 | 91.7 | 3.5 | 50.0 | 1.6 | 36.0 |
| Maximum | 3.0 | 45.5 | 1.5 | 109.6 | 6.5 | 56.9 | 5.5 | 43.9 |
| NA | 3 | 0 | 4 | 4 | 3 | 1 | 0 | 0 |

```
sessionInfo()
```

```
## R version 4.3.2 (2023-10-31)
## Platform: aarch64-apple-darwin20 (64-bit)
## Running under: macOS Sonoma 14.3.1
## 
## Matrix products: default
## BLAS:   /Library/Frameworks/R.framework/Versions/4.3-arm64/Resources/lib/libRblas.0.dylib 
## LAPACK: /Library/Frameworks/R.framework/Versions/4.3-arm64/Resources/lib/libRlapack.dylib;  LAPACK version 3.11.0
## 
## locale:
## [1] en_US.UTF-8/en_US.UTF-8/en_US.UTF-8/C/en_US.UTF-8/en_US.UTF-8
## 
## time zone: Europe/Paris
## tzcode source: internal
## 
## attached base packages:
## [1] stats     graphics  grDevices utils     datasets  methods   base     
## 
## other attached packages:
##  [1] dunn.test_1.3.5 gt_0.10.1       gtsummary_1.7.2 car_3.1-2      
##  [5] carData_3.0-5   broom_1.0.5     ggpubr_0.6.0    scales_1.3.0   
##  [9] lubridate_1.9.3 forcats_1.0.0   stringr_1.5.1   dplyr_1.1.4    
## [13] purrr_1.0.2     readr_2.1.5     tidyr_1.3.0     tibble_3.2.1   
## [17] ggplot2_3.4.4   tidyverse_2.0.0
## 
## loaded via a namespace (and not attached):
##  [1] sass_0.4.8           utf8_1.2.4           generics_0.1.3      
##  [4] xml2_1.3.6           rstatix_0.7.2        stringi_1.8.3       
##  [7] hms_1.1.3            digest_0.6.34        magrittr_2.0.3      
## [10] evaluate_0.23        grid_4.3.2           timechange_0.3.0    
## [13] fastmap_1.1.1        broom.helpers_1.14.0 jsonlite_1.8.8      
## [16] backports_1.4.1      fansi_1.0.6          jquerylib_0.1.4     
## [19] abind_1.4-5          cli_3.6.2            rlang_1.1.3         
## [22] commonmark_1.9.0     munsell_0.5.0        withr_3.0.0         
## [25] cachem_1.0.8         yaml_2.3.8           tools_4.3.2         
## [28] tzdb_0.4.0           ggsignif_0.6.4       colorspace_2.1-0    
## [31] vctrs_0.6.5          R6_2.5.1             lifecycle_1.0.4     
## [34] pkgconfig_2.0.3      pillar_1.9.0         bslib_0.6.1         
## [37] gtable_0.3.4         glue_1.7.0           highr_0.10          
## [40] xfun_0.41            tidyselect_1.2.0     rstudioapi_0.15.0   
## [43] knitr_1.45           farver_2.1.1         htmltools_0.5.7     
## [46] labeling_0.4.3       rmarkdown_2.25       compiler_4.3.2      
## [49] markdown_1.12
```
