# Supplementary material for: A conserved NR5A1-responsive enhancer regulates SRY in testis-determination
Source: Nat Commun. 2024 Mar 30;15:2796. doi: 10.1038/s41467-024-47162-2 (PMC10981742; doi:10.1038/s41467-024-47162-2)
Supplement: Supplementary file 19 — Supplementary Dataset 16 [file 41467_2024_47162_MOESM19_ESM.html]

Supplementary\_Data\_16


# Supplementary\_Data\_16

#### Denis Houzelstein

#### 2024-02-21

Luciferase on HeLa cells - statistical analysis in R

```
library(tidyverse)
```

```
## ── Attaching core tidyverse packages ──────────────────────── tidyverse 2.0.0 ──
## ✔ dplyr     1.1.4     ✔ readr     2.1.5
## ✔ forcats   1.0.0     ✔ stringr   1.5.1
## ✔ ggplot2   3.4.4     ✔ tibble    3.2.1
## ✔ lubridate 1.9.3     ✔ tidyr     1.3.0
## ✔ purrr     1.0.2     
## ── Conflicts ────────────────────────────────────────── tidyverse_conflicts() ──
## ✖ dplyr::filter() masks stats::filter()
## ✖ dplyr::lag()    masks stats::lag()
## ℹ Use the conflicted package (<http://conflicted.r-lib.org/>) to force all conflicts to become errors
```

```
library(scales) # scientific format
```

```
## 
## Attaching package: 'scales'
## 
## The following object is masked from 'package:purrr':
## 
##     discard
## 
## The following object is masked from 'package:readr':
## 
##     col_factor
```

```
library(coin) # Approximative Two-Sample Fisher-Pitman Permutation Test
```

```
## Loading required package: survival
## 
## Attaching package: 'coin'
## 
## The following object is masked from 'package:scales':
## 
##     pvalue
```

```
library(ggpubr)
library(broom)
library(car)
```

```
## Loading required package: carData
## 
## Attaching package: 'car'
## 
## The following object is masked from 'package:dplyr':
## 
##     recode
## 
## The following object is masked from 'package:purrr':
## 
##     some
```

```
library(gtsummary)  
library(gt)
```

# 1 Data

data from Luciferase42  
42-HeLa\_Dual-Glo\_1-26-2023\_10-34-21 AM.xml  
42-HeLa\_Dual-Glo\_1-26-2023\_10-34-21 AM.csv

## 1.1 Luciferase = Assayed Luciferase Levels

```
Input = (
  "
60226   61051.7 66876.5 63589.5 63154.9 50962.8 55427   75472.8 71021.3 4249.85 70619.6 2421.28
48795.7 55075.2 53277.1 50819.1 53345.4 56443.3 65771.7 66978.2 69013.1 3680.64 1566.12 2107.21
60512.6 71297.1 77161.8 79652.1 70548.1 83508.5 97500.8 80641.5 84564.8 4618    2496.29 64830.9
44968.9 37731.8 43665.4 39291.4 3004.42 46096.6 49983.1 47138.2 62740.5 3152.47 56180   2672.34
56730.9 55696.4 59950.5 57197.4 88169.9 70541.1 89618.9 73322.8 82763.7 4710.04 1931.18 59933.4
45198.8 38547.7 41822.1 42741.7 50140.9 44617.4 62920.5 64911.4 61763.8 3294.51 1393.09 64251.4
53246.9 51343.6 45468   45739.1 2986.42 62682.1 72998.6 70233.1 72270.7 4382.9  1706.14 70032.8
35852.3 35955.7 38397.2 34042.4 4469.94 42973.6 47442.6 51043.2 50613.1 3449.56 1075.05 49453.7
"
)
luciferase = read.delim(textConnection(Input), header = FALSE)

# Stack
luciferase <-
  luciferase |>
  stack() |>
  pull(values)
```

## 1.2 Renilla = Assayed Renilla Levels

```
Input = (
  "
1472410 1562440 1687790 1524190 1610360 2012250 1473980 1911120 1770930 119287  1859870 81448.6
1544910 1802320 1714390 1557590 1711720 1718070 2002650 1886280 2109530 130234  51273.3 78355.5
1219240 1549310 1746110 1621560 1472070 1588340 1862590 1715340 1824380 100317  53486.1 1456070
1488500 1151550 1555550 1256780 71388.7 1531720 1810250 1703350 2166170 104416  2244400 97044.6
473446  495631  484832  494818  593864  555759  646673  536634  592993  36610.9 13402.4 529804
563558  490290  523060  587296  683813  584700  657799  714926  692377  35828.2 15697.6 659306
519301  529884  525803  498938  31775.4 586286  671262  634343  679095  40432.7 15395.1 618741
492362  589801  583858  631868  72923.1 571305  735373  649126  672333  46740.5 14423.8 633089
"
)
renilla = read.delim(textConnection(Input), header = FALSE)

# Stack
renilla <-
  renilla |>
  stack() |>
  pull(values)
```

## 1.3 Names = Experimental Conditions

```
Input = (
  "
Reference   Reference   Reference   Reference   Reference   Reference   Reference   Reference   Reference   Reference   Reference   Reference
Variant_1   Variant_1   Variant_1   Variant_1   Variant_1   Variant_1   Variant_1   Variant_1   Variant_1   Variant_1   Variant_1   Variant_1
Variant_2   Variant_2   Variant_2   Variant_2   Variant_2   Variant_2   Variant_2   Variant_2   Variant_2   Variant_2   Variant_2   Variant_2
delNR5A1    delNR5A1    delNR5A1    delNR5A1    delNR5A1    delNR5A1    delNR5A1    delNR5A1    delNR5A1    delNR5A1    delNR5A1    delNR5A1
Reference+NR5A1 Reference+NR5A1 Reference+NR5A1 Reference+NR5A1 Reference+NR5A1 Reference+NR5A1 Reference+NR5A1 Reference+NR5A1 Reference+NR5A1 Reference+NR5A1 Reference+NR5A1 Reference+NR5A1
Variant_1+NR5A1 Variant_1+NR5A1 Variant_1+NR5A1 Variant_1+NR5A1 Variant_1+NR5A1 Variant_1+NR5A1 Variant_1+NR5A1 Variant_1+NR5A1 Variant_1+NR5A1 Variant_1+NR5A1 Variant_1+NR5A1 Variant_1+NR5A1
Variant_2+NR5A1 Variant_2+NR5A1 Variant_2+NR5A1 Variant_2+NR5A1 Variant_2+NR5A1 Variant_2+NR5A1 Variant_2+NR5A1 Variant_2+NR5A1 Variant_2+NR5A1 Variant_2+NR5A1 Variant_2+NR5A1 Variant_2+NR5A1
delNR5A1+NR5A1  delNR5A1+NR5A1  delNR5A1+NR5A1  delNR5A1+NR5A1  delNR5A1+NR5A1  delNR5A1+NR5A1  delNR5A1+NR5A1  delNR5A1+NR5A1  delNR5A1+NR5A1  delNR5A1+NR5A1  delNR5A1+NR5A1  delNR5A1+NR5A1"
)
names = read.delim(textConnection(Input), header = FALSE)

# Stack
names <-
  names |>
  stack() |>
  pull(values)
```

## 1.4 Reporter = Luciferase expressing vector

```
Input = (
  "
Reference   Reference   Reference   Reference   Reference   Reference   Reference   Reference   Reference   Reference   Reference   Reference
Variant_1   Variant_1   Variant_1   Variant_1   Variant_1   Variant_1   Variant_1   Variant_1   Variant_1   Variant_1   Variant_1   Variant_1
Variant_2   Variant_2   Variant_2   Variant_2   Variant_2   Variant_2   Variant_2   Variant_2   Variant_2   Variant_2   Variant_2   Variant_2
delNR5A1    delNR5A1    delNR5A1    delNR5A1    delNR5A1    delNR5A1    delNR5A1    delNR5A1    delNR5A1    delNR5A1    delNR5A1    delNR5A1
Reference   Reference   Reference   Reference   Reference   Reference   Reference   Reference   Reference   Reference   Reference   Reference
Variant_1   Variant_1   Variant_1   Variant_1   Variant_1   Variant_1   Variant_1   Variant_1   Variant_1   Variant_1   Variant_1   Variant_1
Variant_2   Variant_2   Variant_2   Variant_2   Variant_2   Variant_2   Variant_2   Variant_2   Variant_2   Variant_2   Variant_2   Variant_2
delNR5A1    delNR5A1    delNR5A1    delNR5A1    delNR5A1    delNR5A1    delNR5A1    delNR5A1    delNR5A1    delNR5A1    delNR5A1    delNR5A1"
)
reporter = read.delim(textConnection(Input), header = FALSE)
# Stack
reporter <-
  reporter |>
  stack() |>
  pull(values)
```

## 1.5 Factor1 = transcription factor (NR5A1)

```
Input = (
  "
-   -   -   -   -   -   -   -   -   -   -   -
-   -   -   -   -   -   -   -   -   -   -   -
-   -   -   -   -   -   -   -   -   -   -   -
-   -   -   -   -   -   -   -   -   -   -   -
NR5A1   NR5A1   NR5A1   NR5A1   NR5A1   NR5A1   NR5A1   NR5A1   NR5A1   NR5A1   NR5A1   NR5A1
NR5A1   NR5A1   NR5A1   NR5A1   NR5A1   NR5A1   NR5A1   NR5A1   NR5A1   NR5A1   NR5A1   NR5A1
NR5A1   NR5A1   NR5A1   NR5A1   NR5A1   NR5A1   NR5A1   NR5A1   NR5A1   NR5A1   NR5A1   NR5A1
NR5A1   NR5A1   NR5A1   NR5A1   NR5A1   NR5A1   NR5A1   NR5A1   NR5A1   NR5A1   NR5A1   NR5A1
"
)
factor1 = read.delim(textConnection(Input), header = FALSE)

# Stack
factor1 <-
  factor1 |>
  stack() |>
  pull(values)
```

# 2 Data Organization

## 2.1 Generate the dataframe

```
level_order<-c("Reference",
      "Variant_1",
      "Variant_2",
      "delNR5A1",
      "Reference+NR5A1",
      "Variant_1+NR5A1",
      "Variant_2+NR5A1",
      "delNR5A1+NR5A1")

dataframe0 <-
  tibble(names,
         reporter,
         factor1,
         renilla,
         luciferase) |>
  mutate_if(is.character, as.factor) |>
  arrange(factor(names, levels = level_order))
```

## 2.2 Exclude Renilla outliers by the Inter Quartile Range (IQR) method

```
boxplot(renilla ~ names, dataframe0)
```

```
dataframe1 <- dataframe0 |>
  group_by(names) |>
  mutate(
    IQR = IQR(renilla, na.rm = TRUE),
    Outlier_upper = quantile(renilla, probs = c(.75), na.rm = TRUE) + 1.5 * IQR,
    Outlier_lower = quantile(renilla, probs = c(.25), na.rm = TRUE) - 1.5 * IQR,
    renilla_wo_extremes = if_else(renilla <= Outlier_lower | renilla >= Outlier_upper, NA, renilla))

boxplot(renilla_wo_extremes ~ names, dataframe1)
```

## 2.3 New column containing the Luc/Ren Ratio Computation from Luc and Ren Columns

```
dataframe2 <-
  dataframe1 |> mutate(Luc_Ren = luciferase / renilla_wo_extremes)
```

## 2.4 Initial Data Exploration Through Scatterplots

### 2.4.1 Renilla

```
ggplot(dataframe2,
       aes(
         x = factor(names, level = level_order),
         y = renilla_wo_extremes,
         colour = reporter,
         group = names,
       )) +
  geom_boxplot() +
  geom_jitter(width = 0.2) +
  theme(axis.text.x = element_text(
    vjust = 1,
    hjust = 1,
    size = 10,
    angle = 30
  )) + theme(
    panel.grid.major = element_line(colour = "gray85"),
    panel.grid.minor = element_line(colour = "gray90"),
    panel.background = element_rect(fill = NA)
  ) +
  scale_y_continuous(labels = scientific)
```

```
## Warning: Removed 12 rows containing non-finite values (`stat_boxplot()`).
```

```
## Warning: Removed 12 rows containing missing values (`geom_point()`).
```

### 2.4.2 Luciferase

```
ggplot(dataframe2,
       aes(
         x = factor(names, level = level_order),
         y = luciferase,
         colour = reporter,
         group = names
       )) +
  geom_boxplot() +
  geom_jitter(width = 0.2) +
  theme(axis.text.x = element_text(
    vjust = 1,
    hjust = 1,
    size = 10,
    angle = 30
  )) + theme(
    panel.grid.major = element_line(colour = "gray85"),
    panel.grid.minor = element_line(colour = "gray90"),
    panel.background = element_rect(fill = NA)
  ) +
  scale_y_continuous(labels = scientific)
```

## 2.5 luciferase/renilla

```
ggplot(dataframe2,
       aes(
         x = factor(names, level = level_order),
         y = Luc_Ren,
         colour = reporter,
         group = names
       )) +
  geom_boxplot() +
  geom_jitter(width = 0.2) +
  theme(axis.text.x = element_text(
    vjust = 1,
    hjust = 1,
    size = 10,
    angle = 30
  )) + theme(
    panel.grid.major = element_line(colour = "gray85"),
    panel.grid.minor = element_line(colour = "gray90"),
    panel.background = element_rect(fill = NA)
  ) +
  scale_y_continuous(labels = scientific)
```

```
## Warning: Removed 12 rows containing non-finite values (`stat_boxplot()`).
```

```
## Warning: Removed 12 rows containing missing values (`geom_point()`).
```

## 2.6 Remove the outliers from Luciferase/Renilla by the IQR method

```
boxplot(Luc_Ren ~ names, dataframe2)
```

```
dataframe3 <- dataframe2 |>
  group_by(names) |>
  mutate(
    IQR = IQR(Luc_Ren, na.rm = TRUE),
    Outlier_upper = quantile(Luc_Ren, probs = c(.75), na.rm = TRUE) + 1.5 * IQR,
    Outlier_lower = quantile(Luc_Ren, probs = c(.25), na.rm = TRUE) - 1.5 * IQR,
    Luc_Ren_wo_outliers = if_else(Luc_Ren <= Outlier_lower | Luc_Ren >= Outlier_upper, NA, Luc_Ren),
        )

boxplot(Luc_Ren_wo_outliers ~ names, dataframe3)
```

## 2.7 Calculate the relative ratio response (RRR)

see:  
RRR = (well\_value - mean\_neg))/(mean\_pos-mean\_neg)

Relative Response ratio (Promega - Dual-Glo® Luciferase Assay System,
Instructions for use of Products E2920, E2940 and E2980)

https://www.promega.com/-/media/files/resources/protocols/technical-manuals/0/dual-glo-luciferase-assay-system-protocol.pdf

```
dataframe4 <- dataframe3 |>
  group_by(names) |>
  mutate(mean = mean(Luc_Ren_wo_outliers, na.rm = TRUE)) |>
  ungroup() |>
  mutate(
    mean_neg = min(mean),
    mean_pos = max(mean),
    RRR = (Luc_Ren_wo_outliers - mean_neg) / (mean_pos - mean_neg),
    RRRp = RRR * 100,
    block = "a") |>
  mutate_if(is.character, as.factor)
```

mean\_pos = mean of the positive reference

mean\_neg = mean of the negative reference

RRR = Relative Response Ratio

RRRp = Relative Response Ratio in percent

block = necessary for the Approximative Two-Sample Fisher-Pitman
Permutation Test, it allows the stratification (it has to be a
factor)

```
ggplot(dataframe4,
       aes(
         x = factor(names, level = level_order),
         y = RRRp,
         colour = reporter,
         group = names
       )) +
  geom_boxplot() +
  geom_jitter(width = 0.2) +
  theme(axis.text.x = element_text(
    vjust = 1,
    hjust = 1,
    size = 10,
    angle = 30
  )) + theme(
    panel.grid.major = element_line(colour = "gray85"),
    panel.grid.minor = element_line(colour = "gray90"),
    panel.background = element_rect(fill = NA)
  )  +
  ylim(-10,110)
```

```
## Warning: Removed 18 rows containing non-finite values (`stat_boxplot()`).
```

```
## Warning: Removed 18 rows containing missing values (`geom_point()`).
```

# 3 Statistical tests

## 3.1 Normality test

```
shapiro_test_result <- dataframe4 |> 
  group_by(names) |> 
  do(tidy(shapiro.test(.$RRRp)))

print(shapiro_test_result)
```

```
## # A tibble: 8 × 4
## # Groups:   names [8]
##   names           statistic p.value method                     
##   <fct>               <dbl>   <dbl> <chr>                      
## 1 delNR5A1            0.967  0.855  Shapiro-Wilk normality test
## 2 delNR5A1+NR5A1      0.896  0.142  Shapiro-Wilk normality test
## 3 Reference           0.971  0.905  Shapiro-Wilk normality test
## 4 Reference+NR5A1     0.925  0.399  Shapiro-Wilk normality test
## 5 Variant_1           0.850  0.0954 Shapiro-Wilk normality test
## 6 Variant_1+NR5A1     0.901  0.224  Shapiro-Wilk normality test
## 7 Variant_2           0.934  0.493  Shapiro-Wilk normality test
## 8 Variant_2+NR5A1     0.919  0.307  Shapiro-Wilk normality test
```

```
p_value <- min(shapiro_test_result$p.value)
p_value
```

```
## [1] 0.09539483
```

```
if (p_value < 0.05) {
  cat("The overall p-value obtained from the Bartlett test is less than 0.05, suggesting evidence supporting the rejection of the null hypothesis of homogeneity of variances.\n")
} else {
  cat("The overall p-value obtained from the Bartlett test is greater than or equal to 0.05, indicating insufficient evidence to reject the null hypothesis of homogeneity of variances.\n")
}
```

```
## The overall p-value obtained from the Bartlett test is greater than or equal to 0.05, indicating insufficient evidence to reject the null hypothesis of homogeneity of variances.
```

## 3.2 Homogeneity of variances

The conditions with or without the addition of the transcription
factor NR5A1 exhibited clearly distinct variances. As a result, a direct
comparison between these conditions is not informative.  
To assess the comparability of only conditions with NR5A1 added, both
Bartlett and Levene tests were conducted.

```
dataframe4b <- dataframe4 |>
  filter(factor1 == "NR5A1")

bartlett_test_result <- bartlett.test(RRRp ~ names, data = dataframe4b)
print(bartlett_test_result)
```

```
## 
##  Bartlett test of homogeneity of variances
## 
## data:  RRRp by names
## Bartlett's K-squared = 2.4295, df = 3, p-value = 0.4882
```

```
p_value <- bartlett_test_result$p.value
if (p_value < 0.05) {
  cat("The overall p-value obtained from the Bartlett test is less than 0.05, suggesting evidence supporting the rejection of the null hypothesis of homogeneity of variances.\n")
} else {
  cat("The overall p-value obtained from the Bartlett test is greater than or equal to 0.05, indicating insufficient evidence to reject the null hypothesis of homogeneity of variances.\n")
}
```

```
## The overall p-value obtained from the Bartlett test is greater than or equal to 0.05, indicating insufficient evidence to reject the null hypothesis of homogeneity of variances.
```

```
levene_test_result <- leveneTest(RRRp ~ names, data = dataframe4b)
print(levene_test_result)
```

```
## Levene's Test for Homogeneity of Variance (center = median)
##       Df F value Pr(>F)
## group  3  0.9606  0.421
##       39
```

```
p_value <- levene_test_result$`Pr(>F)`[1]
if (p_value < 0.05) {
  cat("The overall p-value obtained from the Levene test is less than 0.05, suggesting evidence supporting the rejection of the null hypothesis of homogeneity of variances.\n")
} else {
  cat("The overall p-value obtained from the Levene test is greater than or equal to 0.05, indicating insufficient evidence to reject the null hypothesis of homogeneity of variances.\n")
}
```

```
## The overall p-value obtained from the Levene test is greater than or equal to 0.05, indicating insufficient evidence to reject the null hypothesis of homogeneity of variances.
```

```
dataframe5 <- dataframe4[dataframe4$factor1 == "NR5A1", ]
dataframe5$Other <- unname(vapply(as.character(dataframe5$names), FUN = function(x)  if(x == "Reference+NR5A1") x else "Other", FUN.VALUE = ""))
ggplot(dataframe5,
       aes(
         x = factor(names, level = level_order),
         y = RRRp,
         colour = reporter,
         group = names
       )) +
  geom_boxplot() +
  geom_jitter(width = 0.2) +
  theme(axis.text.x = element_text(
    vjust = 1,
    hjust = 1,
    size = 10,
    angle = 30
  )) + theme(
    panel.grid.major = element_line(colour = "gray85"),
    panel.grid.minor = element_line(colour = "gray90"),
    panel.background = element_rect(fill = NA)
  ) +
  scale_y_continuous(labels = scientific) +
  xlab(NULL)
```

```
## Warning: Removed 5 rows containing non-finite values (`stat_boxplot()`).
```

```
## Warning: Removed 5 rows containing missing values (`geom_point()`).
```

## 3.3 Kruskal-Wallis rank sum test

```
library(dunn.test)
bb <- dunn.test(dataframe4$RRRp, g = dataframe4$names, kw = T, list = T, method = "bh")
```

```
##   Kruskal-Wallis rank sum test
## 
## data: x and group
## Kruskal-Wallis chi-squared = 77.4383, df = 7, p-value = 0
## 
## 
##                            Comparison of x by group                            
##                              (Benjamini-Hochberg)                              
## Col Mean-|
## Row Mean |   delNR5A1   delNR5A1   Referenc   Referenc   Variant_   Variant_
## ---------+------------------------------------------------------------------
## delNR5A1 |  -3.972807
##          |    0.0001*
##          |
## Referenc |  -1.624848   2.104570
##          |     0.0695     0.0309
##          |
## Referenc |  -6.759258  -3.024443  -4.838239
##          |    0.0000*    0.0029*    0.0000*
##          |
## Variant_ |  -0.691265   2.929527   0.841944   5.549015
##          |     0.2447    0.0037*     0.2073    0.0000*
##          |
## Variant_ |  -4.667717  -0.890126  -2.849288   2.043452  -3.622429
##          |    0.0000*     0.2091    0.0044*     0.0338    0.0004*
##          |
## Variant_ |  -2.595633   1.224336  -0.878838   4.067897  -1.713765   2.024444
##          |    0.0088*     0.1405     0.2043    0.0001*     0.0606     0.0334
##          |
## Variant_ |  -5.836005  -1.988718  -3.911672   1.063900  -4.664221  -1.027640
##          |    0.0000*     0.0344    0.0001*     0.1749    0.0000*     0.1774
## Col Mean-|
## Row Mean |   Variant_
## ---------+-----------
## Variant_ |  -3.099725
##          |    0.0025*
## 
## 
## List of pairwise comparisons: Z statistic (adjusted p-value)
## -------------------------------------------------------
## delNR5A1 - delNR5A1+NR5A1         : -3.972807 (0.0001)*
## delNR5A1 - Reference              : -1.624848 (0.0695)
## delNR5A1+NR5A1 - Reference        :  2.104570 (0.0309)
## delNR5A1 - Reference+NR5A1        : -6.759258 (0.0000)*
## delNR5A1+NR5A1 - Reference+NR5A1  : -3.024443 (0.0029)*
## Reference - Reference+NR5A1       : -4.838239 (0.0000)*
## delNR5A1 - Variant_1              : -0.691265 (0.2447)
## delNR5A1+NR5A1 - Variant_1        :  2.929527 (0.0037)*
## Reference - Variant_1             :  0.841944 (0.2073)
## Reference+NR5A1 - Variant_1       :  5.549015 (0.0000)*
## delNR5A1 - Variant_1+NR5A1        : -4.667717 (0.0000)*
## delNR5A1+NR5A1 - Variant_1+NR5A1  : -0.890126 (0.2091)
## Reference - Variant_1+NR5A1       : -2.849288 (0.0044)*
## Reference+NR5A1 - Variant_1+NR5A1 :  2.043452 (0.0338)
## Variant_1 - Variant_1+NR5A1       : -3.622429 (0.0004)*
## delNR5A1 - Variant_2              : -2.595633 (0.0088)*
## delNR5A1+NR5A1 - Variant_2        :  1.224336 (0.1405)
## Reference - Variant_2             : -0.878838 (0.2043)
## Reference+NR5A1 - Variant_2       :  4.067897 (0.0001)*
## Variant_1 - Variant_2             : -1.713765 (0.0606)
## Variant_1+NR5A1 - Variant_2       :  2.024444 (0.0334)
## delNR5A1 - Variant_2+NR5A1        : -5.836005 (0.0000)*
## delNR5A1+NR5A1 - Variant_2+NR5A1  : -1.988718 (0.0344)
## Reference - Variant_2+NR5A1       : -3.911672 (0.0001)*
## Reference+NR5A1 - Variant_2+NR5A1 :  1.063900 (0.1749)
## Variant_1 - Variant_2+NR5A1       : -4.664221 (0.0000)*
## Variant_1+NR5A1 - Variant_2+NR5A1 : -1.027640 (0.1774)
## Variant_2 - Variant_2+NR5A1       : -3.099725 (0.0025)*
## 
## alpha = 0.05
## Reject Ho if p <= alpha/2
```

## 3.4 Wilcoxon rank sum exact test

```
wilcox.test(RRRp~Other, data = dataframe5)
```

```
## 
##  Wilcoxon rank sum exact test
## 
## data:  RRRp by Other
## W = 2, p-value = 4.172e-09
## alternative hypothesis: true location shift is not equal to 0
```

# 4 End results

## 4.1 Plot

```
ggplot(dataframe4,
       aes(
         x = factor(names, level = level_order),
         y = RRRp,
         colour = reporter,
         group = names
       )) +
  geom_boxplot() +
  geom_jitter(width = 0.2) +
  theme(axis.text.x = element_text(
    vjust = 1,
    hjust = 1,
    size = 10,
    angle = 30
  )) + theme(
    panel.grid.major = element_line(colour = "gray85"),
    panel.grid.minor = element_line(colour = "gray90"),
    panel.background = element_rect(fill = NA)
  ) +
  scale_y_continuous(labels = scientific) +
  xlab(NULL)
```

```
## Warning: Removed 15 rows containing non-finite values (`stat_boxplot()`).
```

```
## Warning: Removed 15 rows containing missing values (`geom_point()`).
```

```
gt(dataframe5)
```

| names | reporter | factor1 | renilla | luciferase | IQR | Outlier\_upper | Outlier\_lower | renilla\_wo\_extremes | Luc\_Ren | Luc\_Ren\_wo\_outliers | mean | mean\_neg | mean\_pos | RRR | RRRp | block | Other |
| --- | --- | --- | --- | --- | --- | --- | --- | --- | --- | --- | --- | --- | --- | --- | --- | --- | --- |
| Reference+NR5A1 | Reference | NR5A1 | 473446.0 | 56730.90 | 0.02144612 | 0.17026626 | 0.08448180 | 473446.0 | 0.11982549 | 0.11982549 | 0.12747532 | 0.02903769 | 0.1274753 | 0.9222876 | 92.22876 | a | Reference+NR5A1 |
| Reference+NR5A1 | Reference | NR5A1 | 495631.0 | 55696.40 | 0.02144612 | 0.17026626 | 0.08448180 | 495631.0 | 0.11237473 | 0.11237473 | 0.12747532 | 0.02903769 | 0.1274753 | 0.8465974 | 84.65974 | a | Reference+NR5A1 |
| Reference+NR5A1 | Reference | NR5A1 | 484832.0 | 59950.50 | 0.02144612 | 0.17026626 | 0.08448180 | 484832.0 | 0.12365211 | 0.12365211 | 0.12747532 | 0.02903769 | 0.1274753 | 0.9611611 | 96.11611 | a | Reference+NR5A1 |
| Reference+NR5A1 | Reference | NR5A1 | 494818.0 | 57197.40 | 0.02144612 | 0.17026626 | 0.08448180 | 494818.0 | 0.11559280 | 0.11559280 | 0.12747532 | 0.02903769 | 0.1274753 | 0.8792889 | 87.92889 | a | Reference+NR5A1 |
| Reference+NR5A1 | Reference | NR5A1 | 593864.0 | 88169.90 | 0.02144612 | 0.17026626 | 0.08448180 | 593864.0 | 0.14846817 | 0.14846817 | 0.12747532 | 0.02903769 | 0.1274753 | 1.2132604 | 121.32604 | a | Reference+NR5A1 |
| Reference+NR5A1 | Reference | NR5A1 | 555759.0 | 70541.10 | 0.02144612 | 0.17026626 | 0.08448180 | 555759.0 | 0.12692750 | 0.12692750 | 0.12747532 | 0.02903769 | 0.1274753 | 0.9944349 | 99.44349 | a | Reference+NR5A1 |
| Reference+NR5A1 | Reference | NR5A1 | 646673.0 | 89618.90 | 0.02144612 | 0.17026626 | 0.08448180 | 646673.0 | 0.13858457 | 0.13858457 | 0.12747532 | 0.02903769 | 0.1274753 | 1.1128557 | 111.28557 | a | Reference+NR5A1 |
| Reference+NR5A1 | Reference | NR5A1 | 536634.0 | 73322.80 | 0.02144612 | 0.17026626 | 0.08448180 | 536634.0 | 0.13663465 | 0.13663465 | 0.12747532 | 0.02903769 | 0.1274753 | 1.0930471 | 109.30471 | a | Reference+NR5A1 |
| Reference+NR5A1 | Reference | NR5A1 | 592993.0 | 82763.70 | 0.02144612 | 0.17026626 | 0.08448180 | 592993.0 | 0.13956944 | 0.13956944 | 0.12747532 | 0.02903769 | 0.1274753 | 1.1228607 | 112.28607 | a | Reference+NR5A1 |
| Reference+NR5A1 | Reference | NR5A1 | 36610.9 | 4710.04 | 0.02144612 | 0.17026626 | 0.08448180 | NA | NA | NA | 0.12747532 | 0.02903769 | 0.1274753 | NA | NA | a | Reference+NR5A1 |
| Reference+NR5A1 | Reference | NR5A1 | 13402.4 | 1931.18 | 0.02144612 | 0.17026626 | 0.08448180 | NA | NA | NA | 0.12747532 | 0.02903769 | 0.1274753 | NA | NA | a | Reference+NR5A1 |
| Reference+NR5A1 | Reference | NR5A1 | 529804.0 | 59933.40 | 0.02144612 | 0.17026626 | 0.08448180 | 529804.0 | 0.11312372 | 0.11312372 | 0.12747532 | 0.02903769 | 0.1274753 | 0.8542062 | 85.42062 | a | Reference+NR5A1 |
| Variant\_1+NR5A1 | Variant\_1 | NR5A1 | 563558.0 | 45198.80 | 0.01351059 | 0.11066317 | 0.05662083 | 563558.0 | 0.08020257 | 0.08020257 | 0.08342983 | 0.02903769 | 0.1274753 | 0.5197695 | 51.97695 | a | Other |
| Variant\_1+NR5A1 | Variant\_1 | NR5A1 | 490290.0 | 38547.70 | 0.01351059 | 0.11066317 | 0.05662083 | 490290.0 | 0.07862224 | 0.07862224 | 0.08342983 | 0.02903769 | 0.1274753 | 0.5037155 | 50.37155 | a | Other |
| Variant\_1+NR5A1 | Variant\_1 | NR5A1 | 523060.0 | 41822.10 | 0.01351059 | 0.11066317 | 0.05662083 | 523060.0 | 0.07995660 | 0.07995660 | 0.08342983 | 0.02903769 | 0.1274753 | 0.5172708 | 51.72708 | a | Other |
| Variant\_1+NR5A1 | Variant\_1 | NR5A1 | 587296.0 | 42741.70 | 0.01351059 | 0.11066317 | 0.05662083 | 587296.0 | 0.07277710 | 0.07277710 | 0.08342983 | 0.02903769 | 0.1274753 | 0.4443363 | 44.43363 | a | Other |
| Variant\_1+NR5A1 | Variant\_1 | NR5A1 | 683813.0 | 50140.90 | 0.01351059 | 0.11066317 | 0.05662083 | 683813.0 | 0.07332546 | 0.07332546 | 0.08342983 | 0.02903769 | 0.1274753 | 0.4499069 | 44.99069 | a | Other |
| Variant\_1+NR5A1 | Variant\_1 | NR5A1 | 584700.0 | 44617.40 | 0.01351059 | 0.11066317 | 0.05662083 | 584700.0 | 0.07630819 | 0.07630819 | 0.08342983 | 0.02903769 | 0.1274753 | 0.4802077 | 48.02077 | a | Other |
| Variant\_1+NR5A1 | Variant\_1 | NR5A1 | 657799.0 | 62920.50 | 0.01351059 | 0.11066317 | 0.05662083 | 657799.0 | 0.09565308 | 0.09565308 | 0.08342983 | 0.02903769 | 0.1274753 | 0.6767269 | 67.67269 | a | Other |
| Variant\_1+NR5A1 | Variant\_1 | NR5A1 | 714926.0 | 64911.40 | 0.01351059 | 0.11066317 | 0.05662083 | 714926.0 | 0.09079457 | 0.09079457 | 0.08342983 | 0.02903769 | 0.1274753 | 0.6273707 | 62.73707 | a | Other |
| Variant\_1+NR5A1 | Variant\_1 | NR5A1 | 692377.0 | 61763.80 | 0.01351059 | 0.11066317 | 0.05662083 | 692377.0 | 0.08920545 | 0.08920545 | 0.08342983 | 0.02903769 | 0.1274753 | 0.6112272 | 61.12272 | a | Other |
| Variant\_1+NR5A1 | Variant\_1 | NR5A1 | 35828.2 | 3294.51 | 0.01351059 | 0.11066317 | 0.05662083 | NA | NA | NA | 0.08342983 | 0.02903769 | 0.1274753 | NA | NA | a | Other |
| Variant\_1+NR5A1 | Variant\_1 | NR5A1 | 15697.6 | 1393.09 | 0.01351059 | 0.11066317 | 0.05662083 | NA | NA | NA | 0.08342983 | 0.02903769 | 0.1274753 | NA | NA | a | Other |
| Variant\_1+NR5A1 | Variant\_1 | NR5A1 | 659306.0 | 64251.40 | 0.01351059 | 0.11066317 | 0.05662083 | 659306.0 | 0.09745308 | 0.09745308 | 0.08342983 | 0.02903769 | 0.1274753 | 0.6950126 | 69.50126 | a | Other |
| Variant\_2+NR5A1 | Variant\_2 | NR5A1 | 519301.0 | 53246.90 | 0.01313349 | 0.12827432 | 0.07574037 | 519301.0 | 0.10253572 | 0.10253572 | 0.10235920 | 0.02903769 | 0.1274753 | 0.7466457 | 74.66457 | a | Other |
| Variant\_2+NR5A1 | Variant\_2 | NR5A1 | 529884.0 | 51343.60 | 0.01313349 | 0.12827432 | 0.07574037 | 529884.0 | 0.09689592 | 0.09689592 | 0.10235920 | 0.02903769 | 0.1274753 | 0.6893526 | 68.93526 | a | Other |
| Variant\_2+NR5A1 | Variant\_2 | NR5A1 | 525803.0 | 45468.00 | 0.01313349 | 0.12827432 | 0.07574037 | 525803.0 | 0.08647345 | 0.08647345 | 0.10235920 | 0.02903769 | 0.1274753 | 0.5834737 | 58.34737 | a | Other |
| Variant\_2+NR5A1 | Variant\_2 | NR5A1 | 498938.0 | 45739.10 | 0.01313349 | 0.12827432 | 0.07574037 | 498938.0 | 0.09167291 | 0.09167291 | 0.10235920 | 0.02903769 | 0.1274753 | 0.6362935 | 63.62935 | a | Other |
| Variant\_2+NR5A1 | Variant\_2 | NR5A1 | 31775.4 | 2986.42 | 0.01313349 | 0.12827432 | 0.07574037 | 31775.4 | 0.09398528 | 0.09398528 | 0.10235920 | 0.02903769 | 0.1274753 | 0.6597842 | 65.97842 | a | Other |
| Variant\_2+NR5A1 | Variant\_2 | NR5A1 | 586286.0 | 62682.10 | 0.01313349 | 0.12827432 | 0.07574037 | 586286.0 | 0.10691386 | 0.10691386 | 0.10235920 | 0.02903769 | 0.1274753 | 0.7911220 | 79.11220 | a | Other |
| Variant\_2+NR5A1 | Variant\_2 | NR5A1 | 671262.0 | 72998.60 | 0.01313349 | 0.12827432 | 0.07574037 | 671262.0 | 0.10874830 | 0.10874830 | 0.10235920 | 0.02903769 | 0.1274753 | 0.8097575 | 80.97575 | a | Other |
| Variant\_2+NR5A1 | Variant\_2 | NR5A1 | 634343.0 | 70233.10 | 0.01313349 | 0.12827432 | 0.07574037 | 634343.0 | 0.11071786 | 0.11071786 | 0.10235920 | 0.02903769 | 0.1274753 | 0.8297657 | 82.97657 | a | Other |
| Variant\_2+NR5A1 | Variant\_2 | NR5A1 | 679095.0 | 72270.70 | 0.01313349 | 0.12827432 | 0.07574037 | 679095.0 | 0.10642208 | 0.10642208 | 0.10235920 | 0.02903769 | 0.1274753 | 0.7861261 | 78.61261 | a | Other |
| Variant\_2+NR5A1 | Variant\_2 | NR5A1 | 40432.7 | 4382.90 | 0.01313349 | 0.12827432 | 0.07574037 | 40432.7 | 0.10839988 | 0.10839988 | 0.10235920 | 0.02903769 | 0.1274753 | 0.8062181 | 80.62181 | a | Other |
| Variant\_2+NR5A1 | Variant\_2 | NR5A1 | 15395.1 | 1706.14 | 0.01313349 | 0.12827432 | 0.07574037 | NA | NA | NA | 0.10235920 | 0.02903769 | 0.1274753 | NA | NA | a | Other |
| Variant\_2+NR5A1 | Variant\_2 | NR5A1 | 618741.0 | 70032.80 | 0.01313349 | 0.12827432 | 0.07574037 | 618741.0 | 0.11318597 | 0.11318597 | 0.10235920 | 0.02903769 | 0.1274753 | 0.8548386 | 85.48386 | a | Other |
| delNR5A1+NR5A1 | delNR5A1 | NR5A1 | 492362.0 | 35852.30 | 0.01152458 | 0.09252188 | 0.04642354 | 492362.0 | 0.07281695 | 0.07281695 | 0.06956795 | 0.02903769 | 0.1274753 | 0.4447411 | 44.47411 | a | Other |
| delNR5A1+NR5A1 | delNR5A1 | NR5A1 | 589801.0 | 35955.70 | 0.01152458 | 0.09252188 | 0.04642354 | 589801.0 | 0.06096243 | 0.06096243 | 0.06956795 | 0.02903769 | 0.1274753 | 0.3243144 | 32.43144 | a | Other |
| delNR5A1+NR5A1 | delNR5A1 | NR5A1 | 583858.0 | 38397.20 | 0.01152458 | 0.09252188 | 0.04642354 | 583858.0 | 0.06576462 | 0.06576462 | 0.06956795 | 0.02903769 | 0.1274753 | 0.3730985 | 37.30985 | a | Other |
| delNR5A1+NR5A1 | delNR5A1 | NR5A1 | 631868.0 | 34042.40 | 0.01152458 | 0.09252188 | 0.04642354 | 631868.0 | 0.05387581 | 0.05387581 | 0.06956795 | 0.02903769 | 0.1274753 | 0.2523235 | 25.23235 | a | Other |
| delNR5A1+NR5A1 | delNR5A1 | NR5A1 | 72923.1 | 4469.94 | 0.01152458 | 0.09252188 | 0.04642354 | 72923.1 | 0.06129663 | 0.06129663 | 0.06956795 | 0.02903769 | 0.1274753 | 0.3277094 | 32.77094 | a | Other |
| delNR5A1+NR5A1 | delNR5A1 | NR5A1 | 571305.0 | 42973.60 | 0.01152458 | 0.09252188 | 0.04642354 | 571305.0 | 0.07522007 | 0.07522007 | 0.06956795 | 0.02903769 | 0.1274753 | 0.4691537 | 46.91537 | a | Other |
| delNR5A1+NR5A1 | delNR5A1 | NR5A1 | 735373.0 | 47442.60 | 0.01152458 | 0.09252188 | 0.04642354 | 735373.0 | 0.06451501 | 0.06451501 | 0.06956795 | 0.02903769 | 0.1274753 | 0.3604041 | 36.04041 | a | Other |
| delNR5A1+NR5A1 | delNR5A1 | NR5A1 | 649126.0 | 51043.20 | 0.01152458 | 0.09252188 | 0.04642354 | 649126.0 | 0.07863373 | 0.07863373 | 0.06956795 | 0.02903769 | 0.1274753 | 0.5038322 | 50.38322 | a | Other |
| delNR5A1+NR5A1 | delNR5A1 | NR5A1 | 672333.0 | 50613.10 | 0.01152458 | 0.09252188 | 0.04642354 | 672333.0 | 0.07527981 | 0.07527981 | 0.06956795 | 0.02903769 | 0.1274753 | 0.4697606 | 46.97606 | a | Other |
| delNR5A1+NR5A1 | delNR5A1 | NR5A1 | 46740.5 | 3449.56 | 0.01152458 | 0.09252188 | 0.04642354 | 46740.5 | 0.07380238 | 0.07380238 | 0.06956795 | 0.02903769 | 0.1274753 | 0.4547518 | 45.47518 | a | Other |
| delNR5A1+NR5A1 | delNR5A1 | NR5A1 | 14423.8 | 1075.05 | 0.01152458 | 0.09252188 | 0.04642354 | 14423.8 | 0.07453306 | 0.07453306 | 0.06956795 | 0.02903769 | 0.1274753 | 0.4621746 | 46.21746 | a | Other |
| delNR5A1+NR5A1 | delNR5A1 | NR5A1 | 633089.0 | 49453.70 | 0.01152458 | 0.09252188 | 0.04642354 | 633089.0 | 0.07811493 | 0.07811493 | 0.06956795 | 0.02903769 | 0.1274753 | 0.4985618 | 49.85618 | a | Other |

```
library(gtsummary)
dataframe4 |> tbl_summary(
  type = all_continuous() ~ "continuous2",
  include = c(names, RRRp),
  by = names,
  statistic = all_continuous() ~ c("{median}","{mean}", "{sd}", "{min}", "{max}"),
  digits = list(
              all_categorical() ~c(0,1),
              all_continuous() ~ c(1,1)),
  missing_text = "NA"
  )|>
  bold_labels() |>
  italicize_levels()
```

| **Characteristic** | **delNR5A1**, N = 12 | **delNR5A1+NR5A1**, N = 12 | **Reference**, N = 12 | **Reference+NR5A1**, N = 12 | **Variant\_1**, N = 12 | **Variant\_1+NR5A1**, N = 12 | **Variant\_2**, N = 12 | **Variant\_2+NR5A1**, N = 12 |
| --- | --- | --- | --- | --- | --- | --- | --- | --- |
| RRRp |  |  |  |  |  |  |  |  |
| Median | -0.1 | 45.0 | 10.6 | 97.8 | 3.1 | 51.9 | 18.7 | 78.6 |
| Mean | 0.0 | 41.2 | 10.7 | 100.0 | 2.9 | 55.3 | 19.2 | 74.5 |
| SD | 2.2 | 8.1 | 1.3 | 12.9 | 0.9 | 9.2 | 3.0 | 8.9 |
| Minimum | -4.1 | 25.2 | 8.7 | 84.7 | 1.5 | 44.4 | 15.4 | 58.3 |
| Maximum | 3.8 | 50.4 | 12.9 | 121.3 | 3.9 | 69.5 | 23.9 | 85.5 |
| NA | 1 | 0 | 3 | 2 | 4 | 2 | 2 | 1 |

```
sessionInfo()
```

```
## R version 4.3.2 (2023-10-31)
## Platform: aarch64-apple-darwin20 (64-bit)
## Running under: macOS Sonoma 14.3.1
## 
## Matrix products: default
## BLAS:   /Library/Frameworks/R.framework/Versions/4.3-arm64/Resources/lib/libRblas.0.dylib 
## LAPACK: /Library/Frameworks/R.framework/Versions/4.3-arm64/Resources/lib/libRlapack.dylib;  LAPACK version 3.11.0
## 
## locale:
## [1] en_US.UTF-8/en_US.UTF-8/en_US.UTF-8/C/en_US.UTF-8/en_US.UTF-8
## 
## time zone: Europe/Paris
## tzcode source: internal
## 
## attached base packages:
## [1] stats     graphics  grDevices utils     datasets  methods   base     
## 
## other attached packages:
##  [1] dunn.test_1.3.5 gt_0.10.1       gtsummary_1.7.2 car_3.1-2      
##  [5] carData_3.0-5   broom_1.0.5     ggpubr_0.6.0    coin_1.4-3     
##  [9] survival_3.5-7  scales_1.3.0    lubridate_1.9.3 forcats_1.0.0  
## [13] stringr_1.5.1   dplyr_1.1.4     purrr_1.0.2     readr_2.1.5    
## [17] tidyr_1.3.0     tibble_3.2.1    ggplot2_3.4.4   tidyverse_2.0.0
## 
## loaded via a namespace (and not attached):
##  [1] gtable_0.3.4         xfun_0.41            bslib_0.6.1         
##  [4] rstatix_0.7.2        lattice_0.22-5       tzdb_0.4.0          
##  [7] vctrs_0.6.5          tools_4.3.2          generics_0.1.3      
## [10] stats4_4.3.2         parallel_4.3.2       sandwich_3.1-0      
## [13] fansi_1.0.6          highr_0.10           pkgconfig_2.0.3     
## [16] Matrix_1.6-5         lifecycle_1.0.4      farver_2.1.1        
## [19] compiler_4.3.2       munsell_0.5.0        codetools_0.2-19    
## [22] htmltools_0.5.7      sass_0.4.8           yaml_2.3.8          
## [25] pillar_1.9.0         jquerylib_0.1.4      broom.helpers_1.14.0
## [28] MASS_7.3-60.0.1      cachem_1.0.8         abind_1.4-5         
## [31] multcomp_1.4-25      commonmark_1.9.0     tidyselect_1.2.0    
## [34] digest_0.6.34        mvtnorm_1.2-4        stringi_1.8.3       
## [37] labeling_0.4.3       splines_4.3.2        fastmap_1.1.1       
## [40] grid_4.3.2           colorspace_2.1-0     cli_3.6.2           
## [43] magrittr_2.0.3       utf8_1.2.4           TH.data_1.1-2       
## [46] libcoin_1.0-10       withr_3.0.0          backports_1.4.1     
## [49] timechange_0.3.0     rmarkdown_2.25       matrixStats_1.2.0   
## [52] ggsignif_0.6.4       zoo_1.8-12           modeltools_0.2-23   
## [55] hms_1.1.3            evaluate_0.23        knitr_1.45          
## [58] markdown_1.12        rlang_1.1.3          glue_1.7.0          
## [61] xml2_1.3.6           rstudioapi_0.15.0    jsonlite_1.8.8      
## [64] R6_2.5.1
```
