# Supplementary material for: A conserved NR5A1-responsive enhancer regulates SRY in testis-determination
Source: Nat Commun. 2024 Mar 30;15:2796. doi: 10.1038/s41467-024-47162-2 (PMC10981742; doi:10.1038/s41467-024-47162-2)
Supplement: Supplementary file 21 — Supplementary Dataset 18 [file 41467_2024_47162_MOESM21_ESM.html]

LMM\_SRY


Code 

- Show All Code
- Hide All Code

# LMM\_SRY

#### Vincent Laville

#### 2023-12-01

## Data

```
data <- read.table("data_pcr_sry.txt", header = T, sep = "\t", dec = ".", fill = T)
data <- na.omit(data)
data$genotype <- factor(data$genotype, levels = c("WT", "Mut"))
data$Time <- factor(data$Time, 
                    levels = c("iPS", "M1_36h00", "M2_06h00", "M2_12h00", "M2_24h00", "M2_36h00", "M2_48h00", "M3_24h00", "M3_48h00"))
```

```
data %>%
  kbl() %>%
  kable_paper("hover", full_width = F) %>%
  kable_styling(bootstrap_options = c("striped", "hover")) %>%
  scroll_box(height = "300px")
```

|  | Sample\_Reference | genotype | dCt\_NA | Experiment | Time |
| --- | --- | --- | --- | --- | --- |
| 1 | 21\_85 | WT | 14.596634 | iPS07 | M1\_36h00 |
| 2 | 21\_86 | WT | 14.803952 | iPS07 | M1\_36h00 |
| 3 | 21\_92 | Mut | 16.311497 | iPS07 | M1\_36h00 |
| 4 | 21\_93 | Mut | 16.426530 | iPS07 | M1\_36h00 |
| 5 | 21\_94 | Mut | 16.089224 | iPS07 | M1\_36h00 |
| 6 | 21\_95 | Mut | 15.441164 | iPS07 | M1\_36h00 |
| 7 | 21\_96 | Mut | 15.629291 | iPS07 | M1\_36h00 |
| 8 | 21\_121 | WT | 11.567433 | iPS09 | M1\_36h00 |
| 9 | 21\_122 | WT | 11.983450 | iPS09 | M1\_36h00 |
| 10 | 21\_123 | WT | 12.692663 | iPS09 | M1\_36h00 |
| 11 | 21\_124 | WT | 12.183833 | iPS09 | M1\_36h00 |
| 12 | 21\_125 | WT | 11.365223 | iPS09 | M1\_36h00 |
| 14 | 21\_127 | Mut | 12.557431 | iPS09 | M1\_36h00 |
| 16 | 21\_129 | Mut | 12.604310 | iPS09 | M1\_36h00 |
| 17 | 21\_130 | Mut | 12.560797 | iPS09 | M1\_36h00 |
| 18 | 22\_60 | WT | 12.469458 | iPS12 | M1\_36h00 |
| 19 | 22\_61 | WT | 11.886395 | iPS12 | M1\_36h00 |
| 20 | 22\_62 | WT | 12.458997 | iPS12 | M1\_36h00 |
| 21 | 22\_63 | WT | 12.269345 | iPS12 | M1\_36h00 |
| 22 | 22\_64 | WT | 12.063809 | iPS12 | M1\_36h00 |
| 23 | 22\_65 | Mut | 13.099315 | iPS12 | M1\_36h00 |
| 24 | 22\_66 | Mut | 12.986908 | iPS12 | M1\_36h00 |
| 25 | 22\_67 | Mut | 13.427801 | iPS12 | M1\_36h00 |
| 26 | 22\_68 | Mut | 13.286646 | iPS12 | M1\_36h00 |
| 27 | 22\_69 | Mut | 13.250400 | iPS12 | M1\_36h00 |
| 28 | 22\_70 | Mut | 13.257294 | iPS12 | M1\_36h00 |
| 29 | 23\_181 | WT | 12.029183 | iPS26a | M1\_36h00 |
| 30 | 23\_182 | WT | 12.434442 | iPS26a | M1\_36h00 |
| 31 | 23\_183 | WT | 12.430442 | iPS26a | M1\_36h00 |
| 32 | 23\_184 | WT | 12.386509 | iPS26a | M1\_36h00 |
| 33 | 23\_185 | WT | 12.050018 | iPS26a | M1\_36h00 |
| 34 | 23\_186 | WT | 12.461838 | iPS26a | M1\_36h00 |
| 35 | 23\_187 | WT | 11.419987 | iPS26b | M1\_36h00 |
| 36 | 23\_188 | WT | 11.680879 | iPS26b | M1\_36h00 |
| 37 | 23\_189 | WT | 11.641933 | iPS26b | M1\_36h00 |
| 38 | 23\_190 | WT | 11.775509 | iPS26b | M1\_36h00 |
| 39 | 23\_191 | WT | 11.477846 | iPS26b | M1\_36h00 |
| 40 | 23\_192 | WT | 11.563967 | iPS26b | M1\_36h00 |
| 41 | 23\_193 | Mut | 13.096592 | iPS26a | M1\_36h00 |
| 42 | 23\_194 | Mut | 12.511234 | iPS26a | M1\_36h00 |
| 43 | 23\_195 | Mut | 13.157060 | iPS26a | M1\_36h00 |
| 44 | 23\_196 | Mut | 12.914510 | iPS26a | M1\_36h00 |
| 45 | 23\_197 | Mut | 12.795907 | iPS26a | M1\_36h00 |
| 46 | 23\_198 | Mut | 13.135006 | iPS26a | M1\_36h00 |
| 47 | 23\_199 | Mut | 13.566621 | iPS26b | M1\_36h00 |
| 48 | 23\_200 | Mut | 13.385669 | iPS26b | M1\_36h00 |
| 49 | 23\_201 | Mut | 13.106202 | iPS26b | M1\_36h00 |
| 50 | 23\_202 | Mut | 13.481652 | iPS26b | M1\_36h00 |
| 51 | 23\_203 | Mut | 13.157958 | iPS26b | M1\_36h00 |
| 52 | 23\_204 | Mut | 13.405045 | iPS26b | M1\_36h00 |
| 53 | 22\_71 | WT | 8.005868 | iPS12 | M2\_06h00 |
| 54 | 22\_72 | WT | 8.285998 | iPS12 | M2\_06h00 |
| 55 | 22\_73 | WT | 8.231141 | iPS12 | M2\_06h00 |
| 56 | 22\_74 | WT | 8.474505 | iPS12 | M2\_06h00 |
| 57 | 22\_75 | WT | 7.892482 | iPS12 | M2\_06h00 |
| 60 | 22\_78 | Mut | 9.195338 | iPS12 | M2\_06h00 |
| 61 | 22\_79 | Mut | 9.167594 | iPS12 | M2\_06h00 |
| 62 | 22\_80 | Mut | 8.839355 | iPS12 | M2\_06h00 |
| 63 | 22\_81 | Mut | 9.085207 | iPS12 | M2\_06h00 |
| 64 | 22\_82 | Mut | 8.584562 | iPS12 | M2\_06h00 |
| 65 | 23\_205 | WT | 11.027844 | iPS26a | M2\_06h00 |
| 66 | 23\_206 | WT | 10.831761 | iPS26a | M2\_06h00 |
| 67 | 23\_207 | WT | 10.951078 | iPS26a | M2\_06h00 |
| 68 | 23\_208 | WT | 10.727536 | iPS26b | M2\_06h00 |
| 69 | 23\_209 | WT | 10.719599 | iPS26b | M2\_06h00 |
| 70 | 23\_210 | WT | 10.850361 | iPS26b | M2\_06h00 |
| 71 | 23\_211 | Mut | 11.816408 | iPS26a | M2\_06h00 |
| 72 | 23\_212 | Mut | 11.631556 | iPS26a | M2\_06h00 |
| 73 | 23\_213 | Mut | 11.504724 | iPS26a | M2\_06h00 |
| 74 | 23\_214 | Mut | 11.924516 | iPS26b | M2\_06h00 |
| 75 | 23\_215 | Mut | 11.463224 | iPS26b | M2\_06h00 |
| 76 | 23\_216 | Mut | 11.842700 | iPS26b | M2\_06h00 |
| 78 | 22\_84 | WT | 10.340000 | iPS12 | M2\_12h00 |
| 79 | 22\_85 | WT | 10.600000 | iPS12 | M2\_12h00 |
| 80 | 22\_86 | WT | 10.360000 | iPS12 | M2\_12h00 |
| 81 | 22\_87 | WT | 10.350000 | iPS12 | M2\_12h00 |
| 82 | 22\_88 | WT | 10.130000 | iPS12 | M2\_12h00 |
| 83 | 22\_89 | Mut | 9.910000 | iPS12 | M2\_12h00 |
| 84 | 22\_90 | Mut | 10.250000 | iPS12 | M2\_12h00 |
| 85 | 22\_91 | Mut | 9.670000 | iPS12 | M2\_12h00 |
| 86 | 22\_92 | Mut | 9.810000 | iPS12 | M2\_12h00 |
| 87 | 22\_93 | Mut | 10.320000 | iPS12 | M2\_12h00 |
| 88 | 22\_94 | Mut | 9.890000 | iPS12 | M2\_12h00 |
| 89 | 23\_217 | WT | 9.814057 | iPS26a | M2\_12h00 |
| 90 | 23\_218 | WT | 9.932432 | iPS26a | M2\_12h00 |
| 91 | 23\_219 | WT | 9.870649 | iPS26a | M2\_12h00 |
| 92 | 23\_220 | WT | 9.833612 | iPS26b | M2\_12h00 |
| 93 | 23\_221 | WT | 9.910166 | iPS26b | M2\_12h00 |
| 94 | 23\_222 | WT | 9.823281 | iPS26b | M2\_12h00 |
| 95 | 23\_223 | Mut | 10.770520 | iPS26a | M2\_12h00 |
| 96 | 23\_224 | Mut | 10.251056 | iPS26a | M2\_12h00 |
| 97 | 23\_225 | Mut | 10.892659 | iPS26a | M2\_12h00 |
| 98 | 23\_226 | Mut | 10.372483 | iPS26b | M2\_12h00 |
| 99 | 23\_227 | Mut | 10.278356 | iPS26b | M2\_12h00 |
| 100 | 23\_228 | Mut | 10.594420 | iPS26b | M2\_12h00 |
| 101 | 21\_131 | WT | 9.219157 | iPS09 | M2\_24h00 |
| 102 | 21\_132 | WT | 8.908860 | iPS09 | M2\_24h00 |
| 103 | 21\_133 | WT | 9.157994 | iPS09 | M2\_24h00 |
| 104 | 21\_134 | WT | 9.072147 | iPS09 | M2\_24h00 |
| 105 | 21\_135 | WT | 8.965787 | iPS09 | M2\_24h00 |
| 106 | 21\_136 | Mut | 8.823237 | iPS09 | M2\_24h00 |
| 108 | 21\_138 | Mut | 8.850614 | iPS09 | M2\_24h00 |
| 109 | 21\_139 | Mut | 8.848917 | iPS09 | M2\_24h00 |
| 111 | 22\_95 | WT | 9.500000 | iPS12 | M2\_24h00 |
| 112 | 22\_96 | WT | 9.760000 | iPS12 | M2\_24h00 |
| 113 | 22\_97 | WT | 9.500000 | iPS12 | M2\_24h00 |
| 114 | 22\_98 | WT | 9.340000 | iPS12 | M2\_24h00 |
| 115 | 22\_99 | WT | 9.570000 | iPS12 | M2\_24h00 |
| 116 | 22\_100 | WT | 9.090000 | iPS12 | M2\_24h00 |
| 117 | 22\_101 | Mut | 9.080000 | iPS12 | M2\_24h00 |
| 118 | 22\_102 | Mut | 9.130000 | iPS12 | M2\_24h00 |
| 119 | 22\_103 | Mut | 9.310000 | iPS12 | M2\_24h00 |
| 120 | 22\_104 | Mut | 8.850000 | iPS12 | M2\_24h00 |
| 121 | 22\_105 | Mut | 9.120000 | iPS12 | M2\_24h00 |
| 122 | 22\_106 | Mut | 8.830000 | iPS12 | M2\_24h00 |
| 123 | 23\_229 | WT | 8.381206 | iPS26a | M2\_36h00 |
| 124 | 23\_230 | WT | 8.215758 | iPS26a | M2\_36h00 |
| 125 | 23\_231 | WT | 8.471165 | iPS26a | M2\_36h00 |
| 126 | 23\_232 | WT | 8.737809 | iPS26b | M2\_36h00 |
| 127 | 23\_233 | WT | 8.748088 | iPS26b | M2\_36h00 |
| 128 | 23\_234 | WT | 8.853533 | iPS26b | M2\_36h00 |
| 129 | 23\_235 | Mut | 9.870363 | iPS26a | M2\_36h00 |
| 130 | 23\_236 | Mut | 9.729233 | iPS26a | M2\_36h00 |
| 131 | 23\_237 | Mut | 10.108756 | iPS26a | M2\_36h00 |
| 132 | 23\_238 | Mut | 8.830957 | iPS26b | M2\_36h00 |
| 133 | 23\_239 | Mut | 8.857772 | iPS26b | M2\_36h00 |
| 134 | 23\_240 | Mut | 8.999436 | iPS26b | M2\_36h00 |
| 135 | 21\_87 | WT | 13.383926 | iPS07 | M2\_48h00 |
| 136 | 21\_88 | WT | 14.098783 | iPS07 | M2\_48h00 |
| 137 | 21\_89 | WT | 13.367328 | iPS07 | M2\_48h00 |
| 138 | 21\_90 | WT | 13.598455 | iPS07 | M2\_48h00 |
| 139 | 21\_91 | WT | 13.820448 | iPS07 | M2\_48h00 |
| 140 | 21\_97 | Mut | 13.482145 | iPS07 | M2\_48h00 |
| 141 | 21\_98 | Mut | 13.525383 | iPS07 | M2\_48h00 |
| 142 | 21\_99 | Mut | 13.461279 | iPS07 | M2\_48h00 |
| 144 | 21\_101 | Mut | 13.532344 | iPS07 | M2\_48h00 |
| 145 | 22\_107 | WT | 10.599298 | iPS12 | M2\_48h00 |
| 146 | 22\_108 | WT | 10.866567 | iPS12 | M2\_48h00 |
| 147 | 22\_109 | WT | 10.637803 | iPS12 | M2\_48h00 |
| 148 | 22\_110 | WT | 10.907965 | iPS12 | M2\_48h00 |
| 149 | 22\_111 | WT | 10.961612 | iPS12 | M2\_48h00 |
| 151 | 22\_113 | Mut | 10.732405 | iPS12 | M2\_48h00 |
| 152 | 22\_114 | Mut | 11.229876 | iPS12 | M2\_48h00 |
| 153 | 22\_115 | Mut | 11.136715 | iPS12 | M2\_48h00 |
| 154 | 22\_116 | Mut | 10.804056 | iPS12 | M2\_48h00 |
| 155 | 22\_117 | Mut | 10.861813 | iPS12 | M2\_48h00 |
| 156 | 22\_118 | Mut | 10.848863 | iPS12 | M2\_48h00 |
| 157 | 23\_241 | WT | 8.735721 | iPS26a | M2\_48h00 |
| 158 | 23\_242 | WT | 8.900798 | iPS26a | M2\_48h00 |
| 159 | 23\_243 | WT | 8.976192 | iPS26a | M2\_48h00 |
| 160 | 23\_244 | WT | 8.979135 | iPS26b | M2\_48h00 |
| 161 | 23\_245 | WT | 8.983508 | iPS26b | M2\_48h00 |
| 162 | 23\_246 | WT | 9.221233 | iPS26b | M2\_48h00 |
| 163 | 23\_247 | Mut | 10.678058 | iPS26a | M2\_48h00 |
| 164 | 23\_248 | Mut | 10.811870 | iPS26a | M2\_48h00 |
| 165 | 23\_249 | Mut | 10.592746 | iPS26a | M2\_48h00 |
| 166 | 23\_250 | Mut | 9.965942 | iPS26b | M2\_48h00 |
| 167 | 23\_251 | Mut | 9.827739 | iPS26b | M2\_48h00 |
| 168 | 23\_252 | Mut | 9.839309 | iPS26b | M2\_48h00 |
| 169 | 23\_253 | WT | 10.014881 | iPS26a | M3\_24h00 |
| 170 | 23\_254 | WT | 10.014911 | iPS26a | M3\_24h00 |
| 171 | 23\_255 | WT | 9.972318 | iPS26a | M3\_24h00 |
| 172 | 23\_256 | WT | 9.424125 | iPS26b | M3\_24h00 |
| 173 | 23\_257 | WT | 9.308253 | iPS26b | M3\_24h00 |
| 174 | 23\_258 | WT | 9.679753 | iPS26b | M3\_24h00 |
| 175 | 23\_259 | Mut | 11.346791 | iPS26a | M3\_24h00 |
| 176 | 23\_260 | Mut | 11.153167 | iPS26a | M3\_24h00 |
| 177 | 23\_261 | Mut | 11.258534 | iPS26a | M3\_24h00 |
| 178 | 23\_262 | Mut | 11.993087 | iPS26b | M3\_24h00 |
| 179 | 23\_263 | Mut | 11.824392 | iPS26b | M3\_24h00 |
| 180 | 23\_264 | Mut | 12.080067 | iPS26b | M3\_24h00 |
| 181 | 21\_146 | WT | 13.509595 | iPS09 | M3\_48h00 |
| 182 | 21\_147 | WT | 13.194978 | iPS09 | M3\_48h00 |
| 183 | 21\_148 | WT | 14.252355 | iPS09 | M3\_48h00 |
| 184 | 21\_149 | WT | 14.454320 | iPS09 | M3\_48h00 |
| 185 | 21\_150 | WT | 13.709505 | iPS09 | M3\_48h00 |
| 186 | 21\_151 | Mut | 14.139384 | iPS09 | M3\_48h00 |
| 187 | 21\_152 | Mut | 13.557335 | iPS09 | M3\_48h00 |
| 188 | 21\_153 | Mut | 13.308678 | iPS09 | M3\_48h00 |
| 189 | 21\_154 | Mut | 14.139130 | iPS09 | M3\_48h00 |
| 190 | 21\_155 | Mut | 13.742890 | iPS09 | M3\_48h00 |
| 191 | 23\_265 | WT | 12.095206 | iPS26a | M3\_48h00 |
| 192 | 23\_266 | WT | 11.767037 | iPS26a | M3\_48h00 |
| 193 | 23\_267 | WT | 11.753289 | iPS26a | M3\_48h00 |
| 194 | 23\_268 | WT | 9.824379 | iPS26b | M3\_48h00 |
| 195 | 23\_269 | WT | 9.775112 | iPS26b | M3\_48h00 |
| 196 | 23\_270 | WT | 9.936977 | iPS26b | M3\_48h00 |
| 197 | 23\_271 | Mut | 11.299758 | iPS26a | M3\_48h00 |
| 198 | 23\_272 | Mut | 11.027251 | iPS26a | M3\_48h00 |
| 199 | 23\_273 | Mut | 11.021998 | iPS26a | M3\_48h00 |
| 200 | 23\_274 | Mut | 11.989670 | iPS26b | M3\_48h00 |
| 201 | 23\_275 | Mut | 12.324713 | iPS26b | M3\_48h00 |
| 202 | 23\_276 | Mut | 12.381289 | iPS26b | M3\_48h00 |
| 203 | 21\_107 | WT | 11.195033 | iPS07 | iPS |
| 204 | 21\_108 | WT | 12.530930 | iPS07 | iPS |
| 205 | 21\_109 | WT | 13.016473 | iPS07 | iPS |
| 206 | 21\_110 | WT | 12.288249 | iPS07 | iPS |
| 207 | 21\_111 | WT | 11.495680 | iPS07 | iPS |
| 208 | 21\_112 | WT | 12.322269 | iPS07 | iPS |
| 209 | 21\_113 | WT | 11.809245 | iPS07 | iPS |
| 210 | 21\_115 | Mut | 8.645469 | iPS09 | iPS |
| 211 | 21\_116 | Mut | 9.233728 | iPS09 | iPS |
| 212 | 21\_117 | Mut | 8.221293 | iPS09 | iPS |
| 213 | 21\_118 | Mut | 8.164405 | iPS09 | iPS |
| 214 | 21\_119 | Mut | 9.148843 | iPS09 | iPS |
| 215 | 21\_120 | Mut | 9.132396 | iPS09 | iPS |
| 216 | 23\_157 | WT | 10.019725 | iPS19 | iPS |
| 217 | 23\_158 | WT | 9.890490 | iPS19 | iPS |
| 218 | 23\_159 | WT | 10.114000 | iPS19 | iPS |
| 219 | 23\_160 | WT | 10.113975 | iPS19 | iPS |
| 220 | 23\_161 | WT | 9.815210 | iPS19 | iPS |
| 221 | 23\_162 | WT | 9.920915 | iPS19 | iPS |
| 222 | 23\_169 | WT | 8.794045 | iPS19 | iPS |
| 223 | 23\_170 | WT | 9.215239 | iPS19 | iPS |
| 224 | 23\_171 | WT | 9.283852 | iPS19 | iPS |
| 225 | 23\_172 | WT | 9.501702 | iPS19 | iPS |
| 226 | 23\_173 | WT | 9.517063 | iPS19 | iPS |
| 227 | 23\_174 | WT | 9.074023 | iPS19 | iPS |
| 228 | 23\_163 | Mut | 9.833785 | iPS19 | iPS |
| 229 | 23\_164 | Mut | 10.197085 | iPS19 | iPS |
| 230 | 23\_165 | Mut | 10.180220 | iPS19 | iPS |
| 231 | 23\_166 | Mut | 9.933255 | iPS19 | iPS |
| 232 | 23\_167 | Mut | 10.197280 | iPS19 | iPS |
| 233 | 23\_168 | Mut | 10.047490 | iPS19 | iPS |
| 234 | 23\_175 | Mut | 9.281657 | iPS19 | iPS |
| 235 | 23\_176 | Mut | 9.568093 | iPS19 | iPS |
| 236 | 23\_177 | Mut | 9.750725 | iPS19 | iPS |
| 237 | 23\_178 | Mut | 9.291428 | iPS19 | iPS |
| 238 | 23\_179 | Mut | 9.459742 | iPS19 | iPS |
| 239 | 23\_180 | Mut | 9.569765 | iPS19 | iPS |

# Plots

We first examine the distribution of `dCt_NA` across the
different timepoints and colored by experiments.

```
ggplot(data = data, aes(x = genotype, y = dCt_NA)) +
         geom_boxplot(outlier.shape = NA) +
         geom_jitter(aes(colour = Experiment), size = 0.8) +
         theme_classic() + facet_grid(. ~ Time)
```

Figure 1: dCt (raw data) as a function of the genotype at each time
point

We next look at the distribution of `dCt_NA` across the
different experiments and colored by timepoints.

```
ggplot(data = data, aes(x = genotype, y = dCt_NA, colour = Time)) +
         geom_boxplot(outlier.shape = NA) +
         geom_jitter(position=position_jitterdodge(jitter.width = 0.1), size = 0.8) +
         theme_classic() + facet_grid(. ~ Experiment)
```

Figure 2: dCt (raw data) as a function of the genotype in each
experiment

# Analysis

We use a mixed-effect model, to analyse the dependent variable
`dCt_NA` with respect to:

- fixed effects (i.e., the `genotype` and
  `Time` variables)
- random effects (i.e., the `Experiment`
  variable)

We include an interaction term between `genotype`and
`Timepoint` as we are interested in the effect of
`genotype`at each `Timepoint` and the effect of
`genotype`seems not to be homogeneous at each timepoint.

We obtain the following:

```
mod = lmer(dCt_NA ~ genotype*Time + (1 | Experiment), data = data)

summary(mod)
```

```
## Linear mixed model fit by REML. t-tests use Satterthwaite's method [
## lmerModLmerTest]
## Formula: dCt_NA ~ genotype * Time + (1 | Experiment)
##    Data: data
## 
## REML criterion at convergence: 542.1
## 
## Scaled residuals: 
##     Min      1Q  Median      3Q     Max 
## -2.8157 -0.4992  0.1088  0.5813  2.6207 
## 
## Random effects:
##  Groups     Name        Variance Std.Dev.
##  Experiment (Intercept) 1.6414   1.2812  
##  Residual               0.5545   0.7446  
## Number of obs: 230, groups:  Experiment, 6
## 
## Fixed effects:
##                           Estimate Std. Error        df t value Pr(>|t|)    
## (Intercept)                9.43486    0.56965   6.71332  16.563 1.08e-06 ***
## genotypeMut               -0.11411    0.25876 207.70085  -0.441 0.659685    
## TimeM1_36h00               3.23712    0.30538 211.55862  10.600  < 2e-16 ***
## TimeM2_06h00               1.02458    0.35921 211.20611   2.852 0.004771 ** 
## TimeM2_12h00               1.47604    0.35921 211.20611   4.109 5.68e-05 ***
## TimeM2_24h00               0.42398    0.35392 210.82482   1.198 0.232278    
## TimeM2_36h00              -0.05434    0.41754 210.54242  -0.130 0.896579    
## TimeM2_48h00               1.37657    0.31459 211.11933   4.376 1.90e-05 ***
## TimeM3_24h00               1.11344    0.41754 210.54242   2.667 0.008256 ** 
## TimeM3_48h00               3.33745    0.35231 210.89688   9.473  < 2e-16 ***
## genotypeMut:TimeM1_36h00   1.21544    0.34062 207.67764   3.568 0.000446 ***
## genotypeMut:TimeM2_06h00   0.93747    0.40959 207.26600   2.289 0.023100 *  
## genotypeMut:TimeM2_12h00   0.27831    0.40449 207.27470   0.688 0.492186    
## genotypeMut:TimeM2_24h00  -0.11504    0.42604 207.32854  -0.270 0.787410    
## genotypeMut:TimeM2_36h00   0.94560    0.50177 207.16699   1.885 0.060896 .  
## genotypeMut:TimeM2_48h00   0.64849    0.36744 207.26511   1.765 0.079057 .  
## genotypeMut:TimeM3_24h00   1.98774    0.50177 207.16699   3.961 0.000102 ***
## genotypeMut:TimeM3_48h00   0.53768    0.40959 207.26600   1.313 0.190728    
## ---
## Signif. codes:  0 '***' 0.001 '**' 0.01 '*' 0.05 '.' 0.1 ' ' 1
```

```
## 
## Correlation matrix not shown by default, as p = 18 > 12.
## Use print(x, correlation=TRUE)  or
##     vcov(x)        if you need it
```

```
hist(residuals(mod), nclass = 50)
```

Figure 3: Histogram of the residuals from the linear mixed models

We can compute the marginal effects of the fixed effects and their
interaction term.

```
Anova(mod)
```

```
## Analysis of Deviance Table (Type II Wald chisquare tests)
## 
## Response: dCt_NA
##                 Chisq Df Pr(>Chisq)    
## genotype       32.440  1   1.23e-08 ***
## Time          650.888  8  < 2.2e-16 ***
## genotype:Time  30.773  8  0.0001542 ***
## ---
## Signif. codes:  0 '***' 0.001 '**' 0.01 '*' 0.05 '.' 0.1 ' ' 1
```

We are interested in the `genotype` effect at each
timepoint.

```
emm.all <- emmeans(mod,  ~ genotype | Time)
pairs(emm.all)
```

```
## Time = iPS:
##  contrast estimate    SE  df t.ratio p.value
##  WT - Mut    0.114 0.259 208   0.441  0.6599
## 
## Time = M1_36h00:
##  contrast estimate    SE  df t.ratio p.value
##  WT - Mut   -1.101 0.212 207  -5.189  <.0001
## 
## Time = M2_06h00:
##  contrast estimate    SE  df t.ratio p.value
##  WT - Mut   -0.823 0.318 207  -2.593  0.0102
## 
## Time = M2_12h00:
##  contrast estimate    SE  df t.ratio p.value
##  WT - Mut   -0.164 0.311 207  -0.528  0.5980
## 
## Time = M2_24h00:
##  contrast estimate    SE  df t.ratio p.value
##  WT - Mut    0.229 0.336 207   0.683  0.4954
## 
## Time = M2_36h00:
##  contrast estimate    SE  df t.ratio p.value
##  WT - Mut   -0.831 0.430 207  -1.934  0.0545
## 
## Time = M2_48h00:
##  contrast estimate    SE  df t.ratio p.value
##  WT - Mut   -0.534 0.264 207  -2.027  0.0439
## 
## Time = M3_24h00:
##  contrast estimate    SE  df t.ratio p.value
##  WT - Mut   -1.874 0.430 207  -4.358  <.0001
## 
## Time = M3_48h00:
##  contrast estimate    SE  df t.ratio p.value
##  WT - Mut   -0.424 0.318 207  -1.334  0.1836
## 
## Degrees-of-freedom method: kenward-roger
```

We now adjust the p-values using the Benjamini-Hochberg to identify
at which timepoints the `dCT_NA` are significantly different
between WT and Mut.

```
p <- summary(pairs(emm.all))$p.value
adj.p <- p.adjust(p, method = "BH")
names(adj.p) <- levels(data$Time)
adj.p
```

```
##          iPS     M1_36h00     M2_06h00     M2_12h00     M2_24h00     M2_36h00 
## 6.599230e-01 4.524875e-06 3.056168e-02 6.599230e-01 6.369063e-01 9.803219e-02 
##     M2_48h00     M3_24h00     M3_48h00 
## 9.803219e-02 9.289781e-05 2.754734e-01
```

From these p-values, we can conclude that the genotype significantly
impacts the expression level of SRY at M1\_36h, M3\_24h and M2\_6h to a
lesser extent.

We can plot the marginal means estimated by the mixed model for the
`genotype`as a function of `Time`.

```
emmip(mod, genotype ~ Time)
```

Figure 4: Mean dCt predicted by the linear model as a function of time

This plot is the same as the previous one but using the actual data.
Note that this does not take into account the variability across
experiments.

```
df <- aggregate(data[, 3], by = list(data$genotype, data$Time), mean)
ggplot(df, aes(x=Group.2, y = x, group = Group.1)) + geom_line(aes(color=Group.1)) + geom_point(aes(color=Group.1))
```

Figure 5: Mean dCt computed from the actual data (across experiments) as
a function of time

# Interpretation

Overall, the `dCt`is higher in mutant mice, while
significantly only at certain timepoints, showing a lower expression of
SRY in mutant mice compared to wild-type.

```
sessionInfo()
```

```
## R version 4.3.2 (2023-10-31)
## Platform: aarch64-apple-darwin20 (64-bit)
## Running under: macOS Sonoma 14.3.1
## 
## Matrix products: default
## BLAS:   /Library/Frameworks/R.framework/Versions/4.3-arm64/Resources/lib/libRblas.0.dylib 
## LAPACK: /Library/Frameworks/R.framework/Versions/4.3-arm64/Resources/lib/libRlapack.dylib;  LAPACK version 3.11.0
## 
## locale:
## [1] en_US.UTF-8/en_US.UTF-8/en_US.UTF-8/C/en_US.UTF-8/en_US.UTF-8
## 
## time zone: Europe/Paris
## tzcode source: internal
## 
## attached base packages:
## [1] stats     graphics  grDevices utils     datasets  methods   base     
## 
## other attached packages:
##  [1] corrplot_0.92    car_3.1-2        carData_3.0-5    ggbeeswarm_0.7.2
##  [5] emmeans_1.9.0    lmerTest_3.1-3   lme4_1.1-35.1    Matrix_1.6-5    
##  [9] kableExtra_1.3.4 lubridate_1.9.3  forcats_1.0.0    stringr_1.5.1   
## [13] dplyr_1.1.4      purrr_1.0.2      readr_2.1.5      tidyr_1.3.0     
## [17] tibble_3.2.1     ggplot2_3.4.4    tidyverse_2.0.0 
## 
## loaded via a namespace (and not attached):
##  [1] tidyselect_1.2.0    viridisLite_0.4.2   farver_2.1.1       
##  [4] vipor_0.4.7         fastmap_1.1.1       TH.data_1.1-2      
##  [7] digest_0.6.34       estimability_1.4.1  timechange_0.3.0   
## [10] lifecycle_1.0.4     survival_3.5-7      magrittr_2.0.3     
## [13] compiler_4.3.2      rlang_1.1.3         sass_0.4.8         
## [16] tools_4.3.2         utf8_1.2.4          yaml_2.3.8         
## [19] knitr_1.45          labeling_0.4.3      xml2_1.3.6         
## [22] multcomp_1.4-25     abind_1.4-5         withr_3.0.0        
## [25] numDeriv_2016.8-1.1 grid_4.3.2          fansi_1.0.6        
## [28] xtable_1.8-4        colorspace_2.1-0    scales_1.3.0       
## [31] MASS_7.3-60.0.1     cli_3.6.2           mvtnorm_1.2-4      
## [34] rmarkdown_2.25      generics_0.1.3      rstudioapi_0.15.0  
## [37] httr_1.4.7          tzdb_0.4.0          minqa_1.2.6        
## [40] cachem_1.0.8        splines_4.3.2       parallel_4.3.2     
## [43] rvest_1.0.3         vctrs_0.6.5         boot_1.3-29        
## [46] webshot_0.5.5       sandwich_3.1-0      jsonlite_1.8.8     
## [49] hms_1.1.3           pbkrtest_0.5.2      beeswarm_0.4.0     
## [52] systemfonts_1.0.5   jquerylib_0.1.4     glue_1.7.0         
## [55] nloptr_2.0.3        codetools_0.2-19    stringi_1.8.3      
## [58] gtable_0.3.4        munsell_0.5.0       pillar_1.9.0       
## [61] htmltools_0.5.7     R6_2.5.1            evaluate_0.23      
## [64] lattice_0.22-5      highr_0.10          backports_1.4.1    
## [67] broom_1.0.5         bslib_0.6.1         Rcpp_1.0.12        
## [70] svglite_2.1.3       coda_0.19-4.1       nlme_3.1-164       
## [73] xfun_0.41           zoo_1.8-12          pkgconfig_2.0.3
```
