# Supplementary material for: A conserved NR5A1-responsive enhancer regulates SRY in testis-determination
Source: Nat Commun. 2024 Mar 30;15:2796. doi: 10.1038/s41467-024-47162-2 (PMC10981742; doi:10.1038/s41467-024-47162-2)
Supplement: Supplementary file 22 — Supplementary Dataset 19 [file 41467_2024_47162_MOESM22_ESM.html]

LMM\_SOX9


Code 

- Show All Code
- Hide All Code

# LMM\_SOX9

#### Vincent Laville

#### 2024-02-23

## Data

```
data <- read.table("data_pcr_sox9.txt", header = T, sep = "\t", dec = ".", fill = T)
data <- na.omit(data)
data$genotype <- factor(data$genotype, levels = c("WT", "Mut"))
data$Time <- factor(data$Time, 
                    levels = c("iPS", "M1_36h00", "M2_06h00", "M2_12h00", "M2_24h00", "M2_36h00", "M2_48h00", "M3_24h00", "M3_48h00"))
```

```
data %>%
  kbl() %>%
  kable_paper("hover", full_width = F) %>%
  kable_styling(bootstrap_options = c("striped", "hover")) %>%
  scroll_box(height = "300px")
```

|  | Sample\_Reference | genotype | dCt\_NA | Experiment | Time |
| --- | --- | --- | --- | --- | --- |
| 1 | 21-85 | WT | 8.218367 | iPS07 | M1\_36h00 |
| 2 | 21-86 | WT | 7.438973 | iPS07 | M1\_36h00 |
| 3 | 21-92 | Mut | 7.678646 | iPS07 | M1\_36h00 |
| 4 | 21-93 | Mut | 8.323477 | iPS07 | M1\_36h00 |
| 5 | 21-94 | Mut | 7.918076 | iPS07 | M1\_36h00 |
| 6 | 21-95 | Mut | 7.324906 | iPS07 | M1\_36h00 |
| 7 | 21-96 | Mut | 7.471019 | iPS07 | M1\_36h00 |
| 8 | 21-121 | WT | 7.155493 | iPS09 | M1\_36h00 |
| 9 | 21-122 | WT | 6.790472 | iPS09 | M1\_36h00 |
| 10 | 21-123 | WT | 7.016722 | iPS09 | M1\_36h00 |
| 11 | 21-124 | WT | 6.890930 | iPS09 | M1\_36h00 |
| 12 | 21-125 | WT | 7.129077 | iPS09 | M1\_36h00 |
| 13 | 21-126 | Mut | 6.508825 | iPS09 | M1\_36h00 |
| 14 | 21-127 | Mut | 6.393469 | iPS09 | M1\_36h00 |
| 15 | 21-128 | Mut | 6.223399 | iPS09 | M1\_36h00 |
| 16 | 21-129 | Mut | 6.215586 | iPS09 | M1\_36h00 |
| 17 | 21-130 | Mut | 6.137569 | iPS09 | M1\_36h00 |
| 18 | 22-59 | WT | 7.216948 | iPS12 | M1\_36h00 |
| 19 | 22-60 | WT | 7.410435 | iPS12 | M1\_36h00 |
| 20 | 22-61 | WT | 7.238029 | iPS12 | M1\_36h00 |
| 21 | 22-62 | WT | 7.030680 | iPS12 | M1\_36h00 |
| 22 | 22-63 | WT | 7.309076 | iPS12 | M1\_36h00 |
| 24 | 22-65 | Mut | 6.146671 | iPS12 | M1\_36h00 |
| 25 | 22-66 | Mut | 6.165782 | iPS12 | M1\_36h00 |
| 26 | 22-67 | Mut | 6.178772 | iPS12 | M1\_36h00 |
| 27 | 22-68 | Mut | 6.225652 | iPS12 | M1\_36h00 |
| 29 | 22-70 | Mut | 6.277790 | iPS12 | M1\_36h00 |
| 30 | 23-181 | WT | 7.805886 | iPS26a | M1\_36h00 |
| 31 | 23-182 | WT | 8.208788 | iPS26a | M1\_36h00 |
| 32 | 23-183 | WT | 8.270644 | iPS26a | M1\_36h00 |
| 33 | 23-184 | WT | 7.906137 | iPS26a | M1\_36h00 |
| 34 | 23-185 | WT | 8.309672 | iPS26a | M1\_36h00 |
| 35 | 23-186 | WT | 8.331521 | iPS26a | M1\_36h00 |
| 36 | 23-187 | WT | 8.659797 | iPS26b | M1\_36h00 |
| 37 | 23-188 | WT | 9.364963 | iPS26b | M1\_36h00 |
| 38 | 23-189 | WT | 8.528527 | iPS26b | M1\_36h00 |
| 39 | 23-190 | WT | 8.782401 | iPS26b | M1\_36h00 |
| 40 | 23-191 | WT | 9.599367 | iPS26b | M1\_36h00 |
| 41 | 23-192 | WT | 8.303328 | iPS26b | M1\_36h00 |
| 42 | 23-193 | Mut | 8.651905 | iPS26a | M1\_36h00 |
| 43 | 23-194 | Mut | 8.376658 | iPS26a | M1\_36h00 |
| 44 | 23-195 | Mut | 8.386757 | iPS26a | M1\_36h00 |
| 45 | 23-196 | Mut | 8.517802 | iPS26a | M1\_36h00 |
| 46 | 23-197 | Mut | 8.688960 | iPS26a | M1\_36h00 |
| 47 | 23-198 | Mut | 8.492869 | iPS26a | M1\_36h00 |
| 48 | 23-199 | Mut | 7.945904 | iPS26b | M1\_36h00 |
| 49 | 23-200 | Mut | 8.245824 | iPS26b | M1\_36h00 |
| 50 | 23-201 | Mut | 8.143124 | iPS26b | M1\_36h00 |
| 51 | 23-202 | Mut | 8.167578 | iPS26b | M1\_36h00 |
| 52 | 23-203 | Mut | 8.187634 | iPS26b | M1\_36h00 |
| 53 | 23-204 | Mut | 8.617719 | iPS26b | M1\_36h00 |
| 54 | 22-71 | WT | 8.647383 | iPS12 | M2\_06h00 |
| 55 | 22-72 | WT | 9.412343 | iPS12 | M2\_06h00 |
| 56 | 22-73 | WT | 9.096348 | iPS12 | M2\_06h00 |
| 57 | 22-74 | WT | 9.970729 | iPS12 | M2\_06h00 |
| 58 | 22-75 | WT | 9.938560 | iPS12 | M2\_06h00 |
| 61 | 22-78 | Mut | 8.184610 | iPS12 | M2\_06h00 |
| 62 | 22-79 | Mut | 9.098640 | iPS12 | M2\_06h00 |
| 63 | 22-80 | Mut | 9.536485 | iPS12 | M2\_06h00 |
| 64 | 22-81 | Mut | 9.921793 | iPS12 | M2\_06h00 |
| 65 | 22-82 | Mut | 9.317197 | iPS12 | M2\_06h00 |
| 66 | 23-205 | WT | 8.907813 | iPS26a | M2\_06h00 |
| 67 | 23-206 | WT | 9.083361 | iPS26a | M2\_06h00 |
| 68 | 23-207 | WT | 9.431648 | iPS26a | M2\_06h00 |
| 69 | 23-208 | WT | 9.592413 | iPS26b | M2\_06h00 |
| 70 | 23-209 | WT | 10.033072 | iPS26b | M2\_06h00 |
| 71 | 23-210 | WT | 9.555004 | iPS26b | M2\_06h00 |
| 72 | 23-211 | Mut | 9.213553 | iPS26a | M2\_06h00 |
| 73 | 23-212 | Mut | 9.090539 | iPS26a | M2\_06h00 |
| 74 | 23-213 | Mut | 9.233725 | iPS26a | M2\_06h00 |
| 75 | 23-214 | Mut | 8.873874 | iPS26b | M2\_06h00 |
| 76 | 23-215 | Mut | 9.282277 | iPS26b | M2\_06h00 |
| 77 | 23-216 | Mut | 9.747164 | iPS26b | M2\_06h00 |
| 79 | 22-84 | WT | 9.020415 | iPS12 | M2\_12h00 |
| 82 | 22-87 | WT | 9.084134 | iPS12 | M2\_12h00 |
| 83 | 22-88 | WT | 8.918715 | iPS12 | M2\_12h00 |
| 84 | 22-89 | Mut | 8.507957 | iPS12 | M2\_12h00 |
| 85 | 22-90 | Mut | 8.034786 | iPS12 | M2\_12h00 |
| 86 | 22-91 | Mut | 10.790611 | iPS12 | M2\_12h00 |
| 87 | 22-92 | Mut | 8.389786 | iPS12 | M2\_12h00 |
| 88 | 22-93 | Mut | 9.721986 | iPS12 | M2\_12h00 |
| 89 | 22-94 | Mut | 10.103291 | iPS12 | M2\_12h00 |
| 90 | 23-217 | WT | 8.711112 | iPS26a | M2\_12h00 |
| 91 | 23-218 | WT | 9.002685 | iPS26a | M2\_12h00 |
| 92 | 23-219 | WT | 9.338509 | iPS26a | M2\_12h00 |
| 93 | 23-220 | WT | 9.601033 | iPS26b | M2\_12h00 |
| 94 | 23-221 | WT | 10.156795 | iPS26b | M2\_12h00 |
| 95 | 23-222 | WT | 9.660587 | iPS26b | M2\_12h00 |
| 96 | 23-223 | Mut | 8.859579 | iPS26a | M2\_12h00 |
| 97 | 23-224 | Mut | 8.848585 | iPS26a | M2\_12h00 |
| 98 | 23-225 | Mut | 8.798873 | iPS26a | M2\_12h00 |
| 99 | 23-226 | Mut | 8.992127 | iPS26b | M2\_12h00 |
| 100 | 23-227 | Mut | 9.084956 | iPS26b | M2\_12h00 |
| 101 | 23-228 | Mut | 9.934845 | iPS26b | M2\_12h00 |
| 102 | 21-131 | WT | 7.072322 | iPS09 | M2\_24h00 |
| 103 | 21-132 | WT | 6.148624 | iPS09 | M2\_24h00 |
| 104 | 21-133 | WT | 6.861230 | iPS09 | M2\_24h00 |
| 105 | 21-134 | WT | 5.888372 | iPS09 | M2\_24h00 |
| 106 | 21-135 | WT | 5.811749 | iPS09 | M2\_24h00 |
| 107 | 21-136 | Mut | 5.876976 | iPS09 | M2\_24h00 |
| 108 | 21-137 | Mut | 6.042642 | iPS09 | M2\_24h00 |
| 109 | 21-138 | Mut | 5.872281 | iPS09 | M2\_24h00 |
| 110 | 21-139 | Mut | 5.887489 | iPS09 | M2\_24h00 |
| 111 | 21-140 | Mut | 6.076356 | iPS09 | M2\_24h00 |
| 112 | 22-95 | WT | 6.351077 | iPS12 | M2\_24h00 |
| 113 | 22-96 | WT | 7.320212 | iPS12 | M2\_24h00 |
| 114 | 22-97 | WT | 6.419391 | iPS12 | M2\_24h00 |
| 115 | 22-98 | WT | 6.707798 | iPS12 | M2\_24h00 |
| 116 | 22-99 | WT | 6.634364 | iPS12 | M2\_24h00 |
| 118 | 22-101 | Mut | 8.235849 | iPS12 | M2\_24h00 |
| 119 | 22-102 | Mut | 7.831369 | iPS12 | M2\_24h00 |
| 120 | 22-103 | Mut | 7.470043 | iPS12 | M2\_24h00 |
| 121 | 22-104 | Mut | 8.119498 | iPS12 | M2\_24h00 |
| 122 | 22-105 | Mut | 7.521480 | iPS12 | M2\_24h00 |
| 124 | 23-229 | WT | 7.802969 | iPS26a | M2\_36h00 |
| 125 | 23-230 | WT | 8.205312 | iPS26a | M2\_36h00 |
| 126 | 23-231 | WT | 7.656192 | iPS26a | M2\_36h00 |
| 127 | 23-232 | WT | 8.402710 | iPS26b | M2\_36h00 |
| 128 | 23-233 | WT | 8.795423 | iPS26b | M2\_36h00 |
| 129 | 23-234 | WT | 8.378504 | iPS26b | M2\_36h00 |
| 130 | 23-235 | Mut | 6.582401 | iPS26a | M2\_36h00 |
| 131 | 23-236 | Mut | 6.747534 | iPS26a | M2\_36h00 |
| 132 | 23-237 | Mut | 6.531096 | iPS26a | M2\_36h00 |
| 133 | 23-238 | Mut | 7.753417 | iPS26b | M2\_36h00 |
| 134 | 23-239 | Mut | 7.723957 | iPS26b | M2\_36h00 |
| 135 | 23-240 | Mut | 8.200265 | iPS26b | M2\_36h00 |
| 137 | 22-108 | WT | 6.848177 | iPS12 | M2\_48h00 |
| 138 | 22-109 | WT | 7.004958 | iPS12 | M2\_48h00 |
| 139 | 22-110 | WT | 6.707018 | iPS12 | M2\_48h00 |
| 140 | 22-111 | WT | 6.935081 | iPS12 | M2\_48h00 |
| 142 | 22-113 | Mut | 6.154825 | iPS12 | M2\_48h00 |
| 143 | 22-114 | Mut | 6.564262 | iPS12 | M2\_48h00 |
| 144 | 22-115 | Mut | 7.006395 | iPS12 | M2\_48h00 |
| 145 | 22-116 | Mut | 6.189247 | iPS12 | M2\_48h00 |
| 146 | 22-117 | Mut | 6.806225 | iPS12 | M2\_48h00 |
| 147 | 22-118 | Mut | 7.087976 | iPS12 | M2\_48h00 |
| 148 | 23-241 | WT | 6.685244 | iPS26a | M2\_48h00 |
| 149 | 23-242 | WT | 7.268945 | iPS26a | M2\_48h00 |
| 150 | 23-243 | WT | 7.125162 | iPS26a | M2\_48h00 |
| 151 | 23-244 | WT | 7.656195 | iPS26b | M2\_48h00 |
| 152 | 23-245 | WT | 8.237021 | iPS26b | M2\_48h00 |
| 153 | 23-246 | WT | 7.509246 | iPS26b | M2\_48h00 |
| 154 | 23-247 | Mut | 7.002440 | iPS26a | M2\_48h00 |
| 155 | 23-248 | Mut | 6.398525 | iPS26a | M2\_48h00 |
| 156 | 23-249 | Mut | 6.832315 | iPS26a | M2\_48h00 |
| 157 | 23-250 | Mut | 6.877812 | iPS26b | M2\_48h00 |
| 158 | 23-251 | Mut | 7.117477 | iPS26b | M2\_48h00 |
| 159 | 23-252 | Mut | 6.949720 | iPS26b | M2\_48h00 |
| 160 | 23-253 | WT | 6.013914 | iPS26a | M3\_24h00 |
| 161 | 23-254 | WT | 6.249470 | iPS26a | M3\_24h00 |
| 162 | 23-255 | WT | 6.339054 | iPS26a | M3\_24h00 |
| 163 | 23-256 | WT | 6.901734 | iPS26b | M3\_24h00 |
| 164 | 23-257 | WT | 7.495945 | iPS26b | M3\_24h00 |
| 165 | 23-258 | WT | 6.809646 | iPS26b | M3\_24h00 |
| 166 | 23-259 | Mut | 5.969940 | iPS26a | M3\_24h00 |
| 167 | 23-260 | Mut | 6.134913 | iPS26a | M3\_24h00 |
| 168 | 23-261 | Mut | 5.966927 | iPS26a | M3\_24h00 |
| 169 | 23-262 | Mut | 5.637016 | iPS26b | M3\_24h00 |
| 170 | 23-263 | Mut | 5.838424 | iPS26b | M3\_24h00 |
| 171 | 23-264 | Mut | 6.327909 | iPS26b | M3\_24h00 |
| 172 | 21-146 | WT | 5.258954 | iPS09 | M3\_48h00 |
| 173 | 21-147 | WT | 5.410088 | iPS09 | M3\_48h00 |
| 174 | 21-148 | WT | 5.203708 | iPS09 | M3\_48h00 |
| 175 | 21-149 | WT | 5.762150 | iPS09 | M3\_48h00 |
| 176 | 21-150 | WT | 6.472869 | iPS09 | M3\_48h00 |
| 177 | 21-151 | Mut | 5.540165 | iPS09 | M3\_48h00 |
| 178 | 21-152 | Mut | 5.188900 | iPS09 | M3\_48h00 |
| 179 | 21-153 | Mut | 5.219813 | iPS09 | M3\_48h00 |
| 180 | 21-154 | Mut | 4.884440 | iPS09 | M3\_48h00 |
| 182 | 23-265 | WT | 5.363178 | iPS26a | M3\_48h00 |
| 183 | 23-266 | WT | 5.996024 | iPS26a | M3\_48h00 |
| 184 | 23-267 | WT | 5.936177 | iPS26a | M3\_48h00 |
| 185 | 23-268 | WT | 5.940215 | iPS26b | M3\_48h00 |
| 186 | 23-269 | WT | 6.572287 | iPS26b | M3\_48h00 |
| 187 | 23-270 | WT | 6.301134 | iPS26b | M3\_48h00 |
| 188 | 23-271 | Mut | 6.074183 | iPS26a | M3\_48h00 |
| 189 | 23-272 | Mut | 6.149558 | iPS26a | M3\_48h00 |
| 190 | 23-273 | Mut | 6.485945 | iPS26a | M3\_48h00 |
| 191 | 23-274 | Mut | 5.658109 | iPS26b | M3\_48h00 |
| 192 | 23-275 | Mut | 5.861195 | iPS26b | M3\_48h00 |
| 193 | 23-276 | Mut | 6.101198 | iPS26b | M3\_48h00 |
| 194 | 21-107 | WT | 8.980294 | iPS07 | iPS |
| 195 | 21-108 | WT | 12.253138 | iPS07 | iPS |
| 196 | 21-109 | WT | 11.262143 | iPS07 | iPS |
| 197 | 21-110 | WT | 5.931992 | iPS07 | iPS |
| 198 | 21-111 | WT | 7.690653 | iPS07 | iPS |
| 199 | 21-112 | WT | 6.797914 | iPS07 | iPS |
| 200 | 21-113 | WT | 6.584005 | iPS07 | iPS |
| 201 | 21-115 | Mut | 7.904360 | iPS09 | iPS |
| 202 | 21-116 | Mut | 7.688884 | iPS09 | iPS |
| 203 | 21-117 | Mut | 7.521963 | iPS09 | iPS |
| 204 | 21-118 | Mut | 7.066838 | iPS09 | iPS |
| 205 | 21-119 | Mut | 8.406699 | iPS09 | iPS |
| 206 | 21-120 | Mut | 8.107497 | iPS09 | iPS |
| 208 | 23-158 | WT | 7.879775 | iPS19 | iPS |
| 209 | 23-159 | WT | 7.920730 | iPS19 | iPS |
| 210 | 23-160 | WT | 7.795535 | iPS19 | iPS |
| 211 | 23-161 | WT | 7.604310 | iPS19 | iPS |
| 212 | 23-162 | WT | 7.651695 | iPS19 | iPS |
| 213 | 23-169 | WT | 7.124371 | iPS19 | iPS |
| 214 | 23-170 | WT | 8.392830 | iPS19 | iPS |
| 215 | 23-171 | WT | 7.877830 | iPS19 | iPS |
| 217 | 23-173 | WT | 7.917894 | iPS19 | iPS |
| 218 | 23-174 | WT | 7.250314 | iPS19 | iPS |
| 219 | 23-163 | Mut | 7.699100 | iPS19 | iPS |
| 220 | 23-164 | Mut | 7.808285 | iPS19 | iPS |
| 221 | 23-165 | Mut | 8.324655 | iPS19 | iPS |
| 222 | 23-166 | Mut | 7.988800 | iPS19 | iPS |
| 223 | 23-167 | Mut | 8.286595 | iPS19 | iPS |
| 224 | 23-168 | Mut | 8.002540 | iPS19 | iPS |
| 225 | 23-175 | Mut | 7.786711 | iPS19 | iPS |
| 227 | 23-177 | Mut | 7.996272 | iPS19 | iPS |
| 228 | 23-178 | Mut | 8.175131 | iPS19 | iPS |
| 229 | 23-179 | Mut | 8.349876 | iPS19 | iPS |
| 230 | 23-180 | Mut | 8.746550 | iPS19 | iPS |

# Plots

We first examine the distribution of `dCt_NA` across the
different timepoints and colored by experiments.

```
ggplot(data = data, aes(x = genotype, y = dCt_NA)) +
         geom_boxplot(outlier.shape = NA) +
         geom_jitter(aes(colour = Experiment), size = 0.8) +
         theme_classic() + facet_grid(. ~ Time)
```

Figure 1: dCt (raw data) as a function of the genotype at each time
point

We next look at the distribution of `dCt_NA` across the
different experiments and colored by timepoints.

```
ggplot(data = data, aes(x = genotype, y = dCt_NA, colour = Time)) +
         geom_boxplot(outlier.shape = NA) +
         geom_jitter(position=position_jitterdodge(jitter.width = 0.1), size = 0.8) +
         theme_classic() + facet_grid(. ~ Experiment)
```

Figure 2: dCt (raw data) as a function of the genotype in each
experiment

# Analysis

We use a mixed-effect model, to analyse the dependent variable
`dCt_NA` with respect to:

- fixed effects (i.e., the `genotype` and
  `Time` variables)
- random effects (i.e., the `Experiment`
  variable)

We include an interaction term between `genotype`and
`Timepoint` as we are interested in the effect of
`genotype`at each `Timepoint` and the effect of
`genotype`seems not to be homogeneous at each timepoint.

We obtain the following:

```
mod = lmer(dCt_NA ~ genotype*Time + (1 | Experiment), data = data)

summary(mod)
```

```
## Linear mixed model fit by REML. t-tests use Satterthwaite's method [
## lmerModLmerTest]
## Formula: dCt_NA ~ genotype * Time + (1 | Experiment)
##    Data: data
## 
## REML criterion at convergence: 457.6
## 
## Scaled residuals: 
##     Min      1Q  Median      3Q     Max 
## -3.7315 -0.5306 -0.0415  0.3605  5.6969 
## 
## Random effects:
##  Groups     Name        Variance Std.Dev.
##  Experiment (Intercept) 0.2681   0.5178  
##  Residual               0.4495   0.6704  
## Number of obs: 215, groups:  Experiment, 6
## 
## Fixed effects:
##                           Estimate Std. Error        df t value Pr(>|t|)    
## (Intercept)                8.15495    0.29967  14.25120  27.213 1.09e-13 ***
## genotypeMut                0.33169    0.24767 196.60108   1.339 0.182038    
## TimeM1_36h00              -0.36183    0.28461 162.37753  -1.271 0.205427    
## TimeM2_06h00               1.05839    0.33577 169.58220   3.152 0.001917 ** 
## TimeM2_12h00               0.84969    0.34879 174.26223   2.436 0.015853 *  
## TimeM2_24h00              -1.23628    0.33557 179.83523  -3.684 0.000303 ***
## TimeM2_36h00              -0.38937    0.38564 183.42225  -1.010 0.313984    
## TimeM2_48h00              -1.19595    0.34160 171.79215  -3.501 0.000591 ***
## TimeM3_24h00              -1.96126    0.38564 183.42225  -5.086 9.00e-07 ***
## TimeM3_48h00              -2.22632    0.32728 176.86558  -6.803 1.53e-10 ***
## genotypeMut:TimeM1_36h00  -0.75834    0.31751 196.52502  -2.388 0.017869 *  
## genotypeMut:TimeM2_06h00  -0.52886    0.37824 194.61164  -1.398 0.163642    
## genotypeMut:TimeM2_12h00  -0.35212    0.38611 194.53838  -0.912 0.362920    
## genotypeMut:TimeM2_24h00   0.04019    0.38889 194.49677   0.103 0.917793    
## genotypeMut:TimeM2_36h00  -1.28210    0.45953 193.88784  -2.790 0.005797 ** 
## genotypeMut:TimeM2_48h00  -0.72982    0.37926 194.59818  -1.924 0.055773 .  
## genotypeMut:TimeM3_24h00  -0.98747    0.45953 193.88784  -2.149 0.032886 *  
## genotypeMut:TimeM3_48h00  -0.51707    0.38445 194.59300  -1.345 0.180205    
## ---
## Signif. codes:  0 '***' 0.001 '**' 0.01 '*' 0.05 '.' 0.1 ' ' 1
```

```
## 
## Correlation matrix not shown by default, as p = 18 > 12.
## Use print(x, correlation=TRUE)  or
##     vcov(x)        if you need it
```

```
hist(residuals(mod), nclass = 50)
```

Figure 3: Histogram of the residuals from the linear mixed models

We can compute the marginal effects of the fixed effects and their
interaction term.

```
Anova(mod)
```

```
## Analysis of Deviance Table (Type II Wald chisquare tests)
## 
## Response: dCt_NA
##                  Chisq Df Pr(>Chisq)    
## genotype        4.4017  1    0.03590 *  
## Time          461.9116  8    < 2e-16 ***
## genotype:Time  15.4779  8    0.05049 .  
## ---
## Signif. codes:  0 '***' 0.001 '**' 0.01 '*' 0.05 '.' 0.1 ' ' 1
```

We are interested in the `genotype` effect at each
timepoint.

```
emm.all <- emmeans(mod,  ~ genotype | Time)
pairs(emm.all)
```

```
## Time = iPS:
##  contrast estimate    SE  df t.ratio p.value
##  WT - Mut  -0.3317 0.249 197  -1.330  0.1851
## 
## Time = M1_36h00:
##  contrast estimate    SE  df t.ratio p.value
##  WT - Mut   0.4267 0.190 193   2.250  0.0256
## 
## Time = M2_06h00:
##  contrast estimate    SE  df t.ratio p.value
##  WT - Mut   0.1972 0.286 192   0.690  0.4912
## 
## Time = M2_12h00:
##  contrast estimate    SE  df t.ratio p.value
##  WT - Mut   0.0204 0.296 192   0.069  0.9451
## 
## Time = M2_24h00:
##  contrast estimate    SE  df t.ratio p.value
##  WT - Mut  -0.3719 0.300 192  -1.240  0.2164
## 
## Time = M2_36h00:
##  contrast estimate    SE  df t.ratio p.value
##  WT - Mut   0.9504 0.387 192   2.455  0.0150
## 
## Time = M2_48h00:
##  contrast estimate    SE  df t.ratio p.value
##  WT - Mut   0.3981 0.287 192   1.385  0.1675
## 
## Time = M3_24h00:
##  contrast estimate    SE  df t.ratio p.value
##  WT - Mut   0.6558 0.387 192   1.694  0.0919
## 
## Time = M3_48h00:
##  contrast estimate    SE  df t.ratio p.value
##  WT - Mut   0.1854 0.293 192   0.633  0.5278
## 
## Degrees-of-freedom method: kenward-roger
```

We now adjust the p-values using the Benjamini-Hochberg to identify
at which timepoints the `dCT_NA` are significantly different
between WT and Mut.

```
p <- summary(pairs(emm.all))$p.value
adj.p <- p.adjust(p, method = "BH")
names(adj.p) <- levels(data$Time)
adj.p
```

```
##       iPS  M1_36h00  M2_06h00  M2_12h00  M2_24h00  M2_36h00  M2_48h00  M3_24h00 
## 0.3245508 0.1149889 0.5937511 0.9451419 0.3245508 0.1149889 0.3245508 0.2755613 
##  M3_48h00 
## 0.5937511
```

From these p-values, we can conclude that the genotype does not
significantly impact the expression level of *SOX9* at any
timepoint.

We can plot the marginal means estimated by the mixed model for the
`genotype`as a function of `Time`.

```
emmip_output<-emmip(mod, genotype ~ Time)
emmip_dataframe<-emmip_output$data
emmip_dataframe
```

```
##  genotype Time     yvar    SE    df tvar xvar    
##  WT       iPS      8.15 0.305 14.11 WT   iPS     
##  Mut      iPS      8.49 0.297 13.72 Mut  iPS     
##  WT       M1_36h00 7.79 0.258  8.88 WT   M1_36h00
##  Mut      M1_36h00 7.37 0.251  8.14 Mut  M1_36h00
##  WT       M2_06h00 9.21 0.306 16.56 WT   M2_06h00
##  Mut      M2_06h00 9.02 0.306 16.56 Mut  M2_06h00
##  WT       M2_12h00 9.00 0.320 19.74 WT   M2_12h00
##  Mut      M2_12h00 8.98 0.300 15.46 Mut  M2_12h00
##  WT       M2_24h00 6.92 0.312 18.30 WT   M2_24h00
##  Mut      M2_24h00 7.29 0.312 18.30 Mut  M2_24h00
##  WT       M2_36h00 7.77 0.360 30.48 WT   M2_36h00
##  Mut      M2_36h00 6.82 0.360 30.48 Mut  M2_36h00
##  WT       M2_48h00 6.96 0.312 17.94 WT   M2_48h00
##  Mut      M2_48h00 6.56 0.300 15.46 Mut  M2_48h00
##  WT       M3_24h00 6.19 0.360 30.48 WT   M3_24h00
##  Mut      M3_24h00 5.54 0.360 30.48 Mut  M3_24h00
##  WT       M3_48h00 5.93 0.302 16.25 WT   M3_48h00
##  Mut      M3_48h00 5.74 0.308 17.62 Mut  M3_48h00
## 
## Degrees-of-freedom method: kenward-roger
```

```
emmip(mod, genotype ~ Time)
```

Figure 4: Mean dCt predicted by the linear model as a function of time

This plot is the same as the previous one but using the actual data.
Note that this does not take into account the variability across
experiments.

```
df <- aggregate(data[, 3], by = list(data$genotype, data$Time), mean)
ggplot(df, aes(x=Group.2, y = x, group = Group.1)) + geom_line(aes(color=Group.1)) + geom_point(aes(color=Group.1))
```

Figure 5: Mean dCt computed from the actual data (across experiments) as
a function of time

# Interpretation

Overall, the genotype does not affect *SOX9* expression at any
timepoint.

```
sessionInfo()
```

```
## R version 4.3.2 (2023-10-31)
## Platform: aarch64-apple-darwin20 (64-bit)
## Running under: macOS Sonoma 14.3.1
## 
## Matrix products: default
## BLAS:   /Library/Frameworks/R.framework/Versions/4.3-arm64/Resources/lib/libRblas.0.dylib 
## LAPACK: /Library/Frameworks/R.framework/Versions/4.3-arm64/Resources/lib/libRlapack.dylib;  LAPACK version 3.11.0
## 
## locale:
## [1] en_US.UTF-8/en_US.UTF-8/en_US.UTF-8/C/en_US.UTF-8/en_US.UTF-8
## 
## time zone: Europe/Paris
## tzcode source: internal
## 
## attached base packages:
## [1] stats     graphics  grDevices utils     datasets  methods   base     
## 
## other attached packages:
##  [1] corrplot_0.92    car_3.1-2        carData_3.0-5    ggbeeswarm_0.7.2
##  [5] emmeans_1.9.0    lmerTest_3.1-3   lme4_1.1-35.1    Matrix_1.6-5    
##  [9] kableExtra_1.3.4 lubridate_1.9.3  forcats_1.0.0    stringr_1.5.1   
## [13] dplyr_1.1.4      purrr_1.0.2      readr_2.1.5      tidyr_1.3.0     
## [17] tibble_3.2.1     ggplot2_3.4.4    tidyverse_2.0.0 
## 
## loaded via a namespace (and not attached):
##  [1] tidyselect_1.2.0    viridisLite_0.4.2   farver_2.1.1       
##  [4] vipor_0.4.7         fastmap_1.1.1       TH.data_1.1-2      
##  [7] digest_0.6.34       estimability_1.4.1  timechange_0.3.0   
## [10] lifecycle_1.0.4     survival_3.5-7      magrittr_2.0.3     
## [13] compiler_4.3.2      rlang_1.1.3         sass_0.4.8         
## [16] tools_4.3.2         utf8_1.2.4          yaml_2.3.8         
## [19] knitr_1.45          labeling_0.4.3      xml2_1.3.6         
## [22] multcomp_1.4-25     abind_1.4-5         withr_3.0.0        
## [25] numDeriv_2016.8-1.1 grid_4.3.2          fansi_1.0.6        
## [28] xtable_1.8-4        colorspace_2.1-0    scales_1.3.0       
## [31] MASS_7.3-60.0.1     cli_3.6.2           mvtnorm_1.2-4      
## [34] rmarkdown_2.25      generics_0.1.3      rstudioapi_0.15.0  
## [37] httr_1.4.7          tzdb_0.4.0          minqa_1.2.6        
## [40] cachem_1.0.8        splines_4.3.2       parallel_4.3.2     
## [43] rvest_1.0.3         vctrs_0.6.5         boot_1.3-29        
## [46] webshot_0.5.5       sandwich_3.1-0      jsonlite_1.8.8     
## [49] hms_1.1.3           pbkrtest_0.5.2      beeswarm_0.4.0     
## [52] systemfonts_1.0.5   jquerylib_0.1.4     glue_1.7.0         
## [55] nloptr_2.0.3        codetools_0.2-19    stringi_1.8.3      
## [58] gtable_0.3.4        munsell_0.5.0       pillar_1.9.0       
## [61] htmltools_0.5.7     R6_2.5.1            evaluate_0.23      
## [64] lattice_0.22-5      highr_0.10          backports_1.4.1    
## [67] broom_1.0.5         bslib_0.6.1         Rcpp_1.0.12        
## [70] svglite_2.1.3       coda_0.19-4.1       nlme_3.1-164       
## [73] xfun_0.41           zoo_1.8-12          pkgconfig_2.0.3
```
