# Supplementary material for: A conserved NR5A1-responsive enhancer regulates SRY in testis-determination
Source: Nat Commun. 2024 Mar 30;15:2796. doi: 10.1038/s41467-024-47162-2 (PMC10981742; doi:10.1038/s41467-024-47162-2)
Supplement: Supplementary file 24 — Supplementary Dataset 21 [file 41467_2024_47162_MOESM24_ESM.html]

LMM\_NR5A1


Code 

- Show All Code
- Hide All Code

# LMM\_NR5A1

#### Vincent Laville

#### 2024-02-23

## Data

```
data <- read.table("data_pcr_Nr5a1.txt", header = T, sep = "\t", dec = ".", fill = T)
data <- na.omit(data)
data$genotype <- factor(data$genotype, levels = c("WT", "Mut"))
data$Time <- factor(data$Time, 
                    levels = c("iPS", "M1_36h00", "M2_06h00", "M2_12h00", "M2_24h00", "M2_36h00", "M2_48h00", "M3_24h00", "M3_48h00"))
```

```
data %>%
  kbl() %>%
  kable_paper("hover", full_width = F) %>%
  kable_styling(bootstrap_options = c("striped", "hover")) %>%
  scroll_box(height = "300px")
```

|  | Sample\_Reference | genotype | dCt\_NA | Experiment | Time |
| --- | --- | --- | --- | --- | --- |
| 1 | 23-181 | WT | 15.93525 | iPS26a | M1\_36h00 |
| 2 | 23-182 | WT | 15.69317 | iPS26a | M1\_36h00 |
| 3 | 23-183 | WT | 16.16930 | iPS26a | M1\_36h00 |
| 4 | 23-184 | WT | 16.27874 | iPS26a | M1\_36h00 |
| 5 | 23-185 | WT | 15.68251 | iPS26a | M1\_36h00 |
| 6 | 23-186 | WT | 16.32325 | iPS26a | M1\_36h00 |
| 7 | 23-187 | WT | 16.86910 | iPS26b | M1\_36h00 |
| 8 | 23-188 | WT | 16.78333 | iPS26b | M1\_36h00 |
| 9 | 23-189 | WT | 17.69486 | iPS26b | M1\_36h00 |
| 10 | 23-190 | WT | 16.92030 | iPS26b | M1\_36h00 |
| 11 | 23-191 | WT | 16.61325 | iPS26b | M1\_36h00 |
| 12 | 23-192 | WT | 16.58839 | iPS26b | M1\_36h00 |
| 13 | 23-193 | Mut | 17.15275 | iPS26a | M1\_36h00 |
| 14 | 23-194 | Mut | 16.26768 | iPS26a | M1\_36h00 |
| 15 | 23-195 | Mut | 16.84527 | iPS26a | M1\_36h00 |
| 16 | 23-196 | Mut | 16.49696 | iPS26a | M1\_36h00 |
| 17 | 23-197 | Mut | 16.60151 | iPS26a | M1\_36h00 |
| 18 | 23-198 | Mut | 16.59320 | iPS26a | M1\_36h00 |
| 19 | 23-199 | Mut | 17.27887 | iPS26b | M1\_36h00 |
| 20 | 23-200 | Mut | 16.80976 | iPS26b | M1\_36h00 |
| 21 | 23-201 | Mut | 17.09308 | iPS26b | M1\_36h00 |
| 22 | 23-202 | Mut | 17.80665 | iPS26b | M1\_36h00 |
| 23 | 23-203 | Mut | 17.06839 | iPS26b | M1\_36h00 |
| 24 | 23-204 | Mut | 17.05429 | iPS26b | M1\_36h00 |
| 25 | 21-85 | WT | 14.87994 | iPS07 | M1\_36h00 |
| 26 | 21-86 | WT | 13.64461 | iPS07 | M1\_36h00 |
| 27 | 21-92 | Mut | 14.45938 | iPS07 | M1\_36h00 |
| 29 | 21-94 | Mut | 14.46123 | iPS07 | M1\_36h00 |
| 31 | 21-96 | Mut | 14.33790 | iPS07 | M1\_36h00 |
| 33 | 21-122 | WT | 14.14750 | iPS09 | M1\_36h00 |
| 35 | 21-124 | WT | 14.13711 | iPS09 | M1\_36h00 |
| 36 | 21-125 | WT | 14.19118 | iPS09 | M1\_36h00 |
| 37 | 21-126 | Mut | 13.88132 | iPS09 | M1\_36h00 |
| 38 | 21-127 | Mut | 13.46469 | iPS09 | M1\_36h00 |
| 39 | 21-128 | Mut | 13.87612 | iPS09 | M1\_36h00 |
| 40 | 21-129 | Mut | 13.26429 | iPS09 | M1\_36h00 |
| 41 | 21-130 | Mut | 13.90608 | iPS09 | M1\_36h00 |
| 42 | 22-59 | WT | 14.99565 | iPS12 | M1\_36h00 |
| 43 | 22-60 | WT | 13.68718 | iPS12 | M1\_36h00 |
| 44 | 22-61 | WT | 13.61628 | iPS12 | M1\_36h00 |
| 45 | 22-62 | WT | 13.99041 | iPS12 | M1\_36h00 |
| 46 | 22-63 | WT | 15.51876 | iPS12 | M1\_36h00 |
| 47 | 22-64 | WT | 13.48627 | iPS12 | M1\_36h00 |
| 48 | 22-65 | Mut | 12.78204 | iPS12 | M1\_36h00 |
| 49 | 22-66 | Mut | 13.32247 | iPS12 | M1\_36h00 |
| 50 | 22-67 | Mut | 14.84016 | iPS12 | M1\_36h00 |
| 51 | 22-68 | Mut | 13.65921 | iPS12 | M1\_36h00 |
| 52 | 22-69 | Mut | 12.66083 | iPS12 | M1\_36h00 |
| 53 | 22-70 | Mut | 12.46426 | iPS12 | M1\_36h00 |
| 54 | 23-205 | WT | 14.81993 | iPS26a | M2\_06h00 |
| 55 | 23-206 | WT | 15.09718 | iPS26a | M2\_06h00 |
| 56 | 23-207 | WT | 14.83693 | iPS26a | M2\_06h00 |
| 57 | 23-208 | WT | 15.58483 | iPS26b | M2\_06h00 |
| 58 | 23-209 | WT | 15.55476 | iPS26b | M2\_06h00 |
| 59 | 23-210 | WT | 15.98872 | iPS26b | M2\_06h00 |
| 60 | 23-211 | Mut | 15.99732 | iPS26a | M2\_06h00 |
| 61 | 23-212 | Mut | 16.09822 | iPS26a | M2\_06h00 |
| 62 | 23-213 | Mut | 16.05793 | iPS26a | M2\_06h00 |
| 63 | 23-214 | Mut | 16.32574 | iPS26b | M2\_06h00 |
| 64 | 23-215 | Mut | 16.13133 | iPS26b | M2\_06h00 |
| 65 | 23-216 | Mut | 15.90713 | iPS26b | M2\_06h00 |
| 66 | 22-71 | WT | 14.71000 | iPS12 | M2\_06h00 |
| 67 | 22-72 | WT | 15.28000 | iPS12 | M2\_06h00 |
| 68 | 22-73 | WT | 14.15000 | iPS12 | M2\_06h00 |
| 69 | 22-74 | WT | 15.69000 | iPS12 | M2\_06h00 |
| 70 | 22-75 | WT | 14.97000 | iPS12 | M2\_06h00 |
| 72 | 22-77 | Mut | 13.64000 | iPS12 | M2\_06h00 |
| 73 | 22-78 | Mut | 14.29000 | iPS12 | M2\_06h00 |
| 74 | 22-79 | Mut | 15.26000 | iPS12 | M2\_06h00 |
| 75 | 22-80 | Mut | 15.86000 | iPS12 | M2\_06h00 |
| 76 | 22-81 | Mut | 17.16000 | iPS12 | M2\_06h00 |
| 77 | 22-82 | Mut | 15.18000 | iPS12 | M2\_06h00 |
| 78 | 23-217 | WT | 15.28146 | iPS26a | M2\_12h00 |
| 79 | 23-218 | WT | 15.17375 | iPS26a | M2\_12h00 |
| 80 | 23-219 | WT | 15.77424 | iPS26a | M2\_12h00 |
| 81 | 23-220 | WT | 16.01012 | iPS26b | M2\_12h00 |
| 82 | 23-221 | WT | 16.25421 | iPS26b | M2\_12h00 |
| 83 | 23-222 | WT | 17.12576 | iPS26b | M2\_12h00 |
| 84 | 23-223 | Mut | 15.96393 | iPS26a | M2\_12h00 |
| 85 | 23-224 | Mut | 16.69962 | iPS26a | M2\_12h00 |
| 86 | 23-225 | Mut | 15.76397 | iPS26a | M2\_12h00 |
| 87 | 23-226 | Mut | 16.12905 | iPS26b | M2\_12h00 |
| 88 | 23-227 | Mut | 16.36967 | iPS26b | M2\_12h00 |
| 89 | 23-228 | Mut | 16.04792 | iPS26b | M2\_12h00 |
| 90 | 22-83 | WT | 15.61508 | iPS12 | M2\_12h00 |
| 91 | 22-84 | WT | 17.54580 | iPS12 | M2\_12h00 |
| 92 | 22-85 | WT | 16.06078 | iPS12 | M2\_12h00 |
| 93 | 22-86 | WT | 18.48849 | iPS12 | M2\_12h00 |
| 94 | 22-87 | WT | 16.63318 | iPS12 | M2\_12h00 |
| 95 | 22-88 | WT | 17.04741 | iPS12 | M2\_12h00 |
| 96 | 22-89 | Mut | 17.10482 | iPS12 | M2\_12h00 |
| 98 | 22-91 | Mut | 17.08836 | iPS12 | M2\_12h00 |
| 99 | 22-92 | Mut | 16.97066 | iPS12 | M2\_12h00 |
| 100 | 22-93 | Mut | 17.16529 | iPS12 | M2\_12h00 |
| 103 | 21-132 | WT | 11.76000 | iPS09 | M2\_24h00 |
| 104 | 21-133 | WT | 11.79000 | iPS09 | M2\_24h00 |
| 105 | 21-134 | WT | 11.78000 | iPS09 | M2\_24h00 |
| 107 | 21-136 | Mut | 11.51000 | iPS09 | M2\_24h00 |
| 108 | 21-137 | Mut | 11.55000 | iPS09 | M2\_24h00 |
| 109 | 21-138 | Mut | 11.54000 | iPS09 | M2\_24h00 |
| 110 | 21-139 | Mut | 11.46000 | iPS09 | M2\_24h00 |
| 111 | 21-140 | Mut | 11.58000 | iPS09 | M2\_24h00 |
| 113 | 22-96 | WT | 16.23000 | iPS12 | M2\_24h00 |
| 114 | 22-97 | WT | 15.03000 | iPS12 | M2\_24h00 |
| 115 | 22-98 | WT | 15.54000 | iPS12 | M2\_24h00 |
| 116 | 22-99 | WT | 15.46000 | iPS12 | M2\_24h00 |
| 118 | 22-101 | Mut | 16.52000 | iPS12 | M2\_24h00 |
| 119 | 22-102 | Mut | 16.04000 | iPS12 | M2\_24h00 |
| 120 | 22-103 | Mut | 15.58000 | iPS12 | M2\_24h00 |
| 121 | 22-104 | Mut | 15.47000 | iPS12 | M2\_24h00 |
| 122 | 22-105 | Mut | 15.08000 | iPS12 | M2\_24h00 |
| 123 | 22-106 | Mut | 17.36000 | iPS12 | M2\_24h00 |
| 124 | 23-229 | WT | 16.46554 | iPS26a | M2\_36h00 |
| 125 | 23-230 | WT | 16.26834 | iPS26a | M2\_36h00 |
| 126 | 23-231 | WT | 16.33327 | iPS26a | M2\_36h00 |
| 127 | 23-232 | WT | 16.95216 | iPS26b | M2\_36h00 |
| 128 | 23-233 | WT | 16.68838 | iPS26b | M2\_36h00 |
| 129 | 23-234 | WT | 17.42594 | iPS26b | M2\_36h00 |
| 130 | 23-235 | Mut | 16.85774 | iPS26a | M2\_36h00 |
| 131 | 23-236 | Mut | 16.98782 | iPS26a | M2\_36h00 |
| 132 | 23-237 | Mut | 16.95107 | iPS26a | M2\_36h00 |
| 133 | 23-238 | Mut | 16.89116 | iPS26b | M2\_36h00 |
| 134 | 23-239 | Mut | 16.12063 | iPS26b | M2\_36h00 |
| 135 | 23-240 | Mut | 16.78755 | iPS26b | M2\_36h00 |
| 136 | 23-241 | WT | 17.11957 | iPS26a | M2\_48h00 |
| 137 | 23-242 | WT | 17.16512 | iPS26a | M2\_48h00 |
| 138 | 23-243 | WT | 17.24895 | iPS26a | M2\_48h00 |
| 139 | 23-244 | WT | 17.41967 | iPS26b | M2\_48h00 |
| 140 | 23-245 | WT | 16.50069 | iPS26b | M2\_48h00 |
| 141 | 23-246 | WT | 18.37977 | iPS26b | M2\_48h00 |
| 142 | 23-247 | Mut | 17.21121 | iPS26a | M2\_48h00 |
| 143 | 23-248 | Mut | 17.04853 | iPS26a | M2\_48h00 |
| 144 | 23-249 | Mut | 17.89892 | iPS26a | M2\_48h00 |
| 145 | 23-250 | Mut | 18.22235 | iPS26b | M2\_48h00 |
| 146 | 23-251 | Mut | 17.30764 | iPS26b | M2\_48h00 |
| 147 | 23-252 | Mut | 16.75654 | iPS26b | M2\_48h00 |
| 148 | 21-87 | WT | 13.81000 | iPS07 | M2\_48h00 |
| 149 | 21-88 | WT | 13.89000 | iPS07 | M2\_48h00 |
| 151 | 21-90 | WT | 13.65000 | iPS07 | M2\_48h00 |
| 152 | 21-91 | WT | 13.75000 | iPS07 | M2\_48h00 |
| 153 | 21-97 | Mut | 14.73000 | iPS07 | M2\_48h00 |
| 154 | 21-98 | Mut | 15.44000 | iPS07 | M2\_48h00 |
| 155 | 21-99 | Mut | 14.87000 | iPS07 | M2\_48h00 |
| 156 | 21-100 | Mut | 14.74000 | iPS07 | M2\_48h00 |
| 157 | 21-101 | Mut | 15.65000 | iPS07 | M2\_48h00 |
| 158 | 22-107 | WT | 13.57166 | iPS12 | M2\_48h00 |
| 159 | 22-108 | WT | 15.38534 | iPS12 | M2\_48h00 |
| 160 | 22-109 | WT | 14.97476 | iPS12 | M2\_48h00 |
| 161 | 22-110 | WT | 13.62202 | iPS12 | M2\_48h00 |
| 162 | 22-111 | WT | 14.13200 | iPS12 | M2\_48h00 |
| 164 | 22-113 | Mut | 13.20281 | iPS12 | M2\_48h00 |
| 165 | 22-114 | Mut | 14.14377 | iPS12 | M2\_48h00 |
| 166 | 22-115 | Mut | 14.02785 | iPS12 | M2\_48h00 |
| 167 | 22-116 | Mut | 13.73658 | iPS12 | M2\_48h00 |
| 168 | 22-117 | Mut | 14.29802 | iPS12 | M2\_48h00 |
| 170 | 23-253 | WT | 16.67669 | iPS26a | M3\_24h00 |
| 171 | 23-254 | WT | 16.98704 | iPS26a | M3\_24h00 |
| 172 | 23-255 | WT | 16.46159 | iPS26a | M3\_24h00 |
| 173 | 23-256 | WT | 17.13162 | iPS26b | M3\_24h00 |
| 174 | 23-257 | WT | 16.84997 | iPS26b | M3\_24h00 |
| 175 | 23-258 | WT | 16.99158 | iPS26b | M3\_24h00 |
| 176 | 23-259 | Mut | 16.59091 | iPS26a | M3\_24h00 |
| 177 | 23-260 | Mut | 16.28996 | iPS26a | M3\_24h00 |
| 178 | 23-261 | Mut | 16.66397 | iPS26a | M3\_24h00 |
| 179 | 23-262 | Mut | 18.17070 | iPS26b | M3\_24h00 |
| 180 | 23-263 | Mut | 17.12913 | iPS26b | M3\_24h00 |
| 181 | 23-264 | Mut | 17.27384 | iPS26b | M3\_24h00 |
| 182 | 21-141 | WT | 13.22818 | iPS09 | M1\_36h00 |
| 183 | 21-142 | WT | 13.60437 | iPS09 | M1\_36h00 |
| 184 | 21-143 | WT | 14.69554 | iPS09 | M2\_24h00 |
| 185 | 21-144 | WT | 11.87530 | iPS09 | M2\_24h00 |
| 187 | 23-265 | WT | 15.79067 | iPS26a | M3\_48h00 |
| 188 | 23-266 | WT | 16.18406 | iPS26a | M3\_48h00 |
| 189 | 23-267 | WT | 16.88410 | iPS26a | M3\_48h00 |
| 190 | 23-268 | WT | 16.07465 | iPS26b | M3\_48h00 |
| 191 | 23-269 | WT | 15.83675 | iPS26b | M3\_48h00 |
| 192 | 23-270 | WT | 15.81726 | iPS26b | M3\_48h00 |
| 193 | 23-271 | Mut | 15.83947 | iPS26a | M3\_48h00 |
| 194 | 23-272 | Mut | 16.67408 | iPS26a | M3\_48h00 |
| 195 | 23-273 | Mut | 16.00582 | iPS26a | M3\_48h00 |
| 196 | 23-274 | Mut | 17.15091 | iPS26b | M3\_48h00 |
| 197 | 23-275 | Mut | 17.31805 | iPS26b | M3\_48h00 |
| 198 | 23-276 | Mut | 17.36706 | iPS26b | M3\_48h00 |
| 199 | 21-146 | WT | 16.96000 | iPS09 | M3\_48h00 |
| 200 | 21-147 | WT | 17.28000 | iPS09 | M3\_48h00 |
| 201 | 21-148 | WT | 17.51000 | iPS09 | M3\_48h00 |
| 202 | 21-149 | WT | 18.63000 | iPS09 | M3\_48h00 |
| 203 | 21-150 | WT | 18.80000 | iPS09 | M3\_48h00 |
| 204 | 21-151 | Mut | 17.46000 | iPS09 | M3\_48h00 |
| 205 | 21-152 | Mut | 17.63000 | iPS09 | M3\_48h00 |
| 206 | 21-153 | Mut | 17.38000 | iPS09 | M3\_48h00 |
| 207 | 21-154 | Mut | 17.82000 | iPS09 | M3\_48h00 |
| 209 | 23-157 | WT | 15.54511 | iPS19 | iPS |
| 210 | 23-158 | WT | 13.99728 | iPS19 | iPS |
| 211 | 23-159 | WT | 14.47138 | iPS19 | iPS |
| 212 | 23-160 | WT | 14.06730 | iPS19 | iPS |
| 213 | 23-161 | WT | 14.60817 | iPS19 | iPS |
| 214 | 23-162 | WT | 14.08945 | iPS19 | iPS |
| 215 | 23-169 | WT | 13.25888 | iPS19 | iPS |
| 217 | 23-171 | WT | 13.62191 | iPS19 | iPS |
| 218 | 23-172 | WT | 15.77999 | iPS19 | iPS |
| 219 | 23-173 | WT | 15.08137 | iPS19 | iPS |
| 221 | 23-163 | Mut | 14.32595 | iPS19 | iPS |
| 222 | 23-164 | Mut | 14.95611 | iPS19 | iPS |
| 223 | 23-165 | Mut | 14.94556 | iPS19 | iPS |
| 224 | 23-166 | Mut | 14.88377 | iPS19 | iPS |
| 225 | 23-167 | Mut | 15.38277 | iPS19 | iPS |
| 226 | 23-168 | Mut | 14.79571 | iPS19 | iPS |
| 227 | 23-175 | Mut | 13.74068 | iPS19 | iPS |
| 228 | 23-176 | Mut | 15.06372 | iPS19 | iPS |
| 229 | 23-177 | Mut | 14.13629 | iPS19 | iPS |
| 230 | 23-178 | Mut | 14.07195 | iPS19 | iPS |
| 231 | 23-179 | Mut | 13.90870 | iPS19 | iPS |
| 232 | 23-180 | Mut | 14.94306 | iPS19 | iPS |

# Plots

We first examine the distribution of `dCt_NA` across the
different timepoints and colored by experiments.

```
ggplot(data = data, aes(x = genotype, y = dCt_NA)) +
         geom_boxplot(outlier.shape = NA) +
         geom_jitter(aes(colour = Experiment), size = 0.8) +
         theme_classic() + facet_grid(. ~ Time)
```

Figure 1: dCt (raw data) as a function of the genotype at each time
point

We next look at the distribution of `dCt_NA` across the
different experiments and colored by timepoints.

```
ggplot(data = data, aes(x = genotype, y = dCt_NA, colour = Time)) +
         geom_boxplot(outlier.shape = NA) +
         geom_jitter(position=position_jitterdodge(jitter.width = 0.1), size = 0.8) +
         theme_classic() + facet_grid(. ~ Experiment)
```

Figure 2: dCt (raw data) as a function of the genotype in each
experiment

# Analysis

We use a mixed-effect model, to analyse the dependent variable
`dCt_NA` with respect to:

- fixed effects (i.e., the `genotype` and
  `Time` variables)
- random effects (i.e., the `Experiment`
  variable)

We include an interaction term between `genotype`and
`Timepoint` as we are interested in the effect of
`genotype`at each `Timepoint` and the effect of
`genotype`seems not to be homogeneous at each timepoint.

We obtain the following:

```
mod = lmer(dCt_NA ~ genotype*Time + (1 | Experiment), data = data)

summary(mod)
```

```
## Linear mixed model fit by REML. t-tests use Satterthwaite's method [
## lmerModLmerTest]
## Formula: dCt_NA ~ genotype * Time + (1 | Experiment)
##    Data: data
## 
## REML criterion at convergence: 666.2
## 
## Scaled residuals: 
##      Min       1Q   Median       3Q      Max 
## -2.19211 -0.64718  0.01732  0.62394  2.57978 
## 
## Random effects:
##  Groups     Name        Variance Std.Dev.
##  Experiment (Intercept) 0.8409   0.917   
##  Residual               1.3262   1.152   
## Number of obs: 214, groups:  Experiment, 6
## 
## Fixed effects:
##                           Estimate Std. Error        df t value Pr(>|t|)    
## (Intercept)               14.45208    0.98666   4.70823  14.647 4.16e-05 ***
## genotypeMut                0.14411    0.49309 191.96797   0.292   0.7704    
## TimeM1_36h00               0.61886    1.09376   4.93549   0.566   0.5963    
## TimeM2_06h00               0.24545    1.12711   5.55865   0.218   0.8354    
## TimeM2_12h00               1.55301    1.12285   5.47547   1.383   0.2204    
## TimeM2_24h00              -0.08615    1.14267   5.86254  -0.075   0.9424    
## TimeM2_36h00               1.35170    1.17334   6.51830   1.152   0.2898    
## TimeM2_48h00               0.78372    1.11059   5.24457   0.706   0.5105    
## TimeM3_24h00               1.51251    1.17334   6.51830   1.289   0.2412    
## TimeM3_48h00               2.39773    1.12774   5.56813   2.126   0.0812 .  
## genotypeMut:TimeM1_36h00  -0.16602    0.58935 191.98528  -0.282   0.7785    
## genotypeMut:TimeM2_06h00   0.40490    0.68871 191.97112   0.588   0.5573    
## genotypeMut:TimeM2_12h00  -0.12584    0.69768 191.98284  -0.180   0.8570    
## genotypeMut:TimeM2_24h00  -0.06066    0.71549 192.00607  -0.085   0.9325    
## genotypeMut:TimeM2_36h00  -0.06705    0.82778 191.96797  -0.081   0.9355    
## genotypeMut:TimeM2_48h00   0.11593    0.64395 191.99125   0.180   0.8573    
## genotypeMut:TimeM3_24h00   0.02590    0.82778 191.96797   0.031   0.9751    
## genotypeMut:TimeM3_48h00  -0.06916    0.70467 191.97987  -0.098   0.9219    
## ---
## Signif. codes:  0 '***' 0.001 '**' 0.01 '*' 0.05 '.' 0.1 ' ' 1
```

```
## 
## Correlation matrix not shown by default, as p = 18 > 12.
## Use print(x, correlation=TRUE)  or
##     vcov(x)        if you need it
```

```
hist(residuals(mod), nclass = 50)
```

Figure 3: Histogram of the residuals from the linear mixed models

We can compute the marginal effects of the fixed effects and their
interaction term.

```
Anova(mod)
```

```
## Analysis of Deviance Table (Type II Wald chisquare tests)
## 
## Response: dCt_NA
##                 Chisq Df Pr(>Chisq)    
## genotype       0.7569  1     0.3843    
## Time          63.1893  8  1.099e-10 ***
## genotype:Time  1.1595  8     0.9970    
## ---
## Signif. codes:  0 '***' 0.001 '**' 0.01 '*' 0.05 '.' 0.1 ' ' 1
```

We are interested in the `genotype` effect at each
timepoint.

```
emm.all <- emmeans(mod,  ~ genotype | Time)
pairs(emm.all)
```

```
## Time = iPS:
##  contrast estimate    SE  df t.ratio p.value
##  WT - Mut  -0.1441 0.493 192  -0.292  0.7704
## 
## Time = M1_36h00:
##  contrast estimate    SE  df t.ratio p.value
##  WT - Mut   0.0219 0.323 192   0.068  0.9460
## 
## Time = M2_06h00:
##  contrast estimate    SE  df t.ratio p.value
##  WT - Mut  -0.5490 0.481 192  -1.142  0.2550
## 
## Time = M2_12h00:
##  contrast estimate    SE  df t.ratio p.value
##  WT - Mut  -0.0183 0.494 192  -0.037  0.9705
## 
## Time = M2_24h00:
##  contrast estimate    SE  df t.ratio p.value
##  WT - Mut  -0.0834 0.518 192  -0.161  0.8723
## 
## Time = M2_36h00:
##  contrast estimate    SE  df t.ratio p.value
##  WT - Mut  -0.0771 0.665 192  -0.116  0.9079
## 
## Time = M2_48h00:
##  contrast estimate    SE  df t.ratio p.value
##  WT - Mut  -0.2600 0.414 192  -0.628  0.5309
## 
## Time = M3_24h00:
##  contrast estimate    SE  df t.ratio p.value
##  WT - Mut  -0.1700 0.665 192  -0.256  0.7985
## 
## Time = M3_48h00:
##  contrast estimate    SE  df t.ratio p.value
##  WT - Mut  -0.0749 0.503 192  -0.149  0.8818
## 
## Degrees-of-freedom method: kenward-roger
```

We now adjust the p-values using the Benjamini-Hochberg to identify
at which timepoints the `dCT_NA` are significantly different
between WT and Mut.

```
p <- summary(pairs(emm.all))$p.value
adj.p <- p.adjust(p, method = "BH")
names(adj.p) <- levels(data$Time)
adj.p
```

```
##       iPS  M1_36h00  M2_06h00  M2_12h00  M2_24h00  M2_36h00  M2_48h00  M3_24h00 
## 0.9705236 0.9705236 0.9705236 0.9705236 0.9705236 0.9705236 0.9705236 0.9705236 
##  M3_48h00 
## 0.9705236
```

From these p-values, we can conclude that the genotype does not
significantly impact the expression level of *NR5A1* at any
timepoint.

We can plot the marginal means estimated by the mixed model for the
`genotype`as a function of `Time`.

```
emmip(mod, genotype ~ Time)
```

Figure 4: Mean dCt predicted by the linear model as a function of time

This plot is the same as the previous one but using the actual data.
Note that this does not take into account the variability across
experiments.

```
df <- aggregate(data[, 3], by = list(data$genotype, data$Time), mean)
ggplot(df, aes(x=Group.2, y = x, group = Group.1)) + geom_line(aes(color=Group.1)) + geom_point(aes(color=Group.1))
```

Figure 5: Mean dCt computed from the actual data (across experiments) as
a function of time

# Interpretation

Overall, the genotype does not affect *NR5A1* expression at
any timepoint.

```
sessionInfo()
```

```
## R version 4.3.2 (2023-10-31)
## Platform: aarch64-apple-darwin20 (64-bit)
## Running under: macOS Sonoma 14.3.1
## 
## Matrix products: default
## BLAS:   /Library/Frameworks/R.framework/Versions/4.3-arm64/Resources/lib/libRblas.0.dylib 
## LAPACK: /Library/Frameworks/R.framework/Versions/4.3-arm64/Resources/lib/libRlapack.dylib;  LAPACK version 3.11.0
## 
## locale:
## [1] en_US.UTF-8/en_US.UTF-8/en_US.UTF-8/C/en_US.UTF-8/en_US.UTF-8
## 
## time zone: Europe/Paris
## tzcode source: internal
## 
## attached base packages:
## [1] stats     graphics  grDevices utils     datasets  methods   base     
## 
## other attached packages:
##  [1] corrplot_0.92    car_3.1-2        carData_3.0-5    ggbeeswarm_0.7.2
##  [5] emmeans_1.9.0    lmerTest_3.1-3   lme4_1.1-35.1    Matrix_1.6-5    
##  [9] kableExtra_1.3.4 lubridate_1.9.3  forcats_1.0.0    stringr_1.5.1   
## [13] dplyr_1.1.4      purrr_1.0.2      readr_2.1.5      tidyr_1.3.0     
## [17] tibble_3.2.1     ggplot2_3.4.4    tidyverse_2.0.0 
## 
## loaded via a namespace (and not attached):
##  [1] tidyselect_1.2.0    viridisLite_0.4.2   farver_2.1.1       
##  [4] vipor_0.4.7         fastmap_1.1.1       TH.data_1.1-2      
##  [7] digest_0.6.34       estimability_1.4.1  timechange_0.3.0   
## [10] lifecycle_1.0.4     survival_3.5-7      magrittr_2.0.3     
## [13] compiler_4.3.2      rlang_1.1.3         sass_0.4.8         
## [16] tools_4.3.2         utf8_1.2.4          yaml_2.3.8         
## [19] knitr_1.45          labeling_0.4.3      xml2_1.3.6         
## [22] multcomp_1.4-25     abind_1.4-5         withr_3.0.0        
## [25] numDeriv_2016.8-1.1 grid_4.3.2          fansi_1.0.6        
## [28] xtable_1.8-4        colorspace_2.1-0    scales_1.3.0       
## [31] MASS_7.3-60.0.1     cli_3.6.2           mvtnorm_1.2-4      
## [34] rmarkdown_2.25      generics_0.1.3      rstudioapi_0.15.0  
## [37] httr_1.4.7          tzdb_0.4.0          minqa_1.2.6        
## [40] cachem_1.0.8        splines_4.3.2       parallel_4.3.2     
## [43] rvest_1.0.3         vctrs_0.6.5         boot_1.3-29        
## [46] webshot_0.5.5       sandwich_3.1-0      jsonlite_1.8.8     
## [49] hms_1.1.3           pbkrtest_0.5.2      beeswarm_0.4.0     
## [52] systemfonts_1.0.5   jquerylib_0.1.4     glue_1.7.0         
## [55] nloptr_2.0.3        codetools_0.2-19    stringi_1.8.3      
## [58] gtable_0.3.4        munsell_0.5.0       pillar_1.9.0       
## [61] htmltools_0.5.7     R6_2.5.1            evaluate_0.23      
## [64] lattice_0.22-5      highr_0.10          backports_1.4.1    
## [67] broom_1.0.5         bslib_0.6.1         Rcpp_1.0.12        
## [70] svglite_2.1.3       coda_0.19-4.1       nlme_3.1-164       
## [73] xfun_0.41           zoo_1.8-12          pkgconfig_2.0.3
```
