# Supplementary material for: A conserved NR5A1-responsive enhancer regulates SRY in testis-determination
Source: Nat Commun. 2024 Mar 30;15:2796. doi: 10.1038/s41467-024-47162-2 (PMC10981742; doi:10.1038/s41467-024-47162-2)
Supplement: Supplementary file 25 — Supplementary Dataset 22 [file 41467_2024_47162_MOESM25_ESM.html]

LMM\_FOXL2


Code 

- Show All Code
- Hide All Code

# LMM\_FOXL2

#### Vincent Laville

#### 2024-02-23

## Data

```
data <- read.table("data_pcr_foxl2.txt", header = T, sep = "\t", dec = ".", fill = T)
data <- na.omit(data)
data$genotype <- factor(data$genotype, levels = c("WT", "Mut"))
data$Time <- factor(data$Time, 
                    levels = c("iPS", "M1_36h00", "M2_06h00", "M2_12h00", "M2_24h00", "M2_36h00", "M2_48h00", "M3_24h00", "M3_48h00"))
```

```
data %>%
  kbl() %>%
  kable_paper("hover", full_width = F) %>%
  kable_styling(bootstrap_options = c("striped", "hover")) %>%
  scroll_box(height = "300px")
```

|  | Sample\_Reference | genotype | dCt\_NA | Experiment | Time |
| --- | --- | --- | --- | --- | --- |
| 1 | 21-121 | WT | 13.337956 | iPS09 | M1\_36h00 |
| 2 | 21-122 | WT | 13.084560 | iPS09 | M1\_36h00 |
| 3 | 21-123 | WT | 11.576045 | iPS09 | M1\_36h00 |
| 4 | 21-124 | WT | 11.619881 | iPS09 | M1\_36h00 |
| 5 | 21-125 | WT | 11.865814 | iPS09 | M1\_36h00 |
| 7 | 21-127 | Mut | 12.164787 | iPS09 | M1\_36h00 |
| 8 | 21-128 | Mut | 11.722782 | iPS09 | M1\_36h00 |
| 9 | 21-129 | Mut | 11.716826 | iPS09 | M1\_36h00 |
| 10 | 21-130 | Mut | 11.442282 | iPS09 | M1\_36h00 |
| 11 | 22-59 | WT | 11.282935 | iPS12 | M1\_36h00 |
| 12 | 22-60 | WT | 11.365839 | iPS12 | M1\_36h00 |
| 13 | 22-61 | WT | 11.253326 | iPS12 | M1\_36h00 |
| 14 | 22-62 | WT | 11.605450 | iPS12 | M1\_36h00 |
| 15 | 22-63 | WT | 11.629164 | iPS12 | M1\_36h00 |
| 16 | 22-64 | WT | 11.157555 | iPS12 | M1\_36h00 |
| 17 | 22-65 | Mut | 11.364332 | iPS12 | M1\_36h00 |
| 18 | 22-66 | Mut | 11.428680 | iPS12 | M1\_36h00 |
| 19 | 22-67 | Mut | 11.614505 | iPS12 | M1\_36h00 |
| 20 | 22-68 | Mut | 11.662143 | iPS12 | M1\_36h00 |
| 21 | 22-69 | Mut | 11.170114 | iPS12 | M1\_36h00 |
| 22 | 22-70 | Mut | 11.292369 | iPS12 | M1\_36h00 |
| 23 | 23-181 | WT | 13.038457 | iPS26a | M1\_36h00 |
| 24 | 23-182 | WT | 12.170283 | iPS26a | M1\_36h00 |
| 25 | 23-183 | WT | 12.669509 | iPS26a | M1\_36h00 |
| 26 | 23-184 | WT | 12.930616 | iPS26a | M1\_36h00 |
| 27 | 23-185 | WT | 12.226934 | iPS26a | M1\_36h00 |
| 28 | 23-186 | WT | 12.457699 | iPS26a | M1\_36h00 |
| 29 | 23-187 | WT | 12.533833 | iPS26b | M1\_36h00 |
| 30 | 23-188 | WT | 11.626739 | iPS26b | M1\_36h00 |
| 31 | 23-189 | WT | 11.873219 | iPS26b | M1\_36h00 |
| 32 | 23-190 | WT | 12.100039 | iPS26b | M1\_36h00 |
| 33 | 23-191 | WT | 11.467009 | iPS26b | M1\_36h00 |
| 34 | 23-192 | WT | 12.053930 | iPS26b | M1\_36h00 |
| 35 | 23-193 | Mut | 11.377914 | iPS26a | M1\_36h00 |
| 36 | 23-194 | Mut | 10.982492 | iPS26a | M1\_36h00 |
| 37 | 23-195 | Mut | 11.049905 | iPS26a | M1\_36h00 |
| 38 | 23-196 | Mut | 11.346475 | iPS26a | M1\_36h00 |
| 39 | 23-197 | Mut | 11.493789 | iPS26a | M1\_36h00 |
| 40 | 23-198 | Mut | 11.094538 | iPS26a | M1\_36h00 |
| 41 | 23-199 | Mut | 12.506595 | iPS26b | M1\_36h00 |
| 42 | 23-200 | Mut | 12.581479 | iPS26b | M1\_36h00 |
| 43 | 23-201 | Mut | 12.465068 | iPS26b | M1\_36h00 |
| 44 | 23-202 | Mut | 12.400149 | iPS26b | M1\_36h00 |
| 45 | 23-203 | Mut | 12.430682 | iPS26b | M1\_36h00 |
| 46 | 23-204 | Mut | 12.273001 | iPS26b | M1\_36h00 |
| 47 | 22-71 | WT | 12.157657 | iPS12 | M2\_06h00 |
| 48 | 22-72 | WT | 12.592781 | iPS12 | M2\_06h00 |
| 49 | 22-73 | WT | 11.830259 | iPS12 | M2\_06h00 |
| 50 | 22-74 | WT | 12.588354 | iPS12 | M2\_06h00 |
| 51 | 22-75 | WT | 12.535159 | iPS12 | M2\_06h00 |
| 54 | 22-78 | Mut | 12.436714 | iPS12 | M2\_06h00 |
| 56 | 22-80 | Mut | 12.521514 | iPS12 | M2\_06h00 |
| 57 | 22-81 | Mut | 12.512382 | iPS12 | M2\_06h00 |
| 58 | 22-82 | Mut | 12.509683 | iPS12 | M2\_06h00 |
| 59 | 23-205 | WT | 12.244605 | iPS26a | M2\_06h00 |
| 60 | 23-206 | WT | 11.497357 | iPS26a | M2\_06h00 |
| 61 | 23-207 | WT | 12.133846 | iPS26a | M2\_06h00 |
| 62 | 23-208 | WT | 11.562959 | iPS26b | M2\_06h00 |
| 63 | 23-209 | WT | 10.908604 | iPS26b | M2\_06h00 |
| 64 | 23-210 | WT | 11.398949 | iPS26b | M2\_06h00 |
| 65 | 23-211 | Mut | 10.781353 | iPS26a | M2\_06h00 |
| 66 | 23-212 | Mut | 10.788540 | iPS26a | M2\_06h00 |
| 67 | 23-213 | Mut | 10.581849 | iPS26a | M2\_06h00 |
| 68 | 23-214 | Mut | 12.004765 | iPS26b | M2\_06h00 |
| 69 | 23-215 | Mut | 12.086323 | iPS26b | M2\_06h00 |
| 70 | 23-216 | Mut | 11.616654 | iPS26b | M2\_06h00 |
| 71 | 22-83 | WT | 12.288523 | iPS12 | M2\_12h00 |
| 72 | 22-84 | WT | 12.764523 | iPS12 | M2\_12h00 |
| 73 | 22-85 | WT | 12.960047 | iPS12 | M2\_12h00 |
| 74 | 22-86 | WT | 13.278755 | iPS12 | M2\_12h00 |
| 75 | 22-87 | WT | 13.388270 | iPS12 | M2\_12h00 |
| 76 | 22-88 | WT | 12.966432 | iPS12 | M2\_12h00 |
| 77 | 22-89 | Mut | 12.861193 | iPS12 | M2\_12h00 |
| 78 | 22-90 | Mut | 13.173791 | iPS12 | M2\_12h00 |
| 79 | 22-91 | Mut | 13.326238 | iPS12 | M2\_12h00 |
| 80 | 22-92 | Mut | 13.136984 | iPS12 | M2\_12h00 |
| 81 | 22-93 | Mut | 13.630663 | iPS12 | M2\_12h00 |
| 82 | 22-94 | Mut | 13.369786 | iPS12 | M2\_12h00 |
| 83 | 23-217 | WT | 11.584188 | iPS26a | M2\_12h00 |
| 84 | 23-218 | WT | 11.711983 | iPS26a | M2\_12h00 |
| 85 | 23-219 | WT | 12.077395 | iPS26a | M2\_12h00 |
| 86 | 23-220 | WT | 11.329320 | iPS26b | M2\_12h00 |
| 87 | 23-221 | WT | 11.083722 | iPS26b | M2\_12h00 |
| 88 | 23-222 | WT | 11.637389 | iPS26b | M2\_12h00 |
| 89 | 23-223 | Mut | 10.753638 | iPS26a | M2\_12h00 |
| 90 | 23-224 | Mut | 10.318623 | iPS26a | M2\_12h00 |
| 91 | 23-225 | Mut | 10.714294 | iPS26a | M2\_12h00 |
| 92 | 23-226 | Mut | 12.288255 | iPS26b | M2\_12h00 |
| 93 | 23-227 | Mut | 12.203779 | iPS26b | M2\_12h00 |
| 94 | 23-228 | Mut | 12.230887 | iPS26b | M2\_12h00 |
| 95 | 21-131 | WT | 11.494323 | iPS09 | M2\_24h00 |
| 96 | 21-132 | WT | 11.364604 | iPS09 | M2\_24h00 |
| 97 | 21-133 | WT | 10.988557 | iPS09 | M2\_24h00 |
| 98 | 21-134 | WT | 10.854532 | iPS09 | M2\_24h00 |
| 99 | 21-135 | WT | 11.100658 | iPS09 | M2\_24h00 |
| 100 | 21-136 | Mut | 10.928738 | iPS09 | M2\_24h00 |
| 101 | 21-137 | Mut | 11.260628 | iPS09 | M2\_24h00 |
| 102 | 21-138 | Mut | 11.196057 | iPS09 | M2\_24h00 |
| 103 | 21-139 | Mut | 11.536154 | iPS09 | M2\_24h00 |
| 104 | 21-140 | Mut | 10.927618 | iPS09 | M2\_24h00 |
| 106 | 22-96 | WT | 10.732655 | iPS12 | M2\_24h00 |
| 107 | 22-97 | WT | 10.301692 | iPS12 | M2\_24h00 |
| 108 | 22-98 | WT | 10.411897 | iPS12 | M2\_24h00 |
| 109 | 22-99 | WT | 10.775214 | iPS12 | M2\_24h00 |
| 110 | 22-100 | WT | 10.934202 | iPS12 | M2\_24h00 |
| 111 | 22-101 | Mut | 11.477062 | iPS12 | M2\_24h00 |
| 112 | 22-102 | Mut | 11.133537 | iPS12 | M2\_24h00 |
| 113 | 22-103 | Mut | 11.406934 | iPS12 | M2\_24h00 |
| 114 | 22-104 | Mut | 11.443709 | iPS12 | M2\_24h00 |
| 115 | 22-105 | Mut | 11.123864 | iPS12 | M2\_24h00 |
| 116 | 22-106 | Mut | 11.757652 | iPS12 | M2\_24h00 |
| 117 | 23-229 | WT | 13.241090 | iPS26a | M2\_36h00 |
| 118 | 23-230 | WT | 12.650210 | iPS26a | M2\_36h00 |
| 119 | 23-231 | WT | 13.185615 | iPS26a | M2\_36h00 |
| 120 | 23-232 | WT | 12.026748 | iPS26b | M2\_36h00 |
| 121 | 23-233 | WT | 11.747257 | iPS26b | M2\_36h00 |
| 122 | 23-234 | WT | 12.162182 | iPS26b | M2\_36h00 |
| 123 | 23-235 | Mut | 11.264146 | iPS26a | M2\_36h00 |
| 124 | 23-236 | Mut | 11.262795 | iPS26a | M2\_36h00 |
| 125 | 23-237 | Mut | 11.190652 | iPS26a | M2\_36h00 |
| 126 | 23-238 | Mut | 13.188003 | iPS26b | M2\_36h00 |
| 127 | 23-239 | Mut | 13.323400 | iPS26b | M2\_36h00 |
| 128 | 23-240 | Mut | 12.902325 | iPS26b | M2\_36h00 |
| 129 | 22-107 | WT | 10.988852 | iPS12 | M2\_48h00 |
| 130 | 22-108 | WT | 11.297395 | iPS12 | M2\_48h00 |
| 131 | 22-109 | WT | 11.480168 | iPS12 | M2\_48h00 |
| 133 | 22-111 | WT | 11.382371 | iPS12 | M2\_48h00 |
| 135 | 22-113 | Mut | 10.827781 | iPS12 | M2\_48h00 |
| 136 | 22-114 | Mut | 11.349204 | iPS12 | M2\_48h00 |
| 137 | 22-115 | Mut | 11.081779 | iPS12 | M2\_48h00 |
| 138 | 22-116 | Mut | 11.364933 | iPS12 | M2\_48h00 |
| 139 | 22-117 | Mut | 11.561078 | iPS12 | M2\_48h00 |
| 141 | 23-241 | WT | 13.070153 | iPS26a | M2\_48h00 |
| 142 | 23-242 | WT | 12.614371 | iPS26a | M2\_48h00 |
| 143 | 23-243 | WT | 12.545440 | iPS26a | M2\_48h00 |
| 144 | 23-244 | WT | 12.123823 | iPS26b | M2\_48h00 |
| 145 | 23-245 | WT | 11.712057 | iPS26b | M2\_48h00 |
| 146 | 23-246 | WT | 12.280992 | iPS26b | M2\_48h00 |
| 147 | 23-247 | Mut | 11.140827 | iPS26a | M2\_48h00 |
| 148 | 23-248 | Mut | 10.703077 | iPS26a | M2\_48h00 |
| 149 | 23-249 | Mut | 10.904479 | iPS26a | M2\_48h00 |
| 150 | 23-250 | Mut | 12.649731 | iPS26b | M2\_48h00 |
| 151 | 23-251 | Mut | 12.039384 | iPS26b | M2\_48h00 |
| 152 | 23-252 | Mut | 12.547921 | iPS26b | M2\_48h00 |
| 153 | 23-253 | WT | 10.741589 | iPS26a | M3\_24h00 |
| 154 | 23-254 | WT | 10.518086 | iPS26a | M3\_24h00 |
| 155 | 23-255 | WT | 11.084452 | iPS26a | M3\_24h00 |
| 156 | 23-256 | WT | 11.358186 | iPS26b | M3\_24h00 |
| 157 | 23-257 | WT | 10.878327 | iPS26b | M3\_24h00 |
| 158 | 23-258 | WT | 11.561467 | iPS26b | M3\_24h00 |
| 159 | 23-259 | Mut | 10.633142 | iPS26a | M3\_24h00 |
| 160 | 23-260 | Mut | 10.629014 | iPS26a | M3\_24h00 |
| 161 | 23-261 | Mut | 10.546863 | iPS26a | M3\_24h00 |
| 162 | 23-262 | Mut | 10.483668 | iPS26b | M3\_24h00 |
| 163 | 23-263 | Mut | 10.270163 | iPS26b | M3\_24h00 |
| 164 | 23-264 | Mut | 10.450552 | iPS26b | M3\_24h00 |
| 165 | 21-146 | WT | 15.383508 | iPS09 | M3\_48h00 |
| 166 | 21-147 | WT | 16.025615 | iPS09 | M3\_48h00 |
| 167 | 21-148 | WT | 14.902767 | iPS09 | M3\_48h00 |
| 168 | 21-149 | WT | 15.685615 | iPS09 | M3\_48h00 |
| 169 | 21-150 | WT | 15.249559 | iPS09 | M3\_48h00 |
| 170 | 21-151 | Mut | 15.774586 | iPS09 | M3\_48h00 |
| 171 | 21-152 | Mut | 15.033618 | iPS09 | M3\_48h00 |
| 172 | 21-153 | Mut | 15.495136 | iPS09 | M3\_48h00 |
| 173 | 21-154 | Mut | 14.585554 | iPS09 | M3\_48h00 |
| 174 | 21-155 | Mut | 13.996400 | iPS09 | M3\_48h00 |
| 175 | 23-265 | WT | 8.960779 | iPS26a | M3\_48h00 |
| 176 | 23-266 | WT | 8.899738 | iPS26a | M3\_48h00 |
| 177 | 23-267 | WT | 9.426874 | iPS26a | M3\_48h00 |
| 178 | 23-268 | WT | 11.167173 | iPS26b | M3\_48h00 |
| 179 | 23-269 | WT | 10.355144 | iPS26b | M3\_48h00 |
| 180 | 23-270 | WT | 10.974110 | iPS26b | M3\_48h00 |
| 181 | 23-271 | Mut | 10.656358 | iPS26a | M3\_48h00 |
| 182 | 23-272 | Mut | 10.505435 | iPS26a | M3\_48h00 |
| 183 | 23-273 | Mut | 10.815110 | iPS26a | M3\_48h00 |
| 184 | 23-274 | Mut | 9.984331 | iPS26b | M3\_48h00 |
| 185 | 23-275 | Mut | 9.844891 | iPS26b | M3\_48h00 |
| 186 | 23-276 | Mut | 9.678084 | iPS26b | M3\_48h00 |
| 187 | 23-157 | WT | 11.479421 | iPS19 | iPS |
| 188 | 23-158 | WT | 12.231807 | iPS19 | iPS |
| 189 | 23-159 | WT | 11.421067 | iPS19 | iPS |
| 190 | 23-160 | WT | 11.264953 | iPS19 | iPS |
| 191 | 23-161 | WT | 11.676599 | iPS19 | iPS |
| 192 | 23-162 | WT | 11.722400 | iPS19 | iPS |
| 193 | 23-169 | WT | 11.023532 | iPS19 | iPS |
| 194 | 23-170 | WT | 11.743751 | iPS19 | iPS |
| 195 | 23-171 | WT | 11.844603 | iPS19 | iPS |
| 196 | 23-172 | WT | 11.704054 | iPS19 | iPS |
| 197 | 23-173 | WT | 11.098149 | iPS19 | iPS |
| 199 | 23-163 | Mut | 11.010954 | iPS19 | iPS |
| 200 | 23-164 | Mut | 10.969934 | iPS19 | iPS |
| 201 | 23-165 | Mut | 11.396824 | iPS19 | iPS |
| 202 | 23-166 | Mut | 11.521512 | iPS19 | iPS |
| 203 | 23-167 | Mut | 11.252229 | iPS19 | iPS |
| 204 | 23-168 | Mut | 10.664352 | iPS19 | iPS |
| 205 | 23-175 | Mut | 11.202104 | iPS19 | iPS |
| 206 | 23-176 | Mut | 11.437625 | iPS19 | iPS |
| 207 | 23-177 | Mut | 10.395768 | iPS19 | iPS |
| 208 | 23-178 | Mut | 10.277360 | iPS19 | iPS |
| 209 | 23-179 | Mut | 11.107523 | iPS19 | iPS |
| 210 | 23-180 | Mut | 11.276966 | iPS19 | iPS |

# Plots

We first examine the distribution of `dCt_NA` across the
different timepoints and colored by experiments.

```
ggplot(data = data, aes(x = genotype, y = dCt_NA)) +
         geom_boxplot(outlier.shape = NA) +
         geom_jitter(aes(colour = Experiment), size = 0.8) +
         theme_classic() + facet_grid(. ~ Time)
```

Figure 1: dCt (raw data) as a function of the genotype at each time
point

We next look at the distribution of `dCt_NA` across the
different experiments and colored by timepoints.

```
ggplot(data = data, aes(x = genotype, y = dCt_NA, colour = Time)) +
         geom_boxplot(outlier.shape = NA) +
         geom_jitter(position=position_jitterdodge(jitter.width = 0.1), size = 0.8) +
         theme_classic() + facet_grid(. ~ Experiment)
```

Figure 2: dCt (raw data) as a function of the genotype in each
experiment

# Analysis

We use a mixed-effect model, to analyse the dependent variable
`dCt_NA` with respect to:

- fixed effects (i.e., the `genotype` and
  `Time` variables)
- random effects (i.e., the `Experiment`
  variable)

We include an interaction term between `genotype`and
`Timepoint` as we are interested in the effect of
`genotype`at each `Timepoint` and the effect of
`genotype`seems not to be homogeneous at each timepoint.

We obtain the following:

```
mod = lmer(dCt_NA ~ genotype*Time + (1 | Experiment), data = data)

summary(mod)
```

```
## Linear mixed model fit by REML. t-tests use Satterthwaite's method [
## lmerModLmerTest]
## Formula: dCt_NA ~ genotype * Time + (1 | Experiment)
##    Data: data
## 
## REML criterion at convergence: 544.6
## 
## Scaled residuals: 
##      Min       1Q   Median       3Q      Max 
## -2.73087 -0.60065  0.02483  0.51625  2.74760 
## 
## Random effects:
##  Groups     Name        Variance Std.Dev.
##  Experiment (Intercept) 0.8222   0.9067  
##  Residual               0.8600   0.9274  
## Number of obs: 201, groups:  Experiment, 5
## 
## Fixed effects:
##                            Estimate Std. Error         df t value Pr(>|t|)    
## (Intercept)               1.156e+01  9.489e-01  3.305e+00  12.188 0.000735 ***
## genotypeMut              -5.218e-01  3.871e-01  1.799e+02  -1.348 0.179357    
## TimeM1_36h00              5.301e-01  1.069e+00  3.411e+00   0.496 0.650291    
## TimeM2_06h00              7.283e-01  1.090e+00  3.677e+00   0.668 0.543487    
## TimeM2_12h00              1.006e+00  1.087e+00  3.639e+00   0.926 0.411659    
## TimeM2_24h00             -1.289e+00  1.095e+00  3.747e+00  -1.177 0.308452    
## TimeM2_36h00              1.558e+00  1.121e+00  4.113e+00   1.390 0.235066    
## TimeM2_48h00              7.611e-01  1.093e+00  3.724e+00   0.696 0.527263    
## TimeM3_24h00              7.921e-02  1.121e+00  4.113e+00   0.071 0.946959    
## TimeM3_48h00              6.627e-01  1.089e+00  3.674e+00   0.608 0.578576    
## genotypeMut:TimeM1_36h00  2.490e-01  4.758e-01  1.799e+02   0.523 0.601330    
## genotypeMut:TimeM2_06h00  3.890e-01  5.605e-01  1.799e+02   0.694 0.488522    
## genotypeMut:TimeM2_12h00  5.999e-01  5.415e-01  1.799e+02   1.108 0.269340    
## genotypeMut:TimeM2_24h00  9.734e-01  5.605e-01  1.799e+02   1.737 0.084138 .  
## genotypeMut:TimeM2_36h00  2.082e-01  6.607e-01  1.799e+02   0.315 0.753054    
## genotypeMut:TimeM2_48h00  8.996e-03  5.605e-01  1.799e+02   0.016 0.987211    
## genotypeMut:TimeM3_24h00  3.623e-04  6.607e-01  1.799e+02   0.001 0.999563    
## genotypeMut:TimeM3_48h00  4.617e-01  5.534e-01  1.799e+02   0.834 0.405205    
## ---
## Signif. codes:  0 '***' 0.001 '**' 0.01 '*' 0.05 '.' 0.1 ' ' 1
```

```
## 
## Correlation matrix not shown by default, as p = 18 > 12.
## Use print(x, correlation=TRUE)  or
##     vcov(x)        if you need it
```

```
hist(residuals(mod), nclass = 50)
```

Figure 3: Histogram of the residuals from the linear mixed models

We can compute the marginal effects of the fixed effects and their
interaction term.

```
Anova(mod)
```

```
## Analysis of Deviance Table (Type II Wald chisquare tests)
## 
## Response: dCt_NA
##                 Chisq Df Pr(>Chisq)    
## genotype       2.0673  1     0.1505    
## Time          74.0781  8  7.544e-13 ***
## genotype:Time  5.0314  8     0.7542    
## ---
## Signif. codes:  0 '***' 0.001 '**' 0.01 '*' 0.05 '.' 0.1 ' ' 1
```

We are interested in the `genotype` effect at each
timepoint.

```
emm.all <- emmeans(mod,  ~ genotype | Time)
pairs(emm.all)
```

```
## Time = iPS:
##  contrast estimate    SE  df t.ratio p.value
##  WT - Mut   0.5218 0.387 180   1.348  0.1794
## 
## Time = M1_36h00:
##  contrast estimate    SE  df t.ratio p.value
##  WT - Mut   0.2728 0.277 180   0.986  0.3255
## 
## Time = M2_06h00:
##  contrast estimate    SE  df t.ratio p.value
##  WT - Mut   0.1328 0.405 180   0.328  0.7436
## 
## Time = M2_12h00:
##  contrast estimate    SE  df t.ratio p.value
##  WT - Mut  -0.0781 0.379 180  -0.206  0.8367
## 
## Time = M2_24h00:
##  contrast estimate    SE  df t.ratio p.value
##  WT - Mut  -0.4516 0.405 180  -1.114  0.2667
## 
## Time = M2_36h00:
##  contrast estimate    SE  df t.ratio p.value
##  WT - Mut   0.3136 0.535 180   0.586  0.5588
## 
## Time = M2_48h00:
##  contrast estimate    SE  df t.ratio p.value
##  WT - Mut   0.5128 0.405 180   1.265  0.2074
## 
## Time = M3_24h00:
##  contrast estimate    SE  df t.ratio p.value
##  WT - Mut   0.5215 0.535 180   0.974  0.3314
## 
## Time = M3_48h00:
##  contrast estimate    SE  df t.ratio p.value
##  WT - Mut   0.0601 0.395 180   0.152  0.8793
## 
## Degrees-of-freedom method: kenward-roger
```

We now adjust the p-values using the Benjamini-Hochberg to identify
at which timepoints the `dCT_NA` are significantly different
between WT and Mut.

```
p <- summary(pairs(emm.all))$p.value
adj.p <- p.adjust(p, method = "BH")
names(adj.p) <- levels(data$Time)
adj.p
```

```
##       iPS  M1_36h00  M2_06h00  M2_12h00  M2_24h00  M2_36h00  M2_48h00  M3_24h00 
## 0.5965282 0.5965282 0.8793176 0.8793176 0.5965282 0.8381464 0.5965282 0.5965282 
##  M3_48h00 
## 0.8793176
```

From these p-values, we can conclude that the genotype does not
significantly impact the expression level of *FOXL2* at any
timepoint.

We can plot the marginal means estimated by the mixed model for the
`genotype`as a function of `Time`.

```
emmip(mod, genotype ~ Time)
```

Figure 4: Mean dCt predicted by the linear model as a function of time

This plot is the same as the previous one but using the actual data.
Note that this does not take into account the variability across
experiments.

```
df <- aggregate(data[, 3], by = list(data$genotype, data$Time), mean)
ggplot(df, aes(x=Group.2, y = x, group = Group.1)) + geom_line(aes(color=Group.1)) + geom_point(aes(color=Group.1))
```

Figure 5: Mean dCt computed from the actual data (across experiments) as
a function of time

# Interpretation

Overall, the genotype does not affect *FOXL2* expression at
any timepoint.

```
sessionInfo()
```

```
## R version 4.3.2 (2023-10-31)
## Platform: aarch64-apple-darwin20 (64-bit)
## Running under: macOS Sonoma 14.3.1
## 
## Matrix products: default
## BLAS:   /Library/Frameworks/R.framework/Versions/4.3-arm64/Resources/lib/libRblas.0.dylib 
## LAPACK: /Library/Frameworks/R.framework/Versions/4.3-arm64/Resources/lib/libRlapack.dylib;  LAPACK version 3.11.0
## 
## locale:
## [1] en_US.UTF-8/en_US.UTF-8/en_US.UTF-8/C/en_US.UTF-8/en_US.UTF-8
## 
## time zone: Europe/Paris
## tzcode source: internal
## 
## attached base packages:
## [1] stats     graphics  grDevices utils     datasets  methods   base     
## 
## other attached packages:
##  [1] corrplot_0.92    car_3.1-2        carData_3.0-5    ggbeeswarm_0.7.2
##  [5] emmeans_1.9.0    lmerTest_3.1-3   lme4_1.1-35.1    Matrix_1.6-5    
##  [9] kableExtra_1.3.4 lubridate_1.9.3  forcats_1.0.0    stringr_1.5.1   
## [13] dplyr_1.1.4      purrr_1.0.2      readr_2.1.5      tidyr_1.3.0     
## [17] tibble_3.2.1     ggplot2_3.4.4    tidyverse_2.0.0 
## 
## loaded via a namespace (and not attached):
##  [1] tidyselect_1.2.0    viridisLite_0.4.2   farver_2.1.1       
##  [4] vipor_0.4.7         fastmap_1.1.1       TH.data_1.1-2      
##  [7] digest_0.6.34       estimability_1.4.1  timechange_0.3.0   
## [10] lifecycle_1.0.4     survival_3.5-7      magrittr_2.0.3     
## [13] compiler_4.3.2      rlang_1.1.3         sass_0.4.8         
## [16] tools_4.3.2         utf8_1.2.4          yaml_2.3.8         
## [19] knitr_1.45          labeling_0.4.3      xml2_1.3.6         
## [22] multcomp_1.4-25     abind_1.4-5         withr_3.0.0        
## [25] numDeriv_2016.8-1.1 grid_4.3.2          fansi_1.0.6        
## [28] xtable_1.8-4        colorspace_2.1-0    scales_1.3.0       
## [31] MASS_7.3-60.0.1     cli_3.6.2           mvtnorm_1.2-4      
## [34] rmarkdown_2.25      generics_0.1.3      rstudioapi_0.15.0  
## [37] httr_1.4.7          tzdb_0.4.0          minqa_1.2.6        
## [40] cachem_1.0.8        splines_4.3.2       parallel_4.3.2     
## [43] rvest_1.0.3         vctrs_0.6.5         boot_1.3-29        
## [46] webshot_0.5.5       sandwich_3.1-0      jsonlite_1.8.8     
## [49] hms_1.1.3           pbkrtest_0.5.2      beeswarm_0.4.0     
## [52] systemfonts_1.0.5   jquerylib_0.1.4     glue_1.7.0         
## [55] nloptr_2.0.3        codetools_0.2-19    stringi_1.8.3      
## [58] gtable_0.3.4        munsell_0.5.0       pillar_1.9.0       
## [61] htmltools_0.5.7     R6_2.5.1            evaluate_0.23      
## [64] lattice_0.22-5      highr_0.10          backports_1.4.1    
## [67] broom_1.0.5         bslib_0.6.1         Rcpp_1.0.12        
## [70] svglite_2.1.3       coda_0.19-4.1       nlme_3.1-164       
## [73] xfun_0.41           zoo_1.8-12          pkgconfig_2.0.3
```
