# Supplementary material for: A conserved NR5A1-responsive enhancer regulates SRY in testis-determination
Source: Nat Commun. 2024 Mar 30;15:2796. doi: 10.1038/s41467-024-47162-2 (PMC10981742; doi:10.1038/s41467-024-47162-2)
Supplement: Supplementary file 26 — Supplementary Dataset 23 [file 41467_2024_47162_MOESM26_ESM.html]

LMM\_FGF9


Code 

- Show All Code
- Hide All Code

# LMM\_FGF9

#### Vincent Laville

#### 2024-02-23

## Data

```
data <- read.table("data_pcr_fgf9.txt", header = T, sep = "\t", dec = ".", fill = T)
data <- na.omit(data)
data$genotype <- factor(data$genotype, levels = c("WT", "Mut"))
data$Time <- factor(data$Time, 
                    levels = c("iPS", "M1_36h00", "M2_06h00", "M2_12h00", "M2_24h00", "M2_36h00", "M2_48h00", "M3_24h00", "M3_48h00"))
```

```
data %>%
  kbl() %>%
  kable_paper("hover", full_width = F) %>%
  kable_styling(bootstrap_options = c("striped", "hover")) %>%
  scroll_box(height = "300px")
```

|  | Sample\_Reference | genotype | dCt\_NA | Experiment | Time |
| --- | --- | --- | --- | --- | --- |
| 1 | 21-121 | WT | 12.46154 | iPS09 | M1\_36h00 |
| 2 | 21-122 | WT | 12.54687 | iPS09 | M1\_36h00 |
| 3 | 21-123 | WT | 11.82234 | iPS09 | M1\_36h00 |
| 4 | 21-124 | WT | 13.02666 | iPS09 | M1\_36h00 |
| 5 | 21-125 | WT | 11.69485 | iPS09 | M1\_36h00 |
| 6 | 21-126 | Mut | 11.17416 | iPS09 | M1\_36h00 |
| 7 | 21-127 | Mut | 11.65472 | iPS09 | M1\_36h00 |
| 8 | 21-128 | Mut | 12.31313 | iPS09 | M1\_36h00 |
| 9 | 21-129 | Mut | 11.03377 | iPS09 | M1\_36h00 |
| 10 | 21-130 | Mut | 10.35606 | iPS09 | M1\_36h00 |
| 11 | 22-59 | WT | 12.87990 | iPS12 | M1\_36h00 |
| 12 | 22-60 | WT | 12.49654 | iPS12 | M1\_36h00 |
| 13 | 22-61 | WT | 12.58122 | iPS12 | M1\_36h00 |
| 14 | 22-62 | WT | 12.51467 | iPS12 | M1\_36h00 |
| 15 | 22-63 | WT | 12.85105 | iPS12 | M1\_36h00 |
| 16 | 22-64 | WT | 12.36693 | iPS12 | M1\_36h00 |
| 17 | 22-65 | Mut | 11.37795 | iPS12 | M1\_36h00 |
| 18 | 22-66 | Mut | 11.40796 | iPS12 | M1\_36h00 |
| 20 | 22-68 | Mut | 11.42081 | iPS12 | M1\_36h00 |
| 22 | 22-70 | Mut | 11.36159 | iPS12 | M1\_36h00 |
| 23 | 23-181 | WT | 13.15268 | iPS26a | M1\_36h00 |
| 24 | 23-182 | WT | 12.81992 | iPS26a | M1\_36h00 |
| 25 | 23-183 | WT | 13.18746 | iPS26a | M1\_36h00 |
| 26 | 23-184 | WT | 13.04699 | iPS26a | M1\_36h00 |
| 27 | 23-185 | WT | 12.88933 | iPS26a | M1\_36h00 |
| 28 | 23-186 | WT | 13.16146 | iPS26a | M1\_36h00 |
| 29 | 23-187 | WT | 12.80593 | iPS26b | M1\_36h00 |
| 30 | 23-188 | WT | 13.15962 | iPS26b | M1\_36h00 |
| 31 | 23-189 | WT | 13.10211 | iPS26b | M1\_36h00 |
| 32 | 23-190 | WT | 13.18347 | iPS26b | M1\_36h00 |
| 33 | 23-191 | WT | 13.26135 | iPS26b | M1\_36h00 |
| 34 | 23-192 | WT | 13.01085 | iPS26b | M1\_36h00 |
| 35 | 23-193 | Mut | 12.14293 | iPS26a | M1\_36h00 |
| 36 | 23-194 | Mut | 12.27821 | iPS26a | M1\_36h00 |
| 37 | 23-195 | Mut | 12.46465 | iPS26a | M1\_36h00 |
| 38 | 23-196 | Mut | 12.46030 | iPS26a | M1\_36h00 |
| 39 | 23-197 | Mut | 12.55558 | iPS26a | M1\_36h00 |
| 40 | 23-198 | Mut | 12.13226 | iPS26a | M1\_36h00 |
| 41 | 23-199 | Mut | 12.91510 | iPS26b | M1\_36h00 |
| 42 | 23-200 | Mut | 12.79076 | iPS26b | M1\_36h00 |
| 43 | 23-201 | Mut | 12.69690 | iPS26b | M1\_36h00 |
| 44 | 23-202 | Mut | 13.05962 | iPS26b | M1\_36h00 |
| 45 | 23-203 | Mut | 12.93654 | iPS26b | M1\_36h00 |
| 46 | 23-204 | Mut | 12.63284 | iPS26b | M1\_36h00 |
| 47 | 22-71 | WT | 13.56473 | iPS12 | M2\_06h00 |
| 48 | 22-72 | WT | 13.50299 | iPS12 | M2\_06h00 |
| 50 | 22-74 | WT | 13.55734 | iPS12 | M2\_06h00 |
| 51 | 22-75 | WT | 13.38498 | iPS12 | M2\_06h00 |
| 53 | 22-77 | Mut | 11.97065 | iPS12 | M2\_06h00 |
| 54 | 22-78 | Mut | 11.81595 | iPS12 | M2\_06h00 |
| 55 | 22-79 | Mut | 11.84649 | iPS12 | M2\_06h00 |
| 56 | 22-80 | Mut | 11.93076 | iPS12 | M2\_06h00 |
| 57 | 22-81 | Mut | 12.26734 | iPS12 | M2\_06h00 |
| 58 | 22-82 | Mut | 12.24013 | iPS12 | M2\_06h00 |
| 59 | 23-205 | WT | 12.43467 | iPS26a | M2\_06h00 |
| 60 | 23-206 | WT | 12.25262 | iPS26a | M2\_06h00 |
| 61 | 23-207 | WT | 12.54939 | iPS26a | M2\_06h00 |
| 62 | 23-208 | WT | 12.26983 | iPS26b | M2\_06h00 |
| 63 | 23-209 | WT | 12.11422 | iPS26b | M2\_06h00 |
| 64 | 23-210 | WT | 12.23498 | iPS26b | M2\_06h00 |
| 65 | 23-211 | Mut | 11.98416 | iPS26a | M2\_06h00 |
| 66 | 23-212 | Mut | 12.09958 | iPS26a | M2\_06h00 |
| 67 | 23-213 | Mut | 11.70651 | iPS26a | M2\_06h00 |
| 68 | 23-214 | Mut | 12.17826 | iPS26b | M2\_06h00 |
| 69 | 23-215 | Mut | 12.16790 | iPS26b | M2\_06h00 |
| 70 | 23-216 | Mut | 12.05350 | iPS26b | M2\_06h00 |
| 71 | 22-83 | WT | 13.68977 | iPS12 | M2\_12h00 |
| 72 | 22-84 | WT | 12.88334 | iPS12 | M2\_12h00 |
| 73 | 22-85 | WT | 12.89130 | iPS12 | M2\_12h00 |
| 74 | 22-86 | WT | 13.41430 | iPS12 | M2\_12h00 |
| 75 | 22-87 | WT | 12.91940 | iPS12 | M2\_12h00 |
| 76 | 22-88 | WT | 13.12297 | iPS12 | M2\_12h00 |
| 77 | 22-89 | Mut | 11.22897 | iPS12 | M2\_12h00 |
| 78 | 22-90 | Mut | 11.13885 | iPS12 | M2\_12h00 |
| 79 | 22-91 | Mut | 11.79417 | iPS12 | M2\_12h00 |
| 80 | 22-92 | Mut | 11.09804 | iPS12 | M2\_12h00 |
| 81 | 22-93 | Mut | 11.55017 | iPS12 | M2\_12h00 |
| 82 | 22-94 | Mut | 11.25895 | iPS12 | M2\_12h00 |
| 83 | 23-217 | WT | 12.01773 | iPS26a | M2\_12h00 |
| 84 | 23-218 | WT | 11.98648 | iPS26a | M2\_12h00 |
| 85 | 23-219 | WT | 12.69373 | iPS26a | M2\_12h00 |
| 86 | 23-220 | WT | 11.80496 | iPS26b | M2\_12h00 |
| 87 | 23-221 | WT | 11.77526 | iPS26b | M2\_12h00 |
| 88 | 23-222 | WT | 11.91763 | iPS26b | M2\_12h00 |
| 89 | 23-223 | Mut | 12.18104 | iPS26a | M2\_12h00 |
| 90 | 23-224 | Mut | 12.18077 | iPS26a | M2\_12h00 |
| 91 | 23-225 | Mut | 12.10585 | iPS26a | M2\_12h00 |
| 92 | 23-226 | Mut | 11.81561 | iPS26b | M2\_12h00 |
| 93 | 23-227 | Mut | 12.16261 | iPS26b | M2\_12h00 |
| 94 | 23-228 | Mut | 11.96268 | iPS26b | M2\_12h00 |
| 95 | 21-131 | WT | 12.98639 | iPS09 | M2\_24h00 |
| 96 | 21-132 | WT | 12.27557 | iPS09 | M2\_24h00 |
| 97 | 21-133 | WT | 13.07213 | iPS09 | M2\_24h00 |
| 98 | 21-134 | WT | 12.03032 | iPS09 | M2\_24h00 |
| 99 | 21-135 | WT | 12.27773 | iPS09 | M2\_24h00 |
| 100 | 21-136 | Mut | 10.17652 | iPS09 | M2\_24h00 |
| 101 | 21-137 | Mut | 11.27970 | iPS09 | M2\_24h00 |
| 102 | 21-138 | Mut | 10.56130 | iPS09 | M2\_24h00 |
| 103 | 21-139 | Mut | 10.53302 | iPS09 | M2\_24h00 |
| 104 | 21-140 | Mut | 11.17189 | iPS09 | M2\_24h00 |
| 105 | 21-131 | WT | 12.98639 | iPS09 | M2\_24h00 |
| 106 | 21-132 | WT | 12.27557 | iPS09 | M2\_24h00 |
| 107 | 21-133 | WT | 13.07213 | iPS09 | M2\_24h00 |
| 108 | 21-134 | WT | 12.03032 | iPS09 | M2\_24h00 |
| 109 | 21-135 | WT | 12.27773 | iPS09 | M2\_24h00 |
| 110 | 21-136 | Mut | 10.17652 | iPS09 | M2\_24h00 |
| 111 | 21-137 | Mut | 11.27970 | iPS09 | M2\_24h00 |
| 112 | 21-138 | Mut | 10.56130 | iPS09 | M2\_24h00 |
| 113 | 21-139 | Mut | 10.53302 | iPS09 | M2\_24h00 |
| 114 | 21-140 | Mut | 11.17189 | iPS09 | M2\_24h00 |
| 115 | 23-229 | WT | 13.59186 | iPS26a | M2\_36h00 |
| 116 | 23-230 | WT | 13.68539 | iPS26a | M2\_36h00 |
| 117 | 23-231 | WT | 13.77563 | iPS26a | M2\_36h00 |
| 118 | 23-232 | WT | 13.06587 | iPS26b | M2\_36h00 |
| 119 | 23-233 | WT | 13.13479 | iPS26b | M2\_36h00 |
| 120 | 23-234 | WT | 13.49237 | iPS26b | M2\_36h00 |
| 121 | 23-235 | Mut | 14.13716 | iPS26a | M2\_36h00 |
| 122 | 23-236 | Mut | 13.90191 | iPS26a | M2\_36h00 |
| 123 | 23-237 | Mut | 13.81031 | iPS26a | M2\_36h00 |
| 124 | 23-238 | Mut | 13.01396 | iPS26b | M2\_36h00 |
| 125 | 23-239 | Mut | 13.23651 | iPS26b | M2\_36h00 |
| 126 | 23-240 | Mut | 13.49843 | iPS26b | M2\_36h00 |
| 127 | 22-107 | WT | 12.54905 | iPS12 | M2\_48h00 |
| 128 | 22-108 | WT | 13.24822 | iPS12 | M2\_48h00 |
| 129 | 22-109 | WT | 12.85570 | iPS12 | M2\_48h00 |
| 130 | 22-110 | WT | 12.58749 | iPS12 | M2\_48h00 |
| 131 | 22-111 | WT | 13.29151 | iPS12 | M2\_48h00 |
| 133 | 22-113 | Mut | 11.57384 | iPS12 | M2\_48h00 |
| 134 | 22-114 | Mut | 11.96141 | iPS12 | M2\_48h00 |
| 135 | 22-115 | Mut | 12.33813 | iPS12 | M2\_48h00 |
| 136 | 22-116 | Mut | 11.64191 | iPS12 | M2\_48h00 |
| 137 | 22-117 | Mut | 12.01675 | iPS12 | M2\_48h00 |
| 138 | 22-118 | Mut | 12.00895 | iPS12 | M2\_48h00 |
| 139 | 23-241 | WT | 14.18000 | iPS26a | M2\_48h00 |
| 140 | 23-242 | WT | 14.49000 | iPS26a | M2\_48h00 |
| 141 | 23-243 | WT | 14.61000 | iPS26a | M2\_48h00 |
| 142 | 23-244 | WT | 13.65000 | iPS26b | M2\_48h00 |
| 143 | 23-245 | WT | 14.22000 | iPS26b | M2\_48h00 |
| 144 | 23-246 | WT | 13.72000 | iPS26b | M2\_48h00 |
| 145 | 23-247 | Mut | 13.89000 | iPS26a | M2\_48h00 |
| 146 | 23-248 | Mut | 13.55000 | iPS26a | M2\_48h00 |
| 147 | 23-249 | Mut | 13.70000 | iPS26a | M2\_48h00 |
| 148 | 23-250 | Mut | 14.13000 | iPS26b | M2\_48h00 |
| 149 | 23-251 | Mut | 14.38000 | iPS26b | M2\_48h00 |
| 150 | 23-252 | Mut | 14.16000 | iPS26b | M2\_48h00 |
| 151 | 21-131 | WT | 12.98639 | iPS09 | M2\_24h00 |
| 152 | 21-132 | WT | 12.27557 | iPS09 | M2\_24h00 |
| 153 | 21-133 | WT | 13.07213 | iPS09 | M2\_24h00 |
| 154 | 21-134 | WT | 12.03032 | iPS09 | M2\_24h00 |
| 155 | 21-135 | WT | 12.27773 | iPS09 | M2\_24h00 |
| 156 | 21-136 | Mut | 10.17652 | iPS09 | M2\_24h00 |
| 157 | 21-137 | Mut | 11.27970 | iPS09 | M2\_24h00 |
| 158 | 21-138 | Mut | 10.56130 | iPS09 | M2\_24h00 |
| 159 | 21-139 | Mut | 10.53302 | iPS09 | M2\_24h00 |
| 160 | 21-140 | Mut | 11.17189 | iPS09 | M2\_24h00 |
| 161 | 23-253 | WT | 13.69454 | iPS26a | M3\_24h00 |
| 162 | 23-254 | WT | 13.51696 | iPS26a | M3\_24h00 |
| 163 | 23-255 | WT | 14.25398 | iPS26a | M3\_24h00 |
| 164 | 23-256 | WT | 12.10233 | iPS26b | M3\_24h00 |
| 165 | 23-257 | WT | 12.73347 | iPS26b | M3\_24h00 |
| 166 | 23-258 | WT | 11.99639 | iPS26b | M3\_24h00 |
| 167 | 23-259 | Mut | 11.79358 | iPS26a | M3\_24h00 |
| 168 | 23-260 | Mut | 11.49572 | iPS26a | M3\_24h00 |
| 169 | 23-261 | Mut | 11.67450 | iPS26a | M3\_24h00 |
| 170 | 23-262 | Mut | 11.94252 | iPS26b | M3\_24h00 |
| 171 | 23-263 | Mut | 12.14255 | iPS26b | M3\_24h00 |
| 172 | 23-264 | Mut | 12.12307 | iPS26b | M3\_24h00 |
| 173 | 21-146 | WT | 13.75476 | iPS09 | M3\_48h00 |
| 174 | 21-147 | WT | 12.86544 | iPS09 | M3\_48h00 |
| 175 | 21-148 | WT | 13.21333 | iPS09 | M3\_48h00 |
| 176 | 21-149 | WT | 13.60240 | iPS09 | M3\_48h00 |
| 177 | 21-150 | WT | 12.21891 | iPS09 | M3\_48h00 |
| 179 | 21-152 | Mut | 12.00811 | iPS09 | M3\_48h00 |
| 181 | 21-154 | Mut | 12.17217 | iPS09 | M3\_48h00 |
| 182 | 21-155 | Mut | 12.36491 | iPS09 | M3\_48h00 |
| 183 | 23-265 | WT | 12.52975 | iPS26a | M3\_48h00 |
| 184 | 23-266 | WT | 13.02921 | iPS26a | M3\_48h00 |
| 185 | 23-267 | WT | 13.43431 | iPS26a | M3\_48h00 |
| 186 | 23-268 | WT | 10.79103 | iPS26b | M3\_48h00 |
| 187 | 23-269 | WT | 10.88930 | iPS26b | M3\_48h00 |
| 188 | 23-270 | WT | 10.73752 | iPS26b | M3\_48h00 |
| 189 | 23-271 | Mut | 11.57271 | iPS26a | M3\_48h00 |
| 190 | 23-272 | Mut | 11.34341 | iPS26a | M3\_48h00 |
| 191 | 23-273 | Mut | 11.47600 | iPS26a | M3\_48h00 |
| 192 | 23-274 | Mut | 10.90606 | iPS26b | M3\_48h00 |
| 193 | 23-275 | Mut | 11.47358 | iPS26b | M3\_48h00 |
| 194 | 23-276 | Mut | 11.50285 | iPS26b | M3\_48h00 |
| 195 | 23-157 | WT | 14.62692 | iPS19 | iPS |
| 196 | 23-158 | WT | 14.53885 | iPS19 | iPS |
| 197 | 23-159 | WT | 14.30192 | iPS19 | iPS |
| 198 | 23-160 | WT | 14.82520 | iPS19 | iPS |
| 199 | 23-161 | WT | 14.59451 | iPS19 | iPS |
| 200 | 23-162 | WT | 14.32377 | iPS19 | iPS |
| 201 | 23-169 | WT | 13.96617 | iPS19 | iPS |
| 202 | 23-170 | WT | 14.13391 | iPS19 | iPS |
| 203 | 23-171 | WT | 13.90911 | iPS19 | iPS |
| 204 | 23-172 | WT | 14.53716 | iPS19 | iPS |
| 205 | 23-173 | WT | 14.19427 | iPS19 | iPS |
| 206 | 23-174 | WT | 13.80354 | iPS19 | iPS |
| 207 | 23-163 | Mut | 15.02069 | iPS19 | iPS |
| 208 | 23-164 | Mut | 15.12220 | iPS19 | iPS |
| 209 | 23-165 | Mut | 15.04068 | iPS19 | iPS |
| 210 | 23-166 | Mut | 14.46016 | iPS19 | iPS |
| 211 | 23-167 | Mut | 14.83635 | iPS19 | iPS |
| 212 | 23-168 | Mut | 14.55928 | iPS19 | iPS |
| 213 | 23-175 | Mut | 14.65537 | iPS19 | iPS |
| 214 | 23-176 | Mut | 14.53501 | iPS19 | iPS |
| 215 | 23-177 | Mut | 14.64444 | iPS19 | iPS |
| 216 | 23-178 | Mut | 14.34309 | iPS19 | iPS |
| 217 | 23-179 | Mut | 14.52701 | iPS19 | iPS |
| 218 | 23-180 | Mut | 14.55937 | iPS19 | iPS |

# Plots

We first examine the distribution of `dCt_NA` across the
different timepoints and colored by experiments.

```
ggplot(data = data, aes(x = genotype, y = dCt_NA)) +
         geom_boxplot(outlier.shape = NA) +
         geom_jitter(aes(colour = Experiment), size = 0.8) +
         theme_classic() + facet_grid(. ~ Time)
```

Figure 1: dCt (raw data) as a function of the genotype at each time
point

We next look at the distribution of `dCt_NA` across the
different experiments and colored by timepoints.

```
ggplot(data = data, aes(x = genotype, y = dCt_NA, colour = Time)) +
         geom_boxplot(outlier.shape = NA) +
         geom_jitter(position=position_jitterdodge(jitter.width = 0.1), size = 0.8) +
         theme_classic() + facet_grid(. ~ Experiment)
```

Figure 2: dCt (raw data) as a function of the genotype in each
experiment

# Analysis

We use a mixed-effect model, to analyse the dependent variable
`dCt_NA` with respect to:

- fixed effects (i.e., the `genotype` and
  `Time` variables)
- random effects (i.e., the `Experiment`
  variable)

We include an interaction term between `genotype`and
`Timepoint` as we are interested in the effect of
`genotype`at each `Timepoint` and the effect of
`genotype`seems not to be homogeneous at each timepoint.

We obtain the following:

```
mod = lmer(dCt_NA ~ genotype*Time + (1 | Experiment), data = data)

summary(mod)
```

```
## Linear mixed model fit by REML. t-tests use Satterthwaite's method [
## lmerModLmerTest]
## Formula: dCt_NA ~ genotype * Time + (1 | Experiment)
##    Data: data
## 
## REML criterion at convergence: 394.6
## 
## Scaled residuals: 
##      Min       1Q   Median       3Q      Max 
## -2.81335 -0.54455 -0.00462  0.67134  2.33625 
## 
## Random effects:
##  Groups     Name        Variance Std.Dev.
##  Experiment (Intercept) 0.03876  0.1969  
##  Residual               0.35374  0.5948  
## Number of obs: 211, groups:  Experiment, 5
## 
## Fixed effects:
##                          Estimate Std. Error       df t value Pr(>|t|)    
## (Intercept)               14.3129     0.2612   6.0375  54.791 2.24e-09 ***
## genotypeMut                0.3790     0.2428 190.3308   1.561 0.120185    
## TimeM1_36h00              -1.5303     0.3055   7.2092  -5.009 0.001420 ** 
## TimeM2_06h00              -1.5131     0.3384  10.5791  -4.471 0.001041 ** 
## TimeM2_12h00              -1.6757     0.3301   9.5674  -5.076 0.000551 ***
## TimeM2_24h00              -1.7624     0.3345   8.9060  -5.269 0.000533 ***
## TimeM2_36h00              -0.9657     0.3730  15.3123  -2.589 0.020295 *  
## TimeM2_48h00              -0.7009     0.3339  10.0185  -2.099 0.062125 .  
## TimeM3_24h00              -1.3737     0.3730  15.3123  -3.683 0.002149 ** 
## TimeM3_48h00              -1.9026     0.3337  10.0256  -5.702 0.000196 ***
## genotypeMut:TimeM1_36h00  -1.1262     0.3021 190.3980  -3.728 0.000254 ***
## genotypeMut:TimeM2_06h00  -1.1129     0.3520 190.4137  -3.161 0.001827 ** 
## genotypeMut:TimeM2_12h00  -1.2656     0.3434 190.3308  -3.686 0.000297 ***
## genotypeMut:TimeM2_24h00  -2.1630     0.3258 190.3308  -6.640 3.20e-10 ***
## genotypeMut:TimeM2_36h00  -0.2370     0.4206 190.3308  -0.563 0.573792    
## genotypeMut:TimeM2_48h00  -1.0011     0.3473 190.3485  -2.882 0.004400 ** 
## genotypeMut:TimeM3_24h00  -1.5666     0.4206 190.3308  -3.725 0.000257 ***
## genotypeMut:TimeM3_48h00  -1.2090     0.3615 190.6436  -3.344 0.000994 ***
## ---
## Signif. codes:  0 '***' 0.001 '**' 0.01 '*' 0.05 '.' 0.1 ' ' 1
```

```
## 
## Correlation matrix not shown by default, as p = 18 > 12.
## Use print(x, correlation=TRUE)  or
##     vcov(x)        if you need it
```

```
hist(residuals(mod), nclass = 50)
```

Figure 3: Histogram of the residuals from the linear mixed models

We can compute the marginal effects of the fixed effects and their
interaction term.

```
Anova(mod)
```

```
## Analysis of Deviance Table (Type II Wald chisquare tests)
## 
## Response: dCt_NA
##                 Chisq Df Pr(>Chisq)    
## genotype       83.569  1  < 2.2e-16 ***
## Time          174.091  8  < 2.2e-16 ***
## genotype:Time  53.335  8   9.29e-09 ***
## ---
## Signif. codes:  0 '***' 0.001 '**' 0.01 '*' 0.05 '.' 0.1 ' ' 1
```

We are interested in the `genotype` effect at each
timepoint.

```
emm.all <- emmeans(mod,  ~ genotype | Time)
pairs(emm.all)
```

```
## Time = iPS:
##  contrast estimate    SE  df t.ratio p.value
##  WT - Mut   -0.379 0.243 190  -1.561  0.1202
## 
## Time = M1_36h00:
##  contrast estimate    SE  df t.ratio p.value
##  WT - Mut    0.747 0.180 190   4.157  <.0001
## 
## Time = M2_06h00:
##  contrast estimate    SE  df t.ratio p.value
##  WT - Mut    0.734 0.255 190   2.878  0.0045
## 
## Time = M2_12h00:
##  contrast estimate    SE  df t.ratio p.value
##  WT - Mut    0.887 0.243 190   3.651  0.0003
## 
## Time = M2_24h00:
##  contrast estimate    SE  df t.ratio p.value
##  WT - Mut    1.784 0.217 190   8.214  <.0001
## 
## Time = M2_36h00:
##  contrast estimate    SE  df t.ratio p.value
##  WT - Mut   -0.142 0.343 190  -0.414  0.6796
## 
## Time = M2_48h00:
##  contrast estimate    SE  df t.ratio p.value
##  WT - Mut    0.622 0.248 190   2.505  0.0131
## 
## Time = M3_24h00:
##  contrast estimate    SE  df t.ratio p.value
##  WT - Mut    1.188 0.343 190   3.459  0.0007
## 
## Time = M3_48h00:
##  contrast estimate    SE  df t.ratio p.value
##  WT - Mut    0.830 0.268 191   3.096  0.0023
## 
## Degrees-of-freedom method: kenward-roger
```

We now adjust the p-values using the Benjamini-Hochberg to identify
at which timepoints the `dCT_NA` are significantly different
between WT and Mut.

```
p <- summary(pairs(emm.all))$p.value
adj.p <- p.adjust(p, method = "BH")
names(adj.p) <- levels(data$Time)
adj.p
```

```
##          iPS     M1_36h00     M2_06h00     M2_12h00     M2_24h00     M2_36h00 
## 1.352110e-01 2.194071e-04 6.683199e-03 1.011306e-03 2.900252e-13 6.795518e-01 
##     M2_48h00     M3_24h00     M3_48h00 
## 1.683259e-02 1.508062e-03 4.068326e-03
```

From these p-values, we can conclude that the genotype does not
significantly impact the expression level of *FGF9* at any
timepoint.

We can plot the marginal means estimated by the mixed model for the
`genotype`as a function of `Time`.

```
emmip(mod, genotype ~ Time)
```

Figure 4: Mean dCt predicted by the linear model as a function of time

This plot is the same as the previous one but using the actual data.
Note that this does not take into account the variability across
experiments.

```
df <- aggregate(data[, 3], by = list(data$genotype, data$Time), mean)
ggplot(df, aes(x=Group.2, y = x, group = Group.1)) + geom_line(aes(color=Group.1)) + geom_point(aes(color=Group.1))
```

Figure 5: Mean dCt computed from the actual data (across experiments) as
a function of time

# Interpretation

Overall, the genotype does affect FGF9 expression at every
differentiation time point.

```
sessionInfo()
```

```
## R version 4.3.2 (2023-10-31)
## Platform: aarch64-apple-darwin20 (64-bit)
## Running under: macOS Sonoma 14.3.1
## 
## Matrix products: default
## BLAS:   /Library/Frameworks/R.framework/Versions/4.3-arm64/Resources/lib/libRblas.0.dylib 
## LAPACK: /Library/Frameworks/R.framework/Versions/4.3-arm64/Resources/lib/libRlapack.dylib;  LAPACK version 3.11.0
## 
## locale:
## [1] en_US.UTF-8/en_US.UTF-8/en_US.UTF-8/C/en_US.UTF-8/en_US.UTF-8
## 
## time zone: Europe/Paris
## tzcode source: internal
## 
## attached base packages:
## [1] stats     graphics  grDevices utils     datasets  methods   base     
## 
## other attached packages:
##  [1] corrplot_0.92    car_3.1-2        carData_3.0-5    ggbeeswarm_0.7.2
##  [5] emmeans_1.9.0    lmerTest_3.1-3   lme4_1.1-35.1    Matrix_1.6-5    
##  [9] kableExtra_1.3.4 lubridate_1.9.3  forcats_1.0.0    stringr_1.5.1   
## [13] dplyr_1.1.4      purrr_1.0.2      readr_2.1.5      tidyr_1.3.0     
## [17] tibble_3.2.1     ggplot2_3.4.4    tidyverse_2.0.0 
## 
## loaded via a namespace (and not attached):
##  [1] tidyselect_1.2.0    viridisLite_0.4.2   farver_2.1.1       
##  [4] vipor_0.4.7         fastmap_1.1.1       TH.data_1.1-2      
##  [7] digest_0.6.34       estimability_1.4.1  timechange_0.3.0   
## [10] lifecycle_1.0.4     survival_3.5-7      magrittr_2.0.3     
## [13] compiler_4.3.2      rlang_1.1.3         sass_0.4.8         
## [16] tools_4.3.2         utf8_1.2.4          yaml_2.3.8         
## [19] knitr_1.45          labeling_0.4.3      xml2_1.3.6         
## [22] multcomp_1.4-25     abind_1.4-5         withr_3.0.0        
## [25] numDeriv_2016.8-1.1 grid_4.3.2          fansi_1.0.6        
## [28] xtable_1.8-4        colorspace_2.1-0    scales_1.3.0       
## [31] MASS_7.3-60.0.1     cli_3.6.2           mvtnorm_1.2-4      
## [34] rmarkdown_2.25      generics_0.1.3      rstudioapi_0.15.0  
## [37] httr_1.4.7          tzdb_0.4.0          minqa_1.2.6        
## [40] cachem_1.0.8        splines_4.3.2       parallel_4.3.2     
## [43] rvest_1.0.3         vctrs_0.6.5         boot_1.3-29        
## [46] webshot_0.5.5       sandwich_3.1-0      jsonlite_1.8.8     
## [49] hms_1.1.3           pbkrtest_0.5.2      beeswarm_0.4.0     
## [52] systemfonts_1.0.5   jquerylib_0.1.4     glue_1.7.0         
## [55] nloptr_2.0.3        codetools_0.2-19    stringi_1.8.3      
## [58] gtable_0.3.4        munsell_0.5.0       pillar_1.9.0       
## [61] htmltools_0.5.7     R6_2.5.1            evaluate_0.23      
## [64] lattice_0.22-5      highr_0.10          backports_1.4.1    
## [67] broom_1.0.5         bslib_0.6.1         Rcpp_1.0.12        
## [70] svglite_2.1.3       coda_0.19-4.1       nlme_3.1-164       
## [73] xfun_0.41           zoo_1.8-12          pkgconfig_2.0.3
```
