# Supplementary material for: A conserved NR5A1-responsive enhancer regulates SRY in testis-determination
Source: Nat Commun. 2024 Mar 30;15:2796. doi: 10.1038/s41467-024-47162-2 (PMC10981742; doi:10.1038/s41467-024-47162-2)
Supplement: Supplementary file 33 — Supplementary Dataset 30 [file 41467_2024_47162_MOESM33_ESM.html]

Supplementary\_Data\_30


# Supplementary\_Data\_30

#### Denis

#### 2023-07-10

RT-qPCR analysis - SRY - outlier identification by the IQR method in
R

```
rm(list=ls())
```

```
library(tidyverse)
```

```
## ── Attaching core tidyverse packages ──────────────────────── tidyverse 2.0.0 ──
## ✔ dplyr     1.1.4     ✔ readr     2.1.5
## ✔ forcats   1.0.0     ✔ stringr   1.5.1
## ✔ ggplot2   3.4.4     ✔ tibble    3.2.1
## ✔ lubridate 1.9.3     ✔ tidyr     1.3.0
## ✔ purrr     1.0.2     
## ── Conflicts ────────────────────────────────────────── tidyverse_conflicts() ──
## ✖ dplyr::filter() masks stats::filter()
## ✖ dplyr::lag()    masks stats::lag()
## ℹ Use the conflicted package (<http://conflicted.r-lib.org/>) to force all conflicts to become errors
```

# 1 SRY

## 1.1 SRY\_iPS07

### 1.1.1 SRY\_iPS07-M1\_36h00-Mut

```
Input = ("
names   values  block
Mut 16.31   iPS07-82-M1-1
Mut 16.43   iPS07-82-M1-2
Mut 16.09   iPS07-82-M1-3
Mut 15.44   iPS07-82-M1-4
Mut 15.63   iPS07-82-M1-5"
)
Data = read.table(textConnection(Input),header=TRUE)
Data$names = factor(Data$names,ordered=FALSE, levels=unique(Data$names))
Data$block = factor(Data$block,ordered=FALSE, levels=unique(Data$block))

# SRY_boxplot 1
boxplot(values ~ names,
        data = Data,
        ylab ="values",
        xlab ="names")
```

```
Data1 <- Data |>
  mutate(
    IQR = IQR(values, na.rm = TRUE),
    Outlier_upper = quantile(values, probs = c(.75), na.rm = TRUE) + 1.5 * IQR,
    Outlier_lower = quantile(values, probs = c(.25), na.rm = TRUE) - 1.5 * IQR,
    values_wo_outliers = if_else(values <= Outlier_lower | values >= Outlier_upper, NA, values))

boxplot(values_wo_outliers ~ names, Data1)
```

```
Data1b<- Data1|> select(block, names, values, values_wo_outliers)
Data1b
```

```
##           block names values values_wo_outliers
## 1 iPS07-82-M1-1   Mut  16.31              16.31
## 2 iPS07-82-M1-2   Mut  16.43              16.43
## 3 iPS07-82-M1-3   Mut  16.09              16.09
## 4 iPS07-82-M1-4   Mut  15.44              15.44
## 5 iPS07-82-M1-5   Mut  15.63              15.63
```

### 1.1.2 SRY\_iPS07-M2\_48h00-Wt

```
Input = ("
names   values  block
WT  13.38   iPS07-45-M2_48
WT  14.10   iPS07-45-M2_48
WT  13.37   iPS07-45-M2_48
WT  13.60   iPS07-45-M2_48
WT  13.82   iPS07-45-M2_48
"
)
Data = read.table(textConnection(Input),header=TRUE)
Data$names = factor(Data$names,ordered=FALSE, levels=unique(Data$names))
Data$block = factor(Data$block,ordered=FALSE, levels=unique(Data$block))

# SRY_boxplot 1
boxplot(values ~ names,
        data = Data,
        ylab ="values",
        xlab ="names")
```

```
Data2 <- Data |>
  mutate(
    IQR = IQR(values, na.rm = TRUE),
    Outlier_upper = quantile(values, probs = c(.75), na.rm = TRUE) + 1.5 * IQR,
    Outlier_lower = quantile(values, probs = c(.25), na.rm = TRUE) - 1.5 * IQR,
    values_wo_outliers = if_else(values <= Outlier_lower | values >= Outlier_upper, NA, values))

boxplot(values_wo_outliers ~ names, Data2)
```

```
Data2b<- Data2|> select(block, names, values, values_wo_outliers)
Data2b
```

```
##            block names values values_wo_outliers
## 1 iPS07-45-M2_48    WT  13.38              13.38
## 2 iPS07-45-M2_48    WT  14.10              14.10
## 3 iPS07-45-M2_48    WT  13.37              13.37
## 4 iPS07-45-M2_48    WT  13.60              13.60
## 5 iPS07-45-M2_48    WT  13.82              13.82
```

### 1.1.3 SRY\_iPS07-M2\_48h00-Mut

```
Input = ("
names   values  block
Mut 13.48   iPS07-82-M2_48
Mut 13.53   iPS07-82-M2_48
Mut 13.46   iPS07-82-M2_48
Mut 13.82   iPS07-82-M2_48
Mut 13.53   iPS07-82-M2_48
"
)
Data = read.table(textConnection(Input),header=TRUE)
Data$names = factor(Data$names,ordered=FALSE, levels=unique(Data$names))
Data$block = factor(Data$block,ordered=FALSE, levels=unique(Data$block))

# SRY_boxplot 1
boxplot(values ~ names,
        data = Data,
        ylab ="values",
        xlab ="names")
```

```
Data3 <- Data |>
  mutate(
    IQR = IQR(values, na.rm = TRUE),
    Outlier_upper = quantile(values, probs = c(.75), na.rm = TRUE) + 1.5 * IQR,
    Outlier_lower = quantile(values, probs = c(.25), na.rm = TRUE) - 1.5 * IQR,
    values_wo_outliers = if_else(values <= Outlier_lower | values >= Outlier_upper, NA, values))

boxplot(values_wo_outliers ~ names, Data3)
```

```
Data3b<- Data3|> select(block, names, values, values_wo_outliers)
Data3b
```

```
##            block names values values_wo_outliers
## 1 iPS07-82-M2_48   Mut  13.48              13.48
## 2 iPS07-82-M2_48   Mut  13.53              13.53
## 3 iPS07-82-M2_48   Mut  13.46              13.46
## 4 iPS07-82-M2_48   Mut  13.82                 NA
## 5 iPS07-82-M2_48   Mut  13.53              13.53
```

## 1.2 SRY\_iPS09

### 1.2.1 SRY\_iPS09-M1\_36h00-Wt

```
Input = ("
names   values  block
WT  11.57   iPS09-45-M1_36h00
WT  11.98   iPS09-45-M1_36h00
WT  12.69   iPS09-45-M1_36h00
WT  12.18   iPS09-45-M1_36h00
WT  11.37   iPS09-45-M1_36h00
"
)
Data = read.table(textConnection(Input),header=TRUE)
Data$names = factor(Data$names,ordered=FALSE, levels=unique(Data$names))
Data$block = factor(Data$block,ordered=FALSE, levels=unique(Data$block))

# SRY_boxplot 1
boxplot(values ~ names,
        data = Data,
        ylab ="values",
        xlab ="names")
```

```
Data4 <- Data |>
  mutate(
    IQR = IQR(values, na.rm = TRUE),
    Outlier_upper = quantile(values, probs = c(.75), na.rm = TRUE) + 1.5 * IQR,
    Outlier_lower = quantile(values, probs = c(.25), na.rm = TRUE) - 1.5 * IQR,
    values_wo_outliers = if_else(values <= Outlier_lower | values >= Outlier_upper, NA, values))

boxplot(values_wo_outliers ~ names, Data4)
```

```
Data4b<- Data4|> select(block, names, values, values_wo_outliers)
Data4b
```

```
##               block names values values_wo_outliers
## 1 iPS09-45-M1_36h00    WT  11.57              11.57
## 2 iPS09-45-M1_36h00    WT  11.98              11.98
## 3 iPS09-45-M1_36h00    WT  12.69              12.69
## 4 iPS09-45-M1_36h00    WT  12.18              12.18
## 5 iPS09-45-M1_36h00    WT  11.37              11.37
```

### 1.2.2 SRY\_iPS09-M1\_36h00-Mut

```
Input = ("
names   values  block
Mut 11.65   iPS09-82-M1_36h00
Mut 12.56   iPS09-82-M1_36h00
Mut 12.90   iPS09-82-M1_36h00
Mut 12.60   iPS09-82-M1_36h00
Mut 12.56   iPS09-82-M1_36h00
"
)
Data = read.table(textConnection(Input),header=TRUE)
Data$names = factor(Data$names,ordered=FALSE, levels=unique(Data$names))
Data$block = factor(Data$block,ordered=FALSE, levels=unique(Data$block))

# SRY_boxplot 1
boxplot(values ~ names,
        data = Data,
        ylab ="values",
        xlab ="names")
```

```
Data5 <- Data |>
  mutate(
    IQR = IQR(values, na.rm = TRUE),
    Outlier_upper = quantile(values, probs = c(.75), na.rm = TRUE) + 1.5 * IQR,
    Outlier_lower = quantile(values, probs = c(.25), na.rm = TRUE) - 1.5 * IQR,
    values_wo_outliers = if_else(values <= Outlier_lower | values >= Outlier_upper, NA, values))

boxplot(values_wo_outliers ~ names, Data5)
```

```
Data5b<- Data5|> select(block, names, values, values_wo_outliers)
Data5b
```

```
##               block names values values_wo_outliers
## 1 iPS09-82-M1_36h00   Mut  11.65                 NA
## 2 iPS09-82-M1_36h00   Mut  12.56              12.56
## 3 iPS09-82-M1_36h00   Mut  12.90                 NA
## 4 iPS09-82-M1_36h00   Mut  12.60              12.60
## 5 iPS09-82-M1_36h00   Mut  12.56              12.56
```

### 1.2.3 SRY\_iPS09-M2\_24h00-Wt

```
Input = ("
names   values  block
WT  9.22    iPS09-45-M2_24h00
WT  8.91    iPS09-45-M2_24h00
WT  9.16    iPS09-45-M2_24h00
WT  9.07    iPS09-45-M2_24h00
WT  8.97    iPS09-45-M2_24h00
"
)
Data = read.table(textConnection(Input),header=TRUE)
Data$names = factor(Data$names,ordered=FALSE, levels=unique(Data$names))
Data$block = factor(Data$block,ordered=FALSE, levels=unique(Data$block))

# SRY_boxplot 1
boxplot(values ~ names,
        data = Data,
        ylab ="values",
        xlab ="names")
```

```
Data6 <- Data |>
  mutate(
    IQR = IQR(values, na.rm = TRUE),
    Outlier_upper = quantile(values, probs = c(.75), na.rm = TRUE) + 1.5 * IQR,
    Outlier_lower = quantile(values, probs = c(.25), na.rm = TRUE) - 1.5 * IQR,
    values_wo_outliers = if_else(values <= Outlier_lower | values >= Outlier_upper, NA, values))

boxplot(values_wo_outliers ~ names, Data6)
```

```
Data6b<- Data6|> select(block, names, values, values_wo_outliers)
Data6b
```

```
##               block names values values_wo_outliers
## 1 iPS09-45-M2_24h00    WT   9.22               9.22
## 2 iPS09-45-M2_24h00    WT   8.91               8.91
## 3 iPS09-45-M2_24h00    WT   9.16               9.16
## 4 iPS09-45-M2_24h00    WT   9.07               9.07
## 5 iPS09-45-M2_24h00    WT   8.97               8.97
```

### 1.2.4 SRY\_iPS09-M2\_24h00-Mut

```
Input = ("
names   values  block
Mut 8.82    iPS09-82-M2_24h00
Mut 9.13    iPS09-82-M2_24h00
Mut 8.85    iPS09-82-M2_24h00
Mut 8.85    iPS09-82-M2_24h00
Mut 8.76    iPS09-82-M2_24h00
"
)
Data = read.table(textConnection(Input),header=TRUE)
Data$names = factor(Data$names,ordered=FALSE, levels=unique(Data$names))
Data$block = factor(Data$block,ordered=FALSE, levels=unique(Data$block))

# SRY_boxplot 1
boxplot(values ~ names,
        data = Data,
        ylab ="values",
        xlab ="names")
```

```
Data7 <- Data |>
  mutate(
    IQR = IQR(values, na.rm = TRUE),
    Outlier_upper = quantile(values, probs = c(.75), na.rm = TRUE) + 1.5 * IQR,
    Outlier_lower = quantile(values, probs = c(.25), na.rm = TRUE) - 1.5 * IQR,
    values_wo_outliers = if_else(values <= Outlier_lower | values >= Outlier_upper, NA, values))

boxplot(values_wo_outliers ~ names, Data7)
```

```
Data7b<- Data7|> select(block, names, values, values_wo_outliers)
Data7b
```

```
##               block names values values_wo_outliers
## 1 iPS09-82-M2_24h00   Mut   8.82               8.82
## 2 iPS09-82-M2_24h00   Mut   9.13                 NA
## 3 iPS09-82-M2_24h00   Mut   8.85               8.85
## 4 iPS09-82-M2_24h00   Mut   8.85               8.85
## 5 iPS09-82-M2_24h00   Mut   8.76                 NA
```

### 1.2.5 SRY\_iPS09-M3\_24h00-Wt

```
Input = ("
names   values  block
WT  12.28   iPS09-45-M3_24h00
WT  12.69   iPS09-45-M3_24h00
WT  12.39   iPS09-45-M3_24h00
WT  12.55   iPS09-45-M3_24h00
WT  12.40   iPS09-45-M3_24h00
"
)
Data = read.table(textConnection(Input),header=TRUE)
Data$names = factor(Data$names,ordered=FALSE, levels=unique(Data$names))
Data$block = factor(Data$block,ordered=FALSE, levels=unique(Data$block))

# SRY_boxplot 1
boxplot(values ~ names,
        data = Data,
        ylab ="values",
        xlab ="names")
```

```
Data8 <- Data |>
  mutate(
    IQR = IQR(values, na.rm = TRUE),
    Outlier_upper = quantile(values, probs = c(.75), na.rm = TRUE) + 1.5 * IQR,
    Outlier_lower = quantile(values, probs = c(.25), na.rm = TRUE) - 1.5 * IQR,
    values_wo_outliers = if_else(values <= Outlier_lower | values >= Outlier_upper, NA, values))

boxplot(values_wo_outliers ~ names, Data8)
```

```
Data8b<- Data8|> select(block, names, values, values_wo_outliers)
Data8b
```

```
##               block names values values_wo_outliers
## 1 iPS09-45-M3_24h00    WT  12.28              12.28
## 2 iPS09-45-M3_24h00    WT  12.69              12.69
## 3 iPS09-45-M3_24h00    WT  12.39              12.39
## 4 iPS09-45-M3_24h00    WT  12.55              12.55
## 5 iPS09-45-M3_24h00    WT  12.40              12.40
```

### 1.2.6 SRY\_iPS09-M3\_48h00-Wt

```
Input = ("
names   values  block
WT  13.51   iPS09-45-M3_48h00
WT  13.19   iPS09-45-M3_48h00
WT  14.25   iPS09-45-M3_48h00
WT  14.45   iPS09-45-M3_48h00
WT  13.71   iPS09-45-M3_48h00
"
)
Data = read.table(textConnection(Input),header=TRUE)
Data$names = factor(Data$names,ordered=FALSE, levels=unique(Data$names))
Data$block = factor(Data$block,ordered=FALSE, levels=unique(Data$block))

# SRY_boxplot 1
boxplot(values ~ names,
        data = Data,
        ylab ="values",
        xlab ="names")
```

```
Data9 <- Data |>
  mutate(
    IQR = IQR(values, na.rm = TRUE),
    Outlier_upper = quantile(values, probs = c(.75), na.rm = TRUE) + 1.5 * IQR,
    Outlier_lower = quantile(values, probs = c(.25), na.rm = TRUE) - 1.5 * IQR,
    values_wo_outliers = if_else(values <= Outlier_lower | values >= Outlier_upper, NA, values))

boxplot(values_wo_outliers ~ names, Data9)
```

```
Data9b<- Data9|> select(block, names, values, values_wo_outliers)
Data9b
```

```
##               block names values values_wo_outliers
## 1 iPS09-45-M3_48h00    WT  13.51              13.51
## 2 iPS09-45-M3_48h00    WT  13.19              13.19
## 3 iPS09-45-M3_48h00    WT  14.25              14.25
## 4 iPS09-45-M3_48h00    WT  14.45              14.45
## 5 iPS09-45-M3_48h00    WT  13.71              13.71
```

### 1.2.7 SRY\_iPS09-M3\_48h00-Mut

```
Input = ("
names   values  block
Mut 14.14   iPS09-82-M3_48h00
Mut 13.56   iPS09-82-M3_48h00
Mut 13.31   iPS09-82-M3_48h00
Mut 14.14   iPS09-82-M3_48h00
Mut 13.74   iPS09-82-M3_48h00
"
)
Data = read.table(textConnection(Input),header=TRUE)
Data$names = factor(Data$names,ordered=FALSE, levels=unique(Data$names))
Data$block = factor(Data$block,ordered=FALSE, levels=unique(Data$block))

# SRY_boxplot 1
boxplot(values ~ names,
        data = Data,
        ylab ="values",
        xlab ="names")
```

```
Data10 <- Data |>
  mutate(
    IQR = IQR(values, na.rm = TRUE),
    Outlier_upper = quantile(values, probs = c(.75), na.rm = TRUE) + 1.5 * IQR,
    Outlier_lower = quantile(values, probs = c(.25), na.rm = TRUE) - 1.5 * IQR,
    values_wo_outliers = if_else(values <= Outlier_lower | values >= Outlier_upper, NA, values))

boxplot(values_wo_outliers ~ names, Data10)
```

```
Data10b<- Data10|> select(block, names, values, values_wo_outliers)
Data10b
```

```
##               block names values values_wo_outliers
## 1 iPS09-82-M3_48h00   Mut  14.14              14.14
## 2 iPS09-82-M3_48h00   Mut  13.56              13.56
## 3 iPS09-82-M3_48h00   Mut  13.31              13.31
## 4 iPS09-82-M3_48h00   Mut  14.14              14.14
## 5 iPS09-82-M3_48h00   Mut  13.74              13.74
```

### 1.2.8 SRY\_iPS09-iPS-Wt

```
Input = ("
names   values  block
WT  11.20   iPS07-45-iPS
WT  12.53   iPS07-45-iPS
WT  13.02   iPS07-45-iPS
WT  12.29   iPS07-45-iPS
WT  11.50   iPS07-45-iPS
WT  12.32   iPS07-45-iPS
WT  11.81   iPS07-45-iPS
"
)
Data = read.table(textConnection(Input),header=TRUE)
Data$names = factor(Data$names,ordered=FALSE, levels=unique(Data$names))
Data$block = factor(Data$block,ordered=FALSE, levels=unique(Data$block))

# SRY_boxplot 1
boxplot(values ~ names,
        data = Data,
        ylab ="values",
        xlab ="names")
```

```
Data11 <- Data |>
  mutate(
    IQR = IQR(values, na.rm = TRUE),
    Outlier_upper = quantile(values, probs = c(.75), na.rm = TRUE) + 1.5 * IQR,
    Outlier_lower = quantile(values, probs = c(.25), na.rm = TRUE) - 1.5 * IQR,
    values_wo_outliers = if_else(values <= Outlier_lower | values >= Outlier_upper, NA, values))

boxplot(values_wo_outliers ~ names, Data11)
```

```
Data11b<- Data11|> select(block, names, values, values_wo_outliers)
Data11b
```

```
##          block names values values_wo_outliers
## 1 iPS07-45-iPS    WT  11.20              11.20
## 2 iPS07-45-iPS    WT  12.53              12.53
## 3 iPS07-45-iPS    WT  13.02              13.02
## 4 iPS07-45-iPS    WT  12.29              12.29
## 5 iPS07-45-iPS    WT  11.50              11.50
## 6 iPS07-45-iPS    WT  12.32              12.32
## 7 iPS07-45-iPS    WT  11.81              11.81
```

### 1.2.9 SRY\_iPS09-iPS-Mut

```
Input = ("
names   values  block
Mut 8.65    iPS09-82-iPS
Mut 9.23    iPS09-82-iPS
Mut 8.22    iPS09-82-iPS
Mut 8.16    iPS09-82-iPS
Mut 9.15    iPS09-82-iPS
Mut 9.13    iPS09-82-iPS
"
)
Data = read.table(textConnection(Input),header=TRUE)
Data$names = factor(Data$names,ordered=FALSE, levels=unique(Data$names))
Data$block = factor(Data$block,ordered=FALSE, levels=unique(Data$block))

# SRY_boxplot 1
boxplot(values ~ names,
        data = Data,
        ylab ="values",
        xlab ="names")
```

```
Data12 <- Data |>
  mutate(
    IQR = IQR(values, na.rm = TRUE),
    Outlier_upper = quantile(values, probs = c(.75), na.rm = TRUE) + 1.5 * IQR,
    Outlier_lower = quantile(values, probs = c(.25), na.rm = TRUE) - 1.5 * IQR,
    values_wo_outliers = if_else(values <= Outlier_lower | values >= Outlier_upper, NA, values))

boxplot(values_wo_outliers ~ names, Data12)
```

```
Data12b<- Data12|> select(block, names, values, values_wo_outliers)
Data12b
```

```
##          block names values values_wo_outliers
## 1 iPS09-82-iPS   Mut   8.65               8.65
## 2 iPS09-82-iPS   Mut   9.23               9.23
## 3 iPS09-82-iPS   Mut   8.22               8.22
## 4 iPS09-82-iPS   Mut   8.16               8.16
## 5 iPS09-82-iPS   Mut   9.15               9.15
## 6 iPS09-82-iPS   Mut   9.13               9.13
```

## 1.3 SRY\_iPS12

### 1.3.1 SRY\_iPS12-M1\_36h00-Wt

```
Input = ("
names   values  block
WT  11.93   iPS12_45_M1_36_P
WT  12.47   iPS12_45_M1_36_P
WT  11.89   iPS12_45_M1_36_P
WT  12.46   iPS12_45_M1_36_P
WT  12.27   iPS12_45_M1_36_P
WT  12.06   iPS12_45_M1_36_P
"
)
Data = read.table(textConnection(Input),header=TRUE)
Data$names = factor(Data$names,ordered=FALSE, levels=unique(Data$names))
Data$block = factor(Data$block,ordered=FALSE, levels=unique(Data$block))

# SRY_boxplot 1
boxplot(values ~ names,
        data = Data,
        ylab ="values",
        xlab ="names")
```

```
Data13 <- Data |>
  mutate(
    IQR = IQR(values, na.rm = TRUE),
    Outlier_upper = quantile(values, probs = c(.75), na.rm = TRUE) + 1.5 * IQR,
    Outlier_lower = quantile(values, probs = c(.25), na.rm = TRUE) - 1.5 * IQR,
    values_wo_outliers = if_else(values <= Outlier_lower | values >= Outlier_upper, NA, values))

boxplot(values_wo_outliers ~ names, Data13)
```

```
Data13b<- Data13|> select(block, names, values, values_wo_outliers)
Data13b
```

```
##              block names values values_wo_outliers
## 1 iPS12_45_M1_36_P    WT  11.93              11.93
## 2 iPS12_45_M1_36_P    WT  12.47              12.47
## 3 iPS12_45_M1_36_P    WT  11.89              11.89
## 4 iPS12_45_M1_36_P    WT  12.46              12.46
## 5 iPS12_45_M1_36_P    WT  12.27              12.27
## 6 iPS12_45_M1_36_P    WT  12.06              12.06
```

### 1.3.2 SRY\_iPS12-M1\_36h00-Mut

```
Input = ("
names   values  block
Mut 13.10   iPS12_82_M1_36_P
Mut 12.99   iPS12_82_M1_36_P
Mut 13.43   iPS12_82_M1_36_P
Mut 13.29   iPS12_82_M1_36_P
Mut 13.25   iPS12_82_M1_36_P
Mut 13.26   iPS12_82_M1_36_P
"
)
Data = read.table(textConnection(Input),header=TRUE)
Data$names = factor(Data$names,ordered=FALSE, levels=unique(Data$names))
Data$block = factor(Data$block,ordered=FALSE, levels=unique(Data$block))

# SRY_boxplot 1
boxplot(values ~ names,
        data = Data,
        ylab ="values",
        xlab ="names")
```

```
Data14 <- Data |>
  mutate(
    IQR = IQR(values, na.rm = TRUE),
    Outlier_upper = quantile(values, probs = c(.75), na.rm = TRUE) + 1.5 * IQR,
    Outlier_lower = quantile(values, probs = c(.25), na.rm = TRUE) - 1.5 * IQR,
    values_wo_outliers = if_else(values <= Outlier_lower | values >= Outlier_upper, NA, values))

boxplot(values_wo_outliers ~ names, Data14)
```

```
Data14b<- Data14|> select(block, names, values, values_wo_outliers)
Data14b
```

```
##              block names values values_wo_outliers
## 1 iPS12_82_M1_36_P   Mut  13.10              13.10
## 2 iPS12_82_M1_36_P   Mut  12.99              12.99
## 3 iPS12_82_M1_36_P   Mut  13.43              13.43
## 4 iPS12_82_M1_36_P   Mut  13.29              13.29
## 5 iPS12_82_M1_36_P   Mut  13.25              13.25
## 6 iPS12_82_M1_36_P   Mut  13.26              13.26
```

### 1.3.3 SRY\_iPS12-M2\_06h00-Wt

```
Input = ("
names   values  block
WT  8.01    iPS12_45_M2_06_P
WT  8.29    iPS12_45_M2_06_P
WT  8.23    iPS12_45_M2_06_P
WT  8.47    iPS12_45_M2_06_P
WT  7.89    iPS12_45_M2_06_P
WT  NA  iPS12_45_M2_06_P
"
)
Data = read.table(textConnection(Input),header=TRUE)
Data$names = factor(Data$names,ordered=FALSE, levels=unique(Data$names))
Data$block = factor(Data$block,ordered=FALSE, levels=unique(Data$block))

# SRY_boxplot 1
boxplot(values ~ names,
        data = Data,
        ylab ="values",
        xlab ="names")
```

```
Data15 <- Data |>
  mutate(
    IQR = IQR(values, na.rm = TRUE),
    Outlier_upper = quantile(values, probs = c(.75), na.rm = TRUE) + 1.5 * IQR,
    Outlier_lower = quantile(values, probs = c(.25), na.rm = TRUE) - 1.5 * IQR,
    values_wo_outliers = if_else(values <= Outlier_lower | values >= Outlier_upper, NA, values))

boxplot(values_wo_outliers ~ names, Data15)
```

```
Data15b<- Data15|> select(block, names, values, values_wo_outliers)
Data15b
```

```
##              block names values values_wo_outliers
## 1 iPS12_45_M2_06_P    WT   8.01               8.01
## 2 iPS12_45_M2_06_P    WT   8.29               8.29
## 3 iPS12_45_M2_06_P    WT   8.23               8.23
## 4 iPS12_45_M2_06_P    WT   8.47               8.47
## 5 iPS12_45_M2_06_P    WT   7.89               7.89
## 6 iPS12_45_M2_06_P    WT     NA                 NA
```

### 1.3.4 SRY\_iPS12-M2\_06h00-Mut

```
Input = ("
names   values  block
Mut 5.46    iPS12_82_M2_06_P
Mut 9.20    iPS12_82_M2_06_P
Mut 9.17    iPS12_82_M2_06_P
Mut 8.84    iPS12_82_M2_06_P
Mut 9.09    iPS12_82_M2_06_P
Mut 8.58    iPS12_82_M2_06_P
"
)
Data = read.table(textConnection(Input),header=TRUE)
Data$names = factor(Data$names,ordered=FALSE, levels=unique(Data$names))
Data$block = factor(Data$block,ordered=FALSE, levels=unique(Data$block))

# SRY_boxplot 1
boxplot(values ~ names,
        data = Data,
        ylab ="values",
        xlab ="names")
```

```
Data16 <- Data |>
  mutate(
    IQR = IQR(values, na.rm = TRUE),
    Outlier_upper = quantile(values, probs = c(.75), na.rm = TRUE) + 1.5 * IQR,
    Outlier_lower = quantile(values, probs = c(.25), na.rm = TRUE) - 1.5 * IQR,
    values_wo_outliers = if_else(values <= Outlier_lower | values >= Outlier_upper, NA, values))

boxplot(values_wo_outliers ~ names, Data16)
```

```
Data16b<- Data16|> select(block, names, values, values_wo_outliers)
Data16b
```

```
##              block names values values_wo_outliers
## 1 iPS12_82_M2_06_P   Mut   5.46                 NA
## 2 iPS12_82_M2_06_P   Mut   9.20               9.20
## 3 iPS12_82_M2_06_P   Mut   9.17               9.17
## 4 iPS12_82_M2_06_P   Mut   8.84               8.84
## 5 iPS12_82_M2_06_P   Mut   9.09               9.09
## 6 iPS12_82_M2_06_P   Mut   8.58               8.58
```

### 1.3.5 SRY\_iPS12-M2\_12h00-Wt\_vs\_Mut

```
Input = ("
names   values  block
WT  8.27    iPS12_45_M2_12_P
WT  10.34   iPS12_45_M2_12_P
WT  10.60   iPS12_45_M2_12_P
WT  10.36   iPS12_45_M2_12_P
WT  10.35   iPS12_45_M2_12_P
WT  10.13   iPS12_45_M2_12_P
"
)
Data = read.table(textConnection(Input),header=TRUE)
Data$names = factor(Data$names,ordered=FALSE, levels=unique(Data$names))
Data$block = factor(Data$block,ordered=FALSE, levels=unique(Data$block))

# SRY_boxplot 1
boxplot(values ~ names,
        data = Data,
        ylab ="values",
        xlab ="names")
```

```
Data17 <- Data |>
  mutate(
    IQR = IQR(values, na.rm = TRUE),
    Outlier_upper = quantile(values, probs = c(.75), na.rm = TRUE) + 1.5 * IQR,
    Outlier_lower = quantile(values, probs = c(.25), na.rm = TRUE) - 1.5 * IQR,
    values_wo_outliers = if_else(values <= Outlier_lower | values >= Outlier_upper, NA, values))

boxplot(values_wo_outliers ~ names, Data17)
```

```
Data17b<- Data17|> select(block, names, values, values_wo_outliers)
Data17b
```

```
##              block names values values_wo_outliers
## 1 iPS12_45_M2_12_P    WT   8.27                 NA
## 2 iPS12_45_M2_12_P    WT  10.34              10.34
## 3 iPS12_45_M2_12_P    WT  10.60              10.60
## 4 iPS12_45_M2_12_P    WT  10.36              10.36
## 5 iPS12_45_M2_12_P    WT  10.35              10.35
## 6 iPS12_45_M2_12_P    WT  10.13              10.13
```

### 1.3.6 SRY\_iPS12-M2\_12h00-Mut

```
Input = ("
names   values  block
Mut 9.91    iPS12_82_M2_12_P
Mut 10.25   iPS12_82_M2_12_P
Mut 9.67    iPS12_82_M2_12_P
Mut 9.81    iPS12_82_M2_12_P
Mut 10.32   iPS12_82_M2_12_P
Mut 9.89    iPS12_82_M2_12_P
"
)
Data = read.table(textConnection(Input),header=TRUE)
Data$names = factor(Data$names,ordered=FALSE, levels=unique(Data$names))
Data$block = factor(Data$block,ordered=FALSE, levels=unique(Data$block))

# SRY_boxplot 1
boxplot(values ~ names,
        data = Data,
        ylab ="values",
        xlab ="names")
```

```
Data18 <- Data |>
  mutate(
    IQR = IQR(values, na.rm = TRUE),
    Outlier_upper = quantile(values, probs = c(.75), na.rm = TRUE) + 1.5 * IQR,
    Outlier_lower = quantile(values, probs = c(.25), na.rm = TRUE) - 1.5 * IQR,
    values_wo_outliers = if_else(values <= Outlier_lower | values >= Outlier_upper, NA, values))

boxplot(values_wo_outliers ~ names, Data18)
```

```
Data18b<- Data18|> select(block, names, values, values_wo_outliers)
Data18b
```

```
##              block names values values_wo_outliers
## 1 iPS12_82_M2_12_P   Mut   9.91               9.91
## 2 iPS12_82_M2_12_P   Mut  10.25              10.25
## 3 iPS12_82_M2_12_P   Mut   9.67               9.67
## 4 iPS12_82_M2_12_P   Mut   9.81               9.81
## 5 iPS12_82_M2_12_P   Mut  10.32              10.32
## 6 iPS12_82_M2_12_P   Mut   9.89               9.89
```

### 1.3.7 SRY\_iPS12\_M2\_24h00-Wt

```
Input = ("
names   values  block
WT  9.50    iPS12_45_M2_24_P
WT  9.76    iPS12_45_M2_24_P
WT  9.50    iPS12_45_M2_24_P
WT  9.34    iPS12_45_M2_24_P
WT  9.57    iPS12_45_M2_24_P
WT  9.09    iPS12_45_M2_24_P
"
)
Data = read.table(textConnection(Input),header=TRUE)
Data$names = factor(Data$names,ordered=FALSE, levels=unique(Data$names))
Data$block = factor(Data$block,ordered=FALSE, levels=unique(Data$block))

# SRY_boxplot 1
boxplot(values ~ names,
        data = Data,
        ylab ="values",
        xlab ="names")
```

```
Data19 <- Data |>
  mutate(
    IQR = IQR(values, na.rm = TRUE),
    Outlier_upper = quantile(values, probs = c(.75), na.rm = TRUE) + 1.5 * IQR,
    Outlier_lower = quantile(values, probs = c(.25), na.rm = TRUE) - 1.5 * IQR,
    values_wo_outliers = if_else(values <= Outlier_lower | values >= Outlier_upper, NA, values))

boxplot(values_wo_outliers ~ names, Data19)
```

```
Data19b<- Data19|> select(block, names, values, values_wo_outliers)
Data19b
```

```
##              block names values values_wo_outliers
## 1 iPS12_45_M2_24_P    WT   9.50               9.50
## 2 iPS12_45_M2_24_P    WT   9.76               9.76
## 3 iPS12_45_M2_24_P    WT   9.50               9.50
## 4 iPS12_45_M2_24_P    WT   9.34               9.34
## 5 iPS12_45_M2_24_P    WT   9.57               9.57
## 6 iPS12_45_M2_24_P    WT   9.09                 NA
```

### 1.3.8 SRY\_iPS12\_M2\_24h00-Mut

```
Input = ("
names   values  block
Mut 9.08    iPS12_82_M2_24_P
Mut 9.13    iPS12_82_M2_24_P
Mut 9.31    iPS12_82_M2_24_P
Mut 8.85    iPS12_82_M2_24_P
Mut 9.12    iPS12_82_M2_24_P
Mut 8.83    iPS12_82_M2_24_P
"
)
Data = read.table(textConnection(Input),header=TRUE)
Data$names = factor(Data$names,ordered=FALSE, levels=unique(Data$names))
Data$block = factor(Data$block,ordered=FALSE, levels=unique(Data$block))

# SRY_boxplot 1
boxplot(values ~ names,
        data = Data,
        ylab ="values",
        xlab ="names")
```

```
Data20 <- Data |>
  mutate(
    IQR = IQR(values, na.rm = TRUE),
    Outlier_upper = quantile(values, probs = c(.75), na.rm = TRUE) + 1.5 * IQR,
    Outlier_lower = quantile(values, probs = c(.25), na.rm = TRUE) - 1.5 * IQR,
    values_wo_outliers = if_else(values <= Outlier_lower | values >= Outlier_upper, NA, values))

boxplot(values_wo_outliers ~ names, Data20)
```

```
Data20b<- Data20|> select(block, names, values, values_wo_outliers)
Data20b
```

```
##              block names values values_wo_outliers
## 1 iPS12_82_M2_24_P   Mut   9.08               9.08
## 2 iPS12_82_M2_24_P   Mut   9.13               9.13
## 3 iPS12_82_M2_24_P   Mut   9.31               9.31
## 4 iPS12_82_M2_24_P   Mut   8.85               8.85
## 5 iPS12_82_M2_24_P   Mut   9.12               9.12
## 6 iPS12_82_M2_24_P   Mut   8.83               8.83
```

### 1.3.9 SRY\_iPS12-M2\_48h00-Wt

```
Input = ("
names   values  block
WT  10.60   iPS12_45_M2_48_P
WT  10.87   iPS12_45_M2_48_P
WT  10.64   iPS12_45_M2_48_P
WT  10.91   iPS12_45_M2_48_P
WT  10.96   iPS12_45_M2_48_P
WT  NA  iPS12_45_M2_48_P
"
)
Data = read.table(textConnection(Input),header=TRUE)
Data$names = factor(Data$names,ordered=FALSE, levels=unique(Data$names))
Data$block = factor(Data$block,ordered=FALSE, levels=unique(Data$block))

# SRY_boxplot 1
boxplot(values ~ names,
        data = Data,
        ylab ="values",
        xlab ="names")
```

```
Data21 <- Data |>
  mutate(
    IQR = IQR(values, na.rm = TRUE),
    Outlier_upper = quantile(values, probs = c(.75), na.rm = TRUE) + 1.5 * IQR,
    Outlier_lower = quantile(values, probs = c(.25), na.rm = TRUE) - 1.5 * IQR,
    values_wo_outliers = if_else(values <= Outlier_lower | values >= Outlier_upper, NA, values))

boxplot(values_wo_outliers ~ names, Data21)
```

```
Data21b<- Data21|> select(block, names, values, values_wo_outliers)
Data21b
```

```
##              block names values values_wo_outliers
## 1 iPS12_45_M2_48_P    WT  10.60              10.60
## 2 iPS12_45_M2_48_P    WT  10.87              10.87
## 3 iPS12_45_M2_48_P    WT  10.64              10.64
## 4 iPS12_45_M2_48_P    WT  10.91              10.91
## 5 iPS12_45_M2_48_P    WT  10.96              10.96
## 6 iPS12_45_M2_48_P    WT     NA                 NA
```

### 1.3.10 SRY\_iPS12-M2\_48h00-Mut

```
Input = ("
names   values  block
Mut 10.73   iPS12_82_M2_48_P
Mut 11.23   iPS12_82_M2_48_P
Mut 11.14   iPS12_82_M2_48_P
Mut 10.80   iPS12_82_M2_48_P
Mut 10.86   iPS12_82_M2_48_P
Mut 10.85   iPS12_82_M2_48_P
"
)
Data = read.table(textConnection(Input),header=TRUE)
Data$names = factor(Data$names,ordered=FALSE, levels=unique(Data$names))
Data$block = factor(Data$block,ordered=FALSE, levels=unique(Data$block))

# SRY_boxplot 1
boxplot(values ~ names,
        data = Data,
        ylab ="values",
        xlab ="names")
```

```
Data22 <- Data |>
  mutate(
    IQR = IQR(values, na.rm = TRUE),
    Outlier_upper = quantile(values, probs = c(.75), na.rm = TRUE) + 1.5 * IQR,
    Outlier_lower = quantile(values, probs = c(.25), na.rm = TRUE) - 1.5 * IQR,
    values_wo_outliers = if_else(values <= Outlier_lower | values >= Outlier_upper, NA, values))

boxplot(values_wo_outliers ~ names, Data22)
```

```
Data22b<- Data22|> select(block, names, values, values_wo_outliers)
Data22b
```

```
##              block names values values_wo_outliers
## 1 iPS12_82_M2_48_P   Mut  10.73              10.73
## 2 iPS12_82_M2_48_P   Mut  11.23              11.23
## 3 iPS12_82_M2_48_P   Mut  11.14              11.14
## 4 iPS12_82_M2_48_P   Mut  10.80              10.80
## 5 iPS12_82_M2_48_P   Mut  10.86              10.86
## 6 iPS12_82_M2_48_P   Mut  10.85              10.85
```

## 1.4 SRY\_iPS19

### 1.4.1 SRY\_iPS19-iPS-Wt

```
Input = ("
names   values  block
WT  10.02   iPS19_45_iPS
WT  9.89    iPS19_45_iPS
WT  10.11   iPS19_45_iPS
WT  10.11   iPS19_45_iPS
WT  9.82    iPS19_45_iPS
WT  9.92    iPS19_45_iPS
WT  8.79    iPS19_82_iPS
WT  9.22    iPS19_82_iPS
WT  9.28    iPS19_82_iPS
WT  9.50    iPS19_82_iPS
WT  9.52    iPS19_82_iPS
WT  9.07    iPS19_82_iPS
"
)
Data = read.table(textConnection(Input),header=TRUE)
Data$names = factor(Data$names,ordered=FALSE, levels=unique(Data$names))
Data$block = factor(Data$block,ordered=FALSE, levels=unique(Data$block))

# SRY_boxplot 1
boxplot(values ~ names,
        data = Data,
        ylab ="values",
        xlab ="names")
```

```
Data23 <- Data |>
  mutate(
    IQR = IQR(values, na.rm = TRUE),
    Outlier_upper = quantile(values, probs = c(.75), na.rm = TRUE) + 1.5 * IQR,
    Outlier_lower = quantile(values, probs = c(.25), na.rm = TRUE) - 1.5 * IQR,
    values_wo_outliers = if_else(values <= Outlier_lower | values >= Outlier_upper, NA, values))

boxplot(values_wo_outliers ~ names, Data23)
```

```
Data23b<- Data23|> select(block, names, values, values_wo_outliers)
Data23b
```

```
##           block names values values_wo_outliers
## 1  iPS19_45_iPS    WT  10.02              10.02
## 2  iPS19_45_iPS    WT   9.89               9.89
## 3  iPS19_45_iPS    WT  10.11              10.11
## 4  iPS19_45_iPS    WT  10.11              10.11
## 5  iPS19_45_iPS    WT   9.82               9.82
## 6  iPS19_45_iPS    WT   9.92               9.92
## 7  iPS19_82_iPS    WT   8.79               8.79
## 8  iPS19_82_iPS    WT   9.22               9.22
## 9  iPS19_82_iPS    WT   9.28               9.28
## 10 iPS19_82_iPS    WT   9.50               9.50
## 11 iPS19_82_iPS    WT   9.52               9.52
## 12 iPS19_82_iPS    WT   9.07               9.07
```

### 1.4.2 SRY\_iPS19-iPS-Mut

```
Input = ("
names   values  block
Mut 9.83    iPS19_82_iPS
Mut 10.20   iPS19_82_iPS
Mut 10.18   iPS19_82_iPS
Mut 9.93    iPS19_82_iPS
Mut 10.20   iPS19_82_iPS
Mut 10.05   iPS19_82_iPS
Mut 9.28    iPS19_82_iPS
Mut 9.57    iPS19_82_iPS
Mut 9.75    iPS19_82_iPS
Mut 9.29    iPS19_82_iPS
Mut 9.46    iPS19_82_iPS
Mut 9.57    iPS19_82_iPS
"
)
Data = read.table(textConnection(Input),header=TRUE)
Data$names = factor(Data$names,ordered=FALSE, levels=unique(Data$names))
Data$block = factor(Data$block,ordered=FALSE, levels=unique(Data$block))

# SRY_boxplot 1
boxplot(values ~ names,
        data = Data,
        ylab ="values",
        xlab ="names")
```

```
Data24 <- Data |>
  mutate(
    IQR = IQR(values, na.rm = TRUE),
    Outlier_upper = quantile(values, probs = c(.75), na.rm = TRUE) + 1.5 * IQR,
    Outlier_lower = quantile(values, probs = c(.25), na.rm = TRUE) - 1.5 * IQR,
    values_wo_outliers = if_else(values <= Outlier_lower | values >= Outlier_upper, NA, values))

boxplot(values_wo_outliers ~ names, Data24)
```

```
Data24b<- Data24|> select(block, names, values, values_wo_outliers)
Data24b
```

```
##           block names values values_wo_outliers
## 1  iPS19_82_iPS   Mut   9.83               9.83
## 2  iPS19_82_iPS   Mut  10.20              10.20
## 3  iPS19_82_iPS   Mut  10.18              10.18
## 4  iPS19_82_iPS   Mut   9.93               9.93
## 5  iPS19_82_iPS   Mut  10.20              10.20
## 6  iPS19_82_iPS   Mut  10.05              10.05
## 7  iPS19_82_iPS   Mut   9.28               9.28
## 8  iPS19_82_iPS   Mut   9.57               9.57
## 9  iPS19_82_iPS   Mut   9.75               9.75
## 10 iPS19_82_iPS   Mut   9.29               9.29
## 11 iPS19_82_iPS   Mut   9.46               9.46
## 12 iPS19_82_iPS   Mut   9.57               9.57
```
