# Supplementary material for: A conserved NR5A1-responsive enhancer regulates SRY in testis-determination
Source: Nat Commun. 2024 Mar 30;15:2796. doi: 10.1038/s41467-024-47162-2 (PMC10981742; doi:10.1038/s41467-024-47162-2)
Supplement: Supplementary file 34 — Supplementary Dataset 31 [file 41467_2024_47162_MOESM34_ESM.html]

Supplementary\_Data\_31


# Supplementary\_Data\_31

#### Denis

#### 2023-07-10

RT-qPCR analysis - SOX9 - outlier identification by the IQR method in
R

```
rm(list=ls())
```

```
library(tidyverse)
```

```
## ── Attaching core tidyverse packages ──────────────────────── tidyverse 2.0.0 ──
## ✔ dplyr     1.1.4     ✔ readr     2.1.5
## ✔ forcats   1.0.0     ✔ stringr   1.5.1
## ✔ ggplot2   3.4.4     ✔ tibble    3.2.1
## ✔ lubridate 1.9.3     ✔ tidyr     1.3.0
## ✔ purrr     1.0.2     
## ── Conflicts ────────────────────────────────────────── tidyverse_conflicts() ──
## ✖ dplyr::filter() masks stats::filter()
## ✖ dplyr::lag()    masks stats::lag()
## ℹ Use the conflicted package (<http://conflicted.r-lib.org/>) to force all conflicts to become errors
```

# 1 SOX9

## 1.1 SOX9\_iPS07

### 1.1.1 SOX9\_iPS07-M1\_36h00-Mut

```
Input = ("
names   values  block
Mut 7.68    iPS07-82-M1-1
Mut 8.32    iPS07-82-M1-2
Mut 7.92    iPS07-82-M1-3
Mut 7.32    iPS07-82-M1-4
Mut 7.47    iPS07-82-M1-5
"
)
Data = read.table(textConnection(Input),header=TRUE)
Data$names = factor(Data$names,ordered=FALSE, levels=unique(Data$names))
Data$block = factor(Data$block,ordered=FALSE, levels=unique(Data$block))

# SOX9_boxplot 1
boxplot(values ~ names,
        data = Data,
        ylab ="values",
        xlab ="names")
```

```
Data1 <- Data |>
  mutate(
    IQR = IQR(values, na.rm = TRUE),
    Outlier_upper = quantile(values, probs = c(.75), na.rm = TRUE) + 1.5 * IQR,
    Outlier_lower = quantile(values, probs = c(.25), na.rm = TRUE) - 1.5 * IQR,
    values_wo_outliers = if_else(values <= Outlier_lower | values >= Outlier_upper, NA, values))

boxplot(values_wo_outliers ~ names, Data1)
```

```
Data1b<- Data1|> select(block, names, values, values_wo_outliers)
Data1b
```

```
##           block names values values_wo_outliers
## 1 iPS07-82-M1-1   Mut   7.68               7.68
## 2 iPS07-82-M1-2   Mut   8.32               8.32
## 3 iPS07-82-M1-3   Mut   7.92               7.92
## 4 iPS07-82-M1-4   Mut   7.32               7.32
## 5 iPS07-82-M1-5   Mut   7.47               7.47
```

### 1.1.2 SOX9\_iPS07-M2\_48h00-Wt

```
Input = ("
names   values  block
WT  5.11    iPS07-45-M2_48
WT  4.87    iPS07-45-M2_48
WT  5.08    iPS07-45-M2_48
WT  5.31    iPS07-45-M2_48
WT  4.97    iPS07-45-M2_48
"
)
Data = read.table(textConnection(Input),header=TRUE)
Data$names = factor(Data$names,ordered=FALSE, levels=unique(Data$names))
Data$block = factor(Data$block,ordered=FALSE, levels=unique(Data$block))

# SOX9_boxplot 1
boxplot(values ~ names,
        data = Data,
        ylab ="values",
        xlab ="names")
```

```
Data2 <- Data |>
  mutate(
    IQR = IQR(values, na.rm = TRUE),
    Outlier_upper = quantile(values, probs = c(.75), na.rm = TRUE) + 1.5 * IQR,
    Outlier_lower = quantile(values, probs = c(.25), na.rm = TRUE) - 1.5 * IQR,
    values_wo_outliers = if_else(values <= Outlier_lower | values >= Outlier_upper, NA, values))

boxplot(values_wo_outliers ~ names, Data2)
```

```
Data2b<- Data2|> select(block, names, values, values_wo_outliers)
Data2b
```

```
##            block names values values_wo_outliers
## 1 iPS07-45-M2_48    WT   5.11               5.11
## 2 iPS07-45-M2_48    WT   4.87               4.87
## 3 iPS07-45-M2_48    WT   5.08               5.08
## 4 iPS07-45-M2_48    WT   5.31               5.31
## 5 iPS07-45-M2_48    WT   4.97               4.97
```

### 1.1.3 SOX9\_iPS07-M2\_48h00-Mut

```
Input = ("
names   values  block
Mut 5.73    iPS07-82-M2_48
Mut 8.82    iPS07-82-M2_48
Mut 8.48    iPS07-82-M2_48
Mut 6.00    iPS07-82-M2_48
Mut 7.42    iPS07-82-M2_48
"
)
Data = read.table(textConnection(Input),header=TRUE)
Data$names = factor(Data$names,ordered=FALSE, levels=unique(Data$names))
Data$block = factor(Data$block,ordered=FALSE, levels=unique(Data$block))

# SOX9_boxplot 1
boxplot(values ~ names,
        data = Data,
        ylab ="values",
        xlab ="names")
```

```
Data3 <- Data |>
  mutate(
    IQR = IQR(values, na.rm = TRUE),
    Outlier_upper = quantile(values, probs = c(.75), na.rm = TRUE) + 1.5 * IQR,
    Outlier_lower = quantile(values, probs = c(.25), na.rm = TRUE) - 1.5 * IQR,
    values_wo_outliers = if_else(values <= Outlier_lower | values >= Outlier_upper, NA, values))

boxplot(values_wo_outliers ~ names, Data3)
```

```
Data3b<- Data3|> select(block, names, values, values_wo_outliers)
Data3b
```

```
##            block names values values_wo_outliers
## 1 iPS07-82-M2_48   Mut   5.73               5.73
## 2 iPS07-82-M2_48   Mut   8.82               8.82
## 3 iPS07-82-M2_48   Mut   8.48               8.48
## 4 iPS07-82-M2_48   Mut   6.00               6.00
## 5 iPS07-82-M2_48   Mut   7.42               7.42
```

## 1.2 SOX9\_iPS09

### 1.2.1 SOX9\_iPS09-M1\_36h00-Wt

```
Input = ("
names   values  block
WT  8.98    iPS07-45-iPS
WT  12.25   iPS07-45-iPS
WT  11.26   iPS07-45-iPS
WT  5.93    iPS07-45-iPS
WT  7.69    iPS07-45-iPS
WT  6.80    iPS07-45-iPS
WT  6.58    iPS07-45-iPS
"
)
Data = read.table(textConnection(Input),header=TRUE)
Data$names = factor(Data$names,ordered=FALSE, levels=unique(Data$names))
Data$block = factor(Data$block,ordered=FALSE, levels=unique(Data$block))

# SOX9_boxplot 1
boxplot(values ~ names,
        data = Data,
        ylab ="values",
        xlab ="names")
```

```
Data4 <- Data |>
  mutate(
    IQR = IQR(values, na.rm = TRUE),
    Outlier_upper = quantile(values, probs = c(.75), na.rm = TRUE) + 1.5 * IQR,
    Outlier_lower = quantile(values, probs = c(.25), na.rm = TRUE) - 1.5 * IQR,
    values_wo_outliers = if_else(values <= Outlier_lower | values >= Outlier_upper, NA, values))

boxplot(values_wo_outliers ~ names, Data4)
```

```
Data4b<- Data4|> select(block, names, values, values_wo_outliers)
Data4b
```

```
##          block names values values_wo_outliers
## 1 iPS07-45-iPS    WT   8.98               8.98
## 2 iPS07-45-iPS    WT  12.25              12.25
## 3 iPS07-45-iPS    WT  11.26              11.26
## 4 iPS07-45-iPS    WT   5.93               5.93
## 5 iPS07-45-iPS    WT   7.69               7.69
## 6 iPS07-45-iPS    WT   6.80               6.80
## 7 iPS07-45-iPS    WT   6.58               6.58
```

### 1.2.2 SOX9\_iPS09-M1\_36h00-Mut

```
Input = ("
names   values  block
Mut 7.90    iPS09-82-iPS
Mut 7.69    iPS09-82-iPS
Mut 7.52    iPS09-82-iPS
Mut 7.07    iPS09-82-iPS
Mut 8.41    iPS09-82-iPS
Mut 8.11    iPS09-82-iPS
"
)
Data = read.table(textConnection(Input),header=TRUE)
Data$names = factor(Data$names,ordered=FALSE, levels=unique(Data$names))
Data$block = factor(Data$block,ordered=FALSE, levels=unique(Data$block))

# SOX9_boxplot 1
boxplot(values ~ names,
        data = Data,
        ylab ="values",
        xlab ="names")
```

```
Data5 <- Data |>
  mutate(
    IQR = IQR(values, na.rm = TRUE),
    Outlier_upper = quantile(values, probs = c(.75), na.rm = TRUE) + 1.5 * IQR,
    Outlier_lower = quantile(values, probs = c(.25), na.rm = TRUE) - 1.5 * IQR,
    values_wo_outliers = if_else(values <= Outlier_lower | values >= Outlier_upper, NA, values))

boxplot(values_wo_outliers ~ names, Data5)
```

```
Data5b<- Data5|> select(block, names, values, values_wo_outliers)
Data5b
```

```
##          block names values values_wo_outliers
## 1 iPS09-82-iPS   Mut   7.90               7.90
## 2 iPS09-82-iPS   Mut   7.69               7.69
## 3 iPS09-82-iPS   Mut   7.52               7.52
## 4 iPS09-82-iPS   Mut   7.07               7.07
## 5 iPS09-82-iPS   Mut   8.41               8.41
## 6 iPS09-82-iPS   Mut   8.11               8.11
```

### 1.2.3 SOX9\_iPS09-M2\_24h00-Wt

```
Input = ("
names   values  block
WT  7.16    iPS09-45-M1_36h00
WT  6.79    iPS09-45-M1_36h00
WT  7.02    iPS09-45-M1_36h00
WT  6.89    iPS09-45-M1_36h00
WT  7.13    iPS09-45-M1_36h00
"
)
Data = read.table(textConnection(Input),header=TRUE)
Data$names = factor(Data$names,ordered=FALSE, levels=unique(Data$names))
Data$block = factor(Data$block,ordered=FALSE, levels=unique(Data$block))

# SOX9_boxplot 1
boxplot(values ~ names,
        data = Data,
        ylab ="values",
        xlab ="names")
```

```
Data6 <- Data |>
  mutate(
    IQR = IQR(values, na.rm = TRUE),
    Outlier_upper = quantile(values, probs = c(.75), na.rm = TRUE) + 1.5 * IQR,
    Outlier_lower = quantile(values, probs = c(.25), na.rm = TRUE) - 1.5 * IQR,
    values_wo_outliers = if_else(values <= Outlier_lower | values >= Outlier_upper, NA, values))

boxplot(values_wo_outliers ~ names, Data6)
```

```
Data6b<- Data6|> select(block, names, values, values_wo_outliers)
Data6b
```

```
##               block names values values_wo_outliers
## 1 iPS09-45-M1_36h00    WT   7.16               7.16
## 2 iPS09-45-M1_36h00    WT   6.79               6.79
## 3 iPS09-45-M1_36h00    WT   7.02               7.02
## 4 iPS09-45-M1_36h00    WT   6.89               6.89
## 5 iPS09-45-M1_36h00    WT   7.13               7.13
```

### 1.2.4 SOX9\_iPS09-M2\_24h00-Mut

```
Input = ("
names   values  block
Mut 6.51    iPS09-82-M1_36h00
Mut 6.39    iPS09-82-M1_36h00
Mut 6.22    iPS09-82-M1_36h00
Mut 6.22    iPS09-82-M1_36h00
Mut 6.14    iPS09-82-M1_36h00
"
)
Data = read.table(textConnection(Input),header=TRUE)
Data$names = factor(Data$names,ordered=FALSE, levels=unique(Data$names))
Data$block = factor(Data$block,ordered=FALSE, levels=unique(Data$block))

# SOX9_boxplot 1
boxplot(values ~ names,
        data = Data,
        ylab ="values",
        xlab ="names")
```

```
Data7 <- Data |>
  mutate(
    IQR = IQR(values, na.rm = TRUE),
    Outlier_upper = quantile(values, probs = c(.75), na.rm = TRUE) + 1.5 * IQR,
    Outlier_lower = quantile(values, probs = c(.25), na.rm = TRUE) - 1.5 * IQR,
    values_wo_outliers = if_else(values <= Outlier_lower | values >= Outlier_upper, NA, values))

boxplot(values_wo_outliers ~ names, Data7)
```

```
Data7b<- Data7|> select(block, names, values, values_wo_outliers)
Data7b
```

```
##               block names values values_wo_outliers
## 1 iPS09-82-M1_36h00   Mut   6.51               6.51
## 2 iPS09-82-M1_36h00   Mut   6.39               6.39
## 3 iPS09-82-M1_36h00   Mut   6.22               6.22
## 4 iPS09-82-M1_36h00   Mut   6.22               6.22
## 5 iPS09-82-M1_36h00   Mut   6.14               6.14
```

### 1.2.5 SOX9\_iPS09-M3\_24h00-Wt

```
Input = ("
names   values  block
WT  7.07    iPS09-45-M2_24h00
WT  6.15    iPS09-45-M2_24h00
WT  6.86    iPS09-45-M2_24h00
WT  5.89    iPS09-45-M2_24h00
WT  5.81    iPS09-45-M2_24h00
"
)
Data = read.table(textConnection(Input),header=TRUE)
Data$names = factor(Data$names,ordered=FALSE, levels=unique(Data$names))
Data$block = factor(Data$block,ordered=FALSE, levels=unique(Data$block))

# SOX9_boxplot 1
boxplot(values ~ names,
        data = Data,
        ylab ="values",
        xlab ="names")
```

```
Data8 <- Data |>
  mutate(
    IQR = IQR(values, na.rm = TRUE),
    Outlier_upper = quantile(values, probs = c(.75), na.rm = TRUE) + 1.5 * IQR,
    Outlier_lower = quantile(values, probs = c(.25), na.rm = TRUE) - 1.5 * IQR,
    values_wo_outliers = if_else(values <= Outlier_lower | values >= Outlier_upper, NA, values))

boxplot(values_wo_outliers ~ names, Data8)
```

```
Data8b<- Data8|> select(block, names, values, values_wo_outliers)
Data8b
```

```
##               block names values values_wo_outliers
## 1 iPS09-45-M2_24h00    WT   7.07               7.07
## 2 iPS09-45-M2_24h00    WT   6.15               6.15
## 3 iPS09-45-M2_24h00    WT   6.86               6.86
## 4 iPS09-45-M2_24h00    WT   5.89               5.89
## 5 iPS09-45-M2_24h00    WT   5.81               5.81
```

### 1.2.6 SOX9\_iPS09-M3\_48h00-Wt

```
Input = ("
names   values  block
Mut 5.88    iPS09-82-M2_24h00
Mut 6.04    iPS09-82-M2_24h00
Mut 5.87    iPS09-82-M2_24h00
Mut 5.89    iPS09-82-M2_24h00
Mut 6.08    iPS09-82-M2_24h00
"
)
Data = read.table(textConnection(Input),header=TRUE)
Data$names = factor(Data$names,ordered=FALSE, levels=unique(Data$names))
Data$block = factor(Data$block,ordered=FALSE, levels=unique(Data$block))

# SOX9_boxplot 1
boxplot(values ~ names,
        data = Data,
        ylab ="values",
        xlab ="names")
```

```
Data9 <- Data |>
  mutate(
    IQR = IQR(values, na.rm = TRUE),
    Outlier_upper = quantile(values, probs = c(.75), na.rm = TRUE) + 1.5 * IQR,
    Outlier_lower = quantile(values, probs = c(.25), na.rm = TRUE) - 1.5 * IQR,
    values_wo_outliers = if_else(values <= Outlier_lower | values >= Outlier_upper, NA, values))

boxplot(values_wo_outliers ~ names, Data9)
```

```
Data9b<- Data9|> select(block, names, values, values_wo_outliers)
Data9b
```

```
##               block names values values_wo_outliers
## 1 iPS09-82-M2_24h00   Mut   5.88               5.88
## 2 iPS09-82-M2_24h00   Mut   6.04               6.04
## 3 iPS09-82-M2_24h00   Mut   5.87               5.87
## 4 iPS09-82-M2_24h00   Mut   5.89               5.89
## 5 iPS09-82-M2_24h00   Mut   6.08               6.08
```

### 1.2.7 SOX9\_iPS09-M3\_48h00-Mut

```
Input = ("
names   values  block
WT  6.38    iPS09-45-M3_24h00
WT  5.33    iPS09-45-M3_24h00
WT  5.21    iPS09-45-M3_24h00
WT  4.68    iPS09-45-M3_24h00
WT  5.99    iPS09-45-M3_24h00
"
)
Data = read.table(textConnection(Input),header=TRUE)
Data$names = factor(Data$names,ordered=FALSE, levels=unique(Data$names))
Data$block = factor(Data$block,ordered=FALSE, levels=unique(Data$block))

# SOX9_boxplot 1
boxplot(values ~ names,
        data = Data,
        ylab ="values",
        xlab ="names")
```

```
Data10 <- Data |>
  mutate(
    IQR = IQR(values, na.rm = TRUE),
    Outlier_upper = quantile(values, probs = c(.75), na.rm = TRUE) + 1.5 * IQR,
    Outlier_lower = quantile(values, probs = c(.25), na.rm = TRUE) - 1.5 * IQR,
    values_wo_outliers = if_else(values <= Outlier_lower | values >= Outlier_upper, NA, values))

boxplot(values_wo_outliers ~ names, Data10)
```

```
Data10b<- Data10|> select(block, names, values, values_wo_outliers)
Data10b
```

```
##               block names values values_wo_outliers
## 1 iPS09-45-M3_24h00    WT   6.38               6.38
## 2 iPS09-45-M3_24h00    WT   5.33               5.33
## 3 iPS09-45-M3_24h00    WT   5.21               5.21
## 4 iPS09-45-M3_24h00    WT   4.68               4.68
## 5 iPS09-45-M3_24h00    WT   5.99               5.99
```

### 1.2.8 SOX9\_iPS09-iPS-Wt

```
Input = ("
names   values  block
WT  5.26    iPS09-45-M3_48h00
WT  5.41    iPS09-45-M3_48h00
WT  5.20    iPS09-45-M3_48h00
WT  5.76    iPS09-45-M3_48h00
WT  6.47    iPS09-45-M3_48h00
"
)
Data = read.table(textConnection(Input),header=TRUE)
Data$names = factor(Data$names,ordered=FALSE, levels=unique(Data$names))
Data$block = factor(Data$block,ordered=FALSE, levels=unique(Data$block))

# SOX9_boxplot 1
boxplot(values ~ names,
        data = Data,
        ylab ="values",
        xlab ="names")
```

```
Data11 <- Data |>
  mutate(
    IQR = IQR(values, na.rm = TRUE),
    Outlier_upper = quantile(values, probs = c(.75), na.rm = TRUE) + 1.5 * IQR,
    Outlier_lower = quantile(values, probs = c(.25), na.rm = TRUE) - 1.5 * IQR,
    values_wo_outliers = if_else(values <= Outlier_lower | values >= Outlier_upper, NA, values))

boxplot(values_wo_outliers ~ names, Data11)
```

```
Data11b<- Data11|> select(block, names, values, values_wo_outliers)
Data11b
```

```
##               block names values values_wo_outliers
## 1 iPS09-45-M3_48h00    WT   5.26               5.26
## 2 iPS09-45-M3_48h00    WT   5.41               5.41
## 3 iPS09-45-M3_48h00    WT   5.20               5.20
## 4 iPS09-45-M3_48h00    WT   5.76               5.76
## 5 iPS09-45-M3_48h00    WT   6.47               6.47
```

### 1.2.9 SOX9\_iPS09-iPS-Mut

```
Input = ("
names   values  block
Mut 5.54    iPS09-82-M3_48h00
Mut 5.19    iPS09-82-M3_48h00
Mut 5.22    iPS09-82-M3_48h00
Mut 4.88    iPS09-82-M3_48h00
Mut 4.34    iPS09-82-M3_48h00
"
)
Data = read.table(textConnection(Input),header=TRUE)
Data$names = factor(Data$names,ordered=FALSE, levels=unique(Data$names))
Data$block = factor(Data$block,ordered=FALSE, levels=unique(Data$block))

# SOX9_boxplot 1
boxplot(values ~ names,
        data = Data,
        ylab ="values",
        xlab ="names")
```

```
Data12 <- Data |>
  mutate(
    IQR = IQR(values, na.rm = TRUE),
    Outlier_upper = quantile(values, probs = c(.75), na.rm = TRUE) + 1.5 * IQR,
    Outlier_lower = quantile(values, probs = c(.25), na.rm = TRUE) - 1.5 * IQR,
    values_wo_outliers = if_else(values <= Outlier_lower | values >= Outlier_upper, NA, values))

boxplot(values_wo_outliers ~ names, Data12)
```

```
Data12b<- Data12|> select(block, names, values, values_wo_outliers)
Data12b
```

```
##               block names values values_wo_outliers
## 1 iPS09-82-M3_48h00   Mut   5.54               5.54
## 2 iPS09-82-M3_48h00   Mut   5.19               5.19
## 3 iPS09-82-M3_48h00   Mut   5.22               5.22
## 4 iPS09-82-M3_48h00   Mut   4.88               4.88
## 5 iPS09-82-M3_48h00   Mut   4.34                 NA
```

## 1.3 SOX9\_iPS12

### 1.3.1 SOX9\_iPS12-M1\_36h00-Wt

```
Input = ("
names   values  block
WT  7.22    iPS12_45_M1_36_P
WT  7.41    iPS12_45_M1_36_P
WT  7.24    iPS12_45_M1_36_P
WT  7.03    iPS12_45_M1_36_P
WT  7.31    iPS12_45_M1_36_P
WT  7.72    iPS12_45_M1_36_P
"
)
Data = read.table(textConnection(Input),header=TRUE)
Data$names = factor(Data$names,ordered=FALSE, levels=unique(Data$names))
Data$block = factor(Data$block,ordered=FALSE, levels=unique(Data$block))

# SOX9_boxplot 1
boxplot(values ~ names,
        data = Data,
        ylab ="values",
        xlab ="names")
```

```
Data13 <- Data |>
  mutate(
    IQR = IQR(values, na.rm = TRUE),
    Outlier_upper = quantile(values, probs = c(.75), na.rm = TRUE) + 1.5 * IQR,
    Outlier_lower = quantile(values, probs = c(.25), na.rm = TRUE) - 1.5 * IQR,
    values_wo_outliers = if_else(values <= Outlier_lower | values >= Outlier_upper, NA, values))

boxplot(values_wo_outliers ~ names, Data13)
```

```
Data13b<- Data13|> select(block, names, values, values_wo_outliers)
Data13b
```

```
##              block names values values_wo_outliers
## 1 iPS12_45_M1_36_P    WT   7.22               7.22
## 2 iPS12_45_M1_36_P    WT   7.41               7.41
## 3 iPS12_45_M1_36_P    WT   7.24               7.24
## 4 iPS12_45_M1_36_P    WT   7.03               7.03
## 5 iPS12_45_M1_36_P    WT   7.31               7.31
## 6 iPS12_45_M1_36_P    WT   7.72                 NA
```

### 1.3.2 SOX9\_iPS12-M1\_36h00-Mut

```
Input = ("
names   values  block
Mut 6.15    iPS12_82_M1_36_P
Mut 6.17    iPS12_82_M1_36_P
Mut 6.18    iPS12_82_M1_36_P
Mut 6.23    iPS12_82_M1_36_P
Mut 6.69    iPS12_82_M1_36_P
Mut 6.28    iPS12_82_M1_36_P
"
)
Data = read.table(textConnection(Input),header=TRUE)
Data$names = factor(Data$names,ordered=FALSE, levels=unique(Data$names))
Data$block = factor(Data$block,ordered=FALSE, levels=unique(Data$block))

# SOX9_boxplot 1
boxplot(values ~ names,
        data = Data,
        ylab ="values",
        xlab ="names")
```

```
Data14 <- Data |>
  mutate(
    IQR = IQR(values, na.rm = TRUE),
    Outlier_upper = quantile(values, probs = c(.75), na.rm = TRUE) + 1.5 * IQR,
    Outlier_lower = quantile(values, probs = c(.25), na.rm = TRUE) - 1.5 * IQR,
    values_wo_outliers = if_else(values <= Outlier_lower | values >= Outlier_upper, NA, values))

boxplot(values_wo_outliers ~ names, Data14)
```

```
Data14b<- Data14|> select(block, names, values, values_wo_outliers)
Data14b
```

```
##              block names values values_wo_outliers
## 1 iPS12_82_M1_36_P   Mut   6.15               6.15
## 2 iPS12_82_M1_36_P   Mut   6.17               6.17
## 3 iPS12_82_M1_36_P   Mut   6.18               6.18
## 4 iPS12_82_M1_36_P   Mut   6.23               6.23
## 5 iPS12_82_M1_36_P   Mut   6.69                 NA
## 6 iPS12_82_M1_36_P   Mut   6.28               6.28
```

### 1.3.3 SOX9\_iPS12-M2\_06h00-Wt

```
Input = ("
names   values  block
WT  8.65    iPS12_45_M2_06_P
WT  9.41    iPS12_45_M2_06_P
WT  9.10    iPS12_45_M2_06_P
WT  9.97    iPS12_45_M2_06_P
WT  9.94    iPS12_45_M2_06_P
WT  NA  iPS12_45_M2_06_P
"
)
Data = read.table(textConnection(Input),header=TRUE)
Data$names = factor(Data$names,ordered=FALSE, levels=unique(Data$names))
Data$block = factor(Data$block,ordered=FALSE, levels=unique(Data$block))

# SOX9_boxplot 1
boxplot(values ~ names,
        data = Data,
        ylab ="values",
        xlab ="names")
```

```
Data15 <- Data |>
  mutate(
    IQR = IQR(values, na.rm = TRUE),
    Outlier_upper = quantile(values, probs = c(.75), na.rm = TRUE) + 1.5 * IQR,
    Outlier_lower = quantile(values, probs = c(.25), na.rm = TRUE) - 1.5 * IQR,
    values_wo_outliers = if_else(values <= Outlier_lower | values >= Outlier_upper, NA, values))

boxplot(values_wo_outliers ~ names, Data15)
```

```
Data15b<- Data15|> select(block, names, values, values_wo_outliers)
Data15b
```

```
##              block names values values_wo_outliers
## 1 iPS12_45_M2_06_P    WT   8.65               8.65
## 2 iPS12_45_M2_06_P    WT   9.41               9.41
## 3 iPS12_45_M2_06_P    WT   9.10               9.10
## 4 iPS12_45_M2_06_P    WT   9.97               9.97
## 5 iPS12_45_M2_06_P    WT   9.94               9.94
## 6 iPS12_45_M2_06_P    WT     NA                 NA
```

### 1.3.4 SOX9\_iPS12-M2\_06h00-Mut

```
Input = ("
names   values  block
Mut NA  iPS12_82_M2_06_P
Mut 8.18    iPS12_82_M2_06_P
Mut 9.10    iPS12_82_M2_06_P
Mut 9.54    iPS12_82_M2_06_P
Mut 9.92    iPS12_82_M2_06_P
Mut 9.32    iPS12_82_M2_06_P
"
)
Data = read.table(textConnection(Input),header=TRUE)
Data$names = factor(Data$names,ordered=FALSE, levels=unique(Data$names))
Data$block = factor(Data$block,ordered=FALSE, levels=unique(Data$block))

# SOX9_boxplot 1
boxplot(values ~ names,
        data = Data,
        ylab ="values",
        xlab ="names")
```

```
Data16 <- Data |>
  mutate(
    IQR = IQR(values, na.rm = TRUE),
    Outlier_upper = quantile(values, probs = c(.75), na.rm = TRUE) + 1.5 * IQR,
    Outlier_lower = quantile(values, probs = c(.25), na.rm = TRUE) - 1.5 * IQR,
    values_wo_outliers = if_else(values <= Outlier_lower | values >= Outlier_upper, NA, values))

boxplot(values_wo_outliers ~ names, Data16)
```

```
Data16b<- Data16|> select(block, names, values, values_wo_outliers)
Data16b
```

```
##              block names values values_wo_outliers
## 1 iPS12_82_M2_06_P   Mut     NA                 NA
## 2 iPS12_82_M2_06_P   Mut   8.18                 NA
## 3 iPS12_82_M2_06_P   Mut   9.10               9.10
## 4 iPS12_82_M2_06_P   Mut   9.54               9.54
## 5 iPS12_82_M2_06_P   Mut   9.92               9.92
## 6 iPS12_82_M2_06_P   Mut   9.32               9.32
```

### 1.3.5 SOX9\_iPS12-M2\_12h00-Wt\_vs\_Mut

```
Input = ("
names   values  block
WT  NA  iPS12_45_M2_12_P
WT  9.02    iPS12_45_M2_12_P
WT  8.55    iPS12_45_M2_12_P
WT  10.87   iPS12_45_M2_12_P
WT  9.08    iPS12_45_M2_12_P
WT  8.92    iPS12_45_M2_12_P
"
)
Data = read.table(textConnection(Input),header=TRUE)
Data$names = factor(Data$names,ordered=FALSE, levels=unique(Data$names))
Data$block = factor(Data$block,ordered=FALSE, levels=unique(Data$block))

# SOX9_boxplot 1
boxplot(values ~ names,
        data = Data,
        ylab ="values",
        xlab ="names")
```

```
Data17 <- Data |>
  mutate(
    IQR = IQR(values, na.rm = TRUE),
    Outlier_upper = quantile(values, probs = c(.75), na.rm = TRUE) + 1.5 * IQR,
    Outlier_lower = quantile(values, probs = c(.25), na.rm = TRUE) - 1.5 * IQR,
    values_wo_outliers = if_else(values <= Outlier_lower | values >= Outlier_upper, NA, values))

boxplot(values_wo_outliers ~ names, Data17)
```

```
Data17b<- Data17|> select(block, names, values, values_wo_outliers)
Data17b
```

```
##              block names values values_wo_outliers
## 1 iPS12_45_M2_12_P    WT     NA                 NA
## 2 iPS12_45_M2_12_P    WT   9.02               9.02
## 3 iPS12_45_M2_12_P    WT   8.55                 NA
## 4 iPS12_45_M2_12_P    WT  10.87                 NA
## 5 iPS12_45_M2_12_P    WT   9.08               9.08
## 6 iPS12_45_M2_12_P    WT   8.92               8.92
```

### 1.3.6 SOX9\_iPS12-M2\_12h00-Mut

```
Input = ("
names   values  block
Mut 8.51    iPS12_82_M2_12_P
Mut 8.03    iPS12_82_M2_12_P
Mut 10.79   iPS12_82_M2_12_P
Mut 8.39    iPS12_82_M2_12_P
Mut 9.72    iPS12_82_M2_12_P
Mut 10.10   iPS12_82_M2_12_P
"
)
Data = read.table(textConnection(Input),header=TRUE)
Data$names = factor(Data$names,ordered=FALSE, levels=unique(Data$names))
Data$block = factor(Data$block,ordered=FALSE, levels=unique(Data$block))

# SOX9_boxplot 1
boxplot(values ~ names,
        data = Data,
        ylab ="values",
        xlab ="names")
```

```
Data18 <- Data |>
  mutate(
    IQR = IQR(values, na.rm = TRUE),
    Outlier_upper = quantile(values, probs = c(.75), na.rm = TRUE) + 1.5 * IQR,
    Outlier_lower = quantile(values, probs = c(.25), na.rm = TRUE) - 1.5 * IQR,
    values_wo_outliers = if_else(values <= Outlier_lower | values >= Outlier_upper, NA, values))

boxplot(values_wo_outliers ~ names, Data18)
```

```
Data18b<- Data18|> select(block, names, values, values_wo_outliers)
Data18b
```

```
##              block names values values_wo_outliers
## 1 iPS12_82_M2_12_P   Mut   8.51               8.51
## 2 iPS12_82_M2_12_P   Mut   8.03               8.03
## 3 iPS12_82_M2_12_P   Mut  10.79              10.79
## 4 iPS12_82_M2_12_P   Mut   8.39               8.39
## 5 iPS12_82_M2_12_P   Mut   9.72               9.72
## 6 iPS12_82_M2_12_P   Mut  10.10              10.10
```

### 1.3.7 SOX9\_iPS12\_M2\_24h00-Wt

```
Input = ("
names   values  block
WT  6.35    iPS12_45_M2_24_P
WT  7.32    iPS12_45_M2_24_P
WT  6.42    iPS12_45_M2_24_P
WT  6.71    iPS12_45_M2_24_P
WT  6.63    iPS12_45_M2_24_P
WT  10.74   iPS12_45_M2_24_P
"
)
Data = read.table(textConnection(Input),header=TRUE)
Data$names = factor(Data$names,ordered=FALSE, levels=unique(Data$names))
Data$block = factor(Data$block,ordered=FALSE, levels=unique(Data$block))

# SOX9_boxplot 1
boxplot(values ~ names,
        data = Data,
        ylab ="values",
        xlab ="names")
```

```
Data19 <- Data |>
  mutate(
    IQR = IQR(values, na.rm = TRUE),
    Outlier_upper = quantile(values, probs = c(.75), na.rm = TRUE) + 1.5 * IQR,
    Outlier_lower = quantile(values, probs = c(.25), na.rm = TRUE) - 1.5 * IQR,
    values_wo_outliers = if_else(values <= Outlier_lower | values >= Outlier_upper, NA, values))

boxplot(values_wo_outliers ~ names, Data19)
```

```
Data19b<- Data19|> select(block, names, values, values_wo_outliers)
Data19b
```

```
##              block names values values_wo_outliers
## 1 iPS12_45_M2_24_P    WT   6.35               6.35
## 2 iPS12_45_M2_24_P    WT   7.32               7.32
## 3 iPS12_45_M2_24_P    WT   6.42               6.42
## 4 iPS12_45_M2_24_P    WT   6.71               6.71
## 5 iPS12_45_M2_24_P    WT   6.63               6.63
## 6 iPS12_45_M2_24_P    WT  10.74                 NA
```

### 1.3.8 SOX9\_iPS12\_M2\_24h00-Mut

```
Input = ("
names   values  block
Mut 8.24    iPS12_82_M2_24_P
Mut 7.83    iPS12_82_M2_24_P
Mut 7.47    iPS12_82_M2_24_P
Mut 8.12    iPS12_82_M2_24_P
Mut 7.52    iPS12_82_M2_24_P
Mut 9.68    iPS12_82_M2_24_P
"
)
Data = read.table(textConnection(Input),header=TRUE)
Data$names = factor(Data$names,ordered=FALSE, levels=unique(Data$names))
Data$block = factor(Data$block,ordered=FALSE, levels=unique(Data$block))

# SOX9_boxplot 1
boxplot(values ~ names,
        data = Data,
        ylab ="values",
        xlab ="names")
```

```
Data20 <- Data |>
  mutate(
    IQR = IQR(values, na.rm = TRUE),
    Outlier_upper = quantile(values, probs = c(.75), na.rm = TRUE) + 1.5 * IQR,
    Outlier_lower = quantile(values, probs = c(.25), na.rm = TRUE) - 1.5 * IQR,
    values_wo_outliers = if_else(values <= Outlier_lower | values >= Outlier_upper, NA, values))

boxplot(values_wo_outliers ~ names, Data20)
```

```
Data20b<- Data20|> select(block, names, values, values_wo_outliers)
Data20b
```

```
##              block names values values_wo_outliers
## 1 iPS12_82_M2_24_P   Mut   8.24               8.24
## 2 iPS12_82_M2_24_P   Mut   7.83               7.83
## 3 iPS12_82_M2_24_P   Mut   7.47               7.47
## 4 iPS12_82_M2_24_P   Mut   8.12               8.12
## 5 iPS12_82_M2_24_P   Mut   7.52               7.52
## 6 iPS12_82_M2_24_P   Mut   9.68                 NA
```

### 1.3.9 SOX9\_iPS12-M2\_48h00-Wt

```
Input = ("
names   values  block
WT  5.42    iPS12_45_M2_48_P
WT  6.85    iPS12_45_M2_48_P
WT  7.00    iPS12_45_M2_48_P
WT  6.71    iPS12_45_M2_48_P
WT  6.94    iPS12_45_M2_48_P
WT  NA  iPS12_45_M2_48_P
"
)
Data = read.table(textConnection(Input),header=TRUE)
Data$names = factor(Data$names,ordered=FALSE, levels=unique(Data$names))
Data$block = factor(Data$block,ordered=FALSE, levels=unique(Data$block))

# SOX9_boxplot 1
boxplot(values ~ names,
        data = Data,
        ylab ="values",
        xlab ="names")
```

```
Data21 <- Data |>
  mutate(
    IQR = IQR(values, na.rm = TRUE),
    Outlier_upper = quantile(values, probs = c(.75), na.rm = TRUE) + 1.5 * IQR,
    Outlier_lower = quantile(values, probs = c(.25), na.rm = TRUE) - 1.5 * IQR,
    values_wo_outliers = if_else(values <= Outlier_lower | values >= Outlier_upper, NA, values))

boxplot(values_wo_outliers ~ names, Data21)
```

```
Data21b<- Data21|> select(block, names, values, values_wo_outliers)
Data21b
```

```
##              block names values values_wo_outliers
## 1 iPS12_45_M2_48_P    WT   5.42                 NA
## 2 iPS12_45_M2_48_P    WT   6.85               6.85
## 3 iPS12_45_M2_48_P    WT   7.00               7.00
## 4 iPS12_45_M2_48_P    WT   6.71               6.71
## 5 iPS12_45_M2_48_P    WT   6.94               6.94
## 6 iPS12_45_M2_48_P    WT     NA                 NA
```

### 1.3.10 SOX9\_iPS12-M2\_48h00-Mut

```
Input = ("
names   values  block
Mut 6.15    iPS12_82_M2_48_P
Mut 6.56    iPS12_82_M2_48_P
Mut 7.01    iPS12_82_M2_48_P
Mut 6.19    iPS12_82_M2_48_P
Mut 6.81    iPS12_82_M2_48_P
Mut 7.09    iPS12_82_M2_48_P
"
)
Data = read.table(textConnection(Input),header=TRUE)
Data$names = factor(Data$names,ordered=FALSE, levels=unique(Data$names))
Data$block = factor(Data$block,ordered=FALSE, levels=unique(Data$block))

# SOX9_boxplot 1
boxplot(values ~ names,
        data = Data,
        ylab ="values",
        xlab ="names")
```

```
Data22 <- Data |>
  mutate(
    IQR = IQR(values, na.rm = TRUE),
    Outlier_upper = quantile(values, probs = c(.75), na.rm = TRUE) + 1.5 * IQR,
    Outlier_lower = quantile(values, probs = c(.25), na.rm = TRUE) - 1.5 * IQR,
    values_wo_outliers = if_else(values <= Outlier_lower | values >= Outlier_upper, NA, values))

boxplot(values_wo_outliers ~ names, Data22)
```

```
Data22b<- Data22|> select(block, names, values, values_wo_outliers)
Data22b
```

```
##              block names values values_wo_outliers
## 1 iPS12_82_M2_48_P   Mut   6.15               6.15
## 2 iPS12_82_M2_48_P   Mut   6.56               6.56
## 3 iPS12_82_M2_48_P   Mut   7.01               7.01
## 4 iPS12_82_M2_48_P   Mut   6.19               6.19
## 5 iPS12_82_M2_48_P   Mut   6.81               6.81
## 6 iPS12_82_M2_48_P   Mut   7.09               7.09
```

## 1.4 SOX9\_iPS19

### 1.4.1 SOX9\_iPS19-iPS-Wt

```
Input = ("
names   values  block
WT  8.73    iPS19_45_iPS
WT  7.88    iPS19_45_iPS
WT  7.92    iPS19_45_iPS
WT  7.80    iPS19_45_iPS
WT  7.60    iPS19_45_iPS
WT  7.65    iPS19_45_iPS
WT  7.12    iPS19_82_iPS
WT  8.39    iPS19_82_iPS
WT  7.88    iPS19_82_iPS
WT  9.05    iPS19_82_iPS
WT  7.92    iPS19_82_iPS
WT  7.25    iPS19_82_iPS
"
)
Data = read.table(textConnection(Input),header=TRUE)
Data$names = factor(Data$names,ordered=FALSE, levels=unique(Data$names))
Data$block = factor(Data$block,ordered=FALSE, levels=unique(Data$block))

# SRY_boxplot 1
boxplot(values ~ names,
        data = Data,
        ylab ="values",
        xlab ="names")
```

```
Data23 <- Data |>
  mutate(
    IQR = IQR(values, na.rm = TRUE),
    Outlier_upper = quantile(values, probs = c(.75), na.rm = TRUE) + 1.5 * IQR,
    Outlier_lower = quantile(values, probs = c(.25), na.rm = TRUE) - 1.5 * IQR,
    values_wo_outliers = if_else(values <= Outlier_lower | values >= Outlier_upper, NA, values))

boxplot(values_wo_outliers ~ names, Data23)
```

```
Data23b<- Data23|> select(block, names, values, values_wo_outliers)
Data23b
```

```
##           block names values values_wo_outliers
## 1  iPS19_45_iPS    WT   8.73                 NA
## 2  iPS19_45_iPS    WT   7.88               7.88
## 3  iPS19_45_iPS    WT   7.92               7.92
## 4  iPS19_45_iPS    WT   7.80               7.80
## 5  iPS19_45_iPS    WT   7.60               7.60
## 6  iPS19_45_iPS    WT   7.65               7.65
## 7  iPS19_82_iPS    WT   7.12               7.12
## 8  iPS19_82_iPS    WT   8.39               8.39
## 9  iPS19_82_iPS    WT   7.88               7.88
## 10 iPS19_82_iPS    WT   9.05                 NA
## 11 iPS19_82_iPS    WT   7.92               7.92
## 12 iPS19_82_iPS    WT   7.25               7.25
```

### 1.4.2 SOX9\_iPS19-iPS-Mut

```
Input = ("
names   values  block
Mut 7.70    iPS19_82_iPS
Mut 7.81    iPS19_82_iPS
Mut 8.32    iPS19_82_iPS
Mut 7.99    iPS19_82_iPS
Mut 8.29    iPS19_82_iPS
Mut 8.00    iPS19_82_iPS
Mut 7.79    iPS19_82_iPS
Mut 9.24    iPS19_82_iPS
Mut 8.00    iPS19_82_iPS
Mut 8.18    iPS19_82_iPS
Mut 8.35    iPS19_82_iPS
Mut 8.75    iPS19_82_iPS
"
)
Data = read.table(textConnection(Input),header=TRUE)
Data$names = factor(Data$names,ordered=FALSE, levels=unique(Data$names))
Data$block = factor(Data$block,ordered=FALSE, levels=unique(Data$block))

# SRY_boxplot 1
boxplot(values ~ names,
        data = Data,
        ylab ="values",
        xlab ="names")
```

```
Data24 <- Data |>
  mutate(
    IQR = IQR(values, na.rm = TRUE),
    Outlier_upper = quantile(values, probs = c(.75), na.rm = TRUE) + 1.5 * IQR,
    Outlier_lower = quantile(values, probs = c(.25), na.rm = TRUE) - 1.5 * IQR,
    values_wo_outliers = if_else(values <= Outlier_lower | values >= Outlier_upper, NA, values))

boxplot(values_wo_outliers ~ names, Data24)
```

```
Data24b<- Data24|> select(block, names, values, values_wo_outliers)
Data24b
```

```
##           block names values values_wo_outliers
## 1  iPS19_82_iPS   Mut   7.70               7.70
## 2  iPS19_82_iPS   Mut   7.81               7.81
## 3  iPS19_82_iPS   Mut   8.32               8.32
## 4  iPS19_82_iPS   Mut   7.99               7.99
## 5  iPS19_82_iPS   Mut   8.29               8.29
## 6  iPS19_82_iPS   Mut   8.00               8.00
## 7  iPS19_82_iPS   Mut   7.79               7.79
## 8  iPS19_82_iPS   Mut   9.24                 NA
## 9  iPS19_82_iPS   Mut   8.00               8.00
## 10 iPS19_82_iPS   Mut   8.18               8.18
## 11 iPS19_82_iPS   Mut   8.35               8.35
## 12 iPS19_82_iPS   Mut   8.75               8.75
```
