# Supplementary material for: A conserved NR5A1-responsive enhancer regulates SRY in testis-determination
Source: Nat Commun. 2024 Mar 30;15:2796. doi: 10.1038/s41467-024-47162-2 (PMC10981742; doi:10.1038/s41467-024-47162-2)
Supplement: Supplementary file 35 — Supplementary Dataset 32 [file 41467_2024_47162_MOESM35_ESM.html]

Supplementary\_Data\_32


# Supplementary\_Data\_32

#### Denis

#### 2023-07-10

RT-qPCR analysis - WNT4 - outlier identification by the IQR method in
R

```
rm(list=ls())
```

```
library(tidyverse)
```

```
## ── Attaching core tidyverse packages ──────────────────────── tidyverse 2.0.0 ──
## ✔ dplyr     1.1.4     ✔ readr     2.1.5
## ✔ forcats   1.0.0     ✔ stringr   1.5.1
## ✔ ggplot2   3.4.4     ✔ tibble    3.2.1
## ✔ lubridate 1.9.3     ✔ tidyr     1.3.0
## ✔ purrr     1.0.2     
## ── Conflicts ────────────────────────────────────────── tidyverse_conflicts() ──
## ✖ dplyr::filter() masks stats::filter()
## ✖ dplyr::lag()    masks stats::lag()
## ℹ Use the conflicted package (<http://conflicted.r-lib.org/>) to force all conflicts to become errors
```

# 1 WNT4

## 1.1 WNT4\_iPS09

### 1.1.1 WNT4\_iPS09-M1\_36h00-Wt

```
Input = ("
names   values  block
WT  10.97   iPS09-45-M1_36
WT  10.82   iPS09-45-M1_36
WT  10.60   iPS09-45-M1_36
WT  11.05   iPS09-45-M1_36
WT  10.80   iPS09-45-M1_36
"
)
Data = read.table(textConnection(Input),header=TRUE)
Data$names = factor(Data$names,ordered=FALSE, levels=unique(Data$names))
Data$block = factor(Data$block,ordered=FALSE, levels=unique(Data$block))

# WNT4_boxplot 1
boxplot(values ~ names,
        data = Data,
        ylab ="values",
        xlab ="names")
```

```
Data4 <- Data |>
  mutate(
    IQR = IQR(values, na.rm = TRUE),
    Outlier_upper = quantile(values, probs = c(.75), na.rm = TRUE) + 1.5 * IQR,
    Outlier_lower = quantile(values, probs = c(.25), na.rm = TRUE) - 1.5 * IQR,
    values_wo_outliers = if_else(values <= Outlier_lower | values >= Outlier_upper, NA, values))

boxplot(values_wo_outliers ~ names, Data4)
```

```
Data4b<- Data4|> select(block, names, values, values_wo_outliers)
Data4b
```

```
##            block names values values_wo_outliers
## 1 iPS09-45-M1_36    WT  10.97              10.97
## 2 iPS09-45-M1_36    WT  10.82              10.82
## 3 iPS09-45-M1_36    WT  10.60              10.60
## 4 iPS09-45-M1_36    WT  11.05              11.05
## 5 iPS09-45-M1_36    WT  10.80              10.80
```

### 1.1.2 WNT4\_iPS09-M1\_36h00-Mut

```
Input = ("
names   values  block
Mut 11.27   iPS09-82-M1_36
Mut 10.83   iPS09-82-M1_36
Mut 11.44   iPS09-82-M1_36
Mut 11.40   iPS09-82-M1_36
Mut 10.90   iPS09-82-M1_36
"
)
Data = read.table(textConnection(Input),header=TRUE)
Data$names = factor(Data$names,ordered=FALSE, levels=unique(Data$names))
Data$block = factor(Data$block,ordered=FALSE, levels=unique(Data$block))

# WNT4_boxplot 1
boxplot(values ~ names,
        data = Data,
        ylab ="values",
        xlab ="names")
```

```
Data5 <- Data |>
  mutate(
    IQR = IQR(values, na.rm = TRUE),
    Outlier_upper = quantile(values, probs = c(.75), na.rm = TRUE) + 1.5 * IQR,
    Outlier_lower = quantile(values, probs = c(.25), na.rm = TRUE) - 1.5 * IQR,
    values_wo_outliers = if_else(values <= Outlier_lower | values >= Outlier_upper, NA, values))

boxplot(values_wo_outliers ~ names, Data5)
```

```
Data5b<- Data5|> select(block, names, values, values_wo_outliers)
Data5b
```

```
##            block names values values_wo_outliers
## 1 iPS09-82-M1_36   Mut  11.27              11.27
## 2 iPS09-82-M1_36   Mut  10.83              10.83
## 3 iPS09-82-M1_36   Mut  11.44              11.44
## 4 iPS09-82-M1_36   Mut  11.40              11.40
## 5 iPS09-82-M1_36   Mut  10.90              10.90
```

### 1.1.3 WNT4\_iPS09-M2\_24h00-Wt

```
Input = ("
names   values  block
WT  8.64    iPS09-45-M2_24
WT  8.87    iPS09-45-M2_24
WT  8.64    iPS09-45-M2_24
WT  8.96    iPS09-45-M2_24
WT  8.94    iPS09-45-M2_24
"
)
Data = read.table(textConnection(Input),header=TRUE)
Data$names = factor(Data$names,ordered=FALSE, levels=unique(Data$names))
Data$block = factor(Data$block,ordered=FALSE, levels=unique(Data$block))

# WNT4_boxplot 1
boxplot(values ~ names,
        data = Data,
        ylab ="values",
        xlab ="names")
```

```
Data6 <- Data |>
  mutate(
    IQR = IQR(values, na.rm = TRUE),
    Outlier_upper = quantile(values, probs = c(.75), na.rm = TRUE) + 1.5 * IQR,
    Outlier_lower = quantile(values, probs = c(.25), na.rm = TRUE) - 1.5 * IQR,
    values_wo_outliers = if_else(values <= Outlier_lower | values >= Outlier_upper, NA, values))

boxplot(values_wo_outliers ~ names, Data6)
```

```
Data6b<- Data6|> select(block, names, values, values_wo_outliers)
Data6b
```

```
##            block names values values_wo_outliers
## 1 iPS09-45-M2_24    WT   8.64               8.64
## 2 iPS09-45-M2_24    WT   8.87               8.87
## 3 iPS09-45-M2_24    WT   8.64               8.64
## 4 iPS09-45-M2_24    WT   8.96               8.96
## 5 iPS09-45-M2_24    WT   8.94               8.94
```

### 1.1.4 WNT4\_iPS09-M2\_24h00-Mut

```
Input = ("
names   values  block
Mut 9.18    iPS09-82-M2_24
Mut 9.15    iPS09-82-M2_24
Mut 9.31    iPS09-82-M2_24
Mut 9.25    iPS09-82-M2_24
Mut 8.92    iPS09-82-M2_24
"
)
Data = read.table(textConnection(Input),header=TRUE)
Data$names = factor(Data$names,ordered=FALSE, levels=unique(Data$names))
Data$block = factor(Data$block,ordered=FALSE, levels=unique(Data$block))

# WNT4_boxplot 1
boxplot(values ~ names,
        data = Data,
        ylab ="values",
        xlab ="names")
```

```
Data7 <- Data |>
  mutate(
    IQR = IQR(values, na.rm = TRUE),
    Outlier_upper = quantile(values, probs = c(.75), na.rm = TRUE) + 1.5 * IQR,
    Outlier_lower = quantile(values, probs = c(.25), na.rm = TRUE) - 1.5 * IQR,
    values_wo_outliers = if_else(values <= Outlier_lower | values >= Outlier_upper, NA, values))

boxplot(values_wo_outliers ~ names, Data7)
```

```
Data7b<- Data7|> select(block, names, values, values_wo_outliers)
Data7b
```

```
##            block names values values_wo_outliers
## 1 iPS09-82-M2_24   Mut   9.18               9.18
## 2 iPS09-82-M2_24   Mut   9.15               9.15
## 3 iPS09-82-M2_24   Mut   9.31               9.31
## 4 iPS09-82-M2_24   Mut   9.25               9.25
## 5 iPS09-82-M2_24   Mut   8.92                 NA
```

### 1.1.5 WNT4\_iPS09-M3\_24h00-Wt

```
Input = ("
names   values  block
WT  9.49    iPS09-45-M3_24
WT  9.55    iPS09-45-M3_24
WT  9.94    iPS09-45-M3_24
WT  9.09    iPS09-45-M3_24
WT  14.95   iPS09-45-M3_24
"
)
Data = read.table(textConnection(Input),header=TRUE)
Data$names = factor(Data$names,ordered=FALSE, levels=unique(Data$names))
Data$block = factor(Data$block,ordered=FALSE, levels=unique(Data$block))

# WNT4_boxplot 1
boxplot(values ~ names,
        data = Data,
        ylab ="values",
        xlab ="names")
```

```
Data8 <- Data |>
  mutate(
    IQR = IQR(values, na.rm = TRUE),
    Outlier_upper = quantile(values, probs = c(.75), na.rm = TRUE) + 1.5 * IQR,
    Outlier_lower = quantile(values, probs = c(.25), na.rm = TRUE) - 1.5 * IQR,
    values_wo_outliers = if_else(values <= Outlier_lower | values >= Outlier_upper, NA, values))

boxplot(values_wo_outliers ~ names, Data8)
```

```
Data8b<- Data8|> select(block, names, values, values_wo_outliers)
Data8b
```

```
##            block names values values_wo_outliers
## 1 iPS09-45-M3_24    WT   9.49               9.49
## 2 iPS09-45-M3_24    WT   9.55               9.55
## 3 iPS09-45-M3_24    WT   9.94               9.94
## 4 iPS09-45-M3_24    WT   9.09               9.09
## 5 iPS09-45-M3_24    WT  14.95                 NA
```

### 1.1.6 WNT4\_iPS09-M3\_48h00-Wt

```
Input = ("
names   values  block
WT  14.30   iPS09-45-M3_48
WT  14.66   iPS09-45-M3_48
WT  14.76   iPS09-45-M3_48
WT  15.62   iPS09-45-M3_48
WT  15.87   iPS09-45-M3_48
"
)
Data = read.table(textConnection(Input),header=TRUE)
Data$names = factor(Data$names,ordered=FALSE, levels=unique(Data$names))
Data$block = factor(Data$block,ordered=FALSE, levels=unique(Data$block))

# WNT4_boxplot 1
boxplot(values ~ names,
        data = Data,
        ylab ="values",
        xlab ="names")
```

```
Data9 <- Data |>
  mutate(
    IQR = IQR(values, na.rm = TRUE),
    Outlier_upper = quantile(values, probs = c(.75), na.rm = TRUE) + 1.5 * IQR,
    Outlier_lower = quantile(values, probs = c(.25), na.rm = TRUE) - 1.5 * IQR,
    values_wo_outliers = if_else(values <= Outlier_lower | values >= Outlier_upper, NA, values))

boxplot(values_wo_outliers ~ names, Data9)
```

```
Data9b<- Data9|> select(block, names, values, values_wo_outliers)
Data9b
```

```
##            block names values values_wo_outliers
## 1 iPS09-45-M3_48    WT  14.30              14.30
## 2 iPS09-45-M3_48    WT  14.66              14.66
## 3 iPS09-45-M3_48    WT  14.76              14.76
## 4 iPS09-45-M3_48    WT  15.62              15.62
## 5 iPS09-45-M3_48    WT  15.87              15.87
```

### 1.1.7 WNT4\_iPS09-M3\_48h00-Mut

```
Input = ("
names   values  block
Mut 14.64   iPS09-82-M3_48
Mut 14.22   iPS09-82-M3_48
Mut 14.60   iPS09-82-M3_48
Mut 14.41   iPS09-82-M3_48
Mut 14.32   iPS09-82-M3_48
"
)
Data = read.table(textConnection(Input),header=TRUE)
Data$names = factor(Data$names,ordered=FALSE, levels=unique(Data$names))
Data$block = factor(Data$block,ordered=FALSE, levels=unique(Data$block))

# WNT4_boxplot 1
boxplot(values ~ names,
        data = Data,
        ylab ="values",
        xlab ="names")
```

```
Data10 <- Data |>
  mutate(
    IQR = IQR(values, na.rm = TRUE),
    Outlier_upper = quantile(values, probs = c(.75), na.rm = TRUE) + 1.5 * IQR,
    Outlier_lower = quantile(values, probs = c(.25), na.rm = TRUE) - 1.5 * IQR,
    values_wo_outliers = if_else(values <= Outlier_lower | values >= Outlier_upper, NA, values))

boxplot(values_wo_outliers ~ names, Data10)
```

```
Data10b<- Data10|> select(block, names, values, values_wo_outliers)
Data10b
```

```
##            block names values values_wo_outliers
## 1 iPS09-82-M3_48   Mut  14.64              14.64
## 2 iPS09-82-M3_48   Mut  14.22              14.22
## 3 iPS09-82-M3_48   Mut  14.60              14.60
## 4 iPS09-82-M3_48   Mut  14.41              14.41
## 5 iPS09-82-M3_48   Mut  14.32              14.32
```

## 1.2 WNT4\_iPS12

### 1.2.1 WNT4\_iPS12-M1\_36h00-Wt

```
Input = ("
names   values  block
WT  13.47   iPS12_45_M1_36_P
WT  12.85   iPS12_45_M1_36_P
WT  13.40   iPS12_45_M1_36_P
WT  13.26   iPS12_45_M1_36_P
WT  13.74   iPS12_45_M1_36_P
WT  13.00   iPS12_45_M1_36_P
"
)
Data = read.table(textConnection(Input),header=TRUE)
Data$names = factor(Data$names,ordered=FALSE, levels=unique(Data$names))
Data$block = factor(Data$block,ordered=FALSE, levels=unique(Data$block))

# WNT4_boxplot 1
boxplot(values ~ names,
        data = Data,
        ylab ="values",
        xlab ="names")
```

```
Data13 <- Data |>
  mutate(
    IQR = IQR(values, na.rm = TRUE),
    Outlier_upper = quantile(values, probs = c(.75), na.rm = TRUE) + 1.5 * IQR,
    Outlier_lower = quantile(values, probs = c(.25), na.rm = TRUE) - 1.5 * IQR,
    values_wo_outliers = if_else(values <= Outlier_lower | values >= Outlier_upper, NA, values))

boxplot(values_wo_outliers ~ names, Data13)
```

```
Data13b<- Data13|> select(block, names, values, values_wo_outliers)
Data13b
```

```
##              block names values values_wo_outliers
## 1 iPS12_45_M1_36_P    WT  13.47              13.47
## 2 iPS12_45_M1_36_P    WT  12.85              12.85
## 3 iPS12_45_M1_36_P    WT  13.40              13.40
## 4 iPS12_45_M1_36_P    WT  13.26              13.26
## 5 iPS12_45_M1_36_P    WT  13.74              13.74
## 6 iPS12_45_M1_36_P    WT  13.00              13.00
```

### 1.2.2 WNT4\_iPS12-M1\_36h00-Mut

```
Input = ("
names   values  block
Mut 13.49   iPS12_82_M1_36_P
Mut 13.28   iPS12_82_M1_36_P
Mut 14.93   iPS12_82_M1_36_P
Mut 13.96   iPS12_82_M1_36_P
Mut 13.43   iPS12_82_M1_36_P
Mut 13.52   iPS12_82_M1_36_P
"
)
Data = read.table(textConnection(Input),header=TRUE)
Data$names = factor(Data$names,ordered=FALSE, levels=unique(Data$names))
Data$block = factor(Data$block,ordered=FALSE, levels=unique(Data$block))

# WNT4_boxplot 1
boxplot(values ~ names,
        data = Data,
        ylab ="values",
        xlab ="names")
```

```
Data14 <- Data |>
  mutate(
    IQR = IQR(values, na.rm = TRUE),
    Outlier_upper = quantile(values, probs = c(.75), na.rm = TRUE) + 1.5 * IQR,
    Outlier_lower = quantile(values, probs = c(.25), na.rm = TRUE) - 1.5 * IQR,
    values_wo_outliers = if_else(values <= Outlier_lower | values >= Outlier_upper, NA, values))

boxplot(values_wo_outliers ~ names, Data14)
```

```
Data14b<- Data14|> select(block, names, values, values_wo_outliers)
Data14b
```

```
##              block names values values_wo_outliers
## 1 iPS12_82_M1_36_P   Mut  13.49              13.49
## 2 iPS12_82_M1_36_P   Mut  13.28              13.28
## 3 iPS12_82_M1_36_P   Mut  14.93                 NA
## 4 iPS12_82_M1_36_P   Mut  13.96              13.96
## 5 iPS12_82_M1_36_P   Mut  13.43              13.43
## 6 iPS12_82_M1_36_P   Mut  13.52              13.52
```

### 1.2.3 WNT4\_iPS12-M2\_06h00-Wt

```
Input = ("
names   values  block
WT  10.08   iPS12_45_M2_06_P
WT  10.17   iPS12_45_M2_06_P
WT  10.18   iPS12_45_M2_06_P
WT  10.46   iPS12_45_M2_06_P
WT  10.85   iPS12_45_M2_06_P
WT  NA  iPS12_45_M2_06_P
"
)
Data = read.table(textConnection(Input),header=TRUE)
Data$names = factor(Data$names,ordered=FALSE, levels=unique(Data$names))
Data$block = factor(Data$block,ordered=FALSE, levels=unique(Data$block))

# WNT4_boxplot 1
boxplot(values ~ names,
        data = Data,
        ylab ="values",
        xlab ="names")
```

```
Data15 <- Data |>
  mutate(
    IQR = IQR(values, na.rm = TRUE),
    Outlier_upper = quantile(values, probs = c(.75), na.rm = TRUE) + 1.5 * IQR,
    Outlier_lower = quantile(values, probs = c(.25), na.rm = TRUE) - 1.5 * IQR,
    values_wo_outliers = if_else(values <= Outlier_lower | values >= Outlier_upper, NA, values))

boxplot(values_wo_outliers ~ names, Data15)
```

```
Data15b<- Data15|> select(block, names, values, values_wo_outliers)
Data15b
```

```
##              block names values values_wo_outliers
## 1 iPS12_45_M2_06_P    WT  10.08              10.08
## 2 iPS12_45_M2_06_P    WT  10.17              10.17
## 3 iPS12_45_M2_06_P    WT  10.18              10.18
## 4 iPS12_45_M2_06_P    WT  10.46              10.46
## 5 iPS12_45_M2_06_P    WT  10.85              10.85
## 6 iPS12_45_M2_06_P    WT     NA                 NA
```

### 1.2.4 WNT4\_iPS12-M2\_06h00-Mut

```
Input = ("
names   values  block
Mut NA  iPS12_82_M2_06_P
Mut 9.83    iPS12_82_M2_06_P
Mut 11.24   iPS12_82_M2_06_P
Mut 10.90   iPS12_82_M2_06_P
Mut 11.65   iPS12_82_M2_06_P
Mut 11.10   iPS12_82_M2_06_P
"
)
Data = read.table(textConnection(Input),header=TRUE)
Data$names = factor(Data$names,ordered=FALSE, levels=unique(Data$names))
Data$block = factor(Data$block,ordered=FALSE, levels=unique(Data$block))

# WNT4_boxplot 1
boxplot(values ~ names,
        data = Data,
        ylab ="values",
        xlab ="names")
```

```
Data16 <- Data |>
  mutate(
    IQR = IQR(values, na.rm = TRUE),
    Outlier_upper = quantile(values, probs = c(.75), na.rm = TRUE) + 1.5 * IQR,
    Outlier_lower = quantile(values, probs = c(.25), na.rm = TRUE) - 1.5 * IQR,
    values_wo_outliers = if_else(values <= Outlier_lower | values >= Outlier_upper, NA, values))

boxplot(values_wo_outliers ~ names, Data16)
```

```
Data16b<- Data16|> select(block, names, values, values_wo_outliers)
Data16b
```

```
##              block names values values_wo_outliers
## 1 iPS12_82_M2_06_P   Mut     NA                 NA
## 2 iPS12_82_M2_06_P   Mut   9.83                 NA
## 3 iPS12_82_M2_06_P   Mut  11.24              11.24
## 4 iPS12_82_M2_06_P   Mut  10.90              10.90
## 5 iPS12_82_M2_06_P   Mut  11.65              11.65
## 6 iPS12_82_M2_06_P   Mut  11.10              11.10
```

### 1.2.5 WNT4\_iPS12-M2\_12h00-Wt\_vs\_Mut

```
Input = ("
names   values  block
WT  15.62   iPS12_45_M2_12_P
WT  12.93   iPS12_45_M2_12_P
WT  12.40   iPS12_45_M2_12_P
WT  13.82   iPS12_45_M2_12_P
WT  12.60   iPS12_45_M2_12_P
WT  12.96   iPS12_45_M2_12_P
"
)
Data = read.table(textConnection(Input),header=TRUE)
Data$names = factor(Data$names,ordered=FALSE, levels=unique(Data$names))
Data$block = factor(Data$block,ordered=FALSE, levels=unique(Data$block))

# WNT4_boxplot 1
boxplot(values ~ names,
        data = Data,
        ylab ="values",
        xlab ="names")
```

```
Data17 <- Data |>
  mutate(
    IQR = IQR(values, na.rm = TRUE),
    Outlier_upper = quantile(values, probs = c(.75), na.rm = TRUE) + 1.5 * IQR,
    Outlier_lower = quantile(values, probs = c(.25), na.rm = TRUE) - 1.5 * IQR,
    values_wo_outliers = if_else(values <= Outlier_lower | values >= Outlier_upper, NA, values))

boxplot(values_wo_outliers ~ names, Data17)
```

```
Data17b<- Data17|> select(block, names, values, values_wo_outliers)
Data17b
```

```
##              block names values values_wo_outliers
## 1 iPS12_45_M2_12_P    WT  15.62                 NA
## 2 iPS12_45_M2_12_P    WT  12.93              12.93
## 3 iPS12_45_M2_12_P    WT  12.40              12.40
## 4 iPS12_45_M2_12_P    WT  13.82              13.82
## 5 iPS12_45_M2_12_P    WT  12.60              12.60
## 6 iPS12_45_M2_12_P    WT  12.96              12.96
```

### 1.2.6 WNT4\_iPS12-M2\_12h00-Mut

```
Input = ("
names   values  block
Mut 13.32   iPS12_82_M2_12_P
Mut 12.78   iPS12_82_M2_12_P
Mut 15.30   iPS12_82_M2_12_P
Mut 13.45   iPS12_82_M2_12_P
Mut 13.74   iPS12_82_M2_12_P
Mut 14.86   iPS12_82_M2_12_P
"
)
Data = read.table(textConnection(Input),header=TRUE)
Data$names = factor(Data$names,ordered=FALSE, levels=unique(Data$names))
Data$block = factor(Data$block,ordered=FALSE, levels=unique(Data$block))

# WNT4_boxplot 1
boxplot(values ~ names,
        data = Data,
        ylab ="values",
        xlab ="names")
```

```
Data18 <- Data |>
  mutate(
    IQR = IQR(values, na.rm = TRUE),
    Outlier_upper = quantile(values, probs = c(.75), na.rm = TRUE) + 1.5 * IQR,
    Outlier_lower = quantile(values, probs = c(.25), na.rm = TRUE) - 1.5 * IQR,
    values_wo_outliers = if_else(values <= Outlier_lower | values >= Outlier_upper, NA, values))

boxplot(values_wo_outliers ~ names, Data18)
```

```
Data18b<- Data18|> select(block, names, values, values_wo_outliers)
Data18b
```

```
##              block names values values_wo_outliers
## 1 iPS12_82_M2_12_P   Mut  13.32              13.32
## 2 iPS12_82_M2_12_P   Mut  12.78              12.78
## 3 iPS12_82_M2_12_P   Mut  15.30              15.30
## 4 iPS12_82_M2_12_P   Mut  13.45              13.45
## 5 iPS12_82_M2_12_P   Mut  13.74              13.74
## 6 iPS12_82_M2_12_P   Mut  14.86              14.86
```

### 1.2.7 WNT4\_iPS12\_M2\_24h00-Wt

```
Input = ("
names   values  block
WT  8.60    iPS12_45_M2_24_P
WT  9.67    iPS12_45_M2_24_P
WT  9.08    iPS12_45_M2_24_P
WT  9.42    iPS12_45_M2_24_P
WT  9.34    iPS12_45_M2_24_P
WT  11.92   iPS12_45_M2_24_P
"
)
Data = read.table(textConnection(Input),header=TRUE)
Data$names = factor(Data$names,ordered=FALSE, levels=unique(Data$names))
Data$block = factor(Data$block,ordered=FALSE, levels=unique(Data$block))

# WNT4_boxplot 1
boxplot(values ~ names,
        data = Data,
        ylab ="values",
        xlab ="names")
```

```
Data19 <- Data |>
  mutate(
    IQR = IQR(values, na.rm = TRUE),
    Outlier_upper = quantile(values, probs = c(.75), na.rm = TRUE) + 1.5 * IQR,
    Outlier_lower = quantile(values, probs = c(.25), na.rm = TRUE) - 1.5 * IQR,
    values_wo_outliers = if_else(values <= Outlier_lower | values >= Outlier_upper, NA, values))

boxplot(values_wo_outliers ~ names, Data19)
```

```
Data19b<- Data19|> select(block, names, values, values_wo_outliers)
Data19b
```

```
##              block names values values_wo_outliers
## 1 iPS12_45_M2_24_P    WT   8.60               8.60
## 2 iPS12_45_M2_24_P    WT   9.67               9.67
## 3 iPS12_45_M2_24_P    WT   9.08               9.08
## 4 iPS12_45_M2_24_P    WT   9.42               9.42
## 5 iPS12_45_M2_24_P    WT   9.34               9.34
## 6 iPS12_45_M2_24_P    WT  11.92                 NA
```

### 1.2.8 WNT4\_iPS12\_M2\_24h00-Mut

```
Input = ("
names   values  block
Mut 9.73    iPS12_82_M2_24_P
Mut 9.64    iPS12_82_M2_24_P
Mut 9.57    iPS12_82_M2_24_P
Mut 8.87    iPS12_82_M2_24_P
Mut 9.14    iPS12_82_M2_24_P
Mut 10.31   iPS12_82_M2_24_P
"
)
Data = read.table(textConnection(Input),header=TRUE)
Data$names = factor(Data$names,ordered=FALSE, levels=unique(Data$names))
Data$block = factor(Data$block,ordered=FALSE, levels=unique(Data$block))

# WNT4_boxplot 1
boxplot(values ~ names,
        data = Data,
        ylab ="values",
        xlab ="names")
```

```
Data20 <- Data |>
  mutate(
    IQR = IQR(values, na.rm = TRUE),
    Outlier_upper = quantile(values, probs = c(.75), na.rm = TRUE) + 1.5 * IQR,
    Outlier_lower = quantile(values, probs = c(.25), na.rm = TRUE) - 1.5 * IQR,
    values_wo_outliers = if_else(values <= Outlier_lower | values >= Outlier_upper, NA, values))

boxplot(values_wo_outliers ~ names, Data20)
```

```
Data20b<- Data20|> select(block, names, values, values_wo_outliers)
Data20b
```

```
##              block names values values_wo_outliers
## 1 iPS12_82_M2_24_P   Mut   9.73               9.73
## 2 iPS12_82_M2_24_P   Mut   9.64               9.64
## 3 iPS12_82_M2_24_P   Mut   9.57               9.57
## 4 iPS12_82_M2_24_P   Mut   8.87               8.87
## 5 iPS12_82_M2_24_P   Mut   9.14               9.14
## 6 iPS12_82_M2_24_P   Mut  10.31              10.31
```

### 1.2.9 WNT4\_iPS12-M2\_48h00-Wt

```
Input = ("
names   values  block
WT  9.58    iPS12_45_M2_48_P
WT  8.84    iPS12_45_M2_48_P
WT  9.33    iPS12_45_M2_48_P
WT  8.35    iPS12_45_M2_48_P
WT  8.84    iPS12_45_M2_48_P
WT  NA  iPS12_45_M2_48_P
"
)
Data = read.table(textConnection(Input),header=TRUE)
Data$names = factor(Data$names,ordered=FALSE, levels=unique(Data$names))
Data$block = factor(Data$block,ordered=FALSE, levels=unique(Data$block))

# WNT4_boxplot 1
boxplot(values ~ names,
        data = Data,
        ylab ="values",
        xlab ="names")
```

```
Data21 <- Data |>
  mutate(
    IQR = IQR(values, na.rm = TRUE),
    Outlier_upper = quantile(values, probs = c(.75), na.rm = TRUE) + 1.5 * IQR,
    Outlier_lower = quantile(values, probs = c(.25), na.rm = TRUE) - 1.5 * IQR,
    values_wo_outliers = if_else(values <= Outlier_lower | values >= Outlier_upper, NA, values))

boxplot(values_wo_outliers ~ names, Data21)
```

```
Data21b<- Data21|> select(block, names, values, values_wo_outliers)
Data21b
```

```
##              block names values values_wo_outliers
## 1 iPS12_45_M2_48_P    WT   9.58               9.58
## 2 iPS12_45_M2_48_P    WT   8.84               8.84
## 3 iPS12_45_M2_48_P    WT   9.33               9.33
## 4 iPS12_45_M2_48_P    WT   8.35               8.35
## 5 iPS12_45_M2_48_P    WT   8.84               8.84
## 6 iPS12_45_M2_48_P    WT     NA                 NA
```

### 1.2.10 WNT4\_iPS12-M2\_48h00-Mut

```
Input = ("
names   values  block
Mut 8.84    iPS12_82_M2_48_P
Mut 9.39    iPS12_82_M2_48_P
Mut 9.38    iPS12_82_M2_48_P
Mut 10.07   iPS12_82_M2_48_P
Mut 9.91    iPS12_82_M2_48_P
Mut NA  iPS12_82_M2_48_P
"
)
Data = read.table(textConnection(Input),header=TRUE)
Data$names = factor(Data$names,ordered=FALSE, levels=unique(Data$names))
Data$block = factor(Data$block,ordered=FALSE, levels=unique(Data$block))

# WNT4_boxplot 1
boxplot(values ~ names,
        data = Data,
        ylab ="values",
        xlab ="names")
```

```
Data22 <- Data |>
  mutate(
    IQR = IQR(values, na.rm = TRUE),
    Outlier_upper = quantile(values, probs = c(.75), na.rm = TRUE) + 1.5 * IQR,
    Outlier_lower = quantile(values, probs = c(.25), na.rm = TRUE) - 1.5 * IQR,
    values_wo_outliers = if_else(values <= Outlier_lower | values >= Outlier_upper, NA, values))

boxplot(values_wo_outliers ~ names, Data22)
```

```
Data22b<- Data22|> select(block, names, values, values_wo_outliers)
Data22b
```

```
##              block names values values_wo_outliers
## 1 iPS12_82_M2_48_P   Mut   8.84               8.84
## 2 iPS12_82_M2_48_P   Mut   9.39               9.39
## 3 iPS12_82_M2_48_P   Mut   9.38               9.38
## 4 iPS12_82_M2_48_P   Mut  10.07              10.07
## 5 iPS12_82_M2_48_P   Mut   9.91               9.91
## 6 iPS12_82_M2_48_P   Mut     NA                 NA
```

## 1.3 WNT4\_iPS19

### 1.3.1 WNT4\_iPS19-iPS-Wt

```
Input = ("
names   values  block
WT  13.09   iPS19_45_iPS
WT  10.82   iPS19_45_iPS
WT  11.54   iPS19_45_iPS
WT  12.11   iPS19_45_iPS
WT  11.44   iPS19_45_iPS
WT  11.38   iPS19_45_iPS
WT  10.97   iPS19_82_iPS
WT  12.16   iPS19_82_iPS
WT  11.47   iPS19_82_iPS
WT  13.66   iPS19_82_iPS
WT  12.86   iPS19_82_iPS
WT  11.19   iPS19_82_iPS
"
)
Data = read.table(textConnection(Input),header=TRUE)
Data$names = factor(Data$names,ordered=FALSE, levels=unique(Data$names))
Data$block = factor(Data$block,ordered=FALSE, levels=unique(Data$block))

# SRY_boxplot 1
boxplot(values ~ names,
        data = Data,
        ylab ="values",
        xlab ="names")
```

```
Data23 <- Data |>
  mutate(
    IQR = IQR(values, na.rm = TRUE),
    Outlier_upper = quantile(values, probs = c(.75), na.rm = TRUE) + 1.5 * IQR,
    Outlier_lower = quantile(values, probs = c(.25), na.rm = TRUE) - 1.5 * IQR,
    values_wo_outliers = if_else(values <= Outlier_lower | values >= Outlier_upper, NA, values))

boxplot(values_wo_outliers ~ names, Data23)
```

```
Data23b<- Data23|> select(block, names, values, values_wo_outliers)
Data23b
```

```
##           block names values values_wo_outliers
## 1  iPS19_45_iPS    WT  13.09              13.09
## 2  iPS19_45_iPS    WT  10.82              10.82
## 3  iPS19_45_iPS    WT  11.54              11.54
## 4  iPS19_45_iPS    WT  12.11              12.11
## 5  iPS19_45_iPS    WT  11.44              11.44
## 6  iPS19_45_iPS    WT  11.38              11.38
## 7  iPS19_82_iPS    WT  10.97              10.97
## 8  iPS19_82_iPS    WT  12.16              12.16
## 9  iPS19_82_iPS    WT  11.47              11.47
## 10 iPS19_82_iPS    WT  13.66              13.66
## 11 iPS19_82_iPS    WT  12.86              12.86
## 12 iPS19_82_iPS    WT  11.19              11.19
```

### 1.3.2 WNT4\_iPS19-iPS-Mut

```
Input = ("
names   values  block
Mut 12.39   iPS19_82_iPS
Mut 12.76   iPS19_82_iPS
Mut 12.66   iPS19_82_iPS
Mut 12.07   iPS19_82_iPS
Mut 12.81   iPS19_82_iPS
Mut 12.60   iPS19_82_iPS
Mut 12.46   iPS19_82_iPS
Mut 13.96   iPS19_82_iPS
Mut 13.22   iPS19_82_iPS
Mut 13.66   iPS19_82_iPS
Mut 13.82   iPS19_82_iPS
Mut 14.12   iPS19_82_iPS
"
)
Data = read.table(textConnection(Input),header=TRUE)
Data$names = factor(Data$names,ordered=FALSE, levels=unique(Data$names))
Data$block = factor(Data$block,ordered=FALSE, levels=unique(Data$block))

# SRY_boxplot 1
boxplot(values ~ names,
        data = Data,
        ylab ="values",
        xlab ="names")
```

```
Data24 <- Data |>
  mutate(
    IQR = IQR(values, na.rm = TRUE),
    Outlier_upper = quantile(values, probs = c(.75), na.rm = TRUE) + 1.5 * IQR,
    Outlier_lower = quantile(values, probs = c(.25), na.rm = TRUE) - 1.5 * IQR,
    values_wo_outliers = if_else(values <= Outlier_lower | values >= Outlier_upper, NA, values))

boxplot(values_wo_outliers ~ names, Data24)
```

```
Data24b<- Data24|> select(block, names, values, values_wo_outliers)
Data24b
```

```
##           block names values values_wo_outliers
## 1  iPS19_82_iPS   Mut  12.39              12.39
## 2  iPS19_82_iPS   Mut  12.76              12.76
## 3  iPS19_82_iPS   Mut  12.66              12.66
## 4  iPS19_82_iPS   Mut  12.07              12.07
## 5  iPS19_82_iPS   Mut  12.81              12.81
## 6  iPS19_82_iPS   Mut  12.60              12.60
## 7  iPS19_82_iPS   Mut  12.46              12.46
## 8  iPS19_82_iPS   Mut  13.96              13.96
## 9  iPS19_82_iPS   Mut  13.22              13.22
## 10 iPS19_82_iPS   Mut  13.66              13.66
## 11 iPS19_82_iPS   Mut  13.82              13.82
## 12 iPS19_82_iPS   Mut  14.12              14.12
```
