# Supplementary material for: A conserved NR5A1-responsive enhancer regulates SRY in testis-determination
Source: Nat Commun. 2024 Mar 30;15:2796. doi: 10.1038/s41467-024-47162-2 (PMC10981742; doi:10.1038/s41467-024-47162-2)
Supplement: Supplementary file 36 — Supplementary Dataset 33 [file 41467_2024_47162_MOESM36_ESM.html]

Supplementary\_Data\_33


# Supplementary\_Data\_33

#### Denis

#### 2023-07-11

RT-qPCR analysis - NR5A1 - outlier identification by the IQR method
in R

```
rm(list=ls())
```

```
library(tidyverse)
```

```
## ── Attaching core tidyverse packages ──────────────────────── tidyverse 2.0.0 ──
## ✔ dplyr     1.1.4     ✔ readr     2.1.5
## ✔ forcats   1.0.0     ✔ stringr   1.5.1
## ✔ ggplot2   3.4.4     ✔ tibble    3.2.1
## ✔ lubridate 1.9.3     ✔ tidyr     1.3.0
## ✔ purrr     1.0.2     
## ── Conflicts ────────────────────────────────────────── tidyverse_conflicts() ──
## ✖ dplyr::filter() masks stats::filter()
## ✖ dplyr::lag()    masks stats::lag()
## ℹ Use the conflicted package (<http://conflicted.r-lib.org/>) to force all conflicts to become errors
```

# 1 NR5A1

## 1.1 NR5A1\_iPS07

### 1.1.1 NR5A1\_iPS07-M1\_36h00-Mut

```
Input = ("
names   values  block
Mut 14.46   iPS07-82-M1-1
Mut 15.10   iPS07-82-M1-2
Mut 14.46   iPS07-82-M1-3
Mut 13.82   iPS07-82-M1-4
Mut 14.34   iPS07-82-M1-5
"
)
Data = read.table(textConnection(Input),header=TRUE)
Data$names = factor(Data$names,ordered=FALSE, levels=unique(Data$names))
Data$block = factor(Data$block,ordered=FALSE, levels=unique(Data$block))

# NR5A1_boxplot 1
boxplot(values ~ names,
        data = Data,
        ylab ="values",
        xlab ="names")
```

```
Data1 <- Data |>
  mutate(
    IQR = IQR(values, na.rm = TRUE),
    Outlier_upper = quantile(values, probs = c(.75), na.rm = TRUE) + 1.5 * IQR,
    Outlier_lower = quantile(values, probs = c(.25), na.rm = TRUE) - 1.5 * IQR,
    values_wo_outliers = if_else(values <= Outlier_lower | values >= Outlier_upper, NA, values))

boxplot(values_wo_outliers ~ names, Data1)
```

```
Data1b<- Data1|> select(block, names, values, values_wo_outliers)
Data1b
```

```
##           block names values values_wo_outliers
## 1 iPS07-82-M1-1   Mut  14.46              14.46
## 2 iPS07-82-M1-2   Mut  15.10                 NA
## 3 iPS07-82-M1-3   Mut  14.46              14.46
## 4 iPS07-82-M1-4   Mut  13.82                 NA
## 5 iPS07-82-M1-5   Mut  14.34              14.34
```

### 1.1.2 NR5A1\_iPS07-M2\_48h00-Wt

```
Input = ("
names   values  block
WT  13.81   iPS07-45-M2_48
WT  13.89   iPS07-45-M2_48
WT  13.26   iPS07-45-M2_48
WT  13.65   iPS07-45-M2_48
WT  13.75   iPS07-45-M2_48
"
)
Data = read.table(textConnection(Input),header=TRUE)
Data$names = factor(Data$names,ordered=FALSE, levels=unique(Data$names))
Data$block = factor(Data$block,ordered=FALSE, levels=unique(Data$block))

# NR5A1_boxplot 1
boxplot(values ~ names,
        data = Data,
        ylab ="values",
        xlab ="names")
```

```
Data2 <- Data |>
  mutate(
    IQR = IQR(values, na.rm = TRUE),
    Outlier_upper = quantile(values, probs = c(.75), na.rm = TRUE) + 1.5 * IQR,
    Outlier_lower = quantile(values, probs = c(.25), na.rm = TRUE) - 1.5 * IQR,
    values_wo_outliers = if_else(values <= Outlier_lower | values >= Outlier_upper, NA, values))

boxplot(values_wo_outliers ~ names, Data2)
```

```
Data2b<- Data2|> select(block, names, values, values_wo_outliers)
Data2b
```

```
##            block names values values_wo_outliers
## 1 iPS07-45-M2_48    WT  13.81              13.81
## 2 iPS07-45-M2_48    WT  13.89              13.89
## 3 iPS07-45-M2_48    WT  13.26                 NA
## 4 iPS07-45-M2_48    WT  13.65              13.65
## 5 iPS07-45-M2_48    WT  13.75              13.75
```

### 1.1.3 NR5A1\_iPS07-M2\_48h00-Mut

```
Input = ("
names   values  block
Mut 14.73   iPS07-82-M2_48
Mut 15.44   iPS07-82-M2_48
Mut 14.87   iPS07-82-M2_48
Mut 14.74   iPS07-82-M2_48
Mut 15.65   iPS07-82-M2_48
"
)
Data = read.table(textConnection(Input),header=TRUE)
Data$names = factor(Data$names,ordered=FALSE, levels=unique(Data$names))
Data$block = factor(Data$block,ordered=FALSE, levels=unique(Data$block))

# NR5A1_boxplot 1
boxplot(values ~ names,
        data = Data,
        ylab ="values",
        xlab ="names")
```

```
Data3 <- Data |>
  mutate(
    IQR = IQR(values, na.rm = TRUE),
    Outlier_upper = quantile(values, probs = c(.75), na.rm = TRUE) + 1.5 * IQR,
    Outlier_lower = quantile(values, probs = c(.25), na.rm = TRUE) - 1.5 * IQR,
    values_wo_outliers = if_else(values <= Outlier_lower | values >= Outlier_upper, NA, values))

boxplot(values_wo_outliers ~ names, Data3)
```

```
Data3b<- Data3|> select(block, names, values, values_wo_outliers)
Data3b
```

```
##            block names values values_wo_outliers
## 1 iPS07-82-M2_48   Mut  14.73              14.73
## 2 iPS07-82-M2_48   Mut  15.44              15.44
## 3 iPS07-82-M2_48   Mut  14.87              14.87
## 4 iPS07-82-M2_48   Mut  14.74              14.74
## 5 iPS07-82-M2_48   Mut  15.65              15.65
```

## 1.2 NR5A1\_iPS09

### 1.2.1 NR5A1\_iPS09-M1\_36h00-Wt

```
Input = ("
names   values  block
WT  14.30   iPS09-45-M1_36
WT  14.15   iPS09-45-M1_36
WT  13.34   iPS09-45-M1_36
WT  14.14   iPS09-45-M1_36
WT  14.19   iPS09-45-M1_36
"
)
Data = read.table(textConnection(Input),header=TRUE)
Data$names = factor(Data$names,ordered=FALSE, levels=unique(Data$names))
Data$block = factor(Data$block,ordered=FALSE, levels=unique(Data$block))

# NR5A1_boxplot 1
boxplot(values ~ names,
        data = Data,
        ylab ="values",
        xlab ="names")
```

```
Data4 <- Data |>
  mutate(
    IQR = IQR(values, na.rm = TRUE),
    Outlier_upper = quantile(values, probs = c(.75), na.rm = TRUE) + 1.5 * IQR,
    Outlier_lower = quantile(values, probs = c(.25), na.rm = TRUE) - 1.5 * IQR,
    values_wo_outliers = if_else(values <= Outlier_lower | values >= Outlier_upper, NA, values))

boxplot(values_wo_outliers ~ names, Data4)
```

```
Data4b<- Data4|> select(block, names, values, values_wo_outliers)
Data4b
```

```
##            block names values values_wo_outliers
## 1 iPS09-45-M1_36    WT  14.30                 NA
## 2 iPS09-45-M1_36    WT  14.15              14.15
## 3 iPS09-45-M1_36    WT  13.34                 NA
## 4 iPS09-45-M1_36    WT  14.14              14.14
## 5 iPS09-45-M1_36    WT  14.19              14.19
```

### 1.2.2 NR5A1\_iPS09-M1\_36h00-Mut

```
Input = ("
names   values  block
Mut 13.88   iPS09-82-M1_36
Mut 13.46   iPS09-82-M1_36
Mut 13.88   iPS09-82-M1_36
Mut 13.26   iPS09-82-M1_36
Mut 13.91   iPS09-82-M1_36
"
)
Data = read.table(textConnection(Input),header=TRUE)
Data$names = factor(Data$names,ordered=FALSE, levels=unique(Data$names))
Data$block = factor(Data$block,ordered=FALSE, levels=unique(Data$block))

# NR5A1_boxplot 1
boxplot(values ~ names,
        data = Data,
        ylab ="values",
        xlab ="names")
```

```
Data5 <- Data |>
  mutate(
    IQR = IQR(values, na.rm = TRUE),
    Outlier_upper = quantile(values, probs = c(.75), na.rm = TRUE) + 1.5 * IQR,
    Outlier_lower = quantile(values, probs = c(.25), na.rm = TRUE) - 1.5 * IQR,
    values_wo_outliers = if_else(values <= Outlier_lower | values >= Outlier_upper, NA, values))

boxplot(values_wo_outliers ~ names, Data5)
```

```
Data5b<- Data5|> select(block, names, values, values_wo_outliers)
Data5b
```

```
##            block names values values_wo_outliers
## 1 iPS09-82-M1_36   Mut  13.88              13.88
## 2 iPS09-82-M1_36   Mut  13.46              13.46
## 3 iPS09-82-M1_36   Mut  13.88              13.88
## 4 iPS09-82-M1_36   Mut  13.26              13.26
## 5 iPS09-82-M1_36   Mut  13.91              13.91
```

### 1.2.3 NR5A1\_iPS09-M2\_24h00-Wt

```
Input = ("
names   values  block
WT  11.99   iPS09-45-M2_24
WT  11.76   iPS09-45-M2_24
WT  11.79   iPS09-45-M2_24
WT  11.78   iPS09-45-M2_24
WT  11.36   iPS09-45-M2_24
"
)
Data = read.table(textConnection(Input),header=TRUE)
Data$names = factor(Data$names,ordered=FALSE, levels=unique(Data$names))
Data$block = factor(Data$block,ordered=FALSE, levels=unique(Data$block))

# NR5A1_boxplot 1
boxplot(values ~ names,
        data = Data,
        ylab ="values",
        xlab ="names")
```

```
Data6 <- Data |>
  mutate(
    IQR = IQR(values, na.rm = TRUE),
    Outlier_upper = quantile(values, probs = c(.75), na.rm = TRUE) + 1.5 * IQR,
    Outlier_lower = quantile(values, probs = c(.25), na.rm = TRUE) - 1.5 * IQR,
    values_wo_outliers = if_else(values <= Outlier_lower | values >= Outlier_upper, NA, values))

boxplot(values_wo_outliers ~ names, Data6)
```

```
Data6b<- Data6|> select(block, names, values, values_wo_outliers)
Data6b
```

```
##            block names values values_wo_outliers
## 1 iPS09-45-M2_24    WT  11.99                 NA
## 2 iPS09-45-M2_24    WT  11.76              11.76
## 3 iPS09-45-M2_24    WT  11.79              11.79
## 4 iPS09-45-M2_24    WT  11.78              11.78
## 5 iPS09-45-M2_24    WT  11.36                 NA
```

### 1.2.4 NR5A1\_iPS09-M2\_24h00-Mut

```
Input = ("
names   values  block
Mut 11.51   iPS09-82-M2_24
Mut 11.55   iPS09-82-M2_24
Mut 11.54   iPS09-82-M2_24
Mut 11.46   iPS09-82-M2_24
Mut 11.58   iPS09-82-M2_24
"
)
Data = read.table(textConnection(Input),header=TRUE)
Data$names = factor(Data$names,ordered=FALSE, levels=unique(Data$names))
Data$block = factor(Data$block,ordered=FALSE, levels=unique(Data$block))

# NR5A1_boxplot 1
boxplot(values ~ names,
        data = Data,
        ylab ="values",
        xlab ="names")
```

```
Data7 <- Data |>
  mutate(
    IQR = IQR(values, na.rm = TRUE),
    Outlier_upper = quantile(values, probs = c(.75), na.rm = TRUE) + 1.5 * IQR,
    Outlier_lower = quantile(values, probs = c(.25), na.rm = TRUE) - 1.5 * IQR,
    values_wo_outliers = if_else(values <= Outlier_lower | values >= Outlier_upper, NA, values))

boxplot(values_wo_outliers ~ names, Data7)
```

```
Data7b<- Data7|> select(block, names, values, values_wo_outliers)
Data7b
```

```
##            block names values values_wo_outliers
## 1 iPS09-82-M2_24   Mut  11.51              11.51
## 2 iPS09-82-M2_24   Mut  11.55              11.55
## 3 iPS09-82-M2_24   Mut  11.54              11.54
## 4 iPS09-82-M2_24   Mut  11.46              11.46
## 5 iPS09-82-M2_24   Mut  11.58              11.58
```

### 1.2.5 NR5A1\_iPS09-M3\_24h00-Wt

```
Input = ("
names   values  block
WT  13.23   iPS09-45-M3_24
WT  13.60   iPS09-45-M3_24
WT  14.70   iPS09-45-M3_24
WT  11.88   iPS09-45-M3_24
WT  17.41   iPS09-45-M3_24
"
)
Data = read.table(textConnection(Input),header=TRUE)
Data$names = factor(Data$names,ordered=FALSE, levels=unique(Data$names))
Data$block = factor(Data$block,ordered=FALSE, levels=unique(Data$block))

# NR5A1_boxplot 1
boxplot(values ~ names,
        data = Data,
        ylab ="values",
        xlab ="names")
```

```
Data8 <- Data |>
  mutate(
    IQR = IQR(values, na.rm = TRUE),
    Outlier_upper = quantile(values, probs = c(.75), na.rm = TRUE) + 1.5 * IQR,
    Outlier_lower = quantile(values, probs = c(.25), na.rm = TRUE) - 1.5 * IQR,
    values_wo_outliers = if_else(values <= Outlier_lower | values >= Outlier_upper, NA, values))

boxplot(values_wo_outliers ~ names, Data8)
```

```
Data8b<- Data8|> select(block, names, values, values_wo_outliers)
Data8b
```

```
##            block names values values_wo_outliers
## 1 iPS09-45-M3_24    WT  13.23              13.23
## 2 iPS09-45-M3_24    WT  13.60              13.60
## 3 iPS09-45-M3_24    WT  14.70              14.70
## 4 iPS09-45-M3_24    WT  11.88              11.88
## 5 iPS09-45-M3_24    WT  17.41                 NA
```

### 1.2.6 NR5A1\_iPS09-M3\_48h00-Wt

```
Input = ("
names   values  block
WT  16.96   iPS09-45-M3_48
WT  17.28   iPS09-45-M3_48
WT  17.51   iPS09-45-M3_48
WT  18.63   iPS09-45-M3_48
WT  18.80   iPS09-45-M3_48
"
)
Data = read.table(textConnection(Input),header=TRUE)
Data$names = factor(Data$names,ordered=FALSE, levels=unique(Data$names))
Data$block = factor(Data$block,ordered=FALSE, levels=unique(Data$block))

# NR5A1_boxplot 1
boxplot(values ~ names,
        data = Data,
        ylab ="values",
        xlab ="names")
```

```
Data9 <- Data |>
  mutate(
    IQR = IQR(values, na.rm = TRUE),
    Outlier_upper = quantile(values, probs = c(.75), na.rm = TRUE) + 1.5 * IQR,
    Outlier_lower = quantile(values, probs = c(.25), na.rm = TRUE) - 1.5 * IQR,
    values_wo_outliers = if_else(values <= Outlier_lower | values >= Outlier_upper, NA, values))

boxplot(values_wo_outliers ~ names, Data9)
```

```
Data9b<- Data9|> select(block, names, values, values_wo_outliers)
Data9b
```

```
##            block names values values_wo_outliers
## 1 iPS09-45-M3_48    WT  16.96              16.96
## 2 iPS09-45-M3_48    WT  17.28              17.28
## 3 iPS09-45-M3_48    WT  17.51              17.51
## 4 iPS09-45-M3_48    WT  18.63              18.63
## 5 iPS09-45-M3_48    WT  18.80              18.80
```

### 1.2.7 NR5A1\_iPS09-M3\_48h00-Mut

```
Input = ("
names   values  block
Mut 17.46   iPS09-82-M3_48
Mut 17.63   iPS09-82-M3_48
Mut 17.38   iPS09-82-M3_48
Mut 17.82   iPS09-82-M3_48
Mut 16.53   iPS09-82-M3_48
"
)
Data = read.table(textConnection(Input),header=TRUE)
Data$names = factor(Data$names,ordered=FALSE, levels=unique(Data$names))
Data$block = factor(Data$block,ordered=FALSE, levels=unique(Data$block))

# NR5A1_boxplot 1
boxplot(values ~ names,
        data = Data,
        ylab ="values",
        xlab ="names")
```

```
Data10 <- Data |>
  mutate(
    IQR = IQR(values, na.rm = TRUE),
    Outlier_upper = quantile(values, probs = c(.75), na.rm = TRUE) + 1.5 * IQR,
    Outlier_lower = quantile(values, probs = c(.25), na.rm = TRUE) - 1.5 * IQR,
    values_wo_outliers = if_else(values <= Outlier_lower | values >= Outlier_upper, NA, values))

boxplot(values_wo_outliers ~ names, Data10)
```

```
Data10b<- Data10|> select(block, names, values, values_wo_outliers)
Data10b
```

```
##            block names values values_wo_outliers
## 1 iPS09-82-M3_48   Mut  17.46              17.46
## 2 iPS09-82-M3_48   Mut  17.63              17.63
## 3 iPS09-82-M3_48   Mut  17.38              17.38
## 4 iPS09-82-M3_48   Mut  17.82              17.82
## 5 iPS09-82-M3_48   Mut  16.53                 NA
```

## 1.3 NR5A1\_iPS12

### 1.3.1 NR5A1\_iPS12-M1\_36h00-Wt

```
Input = ("
names   values  block
WT  15.00   iPS12_45_M1_36_P
WT  13.69   iPS12_45_M1_36_P
WT  13.62   iPS12_45_M1_36_P
WT  13.99   iPS12_45_M1_36_P
WT  15.52   iPS12_45_M1_36_P
WT  13.49   iPS12_45_M1_36_P
"
)
Data = read.table(textConnection(Input),header=TRUE)
Data$names = factor(Data$names,ordered=FALSE, levels=unique(Data$names))
Data$block = factor(Data$block,ordered=FALSE, levels=unique(Data$block))

# NR5A1_boxplot 1
boxplot(values ~ names,
        data = Data,
        ylab ="values",
        xlab ="names")
```

```
Data13 <- Data |>
  mutate(
    IQR = IQR(values, na.rm = TRUE),
    Outlier_upper = quantile(values, probs = c(.75), na.rm = TRUE) + 1.5 * IQR,
    Outlier_lower = quantile(values, probs = c(.25), na.rm = TRUE) - 1.5 * IQR,
    values_wo_outliers = if_else(values <= Outlier_lower | values >= Outlier_upper, NA, values))

boxplot(values_wo_outliers ~ names, Data13)
```

```
Data13b<- Data13|> select(block, names, values, values_wo_outliers)
Data13b
```

```
##              block names values values_wo_outliers
## 1 iPS12_45_M1_36_P    WT  15.00              15.00
## 2 iPS12_45_M1_36_P    WT  13.69              13.69
## 3 iPS12_45_M1_36_P    WT  13.62              13.62
## 4 iPS12_45_M1_36_P    WT  13.99              13.99
## 5 iPS12_45_M1_36_P    WT  15.52              15.52
## 6 iPS12_45_M1_36_P    WT  13.49              13.49
```

### 1.3.2 NR5A1\_iPS12-M1\_36h00-Mut

```
Input = ("
names   values  block
Mut 12.78   iPS12_82_M1_36_P
Mut 13.32   iPS12_82_M1_36_P
Mut 14.84   iPS12_82_M1_36_P
Mut 13.66   iPS12_82_M1_36_P
Mut 12.66   iPS12_82_M1_36_P
Mut 12.46   iPS12_82_M1_36_P
"
)
Data = read.table(textConnection(Input),header=TRUE)
Data$names = factor(Data$names,ordered=FALSE, levels=unique(Data$names))
Data$block = factor(Data$block,ordered=FALSE, levels=unique(Data$block))

# NR5A1_boxplot 1
boxplot(values ~ names,
        data = Data,
        ylab ="values",
        xlab ="names")
```

```
Data14 <- Data |>
  mutate(
    IQR = IQR(values, na.rm = TRUE),
    Outlier_upper = quantile(values, probs = c(.75), na.rm = TRUE) + 1.5 * IQR,
    Outlier_lower = quantile(values, probs = c(.25), na.rm = TRUE) - 1.5 * IQR,
    values_wo_outliers = if_else(values <= Outlier_lower | values >= Outlier_upper, NA, values))

boxplot(values_wo_outliers ~ names, Data14)
```

```
Data14b<- Data14|> select(block, names, values, values_wo_outliers)
Data14b
```

```
##              block names values values_wo_outliers
## 1 iPS12_82_M1_36_P   Mut  12.78              12.78
## 2 iPS12_82_M1_36_P   Mut  13.32              13.32
## 3 iPS12_82_M1_36_P   Mut  14.84              14.84
## 4 iPS12_82_M1_36_P   Mut  13.66              13.66
## 5 iPS12_82_M1_36_P   Mut  12.66              12.66
## 6 iPS12_82_M1_36_P   Mut  12.46              12.46
```

### 1.3.3 NR5A1\_iPS12-M2\_06h00-Wt

```
Input = ("
names   values  block
WT  14.71   iPS12_45_M2_06_P
WT  15.28   iPS12_45_M2_06_P
WT  14.15   iPS12_45_M2_06_P
WT  15.69   iPS12_45_M2_06_P
WT  14.97   iPS12_45_M2_06_P
WT  NA  iPS12_45_M2_06_P
"
)
Data = read.table(textConnection(Input),header=TRUE)
Data$names = factor(Data$names,ordered=FALSE, levels=unique(Data$names))
Data$block = factor(Data$block,ordered=FALSE, levels=unique(Data$block))

# NR5A1_boxplot 1
boxplot(values ~ names,
        data = Data,
        ylab ="values",
        xlab ="names")
```

```
Data15 <- Data |>
  mutate(
    IQR = IQR(values, na.rm = TRUE),
    Outlier_upper = quantile(values, probs = c(.75), na.rm = TRUE) + 1.5 * IQR,
    Outlier_lower = quantile(values, probs = c(.25), na.rm = TRUE) - 1.5 * IQR,
    values_wo_outliers = if_else(values <= Outlier_lower | values >= Outlier_upper, NA, values))

boxplot(values_wo_outliers ~ names, Data15)
```

```
Data15b<- Data15|> select(block, names, values, values_wo_outliers)
Data15b
```

```
##              block names values values_wo_outliers
## 1 iPS12_45_M2_06_P    WT  14.71              14.71
## 2 iPS12_45_M2_06_P    WT  15.28              15.28
## 3 iPS12_45_M2_06_P    WT  14.15              14.15
## 4 iPS12_45_M2_06_P    WT  15.69              15.69
## 5 iPS12_45_M2_06_P    WT  14.97              14.97
## 6 iPS12_45_M2_06_P    WT     NA                 NA
```

### 1.3.4 NR5A1\_iPS12-M2\_06h00-Mut

```
Input = ("
names   values  block
Mut 13.64   iPS12_82_M2_06_P
Mut 14.29   iPS12_82_M2_06_P
Mut 15.26   iPS12_82_M2_06_P
Mut 15.86   iPS12_82_M2_06_P
Mut 17.16   iPS12_82_M2_06_P
Mut 15.18   iPS12_82_M2_06_P
"
)
Data = read.table(textConnection(Input),header=TRUE)
Data$names = factor(Data$names,ordered=FALSE, levels=unique(Data$names))
Data$block = factor(Data$block,ordered=FALSE, levels=unique(Data$block))

# NR5A1_boxplot 1
boxplot(values ~ names,
        data = Data,
        ylab ="values",
        xlab ="names")
```

```
Data16 <- Data |>
  mutate(
    IQR = IQR(values, na.rm = TRUE),
    Outlier_upper = quantile(values, probs = c(.75), na.rm = TRUE) + 1.5 * IQR,
    Outlier_lower = quantile(values, probs = c(.25), na.rm = TRUE) - 1.5 * IQR,
    values_wo_outliers = if_else(values <= Outlier_lower | values >= Outlier_upper, NA, values))

boxplot(values_wo_outliers ~ names, Data16)
```

```
Data16b<- Data16|> select(block, names, values, values_wo_outliers)
Data16b
```

```
##              block names values values_wo_outliers
## 1 iPS12_82_M2_06_P   Mut  13.64              13.64
## 2 iPS12_82_M2_06_P   Mut  14.29              14.29
## 3 iPS12_82_M2_06_P   Mut  15.26              15.26
## 4 iPS12_82_M2_06_P   Mut  15.86              15.86
## 5 iPS12_82_M2_06_P   Mut  17.16              17.16
## 6 iPS12_82_M2_06_P   Mut  15.18              15.18
```

### 1.3.5 NR5A1\_iPS12-M2\_12h00-Wt\_vs\_Mut

```
Input = ("
names   values  block
WT  15.62   iPS12_45_M2_12_P
WT  17.55   iPS12_45_M2_12_P
WT  16.06   iPS12_45_M2_12_P
WT  18.49   iPS12_45_M2_12_P
WT  16.63   iPS12_45_M2_12_P
WT  17.05   iPS12_45_M2_12_P
"
)
Data = read.table(textConnection(Input),header=TRUE)
Data$names = factor(Data$names,ordered=FALSE, levels=unique(Data$names))
Data$block = factor(Data$block,ordered=FALSE, levels=unique(Data$block))

# NR5A1_boxplot 1
boxplot(values ~ names,
        data = Data,
        ylab ="values",
        xlab ="names")
```

```
Data17 <- Data |>
  mutate(
    IQR = IQR(values, na.rm = TRUE),
    Outlier_upper = quantile(values, probs = c(.75), na.rm = TRUE) + 1.5 * IQR,
    Outlier_lower = quantile(values, probs = c(.25), na.rm = TRUE) - 1.5 * IQR,
    values_wo_outliers = if_else(values <= Outlier_lower | values >= Outlier_upper, NA, values))

boxplot(values_wo_outliers ~ names, Data17)
```

```
Data17b<- Data17|> select(block, names, values, values_wo_outliers)
Data17b
```

```
##              block names values values_wo_outliers
## 1 iPS12_45_M2_12_P    WT  15.62              15.62
## 2 iPS12_45_M2_12_P    WT  17.55              17.55
## 3 iPS12_45_M2_12_P    WT  16.06              16.06
## 4 iPS12_45_M2_12_P    WT  18.49              18.49
## 5 iPS12_45_M2_12_P    WT  16.63              16.63
## 6 iPS12_45_M2_12_P    WT  17.05              17.05
```

### 1.3.6 NR5A1\_iPS12-M2\_12h00-Mut

```
Input = ("
names   values  block
Mut 17.10   iPS12_82_M2_12_P
Mut 15.31   iPS12_82_M2_12_P
Mut 17.09   iPS12_82_M2_12_P
Mut 16.97   iPS12_82_M2_12_P
Mut 17.17   iPS12_82_M2_12_P
Mut 18.47   iPS12_82_M2_12_P
"
)
Data = read.table(textConnection(Input),header=TRUE)
Data$names = factor(Data$names,ordered=FALSE, levels=unique(Data$names))
Data$block = factor(Data$block,ordered=FALSE, levels=unique(Data$block))

# NR5A1_boxplot 1
boxplot(values ~ names,
        data = Data,
        ylab ="values",
        xlab ="names")
```

```
Data18 <- Data |>
  mutate(
    IQR = IQR(values, na.rm = TRUE),
    Outlier_upper = quantile(values, probs = c(.75), na.rm = TRUE) + 1.5 * IQR,
    Outlier_lower = quantile(values, probs = c(.25), na.rm = TRUE) - 1.5 * IQR,
    values_wo_outliers = if_else(values <= Outlier_lower | values >= Outlier_upper, NA, values))

boxplot(values_wo_outliers ~ names, Data18)
```

```
Data18b<- Data18|> select(block, names, values, values_wo_outliers)
Data18b
```

```
##              block names values values_wo_outliers
## 1 iPS12_82_M2_12_P   Mut  17.10              17.10
## 2 iPS12_82_M2_12_P   Mut  15.31                 NA
## 3 iPS12_82_M2_12_P   Mut  17.09              17.09
## 4 iPS12_82_M2_12_P   Mut  16.97              16.97
## 5 iPS12_82_M2_12_P   Mut  17.17              17.17
## 6 iPS12_82_M2_12_P   Mut  18.47                 NA
```

### 1.3.7 NR5A1\_iPS12\_M2\_24h00-Wt

```
Input = ("
names   values  block
WT  14.25   iPS12_45_M2_24_P
WT  16.23   iPS12_45_M2_24_P
WT  15.03   iPS12_45_M2_24_P
WT  15.54   iPS12_45_M2_24_P
WT  15.46   iPS12_45_M2_24_P
WT  NA  iPS12_45_M2_24_P
"
)
Data = read.table(textConnection(Input),header=TRUE)
Data$names = factor(Data$names,ordered=FALSE, levels=unique(Data$names))
Data$block = factor(Data$block,ordered=FALSE, levels=unique(Data$block))

# NR5A1_boxplot 1
boxplot(values ~ names,
        data = Data,
        ylab ="values",
        xlab ="names")
```

```
Data19 <- Data |>
  mutate(
    IQR = IQR(values, na.rm = TRUE),
    Outlier_upper = quantile(values, probs = c(.75), na.rm = TRUE) + 1.5 * IQR,
    Outlier_lower = quantile(values, probs = c(.25), na.rm = TRUE) - 1.5 * IQR,
    values_wo_outliers = if_else(values <= Outlier_lower | values >= Outlier_upper, NA, values))

boxplot(values_wo_outliers ~ names, Data19)
```

```
Data19b<- Data19|> select(block, names, values, values_wo_outliers)
Data19b
```

```
##              block names values values_wo_outliers
## 1 iPS12_45_M2_24_P    WT  14.25                 NA
## 2 iPS12_45_M2_24_P    WT  16.23              16.23
## 3 iPS12_45_M2_24_P    WT  15.03              15.03
## 4 iPS12_45_M2_24_P    WT  15.54              15.54
## 5 iPS12_45_M2_24_P    WT  15.46              15.46
## 6 iPS12_45_M2_24_P    WT     NA                 NA
```

### 1.3.8 NR5A1\_iPS12\_M2\_24h00-Mut

```
Input = ("
names   values  block
Mut 16.52   iPS12_82_M2_24_P
Mut 16.04   iPS12_82_M2_24_P
Mut 15.58   iPS12_82_M2_24_P
Mut 15.47   iPS12_82_M2_24_P
Mut 15.08   iPS12_82_M2_24_P
Mut 17.36   iPS12_82_M2_24_P
"
)
Data = read.table(textConnection(Input),header=TRUE)
Data$names = factor(Data$names,ordered=FALSE, levels=unique(Data$names))
Data$block = factor(Data$block,ordered=FALSE, levels=unique(Data$block))

# NR5A1_boxplot 1
boxplot(values ~ names,
        data = Data,
        ylab ="values",
        xlab ="names")
```

```
Data20 <- Data |>
  mutate(
    IQR = IQR(values, na.rm = TRUE),
    Outlier_upper = quantile(values, probs = c(.75), na.rm = TRUE) + 1.5 * IQR,
    Outlier_lower = quantile(values, probs = c(.25), na.rm = TRUE) - 1.5 * IQR,
    values_wo_outliers = if_else(values <= Outlier_lower | values >= Outlier_upper, NA, values))

boxplot(values_wo_outliers ~ names, Data20)
```

```
Data20b<- Data20|> select(block, names, values, values_wo_outliers)
Data20b
```

```
##              block names values values_wo_outliers
## 1 iPS12_82_M2_24_P   Mut  16.52              16.52
## 2 iPS12_82_M2_24_P   Mut  16.04              16.04
## 3 iPS12_82_M2_24_P   Mut  15.58              15.58
## 4 iPS12_82_M2_24_P   Mut  15.47              15.47
## 5 iPS12_82_M2_24_P   Mut  15.08              15.08
## 6 iPS12_82_M2_24_P   Mut  17.36              17.36
```

### 1.3.9 NR5A1\_iPS12-M2\_48h00-Wt

```
Input = ("
names   values  block
WT  13.57   iPS12_45_M2_48_P
WT  15.39   iPS12_45_M2_48_P
WT  14.97   iPS12_45_M2_48_P
WT  13.62   iPS12_45_M2_48_P
WT  14.13   iPS12_45_M2_48_P
WT  NA  iPS12_45_M2_48_P
"
)
Data = read.table(textConnection(Input),header=TRUE)
Data$names = factor(Data$names,ordered=FALSE, levels=unique(Data$names))
Data$block = factor(Data$block,ordered=FALSE, levels=unique(Data$block))

# NR5A1_boxplot 1
boxplot(values ~ names,
        data = Data,
        ylab ="values",
        xlab ="names")
```

```
Data21 <- Data |>
  mutate(
    IQR = IQR(values, na.rm = TRUE),
    Outlier_upper = quantile(values, probs = c(.75), na.rm = TRUE) + 1.5 * IQR,
    Outlier_lower = quantile(values, probs = c(.25), na.rm = TRUE) - 1.5 * IQR,
    values_wo_outliers = if_else(values <= Outlier_lower | values >= Outlier_upper, NA, values))

boxplot(values_wo_outliers ~ names, Data21)
```

```
Data21b<- Data21|> select(block, names, values, values_wo_outliers)
Data21b
```

```
##              block names values values_wo_outliers
## 1 iPS12_45_M2_48_P    WT  13.57              13.57
## 2 iPS12_45_M2_48_P    WT  15.39              15.39
## 3 iPS12_45_M2_48_P    WT  14.97              14.97
## 4 iPS12_45_M2_48_P    WT  13.62              13.62
## 5 iPS12_45_M2_48_P    WT  14.13              14.13
## 6 iPS12_45_M2_48_P    WT     NA                 NA
```

### 1.3.10 NR5A1\_iPS12-M2\_48h00-Mut

```
Input = ("
names   values  block
Mut 13.20   iPS12_82_M2_48_P
Mut 14.14   iPS12_82_M2_48_P
Mut 14.03   iPS12_82_M2_48_P
Mut 13.74   iPS12_82_M2_48_P
Mut 14.30   iPS12_82_M2_48_P
Mut NA  iPS12_82_M2_48_P
"
)
Data = read.table(textConnection(Input),header=TRUE)
Data$names = factor(Data$names,ordered=FALSE, levels=unique(Data$names))
Data$block = factor(Data$block,ordered=FALSE, levels=unique(Data$block))

# NR5A1_boxplot 1
boxplot(values ~ names,
        data = Data,
        ylab ="values",
        xlab ="names")
```

```
Data22 <- Data |>
  mutate(
    IQR = IQR(values, na.rm = TRUE),
    Outlier_upper = quantile(values, probs = c(.75), na.rm = TRUE) + 1.5 * IQR,
    Outlier_lower = quantile(values, probs = c(.25), na.rm = TRUE) - 1.5 * IQR,
    values_wo_outliers = if_else(values <= Outlier_lower | values >= Outlier_upper, NA, values))

boxplot(values_wo_outliers ~ names, Data22)
```

```
Data22b<- Data22|> select(block, names, values, values_wo_outliers)
Data22b
```

```
##              block names values values_wo_outliers
## 1 iPS12_82_M2_48_P   Mut  13.20              13.20
## 2 iPS12_82_M2_48_P   Mut  14.14              14.14
## 3 iPS12_82_M2_48_P   Mut  14.03              14.03
## 4 iPS12_82_M2_48_P   Mut  13.74              13.74
## 5 iPS12_82_M2_48_P   Mut  14.30              14.30
## 6 iPS12_82_M2_48_P   Mut     NA                 NA
```

## 1.4 NR5A1\_iPS19

### 1.4.1 NR5A1\_iPS19-iPS-Wt

```
Input = ("
names   values  block
WT  15.55   iPS19_45_iPS
WT  14.00   iPS19_45_iPS
WT  14.47   iPS19_45_iPS
WT  14.07   iPS19_45_iPS
WT  14.61   iPS19_45_iPS
WT  14.09   iPS19_45_iPS
WT  13.26   iPS19_82_iPS
WT  10.85   iPS19_82_iPS
WT  13.62   iPS19_82_iPS
WT  15.78   iPS19_82_iPS
WT  15.08   iPS19_82_iPS
WT  9.18    iPS19_82_iPS
"
)
Data = read.table(textConnection(Input),header=TRUE)
Data$names = factor(Data$names,ordered=FALSE, levels=unique(Data$names))
Data$block = factor(Data$block,ordered=FALSE, levels=unique(Data$block))

# NR5A1_boxplot 1
boxplot(values ~ names,
        data = Data,
        ylab ="values",
        xlab ="names")
```

```
Data23 <- Data |>
  mutate(
    IQR = IQR(values, na.rm = TRUE),
    Outlier_upper = quantile(values, probs = c(.75), na.rm = TRUE) + 1.5 * IQR,
    Outlier_lower = quantile(values, probs = c(.25), na.rm = TRUE) - 1.5 * IQR,
    values_wo_outliers = if_else(values <= Outlier_lower | values >= Outlier_upper, NA, values))

boxplot(values_wo_outliers ~ names, Data23)
```

```
Data23b<- Data23|> select(block, names, values, values_wo_outliers)
Data23b
```

```
##           block names values values_wo_outliers
## 1  iPS19_45_iPS    WT  15.55              15.55
## 2  iPS19_45_iPS    WT  14.00              14.00
## 3  iPS19_45_iPS    WT  14.47              14.47
## 4  iPS19_45_iPS    WT  14.07              14.07
## 5  iPS19_45_iPS    WT  14.61              14.61
## 6  iPS19_45_iPS    WT  14.09              14.09
## 7  iPS19_82_iPS    WT  13.26              13.26
## 8  iPS19_82_iPS    WT  10.85                 NA
## 9  iPS19_82_iPS    WT  13.62              13.62
## 10 iPS19_82_iPS    WT  15.78              15.78
## 11 iPS19_82_iPS    WT  15.08              15.08
## 12 iPS19_82_iPS    WT   9.18                 NA
```

### 1.4.2 NR5A1\_iPS19-iPS-Mut

```
Input = ("
names   values  block
Mut 14.33   iPS19_82_iPS
Mut 14.96   iPS19_82_iPS
Mut 14.95   iPS19_82_iPS
Mut 14.88   iPS19_82_iPS
Mut 15.38   iPS19_82_iPS
Mut 14.80   iPS19_82_iPS
Mut 13.74   iPS19_82_iPS
Mut 15.06   iPS19_82_iPS
Mut 14.14   iPS19_82_iPS
Mut 14.07   iPS19_82_iPS
Mut 13.91   iPS19_82_iPS
Mut 14.94   iPS19_82_iPS
"
)
Data = read.table(textConnection(Input),header=TRUE)
Data$names = factor(Data$names,ordered=FALSE, levels=unique(Data$names))
Data$block = factor(Data$block,ordered=FALSE, levels=unique(Data$block))

# NR5A1_boxplot 1
boxplot(values ~ names,
        data = Data,
        ylab ="values",
        xlab ="names")
```

```
Data24 <- Data |>
  mutate(
    IQR = IQR(values, na.rm = TRUE),
    Outlier_upper = quantile(values, probs = c(.75), na.rm = TRUE) + 1.5 * IQR,
    Outlier_lower = quantile(values, probs = c(.25), na.rm = TRUE) - 1.5 * IQR,
    values_wo_outliers = if_else(values <= Outlier_lower | values >= Outlier_upper, NA, values))

boxplot(values_wo_outliers ~ names, Data24)
```

```
Data24b<- Data24|> select(block, names, values, values_wo_outliers)
Data24b
```

```
##           block names values values_wo_outliers
## 1  iPS19_82_iPS   Mut  14.33              14.33
## 2  iPS19_82_iPS   Mut  14.96              14.96
## 3  iPS19_82_iPS   Mut  14.95              14.95
## 4  iPS19_82_iPS   Mut  14.88              14.88
## 5  iPS19_82_iPS   Mut  15.38              15.38
## 6  iPS19_82_iPS   Mut  14.80              14.80
## 7  iPS19_82_iPS   Mut  13.74              13.74
## 8  iPS19_82_iPS   Mut  15.06              15.06
## 9  iPS19_82_iPS   Mut  14.14              14.14
## 10 iPS19_82_iPS   Mut  14.07              14.07
## 11 iPS19_82_iPS   Mut  13.91              13.91
## 12 iPS19_82_iPS   Mut  14.94              14.94
```
