# Supplementary material for: A conserved NR5A1-responsive enhancer regulates SRY in testis-determination
Source: Nat Commun. 2024 Mar 30;15:2796. doi: 10.1038/s41467-024-47162-2 (PMC10981742; doi:10.1038/s41467-024-47162-2)
Supplement: Supplementary file 37 — Supplementary Dataset 34 [file 41467_2024_47162_MOESM37_ESM.html]

Supplementary\_Data\_34


# Supplementary\_Data\_34

#### Denis

#### 2023-07-10

RT-qPCR analysis - FOXL2 - outlier identification by the IQR method
in R

```
rm(list=ls())
```

```
library(tidyverse)
```

```
## ── Attaching core tidyverse packages ──────────────────────── tidyverse 2.0.0 ──
## ✔ dplyr     1.1.4     ✔ readr     2.1.5
## ✔ forcats   1.0.0     ✔ stringr   1.5.1
## ✔ ggplot2   3.4.4     ✔ tibble    3.2.1
## ✔ lubridate 1.9.3     ✔ tidyr     1.3.0
## ✔ purrr     1.0.2     
## ── Conflicts ────────────────────────────────────────── tidyverse_conflicts() ──
## ✖ dplyr::filter() masks stats::filter()
## ✖ dplyr::lag()    masks stats::lag()
## ℹ Use the conflicted package (<http://conflicted.r-lib.org/>) to force all conflicts to become errors
```

# 1 FOXL2

## 1.1 FOXL2\_iPS09

### 1.1.1 FOXL2\_iPS09-M1\_36h00-Wt

```
Input = ("
names   values  block
WT  13.34   iPS09-45-M1_36
WT  13.08   iPS09-45-M1_36
WT  11.58   iPS09-45-M1_36
WT  11.62   iPS09-45-M1_36
WT  11.87   iPS09-45-M1_36
"
)
Data = read.table(textConnection(Input),header=TRUE)
Data$names = factor(Data$names,ordered=FALSE, levels=unique(Data$names))
Data$block = factor(Data$block,ordered=FALSE, levels=unique(Data$block))

# FOXL2_boxplot 1
boxplot(values ~ names,
        data = Data,
        ylab ="values",
        xlab ="names")
```

```
Data4 <- Data |>
  mutate(
    IQR = IQR(values, na.rm = TRUE),
    Outlier_upper = quantile(values, probs = c(.75), na.rm = TRUE) + 1.5 * IQR,
    Outlier_lower = quantile(values, probs = c(.25), na.rm = TRUE) - 1.5 * IQR,
    values_wo_outliers = if_else(values <= Outlier_lower | values >= Outlier_upper, NA, values))

boxplot(values_wo_outliers ~ names, Data4)
```

```
Data4b<- Data4|> select(block, names, values, values_wo_outliers)
Data4b
```

```
##            block names values values_wo_outliers
## 1 iPS09-45-M1_36    WT  13.34              13.34
## 2 iPS09-45-M1_36    WT  13.08              13.08
## 3 iPS09-45-M1_36    WT  11.58              11.58
## 4 iPS09-45-M1_36    WT  11.62              11.62
## 5 iPS09-45-M1_36    WT  11.87              11.87
```

### 1.1.2 FOXL2\_iPS09-M1\_36h00-Mut

```
Input = ("
names   values  block
Mut 13.22   iPS09-82-M1_36
Mut 12.16   iPS09-82-M1_36
Mut 11.72   iPS09-82-M1_36
Mut 11.72   iPS09-82-M1_36
Mut 11.44   iPS09-82-M1_36
"
)
Data = read.table(textConnection(Input),header=TRUE)
Data$names = factor(Data$names,ordered=FALSE, levels=unique(Data$names))
Data$block = factor(Data$block,ordered=FALSE, levels=unique(Data$block))

# FOXL2_boxplot 1
boxplot(values ~ names,
        data = Data,
        ylab ="values",
        xlab ="names")
```

```
Data5 <- Data |>
  mutate(
    IQR = IQR(values, na.rm = TRUE),
    Outlier_upper = quantile(values, probs = c(.75), na.rm = TRUE) + 1.5 * IQR,
    Outlier_lower = quantile(values, probs = c(.25), na.rm = TRUE) - 1.5 * IQR,
    values_wo_outliers = if_else(values <= Outlier_lower | values >= Outlier_upper, NA, values))

boxplot(values_wo_outliers ~ names, Data5)
```

```
Data5b<- Data5|> select(block, names, values, values_wo_outliers)
Data5b
```

```
##            block names values values_wo_outliers
## 1 iPS09-82-M1_36   Mut  13.22                 NA
## 2 iPS09-82-M1_36   Mut  12.16              12.16
## 3 iPS09-82-M1_36   Mut  11.72              11.72
## 4 iPS09-82-M1_36   Mut  11.72              11.72
## 5 iPS09-82-M1_36   Mut  11.44              11.44
```

### 1.1.3 FOXL2\_iPS09-M2\_24h00-Wt

```
Input = ("
names   values  block
WT  11.49   iPS09-45-M2_24
WT  11.36   iPS09-45-M2_24
WT  10.99   iPS09-45-M2_24
WT  10.85   iPS09-45-M2_24
WT  11.10   iPS09-45-M2_24
"
)
Data = read.table(textConnection(Input),header=TRUE)
Data$names = factor(Data$names,ordered=FALSE, levels=unique(Data$names))
Data$block = factor(Data$block,ordered=FALSE, levels=unique(Data$block))

# FOXL2_boxplot 1
boxplot(values ~ names,
        data = Data,
        ylab ="values",
        xlab ="names")
```

```
Data6 <- Data |>
  mutate(
    IQR = IQR(values, na.rm = TRUE),
    Outlier_upper = quantile(values, probs = c(.75), na.rm = TRUE) + 1.5 * IQR,
    Outlier_lower = quantile(values, probs = c(.25), na.rm = TRUE) - 1.5 * IQR,
    values_wo_outliers = if_else(values <= Outlier_lower | values >= Outlier_upper, NA, values))

boxplot(values_wo_outliers ~ names, Data6)
```

```
Data6b<- Data6|> select(block, names, values, values_wo_outliers)
Data6b
```

```
##            block names values values_wo_outliers
## 1 iPS09-45-M2_24    WT  11.49              11.49
## 2 iPS09-45-M2_24    WT  11.36              11.36
## 3 iPS09-45-M2_24    WT  10.99              10.99
## 4 iPS09-45-M2_24    WT  10.85              10.85
## 5 iPS09-45-M2_24    WT  11.10              11.10
```

### 1.1.4 FOXL2\_iPS09-M2\_24h00-Mut

```
Input = ("
names   values  block
Mut 10.93   iPS09-82-M2_24
Mut 11.26   iPS09-82-M2_24
Mut 11.20   iPS09-82-M2_24
Mut 11.54   iPS09-82-M2_24
Mut 10.93   iPS09-82-M2_24
"
)
Data = read.table(textConnection(Input),header=TRUE)
Data$names = factor(Data$names,ordered=FALSE, levels=unique(Data$names))
Data$block = factor(Data$block,ordered=FALSE, levels=unique(Data$block))

# FOXL2_boxplot 1
boxplot(values ~ names,
        data = Data,
        ylab ="values",
        xlab ="names")
```

```
Data7 <- Data |>
  mutate(
    IQR = IQR(values, na.rm = TRUE),
    Outlier_upper = quantile(values, probs = c(.75), na.rm = TRUE) + 1.5 * IQR,
    Outlier_lower = quantile(values, probs = c(.25), na.rm = TRUE) - 1.5 * IQR,
    values_wo_outliers = if_else(values <= Outlier_lower | values >= Outlier_upper, NA, values))

boxplot(values_wo_outliers ~ names, Data7)
```

```
Data7b<- Data7|> select(block, names, values, values_wo_outliers)
Data7b
```

```
##            block names values values_wo_outliers
## 1 iPS09-82-M2_24   Mut  10.93              10.93
## 2 iPS09-82-M2_24   Mut  11.26              11.26
## 3 iPS09-82-M2_24   Mut  11.20              11.20
## 4 iPS09-82-M2_24   Mut  11.54              11.54
## 5 iPS09-82-M2_24   Mut  10.93              10.93
```

### 1.1.5 FOXL2\_iPS09-M3\_24h00-Wt

```
Input = ("
names   values  block
WT  10.75   iPS09-45-M3_24
WT  10.25   iPS09-45-M3_24
WT  10.49   iPS09-45-M3_24
WT  9.56    iPS09-45-M3_24
WT  15.97   iPS09-45-M3_24
"
)
Data = read.table(textConnection(Input),header=TRUE)
Data$names = factor(Data$names,ordered=FALSE, levels=unique(Data$names))
Data$block = factor(Data$block,ordered=FALSE, levels=unique(Data$block))

# FOXL2_boxplot 1
boxplot(values ~ names,
        data = Data,
        ylab ="values",
        xlab ="names")
```

```
Data8 <- Data |>
  mutate(
    IQR = IQR(values, na.rm = TRUE),
    Outlier_upper = quantile(values, probs = c(.75), na.rm = TRUE) + 1.5 * IQR,
    Outlier_lower = quantile(values, probs = c(.25), na.rm = TRUE) - 1.5 * IQR,
    values_wo_outliers = if_else(values <= Outlier_lower | values >= Outlier_upper, NA, values))

boxplot(values_wo_outliers ~ names, Data8)
```

```
Data8b<- Data8|> select(block, names, values, values_wo_outliers)
Data8b
```

```
##            block names values values_wo_outliers
## 1 iPS09-45-M3_24    WT  10.75              10.75
## 2 iPS09-45-M3_24    WT  10.25              10.25
## 3 iPS09-45-M3_24    WT  10.49              10.49
## 4 iPS09-45-M3_24    WT   9.56               9.56
## 5 iPS09-45-M3_24    WT  15.97                 NA
```

### 1.1.6 FOXL2\_iPS09-M3\_48h00-Wt

```
Input = ("
names   values  block
WT  15.38   iPS09-45-M3_48
WT  16.03   iPS09-45-M3_48
WT  14.90   iPS09-45-M3_48
WT  15.69   iPS09-45-M3_48
WT  15.25   iPS09-45-M3_48
"
)
Data = read.table(textConnection(Input),header=TRUE)
Data$names = factor(Data$names,ordered=FALSE, levels=unique(Data$names))
Data$block = factor(Data$block,ordered=FALSE, levels=unique(Data$block))

# FOXL2_boxplot 1
boxplot(values ~ names,
        data = Data,
        ylab ="values",
        xlab ="names")
```

```
Data9 <- Data |>
  mutate(
    IQR = IQR(values, na.rm = TRUE),
    Outlier_upper = quantile(values, probs = c(.75), na.rm = TRUE) + 1.5 * IQR,
    Outlier_lower = quantile(values, probs = c(.25), na.rm = TRUE) - 1.5 * IQR,
    values_wo_outliers = if_else(values <= Outlier_lower | values >= Outlier_upper, NA, values))

boxplot(values_wo_outliers ~ names, Data9)
```

```
Data9b<- Data9|> select(block, names, values, values_wo_outliers)
Data9b
```

```
##            block names values values_wo_outliers
## 1 iPS09-45-M3_48    WT  15.38              15.38
## 2 iPS09-45-M3_48    WT  16.03              16.03
## 3 iPS09-45-M3_48    WT  14.90              14.90
## 4 iPS09-45-M3_48    WT  15.69              15.69
## 5 iPS09-45-M3_48    WT  15.25              15.25
```

### 1.1.7 FOXL2\_iPS09-M3\_48h00-Mut

```
Input = ("
names   values  block
Mut 15.77   iPS09-82-M3_48
Mut 15.03   iPS09-82-M3_48
Mut 15.50   iPS09-82-M3_48
Mut 14.59   iPS09-82-M3_48
Mut 14.00   iPS09-82-M3_48
"
)
Data = read.table(textConnection(Input),header=TRUE)
Data$names = factor(Data$names,ordered=FALSE, levels=unique(Data$names))
Data$block = factor(Data$block,ordered=FALSE, levels=unique(Data$block))

# FOXL2_boxplot 1
boxplot(values ~ names,
        data = Data,
        ylab ="values",
        xlab ="names")
```

```
Data10 <- Data |>
  mutate(
    IQR = IQR(values, na.rm = TRUE),
    Outlier_upper = quantile(values, probs = c(.75), na.rm = TRUE) + 1.5 * IQR,
    Outlier_lower = quantile(values, probs = c(.25), na.rm = TRUE) - 1.5 * IQR,
    values_wo_outliers = if_else(values <= Outlier_lower | values >= Outlier_upper, NA, values))

boxplot(values_wo_outliers ~ names, Data10)
```

```
Data10b<- Data10|> select(block, names, values, values_wo_outliers)
Data10b
```

```
##            block names values values_wo_outliers
## 1 iPS09-82-M3_48   Mut  15.77              15.77
## 2 iPS09-82-M3_48   Mut  15.03              15.03
## 3 iPS09-82-M3_48   Mut  15.50              15.50
## 4 iPS09-82-M3_48   Mut  14.59              14.59
## 5 iPS09-82-M3_48   Mut  14.00              14.00
```

## 1.2 FOXL2\_iPS12

### 1.2.1 FOXL2\_iPS12-M1\_36h00-Wt

```
Input = ("
names   values  block
WT  11.28   iPS12_45_M1_36_P
WT  11.37   iPS12_45_M1_36_P
WT  11.25   iPS12_45_M1_36_P
WT  11.61   iPS12_45_M1_36_P
WT  11.63   iPS12_45_M1_36_P
WT  11.16   iPS12_45_M1_36_P
"
)
Data = read.table(textConnection(Input),header=TRUE)
Data$names = factor(Data$names,ordered=FALSE, levels=unique(Data$names))
Data$block = factor(Data$block,ordered=FALSE, levels=unique(Data$block))

# FOXL2_boxplot 1
boxplot(values ~ names,
        data = Data,
        ylab ="values",
        xlab ="names")
```

```
Data13 <- Data |>
  mutate(
    IQR = IQR(values, na.rm = TRUE),
    Outlier_upper = quantile(values, probs = c(.75), na.rm = TRUE) + 1.5 * IQR,
    Outlier_lower = quantile(values, probs = c(.25), na.rm = TRUE) - 1.5 * IQR,
    values_wo_outliers = if_else(values <= Outlier_lower | values >= Outlier_upper, NA, values))

boxplot(values_wo_outliers ~ names, Data13)
```

```
Data13b<- Data13|> select(block, names, values, values_wo_outliers)
Data13b
```

```
##              block names values values_wo_outliers
## 1 iPS12_45_M1_36_P    WT  11.28              11.28
## 2 iPS12_45_M1_36_P    WT  11.37              11.37
## 3 iPS12_45_M1_36_P    WT  11.25              11.25
## 4 iPS12_45_M1_36_P    WT  11.61              11.61
## 5 iPS12_45_M1_36_P    WT  11.63              11.63
## 6 iPS12_45_M1_36_P    WT  11.16              11.16
```

### 1.2.2 FOXL2\_iPS12-M1\_36h00-Mut

```
Input = ("
names   values  block
Mut 11.36   iPS12_82_M1_36_P
Mut 11.43   iPS12_82_M1_36_P
Mut 11.61   iPS12_82_M1_36_P
Mut 11.66   iPS12_82_M1_36_P
Mut 11.17   iPS12_82_M1_36_P
Mut 11.29   iPS12_82_M1_36_P
"
)
Data = read.table(textConnection(Input),header=TRUE)
Data$names = factor(Data$names,ordered=FALSE, levels=unique(Data$names))
Data$block = factor(Data$block,ordered=FALSE, levels=unique(Data$block))

# FOXL2_boxplot 1
boxplot(values ~ names,
        data = Data,
        ylab ="values",
        xlab ="names")
```

```
Data14 <- Data |>
  mutate(
    IQR = IQR(values, na.rm = TRUE),
    Outlier_upper = quantile(values, probs = c(.75), na.rm = TRUE) + 1.5 * IQR,
    Outlier_lower = quantile(values, probs = c(.25), na.rm = TRUE) - 1.5 * IQR,
    values_wo_outliers = if_else(values <= Outlier_lower | values >= Outlier_upper, NA, values))

boxplot(values_wo_outliers ~ names, Data14)
```

```
Data14b<- Data14|> select(block, names, values, values_wo_outliers)
Data14b
```

```
##              block names values values_wo_outliers
## 1 iPS12_82_M1_36_P   Mut  11.36              11.36
## 2 iPS12_82_M1_36_P   Mut  11.43              11.43
## 3 iPS12_82_M1_36_P   Mut  11.61              11.61
## 4 iPS12_82_M1_36_P   Mut  11.66              11.66
## 5 iPS12_82_M1_36_P   Mut  11.17              11.17
## 6 iPS12_82_M1_36_P   Mut  11.29              11.29
```

### 1.2.3 FOXL2\_iPS12-M2\_06h00-Wt

```
Input = ("
names   values  block
WT  12.16   iPS12_45_M2_06_P
WT  12.59   iPS12_45_M2_06_P
WT  11.83   iPS12_45_M2_06_P
WT  12.59   iPS12_45_M2_06_P
WT  12.54   iPS12_45_M2_06_P
WT  NA  iPS12_45_M2_06_P
"
)
Data = read.table(textConnection(Input),header=TRUE)
Data$names = factor(Data$names,ordered=FALSE, levels=unique(Data$names))
Data$block = factor(Data$block,ordered=FALSE, levels=unique(Data$block))

# FOXL2_boxplot 1
boxplot(values ~ names,
        data = Data,
        ylab ="values",
        xlab ="names")
```

```
Data15 <- Data |>
  mutate(
    IQR = IQR(values, na.rm = TRUE),
    Outlier_upper = quantile(values, probs = c(.75), na.rm = TRUE) + 1.5 * IQR,
    Outlier_lower = quantile(values, probs = c(.25), na.rm = TRUE) - 1.5 * IQR,
    values_wo_outliers = if_else(values <= Outlier_lower | values >= Outlier_upper, NA, values))

boxplot(values_wo_outliers ~ names, Data15)
```

```
Data15b<- Data15|> select(block, names, values, values_wo_outliers)
Data15b
```

```
##              block names values values_wo_outliers
## 1 iPS12_45_M2_06_P    WT  12.16              12.16
## 2 iPS12_45_M2_06_P    WT  12.59              12.59
## 3 iPS12_45_M2_06_P    WT  11.83              11.83
## 4 iPS12_45_M2_06_P    WT  12.59              12.59
## 5 iPS12_45_M2_06_P    WT  12.54              12.54
## 6 iPS12_45_M2_06_P    WT     NA                 NA
```

### 1.2.4 FOXL2\_iPS12-M2\_06h00-Mut

```
Input = ("
names   values  block
Mut 11.42   iPS12_82_M2_06_P
Mut 12.44   iPS12_82_M2_06_P
Mut 12.95   iPS12_82_M2_06_P
Mut 12.52   iPS12_82_M2_06_P
Mut 12.51   iPS12_82_M2_06_P
Mut 12.51   iPS12_82_M2_06_P
"
)
Data = read.table(textConnection(Input),header=TRUE)
Data$names = factor(Data$names,ordered=FALSE, levels=unique(Data$names))
Data$block = factor(Data$block,ordered=FALSE, levels=unique(Data$block))

# FOXL2_boxplot 1
boxplot(values ~ names,
        data = Data,
        ylab ="values",
        xlab ="names")
```

```
Data16 <- Data |>
  mutate(
    IQR = IQR(values, na.rm = TRUE),
    Outlier_upper = quantile(values, probs = c(.75), na.rm = TRUE) + 1.5 * IQR,
    Outlier_lower = quantile(values, probs = c(.25), na.rm = TRUE) - 1.5 * IQR,
    values_wo_outliers = if_else(values <= Outlier_lower | values >= Outlier_upper, NA, values))

boxplot(values_wo_outliers ~ names, Data16)
```

```
Data16b<- Data16|> select(block, names, values, values_wo_outliers)
Data16b
```

```
##              block names values values_wo_outliers
## 1 iPS12_82_M2_06_P   Mut  11.42                 NA
## 2 iPS12_82_M2_06_P   Mut  12.44              12.44
## 3 iPS12_82_M2_06_P   Mut  12.95                 NA
## 4 iPS12_82_M2_06_P   Mut  12.52              12.52
## 5 iPS12_82_M2_06_P   Mut  12.51              12.51
## 6 iPS12_82_M2_06_P   Mut  12.51              12.51
```

### 1.2.5 FOXL2\_iPS12-M2\_12h00-Wt

```
Input = ("
names   values  block
WT  12.29   iPS12_45_M2_12_P
WT  12.76   iPS12_45_M2_12_P
WT  12.96   iPS12_45_M2_12_P
WT  13.28   iPS12_45_M2_12_P
WT  13.39   iPS12_45_M2_12_P
WT  12.97   iPS12_45_M2_12_P
"
)
Data = read.table(textConnection(Input),header=TRUE)
Data$names = factor(Data$names,ordered=FALSE, levels=unique(Data$names))
Data$block = factor(Data$block,ordered=FALSE, levels=unique(Data$block))

# FOXL2_boxplot 1
boxplot(values ~ names,
        data = Data,
        ylab ="values",
        xlab ="names")
```

```
Data17 <- Data |>
  mutate(
    IQR = IQR(values, na.rm = TRUE),
    Outlier_upper = quantile(values, probs = c(.75), na.rm = TRUE) + 1.5 * IQR,
    Outlier_lower = quantile(values, probs = c(.25), na.rm = TRUE) - 1.5 * IQR,
    values_wo_outliers = if_else(values <= Outlier_lower | values >= Outlier_upper, NA, values))

boxplot(values_wo_outliers ~ names, Data17)
```

```
Data17b<- Data17|> select(block, names, values, values_wo_outliers)
Data17b
```

```
##              block names values values_wo_outliers
## 1 iPS12_45_M2_12_P    WT  12.29              12.29
## 2 iPS12_45_M2_12_P    WT  12.76              12.76
## 3 iPS12_45_M2_12_P    WT  12.96              12.96
## 4 iPS12_45_M2_12_P    WT  13.28              13.28
## 5 iPS12_45_M2_12_P    WT  13.39              13.39
## 6 iPS12_45_M2_12_P    WT  12.97              12.97
```

### 1.2.6 FOXL2\_iPS12-M2\_12h00-Mut

```
Input = ("
names   values  block
Mut 12.86   iPS12_82_M2_12_P
Mut 13.17   iPS12_82_M2_12_P
Mut 13.33   iPS12_82_M2_12_P
Mut 13.14   iPS12_82_M2_12_P
Mut 13.63   iPS12_82_M2_12_P
Mut 13.37   iPS12_82_M2_12_P
"
)
Data = read.table(textConnection(Input),header=TRUE)
Data$names = factor(Data$names,ordered=FALSE, levels=unique(Data$names))
Data$block = factor(Data$block,ordered=FALSE, levels=unique(Data$block))

# FOXL2_boxplot 1
boxplot(values ~ names,
        data = Data,
        ylab ="values",
        xlab ="names")
```

```
Data18 <- Data |>
  mutate(
    IQR = IQR(values, na.rm = TRUE),
    Outlier_upper = quantile(values, probs = c(.75), na.rm = TRUE) + 1.5 * IQR,
    Outlier_lower = quantile(values, probs = c(.25), na.rm = TRUE) - 1.5 * IQR,
    values_wo_outliers = if_else(values <= Outlier_lower | values >= Outlier_upper, NA, values))

boxplot(values_wo_outliers ~ names, Data18)
```

```
Data18b<- Data18|> select(block, names, values, values_wo_outliers)
Data18b
```

```
##              block names values values_wo_outliers
## 1 iPS12_82_M2_12_P   Mut  12.86              12.86
## 2 iPS12_82_M2_12_P   Mut  13.17              13.17
## 3 iPS12_82_M2_12_P   Mut  13.33              13.33
## 4 iPS12_82_M2_12_P   Mut  13.14              13.14
## 5 iPS12_82_M2_12_P   Mut  13.63              13.63
## 6 iPS12_82_M2_12_P   Mut  13.37              13.37
```

### 1.2.7 FOXL2\_iPS12\_M2\_24h00-Wt

```
Input = ("
names   values  block
WT  NA  iPS12_45_M2_24_P
WT  10.73   iPS12_45_M2_24_P
WT  10.30   iPS12_45_M2_24_P
WT  10.41   iPS12_45_M2_24_P
WT  10.78   iPS12_45_M2_24_P
WT  10.93   iPS12_45_M2_24_P
"
)
Data = read.table(textConnection(Input),header=TRUE)
Data$names = factor(Data$names,ordered=FALSE, levels=unique(Data$names))
Data$block = factor(Data$block,ordered=FALSE, levels=unique(Data$block))

# FOXL2_boxplot 1
boxplot(values ~ names,
        data = Data,
        ylab ="values",
        xlab ="names")
```

```
Data19 <- Data |>
  mutate(
    IQR = IQR(values, na.rm = TRUE),
    Outlier_upper = quantile(values, probs = c(.75), na.rm = TRUE) + 1.5 * IQR,
    Outlier_lower = quantile(values, probs = c(.25), na.rm = TRUE) - 1.5 * IQR,
    values_wo_outliers = if_else(values <= Outlier_lower | values >= Outlier_upper, NA, values))

boxplot(values_wo_outliers ~ names, Data19)
```

```
Data19b<- Data19|> select(block, names, values, values_wo_outliers)
Data19b
```

```
##              block names values values_wo_outliers
## 1 iPS12_45_M2_24_P    WT     NA                 NA
## 2 iPS12_45_M2_24_P    WT  10.73              10.73
## 3 iPS12_45_M2_24_P    WT  10.30              10.30
## 4 iPS12_45_M2_24_P    WT  10.41              10.41
## 5 iPS12_45_M2_24_P    WT  10.78              10.78
## 6 iPS12_45_M2_24_P    WT  10.93              10.93
```

### 1.2.8 FOXL2\_iPS12\_M2\_24h00-Mut

```
Input = ("
names   values  block
Mut 11.48   iPS12_82_M2_24_P
Mut 11.13   iPS12_82_M2_24_P
Mut 11.41   iPS12_82_M2_24_P
Mut 11.44   iPS12_82_M2_24_P
Mut 11.12   iPS12_82_M2_24_P
Mut 11.76   iPS12_82_M2_24_P
"
)
Data = read.table(textConnection(Input),header=TRUE)
Data$names = factor(Data$names,ordered=FALSE, levels=unique(Data$names))
Data$block = factor(Data$block,ordered=FALSE, levels=unique(Data$block))

# FOXL2_boxplot 1
boxplot(values ~ names,
        data = Data,
        ylab ="values",
        xlab ="names")
```

```
Data20 <- Data |>
  mutate(
    IQR = IQR(values, na.rm = TRUE),
    Outlier_upper = quantile(values, probs = c(.75), na.rm = TRUE) + 1.5 * IQR,
    Outlier_lower = quantile(values, probs = c(.25), na.rm = TRUE) - 1.5 * IQR,
    values_wo_outliers = if_else(values <= Outlier_lower | values >= Outlier_upper, NA, values))

boxplot(values_wo_outliers ~ names, Data20)
```

```
Data20b<- Data20|> select(block, names, values, values_wo_outliers)
Data20b
```

```
##              block names values values_wo_outliers
## 1 iPS12_82_M2_24_P   Mut  11.48              11.48
## 2 iPS12_82_M2_24_P   Mut  11.13              11.13
## 3 iPS12_82_M2_24_P   Mut  11.41              11.41
## 4 iPS12_82_M2_24_P   Mut  11.44              11.44
## 5 iPS12_82_M2_24_P   Mut  11.12              11.12
## 6 iPS12_82_M2_24_P   Mut  11.76              11.76
```

### 1.2.9 FOXL2\_iPS12-M2\_48h00-Wt

```
Input = ("
names   values  block
WT  10.99   iPS12_45_M2_48_P
WT  11.30   iPS12_45_M2_48_P
WT  11.48   iPS12_45_M2_48_P
WT  10.40   iPS12_45_M2_48_P
WT  11.38   iPS12_45_M2_48_P
WT  NA  iPS12_45_M2_48_P
"
)
Data = read.table(textConnection(Input),header=TRUE)
Data$names = factor(Data$names,ordered=FALSE, levels=unique(Data$names))
Data$block = factor(Data$block,ordered=FALSE, levels=unique(Data$block))

# FOXL2_boxplot 1
boxplot(values ~ names,
        data = Data,
        ylab ="values",
        xlab ="names")
```

```
Data21 <- Data |>
  mutate(
    IQR = IQR(values, na.rm = TRUE),
    Outlier_upper = quantile(values, probs = c(.75), na.rm = TRUE) + 1.5 * IQR,
    Outlier_lower = quantile(values, probs = c(.25), na.rm = TRUE) - 1.5 * IQR,
    values_wo_outliers = if_else(values <= Outlier_lower | values >= Outlier_upper, NA, values))

boxplot(values_wo_outliers ~ names, Data21)
```

```
Data21b<- Data21|> select(block, names, values, values_wo_outliers)
Data21b
```

```
##              block names values values_wo_outliers
## 1 iPS12_45_M2_48_P    WT  10.99              10.99
## 2 iPS12_45_M2_48_P    WT  11.30              11.30
## 3 iPS12_45_M2_48_P    WT  11.48              11.48
## 4 iPS12_45_M2_48_P    WT  10.40                 NA
## 5 iPS12_45_M2_48_P    WT  11.38              11.38
## 6 iPS12_45_M2_48_P    WT     NA                 NA
```

### 1.2.10 FOXL2\_iPS12-M2\_48h00-Mut

```
Input = ("
names   values  block
Mut 10.83   iPS12_82_M2_48_P
Mut 11.35   iPS12_82_M2_48_P
Mut 11.08   iPS12_82_M2_48_P
Mut 11.36   iPS12_82_M2_48_P
Mut 11.56   iPS12_82_M2_48_P
Mut NA  iPS12_82_M2_48_P
"
)
Data = read.table(textConnection(Input),header=TRUE)
Data$names = factor(Data$names,ordered=FALSE, levels=unique(Data$names))
Data$block = factor(Data$block,ordered=FALSE, levels=unique(Data$block))

# FOXL2_boxplot 1
boxplot(values ~ names,
        data = Data,
        ylab ="values",
        xlab ="names")
```

```
Data22 <- Data |>
  mutate(
    IQR = IQR(values, na.rm = TRUE),
    Outlier_upper = quantile(values, probs = c(.75), na.rm = TRUE) + 1.5 * IQR,
    Outlier_lower = quantile(values, probs = c(.25), na.rm = TRUE) - 1.5 * IQR,
    values_wo_outliers = if_else(values <= Outlier_lower | values >= Outlier_upper, NA, values))

boxplot(values_wo_outliers ~ names, Data22)
```

```
Data22b<- Data22|> select(block, names, values, values_wo_outliers)
Data22b
```

```
##              block names values values_wo_outliers
## 1 iPS12_82_M2_48_P   Mut  10.83              10.83
## 2 iPS12_82_M2_48_P   Mut  11.35              11.35
## 3 iPS12_82_M2_48_P   Mut  11.08              11.08
## 4 iPS12_82_M2_48_P   Mut  11.36              11.36
## 5 iPS12_82_M2_48_P   Mut  11.56              11.56
## 6 iPS12_82_M2_48_P   Mut     NA                 NA
```

## 1.3 FOXL2\_iPS19

### 1.3.1 FOXL2\_iPS19-iPS-Wt

```
Input = ("
names   values  block
WT  11.48   iPS19_45_iPS
WT  12.23   iPS19_45_iPS
WT  11.42   iPS19_45_iPS
WT  11.26   iPS19_45_iPS
WT  11.68   iPS19_45_iPS
WT  11.72   iPS19_45_iPS
WT  11.02   iPS19_82_iPS
WT  11.74   iPS19_82_iPS
WT  11.84   iPS19_82_iPS
WT  11.70   iPS19_82_iPS
WT  11.10   iPS19_82_iPS
WT  10.34   iPS19_82_iPS
"
)
Data = read.table(textConnection(Input),header=TRUE)
Data$names = factor(Data$names,ordered=FALSE, levels=unique(Data$names))
Data$block = factor(Data$block,ordered=FALSE, levels=unique(Data$block))

# SRY_boxplot 1
boxplot(values ~ names,
        data = Data,
        ylab ="values",
        xlab ="names")
```

```
Data23 <- Data |>
  mutate(
    IQR = IQR(values, na.rm = TRUE),
    Outlier_upper = quantile(values, probs = c(.75), na.rm = TRUE) + 1.5 * IQR,
    Outlier_lower = quantile(values, probs = c(.25), na.rm = TRUE) - 1.5 * IQR,
    values_wo_outliers = if_else(values <= Outlier_lower | values >= Outlier_upper, NA, values))

boxplot(values_wo_outliers ~ names, Data23)
```

```
Data23b<- Data23|> select(block, names, values, values_wo_outliers)
Data23b
```

```
##           block names values values_wo_outliers
## 1  iPS19_45_iPS    WT  11.48              11.48
## 2  iPS19_45_iPS    WT  12.23              12.23
## 3  iPS19_45_iPS    WT  11.42              11.42
## 4  iPS19_45_iPS    WT  11.26              11.26
## 5  iPS19_45_iPS    WT  11.68              11.68
## 6  iPS19_45_iPS    WT  11.72              11.72
## 7  iPS19_82_iPS    WT  11.02              11.02
## 8  iPS19_82_iPS    WT  11.74              11.74
## 9  iPS19_82_iPS    WT  11.84              11.84
## 10 iPS19_82_iPS    WT  11.70              11.70
## 11 iPS19_82_iPS    WT  11.10              11.10
## 12 iPS19_82_iPS    WT  10.34                 NA
```

### 1.3.2 FOXL2\_iPS19-iPS-Mut

```
Input = ("
names   values  block
Mut 11.01   iPS19_82_iPS
Mut 10.97   iPS19_82_iPS
Mut 11.40   iPS19_82_iPS
Mut 11.52   iPS19_82_iPS
Mut 11.25   iPS19_82_iPS
Mut 10.66   iPS19_82_iPS
Mut 11.20   iPS19_82_iPS
Mut 11.44   iPS19_82_iPS
Mut 10.40   iPS19_82_iPS
Mut 10.28   iPS19_82_iPS
Mut 11.11   iPS19_82_iPS
Mut 11.28   iPS19_82_iPS
"
)
Data = read.table(textConnection(Input),header=TRUE)
Data$names = factor(Data$names,ordered=FALSE, levels=unique(Data$names))
Data$block = factor(Data$block,ordered=FALSE, levels=unique(Data$block))

# SRY_boxplot 1
boxplot(values ~ names,
        data = Data,
        ylab ="values",
        xlab ="names")
```

```
Data24 <- Data |>
  mutate(
    IQR = IQR(values, na.rm = TRUE),
    Outlier_upper = quantile(values, probs = c(.75), na.rm = TRUE) + 1.5 * IQR,
    Outlier_lower = quantile(values, probs = c(.25), na.rm = TRUE) - 1.5 * IQR,
    values_wo_outliers = if_else(values <= Outlier_lower | values >= Outlier_upper, NA, values))

boxplot(values_wo_outliers ~ names, Data24)
```

```
Data24b<- Data24|> select(block, names, values, values_wo_outliers)
Data24b
```

```
##           block names values values_wo_outliers
## 1  iPS19_82_iPS   Mut  11.01              11.01
## 2  iPS19_82_iPS   Mut  10.97              10.97
## 3  iPS19_82_iPS   Mut  11.40              11.40
## 4  iPS19_82_iPS   Mut  11.52              11.52
## 5  iPS19_82_iPS   Mut  11.25              11.25
## 6  iPS19_82_iPS   Mut  10.66              10.66
## 7  iPS19_82_iPS   Mut  11.20              11.20
## 8  iPS19_82_iPS   Mut  11.44              11.44
## 9  iPS19_82_iPS   Mut  10.40              10.40
## 10 iPS19_82_iPS   Mut  10.28              10.28
## 11 iPS19_82_iPS   Mut  11.11              11.11
## 12 iPS19_82_iPS   Mut  11.28              11.28
```
