# Supplementary material for: A conserved NR5A1-responsive enhancer regulates SRY in testis-determination
Source: Nat Commun. 2024 Mar 30;15:2796. doi: 10.1038/s41467-024-47162-2 (PMC10981742; doi:10.1038/s41467-024-47162-2)
Supplement: Supplementary file 38 — Supplementary Dataset 35 [file 41467_2024_47162_MOESM38_ESM.html]

Supplementary\_Data\_35


# Supplementary\_Data\_35

#### Denis

#### 2023-07-10

RT-qPCR analysis - FGF9 - outlier identification by the IQR method in
R

```
rm(list=ls())
```

```
library(tidyverse)
```

```
## ── Attaching core tidyverse packages ──────────────────────── tidyverse 2.0.0 ──
## ✔ dplyr     1.1.4     ✔ readr     2.1.5
## ✔ forcats   1.0.0     ✔ stringr   1.5.1
## ✔ ggplot2   3.4.4     ✔ tibble    3.2.1
## ✔ lubridate 1.9.3     ✔ tidyr     1.3.0
## ✔ purrr     1.0.2     
## ── Conflicts ────────────────────────────────────────── tidyverse_conflicts() ──
## ✖ dplyr::filter() masks stats::filter()
## ✖ dplyr::lag()    masks stats::lag()
## ℹ Use the conflicted package (<http://conflicted.r-lib.org/>) to force all conflicts to become errors
```

# 1 FGF9

## 1.1 FGF9\_iPS09

### 1.1.1 FGF9\_iPS09-M1\_36h00-Wt

```
Input = ("
names   values  block
WT  12.46   iPS09-45-M1_36h00
WT  12.55   iPS09-45-M1_36h00
WT  11.82   iPS09-45-M1_36h00
WT  13.03   iPS09-45-M1_36h00
WT  11.69   iPS09-45-M1_36h00
"
)
Data = read.table(textConnection(Input),header=TRUE)
Data$names = factor(Data$names,ordered=FALSE, levels=unique(Data$names))
Data$block = factor(Data$block,ordered=FALSE, levels=unique(Data$block))

# FGF9_boxplot 1
boxplot(values ~ names,
        data = Data,
        ylab ="values",
        xlab ="names")
```

```
Data4 <- Data |>
  mutate(
    IQR = IQR(values, na.rm = TRUE),
    Outlier_upper = quantile(values, probs = c(.75), na.rm = TRUE) + 1.5 * IQR,
    Outlier_lower = quantile(values, probs = c(.25), na.rm = TRUE) - 1.5 * IQR,
    values_wo_outliers = if_else(values <= Outlier_lower | values >= Outlier_upper, NA, values))

boxplot(values_wo_outliers ~ names, Data4)
```

```
Data4b<- Data4|> select(block, names, values, values_wo_outliers)
Data4b
```

```
##               block names values values_wo_outliers
## 1 iPS09-45-M1_36h00    WT  12.46              12.46
## 2 iPS09-45-M1_36h00    WT  12.55              12.55
## 3 iPS09-45-M1_36h00    WT  11.82              11.82
## 4 iPS09-45-M1_36h00    WT  13.03              13.03
## 5 iPS09-45-M1_36h00    WT  11.69              11.69
```

### 1.1.2 FGF9\_iPS09-M1\_36h00-Mut

```
Input = ("
names   values  block
Mut 11.17   iPS09-82-M1_36h00
Mut 11.65   iPS09-82-M1_36h00
Mut 12.31   iPS09-82-M1_36h00
Mut 11.03   iPS09-82-M1_36h00
Mut 10.36   iPS09-82-M1_36h00
"
)
Data = read.table(textConnection(Input),header=TRUE)
Data$names = factor(Data$names,ordered=FALSE, levels=unique(Data$names))
Data$block = factor(Data$block,ordered=FALSE, levels=unique(Data$block))

# FGF9_boxplot 1
boxplot(values ~ names,
        data = Data,
        ylab ="values",
        xlab ="names")
```

```
Data5 <- Data |>
  mutate(
    IQR = IQR(values, na.rm = TRUE),
    Outlier_upper = quantile(values, probs = c(.75), na.rm = TRUE) + 1.5 * IQR,
    Outlier_lower = quantile(values, probs = c(.25), na.rm = TRUE) - 1.5 * IQR,
    values_wo_outliers = if_else(values <= Outlier_lower | values >= Outlier_upper, NA, values))

boxplot(values_wo_outliers ~ names, Data5)
```

```
Data5b<- Data5|> select(block, names, values, values_wo_outliers)
Data5b
```

```
##               block names values values_wo_outliers
## 1 iPS09-82-M1_36h00   Mut  11.17              11.17
## 2 iPS09-82-M1_36h00   Mut  11.65              11.65
## 3 iPS09-82-M1_36h00   Mut  12.31              12.31
## 4 iPS09-82-M1_36h00   Mut  11.03              11.03
## 5 iPS09-82-M1_36h00   Mut  10.36              10.36
```

### 1.1.3 FGF9\_iPS09-M2\_24h00-Wt

```
Input = ("
names   values  block
WT  12.99   iPS09-45-M2_24h00
WT  12.28   iPS09-45-M2_24h00
WT  13.07   iPS09-45-M2_24h00
WT  12.03   iPS09-45-M2_24h00
WT  12.28   iPS09-45-M2_24h00
"
)
Data = read.table(textConnection(Input),header=TRUE)
Data$names = factor(Data$names,ordered=FALSE, levels=unique(Data$names))
Data$block = factor(Data$block,ordered=FALSE, levels=unique(Data$block))

# FGF9_boxplot 1
boxplot(values ~ names,
        data = Data,
        ylab ="values",
        xlab ="names")
```

```
Data6 <- Data |>
  mutate(
    IQR = IQR(values, na.rm = TRUE),
    Outlier_upper = quantile(values, probs = c(.75), na.rm = TRUE) + 1.5 * IQR,
    Outlier_lower = quantile(values, probs = c(.25), na.rm = TRUE) - 1.5 * IQR,
    values_wo_outliers = if_else(values <= Outlier_lower | values >= Outlier_upper, NA, values))

boxplot(values_wo_outliers ~ names, Data6)
```

```
Data6b<- Data6|> select(block, names, values, values_wo_outliers)
Data6b
```

```
##               block names values values_wo_outliers
## 1 iPS09-45-M2_24h00    WT  12.99              12.99
## 2 iPS09-45-M2_24h00    WT  12.28              12.28
## 3 iPS09-45-M2_24h00    WT  13.07              13.07
## 4 iPS09-45-M2_24h00    WT  12.03              12.03
## 5 iPS09-45-M2_24h00    WT  12.28              12.28
```

### 1.1.4 FGF9\_iPS09-M2\_24h00-Mut

```
Input = ("
names   values  block
Mut 10.18   iPS09-82-M2_24h00
Mut 11.28   iPS09-82-M2_24h00
Mut 10.56   iPS09-82-M2_24h00
Mut 10.53   iPS09-82-M2_24h00
Mut 11.17   iPS09-82-M2_24h00
"
)
Data = read.table(textConnection(Input),header=TRUE)
Data$names = factor(Data$names,ordered=FALSE, levels=unique(Data$names))
Data$block = factor(Data$block,ordered=FALSE, levels=unique(Data$block))

# FGF9_boxplot 1
boxplot(values ~ names,
        data = Data,
        ylab ="values",
        xlab ="names")
```

```
Data7 <- Data |>
  mutate(
    IQR = IQR(values, na.rm = TRUE),
    Outlier_upper = quantile(values, probs = c(.75), na.rm = TRUE) + 1.5 * IQR,
    Outlier_lower = quantile(values, probs = c(.25), na.rm = TRUE) - 1.5 * IQR,
    values_wo_outliers = if_else(values <= Outlier_lower | values >= Outlier_upper, NA, values))

boxplot(values_wo_outliers ~ names, Data7)
```

```
Data7b<- Data7|> select(block, names, values, values_wo_outliers)
Data7b
```

```
##               block names values values_wo_outliers
## 1 iPS09-82-M2_24h00   Mut  10.18              10.18
## 2 iPS09-82-M2_24h00   Mut  11.28              11.28
## 3 iPS09-82-M2_24h00   Mut  10.56              10.56
## 4 iPS09-82-M2_24h00   Mut  10.53              10.53
## 5 iPS09-82-M2_24h00   Mut  11.17              11.17
```

### 1.1.5 FGF9\_iPS09-M3\_24h00-Wt

```
Input = ("
names   values  block
WT  12.84   iPS09-45-M3_24h00
WT  12.27   iPS09-45-M3_24h00
WT  12.63   iPS09-45-M3_24h00
WT  11.92   iPS09-45-M3_24h00
WT  12.37   iPS09-45-M3_24h00
"
)
Data = read.table(textConnection(Input),header=TRUE)
Data$names = factor(Data$names,ordered=FALSE, levels=unique(Data$names))
Data$block = factor(Data$block,ordered=FALSE, levels=unique(Data$block))

# FGF9_boxplot 1
boxplot(values ~ names,
        data = Data,
        ylab ="values",
        xlab ="names")
```

```
Data8 <- Data |>
  mutate(
    IQR = IQR(values, na.rm = TRUE),
    Outlier_upper = quantile(values, probs = c(.75), na.rm = TRUE) + 1.5 * IQR,
    Outlier_lower = quantile(values, probs = c(.25), na.rm = TRUE) - 1.5 * IQR,
    values_wo_outliers = if_else(values <= Outlier_lower | values >= Outlier_upper, NA, values))

boxplot(values_wo_outliers ~ names, Data8)
```

```
Data8b<- Data8|> select(block, names, values, values_wo_outliers)
Data8b
```

```
##               block names values values_wo_outliers
## 1 iPS09-45-M3_24h00    WT  12.84              12.84
## 2 iPS09-45-M3_24h00    WT  12.27              12.27
## 3 iPS09-45-M3_24h00    WT  12.63              12.63
## 4 iPS09-45-M3_24h00    WT  11.92              11.92
## 5 iPS09-45-M3_24h00    WT  12.37              12.37
```

### 1.1.6 FGF9\_iPS09-M3\_48h00-Wt

```
Input = ("
names   values  block
WT  13.75   iPS09-45-M3_48h00
WT  12.87   iPS09-45-M3_48h00
WT  13.21   iPS09-45-M3_48h00
WT  13.60   iPS09-45-M3_48h00
WT  12.22   iPS09-45-M3_48h00
"
)
Data = read.table(textConnection(Input),header=TRUE)
Data$names = factor(Data$names,ordered=FALSE, levels=unique(Data$names))
Data$block = factor(Data$block,ordered=FALSE, levels=unique(Data$block))

# FGF9_boxplot 1
boxplot(values ~ names,
        data = Data,
        ylab ="values",
        xlab ="names")
```

```
Data9 <- Data |>
  mutate(
    IQR = IQR(values, na.rm = TRUE),
    Outlier_upper = quantile(values, probs = c(.75), na.rm = TRUE) + 1.5 * IQR,
    Outlier_lower = quantile(values, probs = c(.25), na.rm = TRUE) - 1.5 * IQR,
    values_wo_outliers = if_else(values <= Outlier_lower | values >= Outlier_upper, NA, values))

boxplot(values_wo_outliers ~ names, Data9)
```

```
Data9b<- Data9|> select(block, names, values, values_wo_outliers)
Data9b
```

```
##               block names values values_wo_outliers
## 1 iPS09-45-M3_48h00    WT  13.75              13.75
## 2 iPS09-45-M3_48h00    WT  12.87              12.87
## 3 iPS09-45-M3_48h00    WT  13.21              13.21
## 4 iPS09-45-M3_48h00    WT  13.60              13.60
## 5 iPS09-45-M3_48h00    WT  12.22              12.22
```

### 1.1.7 FGF9\_iPS09-M3\_48h00-Mut

```
Input = ("
names   values  block
Mut 13.14   iPS09-82-M3_48h00
Mut 12.01   iPS09-82-M3_48h00
Mut 11.46   iPS09-82-M3_48h00
Mut 12.17   iPS09-82-M3_48h00
Mut 12.36   iPS09-82-M3_48h00
"
)
Data = read.table(textConnection(Input),header=TRUE)
Data$names = factor(Data$names,ordered=FALSE, levels=unique(Data$names))
Data$block = factor(Data$block,ordered=FALSE, levels=unique(Data$block))

# FGF9_boxplot 1
boxplot(values ~ names,
        data = Data,
        ylab ="values",
        xlab ="names")
```

```
Data10 <- Data |>
  mutate(
    IQR = IQR(values, na.rm = TRUE),
    Outlier_upper = quantile(values, probs = c(.75), na.rm = TRUE) + 1.5 * IQR,
    Outlier_lower = quantile(values, probs = c(.25), na.rm = TRUE) - 1.5 * IQR,
    values_wo_outliers = if_else(values <= Outlier_lower | values >= Outlier_upper, NA, values))

boxplot(values_wo_outliers ~ names, Data10)
```

```
Data10b<- Data10|> select(block, names, values, values_wo_outliers)
Data10b
```

```
##               block names values values_wo_outliers
## 1 iPS09-82-M3_48h00   Mut  13.14                 NA
## 2 iPS09-82-M3_48h00   Mut  12.01              12.01
## 3 iPS09-82-M3_48h00   Mut  11.46                 NA
## 4 iPS09-82-M3_48h00   Mut  12.17              12.17
## 5 iPS09-82-M3_48h00   Mut  12.36              12.36
```

## 1.2 FGF9\_iPS12

### 1.2.1 FGF9\_iPS12-M1\_36h00-Wt

```
Input = ("
names   values  block
WT  12.88   iPS12_45_M1_36_P
WT  12.50   iPS12_45_M1_36_P
WT  12.58   iPS12_45_M1_36_P
WT  12.51   iPS12_45_M1_36_P
WT  12.85   iPS12_45_M1_36_P
WT  12.37   iPS12_45_M1_36_P
"
)
Data = read.table(textConnection(Input),header=TRUE)
Data$names = factor(Data$names,ordered=FALSE, levels=unique(Data$names))
Data$block = factor(Data$block,ordered=FALSE, levels=unique(Data$block))

# FGF9_boxplot 1
boxplot(values ~ names,
        data = Data,
        ylab ="values",
        xlab ="names")
```

```
Data13 <- Data |>
  mutate(
    IQR = IQR(values, na.rm = TRUE),
    Outlier_upper = quantile(values, probs = c(.75), na.rm = TRUE) + 1.5 * IQR,
    Outlier_lower = quantile(values, probs = c(.25), na.rm = TRUE) - 1.5 * IQR,
    values_wo_outliers = if_else(values <= Outlier_lower | values >= Outlier_upper, NA, values))

boxplot(values_wo_outliers ~ names, Data13)
```

```
Data13b<- Data13|> select(block, names, values, values_wo_outliers)
Data13b
```

```
##              block names values values_wo_outliers
## 1 iPS12_45_M1_36_P    WT  12.88              12.88
## 2 iPS12_45_M1_36_P    WT  12.50              12.50
## 3 iPS12_45_M1_36_P    WT  12.58              12.58
## 4 iPS12_45_M1_36_P    WT  12.51              12.51
## 5 iPS12_45_M1_36_P    WT  12.85              12.85
## 6 iPS12_45_M1_36_P    WT  12.37              12.37
```

### 1.2.2 FGF9\_iPS12-M1\_36h00-Mut

```
Input = ("
names   values  block
Mut 11.38   iPS12_82_M1_36_P
Mut 11.41   iPS12_82_M1_36_P
Mut 12.00   iPS12_82_M1_36_P
Mut 11.42   iPS12_82_M1_36_P
Mut 11.25   iPS12_82_M1_36_P
Mut 11.36   iPS12_82_M1_36_P
"
)
Data = read.table(textConnection(Input),header=TRUE)
Data$names = factor(Data$names,ordered=FALSE, levels=unique(Data$names))
Data$block = factor(Data$block,ordered=FALSE, levels=unique(Data$block))

# FGF9_boxplot 1
boxplot(values ~ names,
        data = Data,
        ylab ="values",
        xlab ="names")
```

```
Data14 <- Data |>
  mutate(
    IQR = IQR(values, na.rm = TRUE),
    Outlier_upper = quantile(values, probs = c(.75), na.rm = TRUE) + 1.5 * IQR,
    Outlier_lower = quantile(values, probs = c(.25), na.rm = TRUE) - 1.5 * IQR,
    values_wo_outliers = if_else(values <= Outlier_lower | values >= Outlier_upper, NA, values))

boxplot(values_wo_outliers ~ names, Data14)
```

```
Data14b<- Data14|> select(block, names, values, values_wo_outliers)
Data14b
```

```
##              block names values values_wo_outliers
## 1 iPS12_82_M1_36_P   Mut  11.38              11.38
## 2 iPS12_82_M1_36_P   Mut  11.41              11.41
## 3 iPS12_82_M1_36_P   Mut  12.00                 NA
## 4 iPS12_82_M1_36_P   Mut  11.42              11.42
## 5 iPS12_82_M1_36_P   Mut  11.25                 NA
## 6 iPS12_82_M1_36_P   Mut  11.36              11.36
```

### 1.2.3 FGF9\_iPS12-M2\_06h00-Wt

```
Input = ("
names   values  block
WT  13.56   iPS12_45_M2_06_P
WT  13.50   iPS12_45_M2_06_P
WT  13.07   iPS12_45_M2_06_P
WT  13.56   iPS12_45_M2_06_P
WT  13.38   iPS12_45_M2_06_P
WT  NA  iPS12_45_M2_06_P
"
)
Data = read.table(textConnection(Input),header=TRUE)
Data$names = factor(Data$names,ordered=FALSE, levels=unique(Data$names))
Data$block = factor(Data$block,ordered=FALSE, levels=unique(Data$block))

# FGF9_boxplot 1
boxplot(values ~ names,
        data = Data,
        ylab ="values",
        xlab ="names")
```

```
Data15 <- Data |>
  mutate(
    IQR = IQR(values, na.rm = TRUE),
    Outlier_upper = quantile(values, probs = c(.75), na.rm = TRUE) + 1.5 * IQR,
    Outlier_lower = quantile(values, probs = c(.25), na.rm = TRUE) - 1.5 * IQR,
    values_wo_outliers = if_else(values <= Outlier_lower | values >= Outlier_upper, NA, values))

boxplot(values_wo_outliers ~ names, Data15)
```

```
Data15b<- Data15|> select(block, names, values, values_wo_outliers)
Data15b
```

```
##              block names values values_wo_outliers
## 1 iPS12_45_M2_06_P    WT  13.56              13.56
## 2 iPS12_45_M2_06_P    WT  13.50              13.50
## 3 iPS12_45_M2_06_P    WT  13.07                 NA
## 4 iPS12_45_M2_06_P    WT  13.56              13.56
## 5 iPS12_45_M2_06_P    WT  13.38              13.38
## 6 iPS12_45_M2_06_P    WT     NA                 NA
```

### 1.2.4 FGF9\_iPS12-M2\_06h00-Mut

```
Input = ("
names   values  block
Mut 11.97   iPS12_82_M2_06_P
Mut 11.82   iPS12_82_M2_06_P
Mut 11.85   iPS12_82_M2_06_P
Mut 11.93   iPS12_82_M2_06_P
Mut 12.27   iPS12_82_M2_06_P
Mut 12.24   iPS12_82_M2_06_P
"
)
Data = read.table(textConnection(Input),header=TRUE)
Data$names = factor(Data$names,ordered=FALSE, levels=unique(Data$names))
Data$block = factor(Data$block,ordered=FALSE, levels=unique(Data$block))

# FGF9_boxplot 1
boxplot(values ~ names,
        data = Data,
        ylab ="values",
        xlab ="names")
```

```
Data16 <- Data |>
  mutate(
    IQR = IQR(values, na.rm = TRUE),
    Outlier_upper = quantile(values, probs = c(.75), na.rm = TRUE) + 1.5 * IQR,
    Outlier_lower = quantile(values, probs = c(.25), na.rm = TRUE) - 1.5 * IQR,
    values_wo_outliers = if_else(values <= Outlier_lower | values >= Outlier_upper, NA, values))

boxplot(values_wo_outliers ~ names, Data16)
```

```
Data16b<- Data16|> select(block, names, values, values_wo_outliers)
Data16b
```

```
##              block names values values_wo_outliers
## 1 iPS12_82_M2_06_P   Mut  11.97              11.97
## 2 iPS12_82_M2_06_P   Mut  11.82              11.82
## 3 iPS12_82_M2_06_P   Mut  11.85              11.85
## 4 iPS12_82_M2_06_P   Mut  11.93              11.93
## 5 iPS12_82_M2_06_P   Mut  12.27              12.27
## 6 iPS12_82_M2_06_P   Mut  12.24              12.24
```

### 1.2.5 FGF9\_iPS12-M2\_12h00-Wt

```
Input = ("
names   values  block
WT  13.69   iPS12_45_M2_12_P
WT  12.88   iPS12_45_M2_12_P
WT  12.89   iPS12_45_M2_12_P
WT  13.41   iPS12_45_M2_12_P
WT  12.92   iPS12_45_M2_12_P
WT  13.12   iPS12_45_M2_12_P
"
)
Data = read.table(textConnection(Input),header=TRUE)
Data$names = factor(Data$names,ordered=FALSE, levels=unique(Data$names))
Data$block = factor(Data$block,ordered=FALSE, levels=unique(Data$block))

# FGF9_boxplot 1
boxplot(values ~ names,
        data = Data,
        ylab ="values",
        xlab ="names")
```

```
Data17 <- Data |>
  mutate(
    IQR = IQR(values, na.rm = TRUE),
    Outlier_upper = quantile(values, probs = c(.75), na.rm = TRUE) + 1.5 * IQR,
    Outlier_lower = quantile(values, probs = c(.25), na.rm = TRUE) - 1.5 * IQR,
    values_wo_outliers = if_else(values <= Outlier_lower | values >= Outlier_upper, NA, values))

boxplot(values_wo_outliers ~ names, Data17)
```

```
Data17b<- Data17|> select(block, names, values, values_wo_outliers)
Data17b
```

```
##              block names values values_wo_outliers
## 1 iPS12_45_M2_12_P    WT  13.69              13.69
## 2 iPS12_45_M2_12_P    WT  12.88              12.88
## 3 iPS12_45_M2_12_P    WT  12.89              12.89
## 4 iPS12_45_M2_12_P    WT  13.41              13.41
## 5 iPS12_45_M2_12_P    WT  12.92              12.92
## 6 iPS12_45_M2_12_P    WT  13.12              13.12
```

### 1.2.6 FGF9\_iPS12-M2\_12h00-Mut

```
Input = ("
names   values  block
Mut 11.23   iPS12_82_M2_12_P
Mut 11.14   iPS12_82_M2_12_P
Mut 11.79   iPS12_82_M2_12_P
Mut 11.10   iPS12_82_M2_12_P
Mut 11.55   iPS12_82_M2_12_P
Mut 11.26   iPS12_82_M2_12_P
"
)
Data = read.table(textConnection(Input),header=TRUE)
Data$names = factor(Data$names,ordered=FALSE, levels=unique(Data$names))
Data$block = factor(Data$block,ordered=FALSE, levels=unique(Data$block))

# FGF9_boxplot 1
boxplot(values ~ names,
        data = Data,
        ylab ="values",
        xlab ="names")
```

```
Data18 <- Data |>
  mutate(
    IQR = IQR(values, na.rm = TRUE),
    Outlier_upper = quantile(values, probs = c(.75), na.rm = TRUE) + 1.5 * IQR,
    Outlier_lower = quantile(values, probs = c(.25), na.rm = TRUE) - 1.5 * IQR,
    values_wo_outliers = if_else(values <= Outlier_lower | values >= Outlier_upper, NA, values))

boxplot(values_wo_outliers ~ names, Data18)
```

```
Data18b<- Data18|> select(block, names, values, values_wo_outliers)
Data18b
```

```
##              block names values values_wo_outliers
## 1 iPS12_82_M2_12_P   Mut  11.23              11.23
## 2 iPS12_82_M2_12_P   Mut  11.14              11.14
## 3 iPS12_82_M2_12_P   Mut  11.79              11.79
## 4 iPS12_82_M2_12_P   Mut  11.10              11.10
## 5 iPS12_82_M2_12_P   Mut  11.55              11.55
## 6 iPS12_82_M2_12_P   Mut  11.26              11.26
```

### 1.2.7 FGF9\_iPS12\_M2\_24h00-Wt

```
Input = ("
names   values  block
WT  13.20   iPS12_45_M2_24_P
WT  13.68   iPS12_45_M2_24_P
WT  13.14   iPS12_45_M2_24_P
WT  13.34   iPS12_45_M2_24_P
WT  13.22   iPS12_45_M2_24_P
WT  14.48   iPS12_45_M2_24_P
"
)
Data = read.table(textConnection(Input),header=TRUE)
Data$names = factor(Data$names,ordered=FALSE, levels=unique(Data$names))
Data$block = factor(Data$block,ordered=FALSE, levels=unique(Data$block))

# FGF9_boxplot 1
boxplot(values ~ names,
        data = Data,
        ylab ="values",
        xlab ="names")
```

```
Data19 <- Data |>
  mutate(
    IQR = IQR(values, na.rm = TRUE),
    Outlier_upper = quantile(values, probs = c(.75), na.rm = TRUE) + 1.5 * IQR,
    Outlier_lower = quantile(values, probs = c(.25), na.rm = TRUE) - 1.5 * IQR,
    values_wo_outliers = if_else(values <= Outlier_lower | values >= Outlier_upper, NA, values))

boxplot(values_wo_outliers ~ names, Data19)
```

```
Data19b<- Data19|> select(block, names, values, values_wo_outliers)
Data19b
```

```
##              block names values values_wo_outliers
## 1 iPS12_45_M2_24_P    WT  13.20              13.20
## 2 iPS12_45_M2_24_P    WT  13.68              13.68
## 3 iPS12_45_M2_24_P    WT  13.14              13.14
## 4 iPS12_45_M2_24_P    WT  13.34              13.34
## 5 iPS12_45_M2_24_P    WT  13.22              13.22
## 6 iPS12_45_M2_24_P    WT  14.48                 NA
```

### 1.2.8 FGF9\_iPS12\_M2\_24h00-Mut

```
Input = ("
names   values  block
Mut 12.18   iPS12_82_M2_24_P
Mut 11.90   iPS12_82_M2_24_P
Mut 12.24   iPS12_82_M2_24_P
Mut 11.95   iPS12_82_M2_24_P
Mut 11.85   iPS12_82_M2_24_P
Mut 12.25   iPS12_82_M2_24_P
"
)
Data = read.table(textConnection(Input),header=TRUE)
Data$names = factor(Data$names,ordered=FALSE, levels=unique(Data$names))
Data$block = factor(Data$block,ordered=FALSE, levels=unique(Data$block))

# FGF9_boxplot 1
boxplot(values ~ names,
        data = Data,
        ylab ="values",
        xlab ="names")
```

```
Data20 <- Data |>
  mutate(
    IQR = IQR(values, na.rm = TRUE),
    Outlier_upper = quantile(values, probs = c(.75), na.rm = TRUE) + 1.5 * IQR,
    Outlier_lower = quantile(values, probs = c(.25), na.rm = TRUE) - 1.5 * IQR,
    values_wo_outliers = if_else(values <= Outlier_lower | values >= Outlier_upper, NA, values))

boxplot(values_wo_outliers ~ names, Data20)
```

```
Data20b<- Data20|> select(block, names, values, values_wo_outliers)
Data20b
```

```
##              block names values values_wo_outliers
## 1 iPS12_82_M2_24_P   Mut  12.18              12.18
## 2 iPS12_82_M2_24_P   Mut  11.90              11.90
## 3 iPS12_82_M2_24_P   Mut  12.24              12.24
## 4 iPS12_82_M2_24_P   Mut  11.95              11.95
## 5 iPS12_82_M2_24_P   Mut  11.85              11.85
## 6 iPS12_82_M2_24_P   Mut  12.25              12.25
```

### 1.2.9 FGF9\_iPS12-M2\_48h00-Wt

```
Input = ("
names   values  block
WT  12.55   iPS12_45_M2_48_P
WT  13.25   iPS12_45_M2_48_P
WT  12.86   iPS12_45_M2_48_P
WT  12.59   iPS12_45_M2_48_P
WT  13.29   iPS12_45_M2_48_P
WT  NA  iPS12_45_M2_48_P
"
)
Data = read.table(textConnection(Input),header=TRUE)
Data$names = factor(Data$names,ordered=FALSE, levels=unique(Data$names))
Data$block = factor(Data$block,ordered=FALSE, levels=unique(Data$block))

# FGF9_boxplot 1
boxplot(values ~ names,
        data = Data,
        ylab ="values",
        xlab ="names")
```

```
Data21 <- Data |>
  mutate(
    IQR = IQR(values, na.rm = TRUE),
    Outlier_upper = quantile(values, probs = c(.75), na.rm = TRUE) + 1.5 * IQR,
    Outlier_lower = quantile(values, probs = c(.25), na.rm = TRUE) - 1.5 * IQR,
    values_wo_outliers = if_else(values <= Outlier_lower | values >= Outlier_upper, NA, values))

boxplot(values_wo_outliers ~ names, Data21)
```

```
Data21b<- Data21|> select(block, names, values, values_wo_outliers)
Data21b
```

```
##              block names values values_wo_outliers
## 1 iPS12_45_M2_48_P    WT  12.55              12.55
## 2 iPS12_45_M2_48_P    WT  13.25              13.25
## 3 iPS12_45_M2_48_P    WT  12.86              12.86
## 4 iPS12_45_M2_48_P    WT  12.59              12.59
## 5 iPS12_45_M2_48_P    WT  13.29              13.29
## 6 iPS12_45_M2_48_P    WT     NA                 NA
```

### 1.2.10 FGF9\_iPS12-M2\_48h00-Mut

```
Input = ("
names   values  block
Mut 11.57   iPS12_82_M2_48_P
Mut 11.96   iPS12_82_M2_48_P
Mut 12.34   iPS12_82_M2_48_P
Mut 11.64   iPS12_82_M2_48_P
Mut 12.02   iPS12_82_M2_48_P
Mut 12.01   iPS12_82_M2_48_P
"
)
Data = read.table(textConnection(Input),header=TRUE)
Data$names = factor(Data$names,ordered=FALSE, levels=unique(Data$names))
Data$block = factor(Data$block,ordered=FALSE, levels=unique(Data$block))

# FGF9_boxplot 1
boxplot(values ~ names,
        data = Data,
        ylab ="values",
        xlab ="names")
```

```
Data22 <- Data |>
  mutate(
    IQR = IQR(values, na.rm = TRUE),
    Outlier_upper = quantile(values, probs = c(.75), na.rm = TRUE) + 1.5 * IQR,
    Outlier_lower = quantile(values, probs = c(.25), na.rm = TRUE) - 1.5 * IQR,
    values_wo_outliers = if_else(values <= Outlier_lower | values >= Outlier_upper, NA, values))

boxplot(values_wo_outliers ~ names, Data22)
```

```
Data22b<- Data22|> select(block, names, values, values_wo_outliers)
Data22b
```

```
##              block names values values_wo_outliers
## 1 iPS12_82_M2_48_P   Mut  11.57              11.57
## 2 iPS12_82_M2_48_P   Mut  11.96              11.96
## 3 iPS12_82_M2_48_P   Mut  12.34              12.34
## 4 iPS12_82_M2_48_P   Mut  11.64              11.64
## 5 iPS12_82_M2_48_P   Mut  12.02              12.02
## 6 iPS12_82_M2_48_P   Mut  12.01              12.01
```

## 1.3 FGF9\_iPS19

### 1.3.1 FGF9\_iPS19-iPS-Wt

```
Input = ("
names   values  block
WT  14.63   iPS19_45_iPS
WT  14.54   iPS19_45_iPS
WT  14.30   iPS19_45_iPS
WT  14.83   iPS19_45_iPS
WT  14.59   iPS19_45_iPS
WT  14.32   iPS19_45_iPS
WT  13.97   iPS19_82_iPS
WT  14.13   iPS19_82_iPS
WT  13.91   iPS19_82_iPS
WT  14.54   iPS19_82_iPS
WT  14.19   iPS19_82_iPS
WT  13.80   iPS19_82_iPS
"
)
Data = read.table(textConnection(Input),header=TRUE)
Data$names = factor(Data$names,ordered=FALSE, levels=unique(Data$names))
Data$block = factor(Data$block,ordered=FALSE, levels=unique(Data$block))

# SRY_boxplot 1
boxplot(values ~ names,
        data = Data,
        ylab ="values",
        xlab ="names")
```

```
Data23 <- Data |>
  mutate(
    IQR = IQR(values, na.rm = TRUE),
    Outlier_upper = quantile(values, probs = c(.75), na.rm = TRUE) + 1.5 * IQR,
    Outlier_lower = quantile(values, probs = c(.25), na.rm = TRUE) - 1.5 * IQR,
    values_wo_outliers = if_else(values <= Outlier_lower | values >= Outlier_upper, NA, values))

boxplot(values_wo_outliers ~ names, Data23)
```

```
Data23b<- Data23|> select(block, names, values, values_wo_outliers)
Data23b
```

```
##           block names values values_wo_outliers
## 1  iPS19_45_iPS    WT  14.63              14.63
## 2  iPS19_45_iPS    WT  14.54              14.54
## 3  iPS19_45_iPS    WT  14.30              14.30
## 4  iPS19_45_iPS    WT  14.83              14.83
## 5  iPS19_45_iPS    WT  14.59              14.59
## 6  iPS19_45_iPS    WT  14.32              14.32
## 7  iPS19_82_iPS    WT  13.97              13.97
## 8  iPS19_82_iPS    WT  14.13              14.13
## 9  iPS19_82_iPS    WT  13.91              13.91
## 10 iPS19_82_iPS    WT  14.54              14.54
## 11 iPS19_82_iPS    WT  14.19              14.19
## 12 iPS19_82_iPS    WT  13.80              13.80
```

### 1.3.2 FGF9\_iPS19-iPS-Mut

```
Input = ("
names   values  block
Mut 15.02   iPS19_82_iPS
Mut 15.12   iPS19_82_iPS
Mut 15.04   iPS19_82_iPS
Mut 14.46   iPS19_82_iPS
Mut 14.84   iPS19_82_iPS
Mut 14.56   iPS19_82_iPS
Mut 14.66   iPS19_82_iPS
Mut 14.54   iPS19_82_iPS
Mut 14.64   iPS19_82_iPS
Mut 14.34   iPS19_82_iPS
Mut 14.53   iPS19_82_iPS
Mut 14.56   iPS19_82_iPS
"
)
Data = read.table(textConnection(Input),header=TRUE)
Data$names = factor(Data$names,ordered=FALSE, levels=unique(Data$names))
Data$block = factor(Data$block,ordered=FALSE, levels=unique(Data$block))

# SRY_boxplot 1
boxplot(values ~ names,
        data = Data,
        ylab ="values",
        xlab ="names")
```

```
Data24 <- Data |>
  mutate(
    IQR = IQR(values, na.rm = TRUE),
    Outlier_upper = quantile(values, probs = c(.75), na.rm = TRUE) + 1.5 * IQR,
    Outlier_lower = quantile(values, probs = c(.25), na.rm = TRUE) - 1.5 * IQR,
    values_wo_outliers = if_else(values <= Outlier_lower | values >= Outlier_upper, NA, values))

boxplot(values_wo_outliers ~ names, Data24)
```

```
Data24b<- Data24|> select(block, names, values, values_wo_outliers)
Data24b
```

```
##           block names values values_wo_outliers
## 1  iPS19_82_iPS   Mut  15.02              15.02
## 2  iPS19_82_iPS   Mut  15.12              15.12
## 3  iPS19_82_iPS   Mut  15.04              15.04
## 4  iPS19_82_iPS   Mut  14.46              14.46
## 5  iPS19_82_iPS   Mut  14.84              14.84
## 6  iPS19_82_iPS   Mut  14.56              14.56
## 7  iPS19_82_iPS   Mut  14.66              14.66
## 8  iPS19_82_iPS   Mut  14.54              14.54
## 9  iPS19_82_iPS   Mut  14.64              14.64
## 10 iPS19_82_iPS   Mut  14.34              14.34
## 11 iPS19_82_iPS   Mut  14.53              14.53
## 12 iPS19_82_iPS   Mut  14.56              14.56
```
